# Exploring DHPM-azole hybrid molecules for antibacterial drug discovery: synthesis and biological assessment

**DOI:** 10.1039/d6ra05427g

**Published:** 2026-08-05

**Authors:** Gobind Kumar, Safiyah Ghumran, Neha Manhas, Pule Seboletswe, Afsana Kajee, Asif Raza, Arun K. Sharma, Parvesh Singh

**Affiliations:** a Discipline of Chemistry, University of KwaZulu-Natal P/Bag X54001, Westville Durban 4000 South Africa singhp4@ukzn.ac.za; b Department of Microbiology, National Health Laboratory Services (NHLS), Inkosi Albert Luthuli Central Hospital Durban South Africa; c Department of Pharmacology, Penn State Cancer Institute, Penn State College of Medicine, 500 University Drive CH72 Hershey PA 17033 USA asharma1@pennstatehealth.psu.edu

## Abstract

A novel series of pyrimidine-based *N*-heterocyclic hybrids incorporating 1,2,4-triazole (10a–10j) and benzotriazole (11a–11j) moieties was synthesized and evaluated for antibacterial activity against a panel of Gram-positive and Gram-negative bacterial pathogens. The structures of all synthesized compounds were confirmed by FT-IR, ^1^H NMR, ^13^C NMR, and HRMS analyses. Several derivatives exhibited potent antibacterial activity against Gram-positive strains, including *Bacillus subtilis*, *Staphylococcus aureus*, and methicillin-resistant *S. aureus* (MRSA), as well as Gram-negative bacteria such as *Salmonella typhi*, *Escherichia coli*, *Pseudomonas aeruginosa*, and *Klebsiella pneumoniae*. Among them, compounds 10b, 10d, 11b, and 11i displayed pronounced activity against Gram-positive strains, with MIC values ranging from 0.78 to 6.25 µg mL^−1^. Particularly, compounds 10d and 11b exhibited potent anti-MRSA activity, with MIC values of 0.78 and 3.12 µg mL^−1^, respectively. Furthermore, compounds 10b, 10c, 10d, 10e, 10h, and 11i demonstrated promising activity against *S. typhi* and *E. coli*, with MIC values ranging from 0.39 to 12.50 µg mL^−1^. Cytotoxicity studies against HaCaT, HEK293T, and FHC normal cell lines revealed low toxicity, indicating a favorable safety profile. *In silico* ADME predictions further suggested desirable drug-like characteristics, including high gastrointestinal absorption and limited blood–brain barrier penetration. Collectively, these findings identify pyrimidine-triazole hybrids as promising antimicrobial lead scaffolds for the development of new antibacterial agents.

## Abstract 2

Molecular hybridization of dihydropyrimidinones with 1,2,4-triazole and benzotriazole afforded potent antibacterial agents active against MRSA and other bacterial pathogens, with low cytotoxicity and promising *in silico* pharmacokinetic profiles.

## Graphical abstract

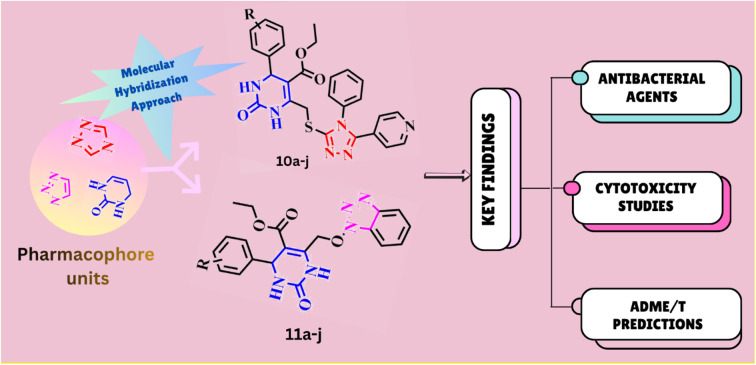

## Introduction

The dawn of antibiotics is undoubtedly a paradigm shift in human history, and antibiotics are as important to human health today as they were almost a century ago.^1^ The distinction between the two eras lies in the fact that antibiotics, while still effective to an extent, are not as potent against microbes as they were when they were first introduced.^2^ This is due to antimicrobial resistance (AMR), an evolutionary phenomenon by which a microorganism gains resistance to a compound to which it was once susceptible.^3^ AMR is a natural reaction of microorganisms to their environment to increase their chances of survival; however, due to the improper use of antibiotics, AMR has become exacerbated.^4,5^ Improper use of antibiotics can be the use of poor-quality drugs, self-medication, as well as the extended and/or unnecessary use of antibiotics in both humans and animals, especially livestock animals.^6–9^

Bacterial resistance is a global health threat, with approximately 7.7 million deaths annually resulting from bacterial infections, of which almost 5 million are linked to drug-resistant strains of bacteria.^10,11^ The World Health Organization (WHO) has classified seven pathogens as significant threats to human health due to their growing drug resistance and the critical need for effective therapies against *Escherichia coli*^12^ and the ESKAPE pathogens, consisting of *Enterococcus faecium*,*Staphylococcus aureus*,*Klebsiella pneumoniae*,*Acinetobacter baumannii*,*Pseudomonas aeruginosa*, and *Enterobacter* spp.^13^ The aforementioned pathogens can survive and/or thrive in healthcare environments due to their intrinsic or acquired resistance, resulting in approximately 330 000 deaths in 2019 alone.^13^ Many of the pathogens listed are Gram-negative bacteria, and as a result of their distinct structure (a protective membrane encasing the cell), they are more likely to develop resistance than Gram-positive bacteria.^14^ AMR microbes extend beyond healthcare environments into communities and the food chain, facilitating their global dissemination. As a result, AMR threatens not only vulnerable and immunocompromised individuals but also the broader population, representing a major global public health concern.^15^ One of the five intervention strategies to combat bacterial AMR is the investment into the development of new antibiotics, indicating the importance of developing new antibiotics, especially one active against *E. coli* and the ESKAPE pathogens.^16^

**Fig. 1:**
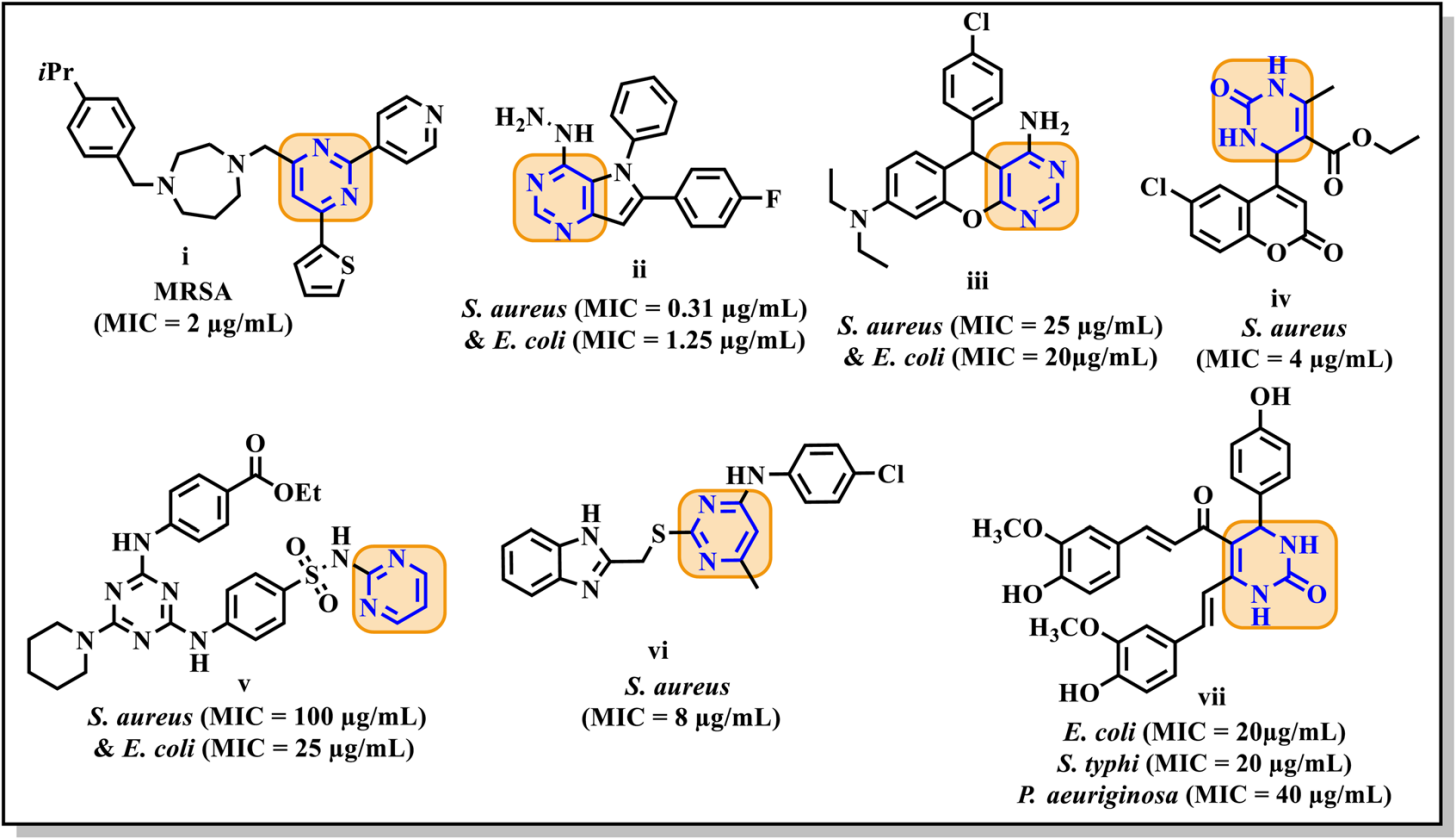
A representation of some potent antibacterial agents containing an *N*-heterocyclic pharmacophore.

Heterocycles, especially pyrimidines, the six-membered heterocyclic rings containing nitrogen, are considered a privileged pharmacophore, with pyrimidine derivatives being reported to have vast biological uses as an antimicrobial,^17^ antiviral,^18^ anticancer,^19^ and anti-inflammatory.^20^ A plethora of market drugs contain pyrimidine in their structure, further illustrating the value of this moiety in medicinal chemistry.^21^Fig. 1 represents the various antibacterial agents containing a pyrimidine scaffold.

Heterocyclic compounds play a vital role in medicinal chemistry, particularly in the development of therapeutic agents. Among them, azole-containing heterocycles have attracted considerable attention because of their broad-spectrum antimicrobial activities. Numerous azole derivatives have been successfully developed as antibacterial and antifungal drugs and are widely used in clinical practice.^22–24^ Representative examples include the imidazole derivatives metronidazole and sulconazole, the triazole derivative Fluconazole, the tetrazole-containing antibiotics ceforanide and cefoperazone, the thiazole-containing antibiotics ceftizoxime and cefdinir, oxazole-based oxazolidinones, and the pyrazole derivative sulfaphenazole. These clinically important agents highlight the therapeutic significance of azole scaffolds and have stimulated continued interest in the design and development of new antimicrobial compounds incorporating azole moieties.^25,26^

Among the various azole derivatives, 1,2,4-triazoles and benzotriazoles represent important classes of five-membered and fused five-membered nitrogen-containing heterocycles, respectively, each incorporating three nitrogen atoms within the ring system. They are also considered bioisosteres of biologically active heterocycles, particularly imidazole,^27^ quinoline,^28^ and indole,^29^ thereby contributing to improved pharmacological and physicochemical properties. Triazole-containing compounds are present in numerous clinically used drugs and exhibit a broad spectrum of biological activities, including antioxidant,^30^ antiviral,^31^ anticancer,^32^ anti-inflammatory,^33^ antibacterial,^34,35^ and antifungal effects.^36^

**Fig. 2:**
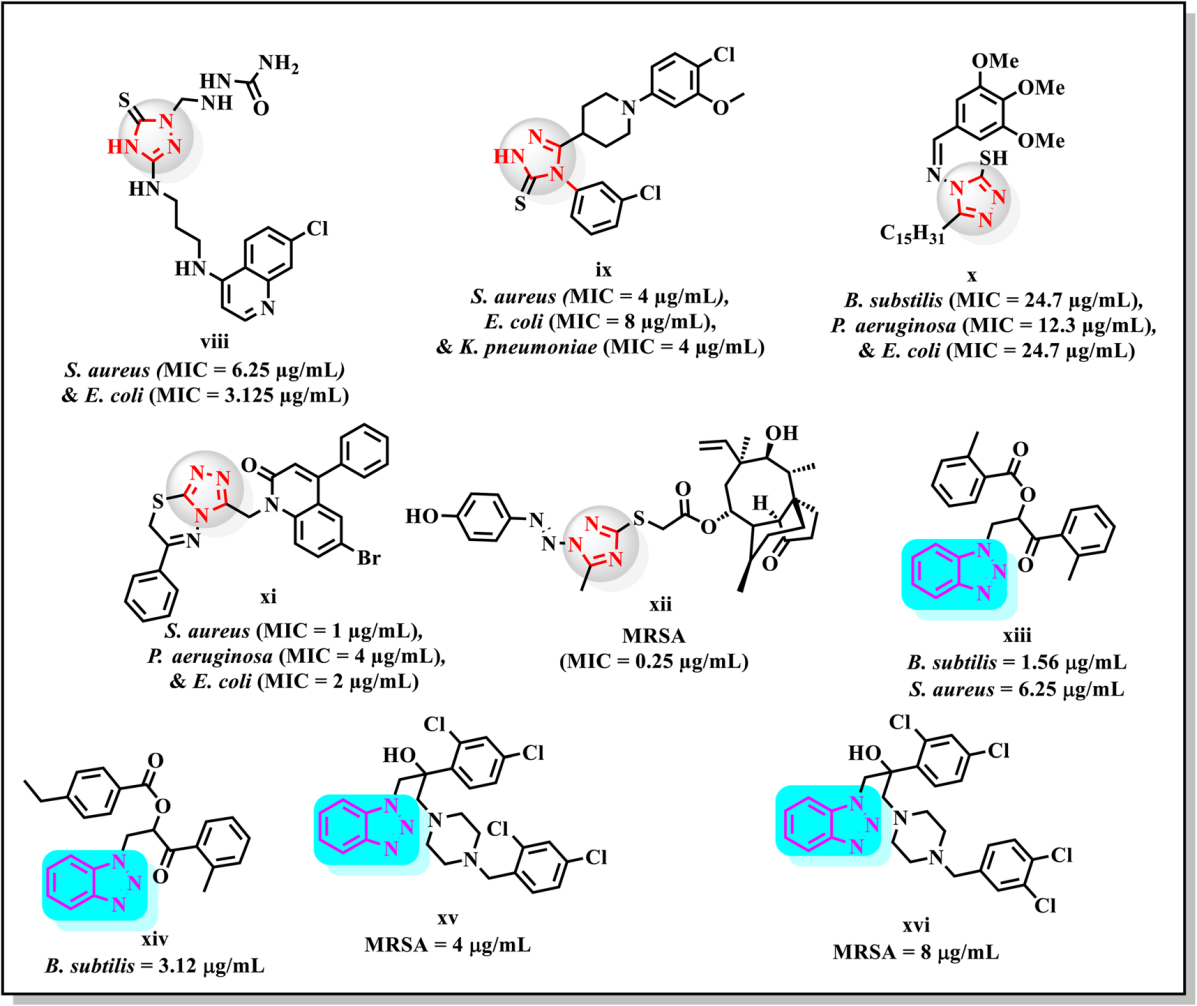
Representative examples of potent antibacterial agents featuring 1,2,4-triazole and benzotriazole pharmacophores are reported in the literature.

Notably, both 1,2,4-triazole and benzotriazole compounds have been extensively investigated for their antibacterial potential. Several reported compounds have demonstrated potent activity against a variety of Gram-positive and Gram-negative bacterial strains, including multidrug-resistant pathogens, as summarized in Fig. 2.^37–52^

A method of drug development that has recently shown considerable promise is molecular hybridization (MH), which involves the combination of two or more pharmacophoric moieties into a single hybrid molecule. Such hybrids are expected to retain the beneficial properties of the parent compounds while enhancing biological activity, overcoming drug resistance, and reducing toxicity and adverse side effects.^12^ In addition, hybrid molecules can interact with multiple biological targets simultaneously and may therefore be useful in the management of co-infections.^53^

**Fig. 3:**
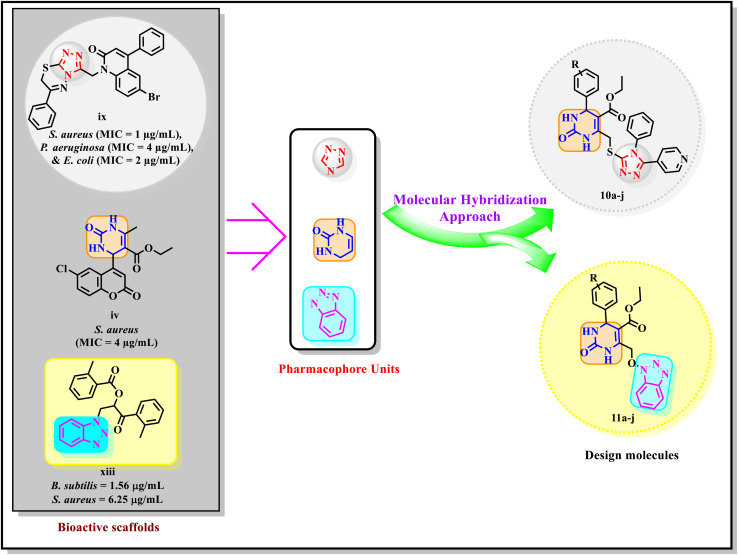
Schematic representation of DHPM-azole hybrids design through molecular hybridization for antibacterial activity.

Thus, considering the well-established pharmacological significance of triazoles and pyrimidines, a series of novel pyrimidine–azole molecular hybrids (10a–j and 11a–j, Fig. 3) were designed and synthesized through an MH approach. The synthesized compounds were structurally characterized using various spectroscopic techniques. Subsequently, the compounds were evaluated for their antibacterial activity against a panel of Gram-positive and Gram-negative bacterial strains, followed by structure–activity relationship analysis. In addition, representative compounds were evaluated for cytotoxicity against three normal cell lines, HaCaT, HEK293T, and FHC, to determine their selectivity indices. Furthermore, the physicochemical properties and ADMET parameters of all synthesized compounds were predicted *in silico* to provide a comprehensive assessment of their drug-likeness and pharmacokinetic potential.

## Results and discussion

### Synthetic chemistry

**Scheme 1:**
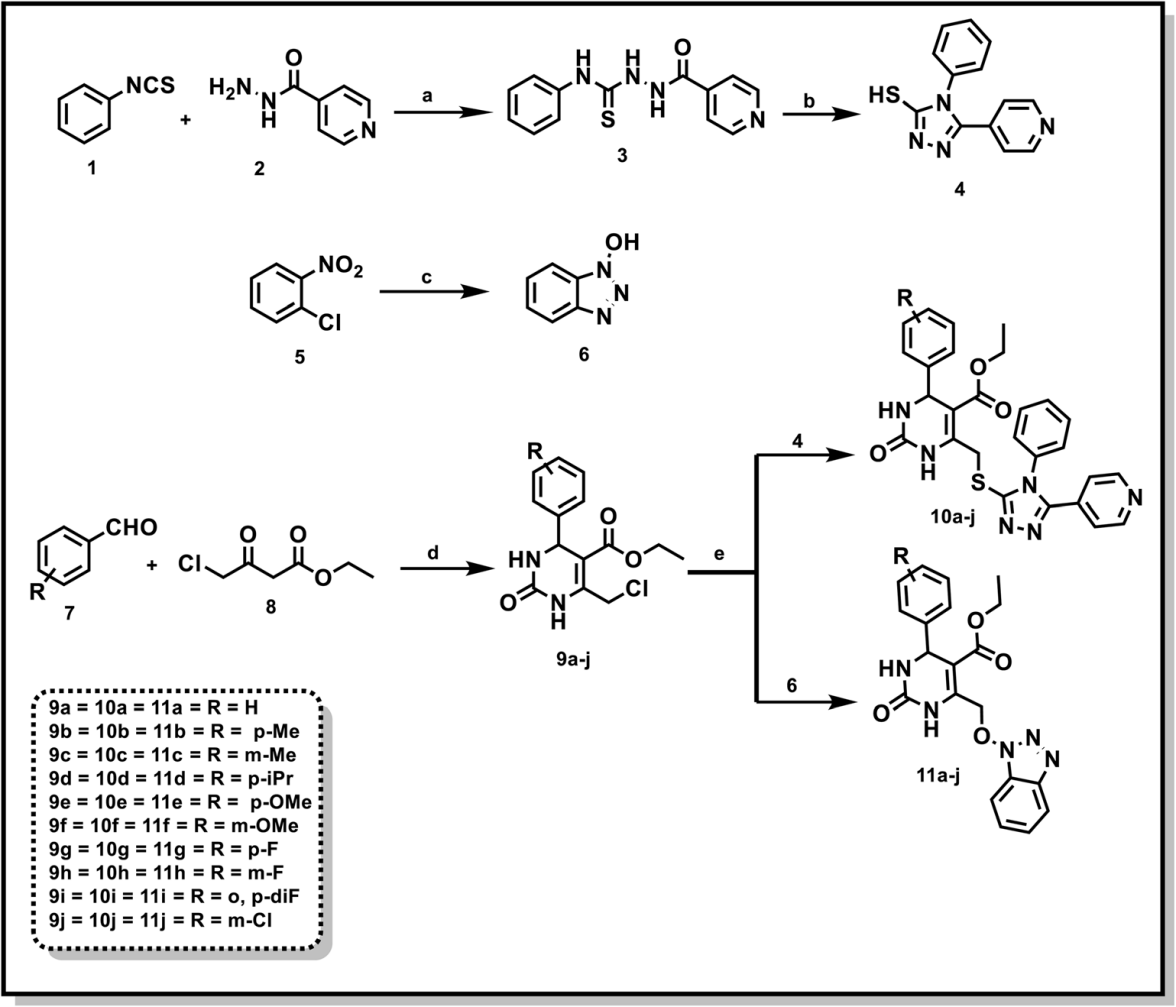
Illustration of the synthesis of molecular hybrids 10a–j and 11a–j. Reaction conditions: (a) ethanol, reflux for 5–15 min; (b) 4 N NaOH solution, reflux for 2–3 hours; (c) hydrazine hydrate (5 eq.), 1-heptanol, 110–120 °C; (d) urea (2 eq.) and conc. HCl 2–3 drops, heated at 100 °C for 2 hours; (e) anhydrous K_2_CO_3_ (2.5 eq.), CH_3_CN, refluxed at 80 °C for 30 minutes.

The synthesis technique for the intermediates and final compounds is illustrated in Scheme 1. Intermediate 4 was synthesized according to a previously reported procedure.^54^ Briefly, refluxing of phenyl isothiocyanate 1 with isoniazid 2 in ethanol for 5–10 minutes afforded the substituted thiosemicarbazide 3. Afterwards, the substituted thiosemicarbazide 3 was treated with a 4 N NaOH solution for 2–5 hours to yield intermediate 4. Intermediate 6 was prepared by heating 2-nitrochlorobenzene 5 with hydrazine hydrate in 1-heptanol at 110–120 °C for 5 hours.^55^ The series of intermediates 9a–j was also synthesized according to the previously reported method.^56^ In the first step, various substituted aromatic aldehydes 7a–j were utilized for the synthesis of 6-chloromethyldihydropyrimidinones 9a–j (Scheme 1). The molecular hybrids 10a–j and 11a–j were synthesized by treating 9a–j with 4 and 7, respectively, using anhydrous K_2_CO_3_ as a base in refluxing CH_3_CN for 30 minutes.

### Spectroscopic characterization

The synthetic molecular hybrids were elucidated using various spectroscopic techniques such as ^1^H-NMR, ^13^C-NMR, infrared radiation (IR), and high-resolution mass spectrometry (HRMS). Moreover, the two-dimensional (2D) NMR (HSQC and HMBC) data were used to explore the complete structure assignment of the representative compounds 10b and 11b.

For example, in series 10, the IR spectrum of 10b showed important characteristic absorbances at *ν* 3360 and 3199 cm^−1^ for N–H stretching. The carbonyl groups (C=O) of ester and amide displayed absorbance at 1690 and 1636 cm^−1^. The absorbance at 1603 cm^−1^ represented alkene stretching (C=C). In its ^1^H NMR spectrum, the pyrimidine ring displayed two NH protons, NH-1 and NH-3, resonating as two singlet peaks far downfield at *δ*_H_ 7.79 and 9.21 ppm, respectively. A characteristic singlet at *δ*_H_ 5.78 ppm for the CH-4 proton confirmed the formation of the pyrimidine ring moiety. Furthermore, as expected, the methyl groups H-10 and H-17 appeared as the most upfield resonances at *δ*_H_ 1.03 (triplet) and 2.25 ppm (singlet), respectively. The CH_2_ (H-18) protons showed two doublets at 5.06 ppm and 5.48 ppm. The pyridine ring displayed doublets for aromatic protons at 8.58 ppm (H-33, H-35) and 7.29 ppm (H-32, H-36). The pyrimidine ring carbon C-2 (C=O) appeared at 152.26 ppm in its ^13^C spectrum, along with the most upfield signals at 14.35 and 21.11 ppm corresponding to C-10 and C-17, respectively. The carbonyl carbon (C-7) displayed the most downfield resonance at 165.15 ppm. The quaternary carbons C-20 and C-23 present in the 1,2,4-triazole ring appeared at 152.80 ppm and 153.05 ppm, respectively. Finally, the HRMS spectrum of compound 10b exhibited a molecular ion peak of *m*/*z* 549.16 and further confirmed the assigned structure.

**Fig. 4:**
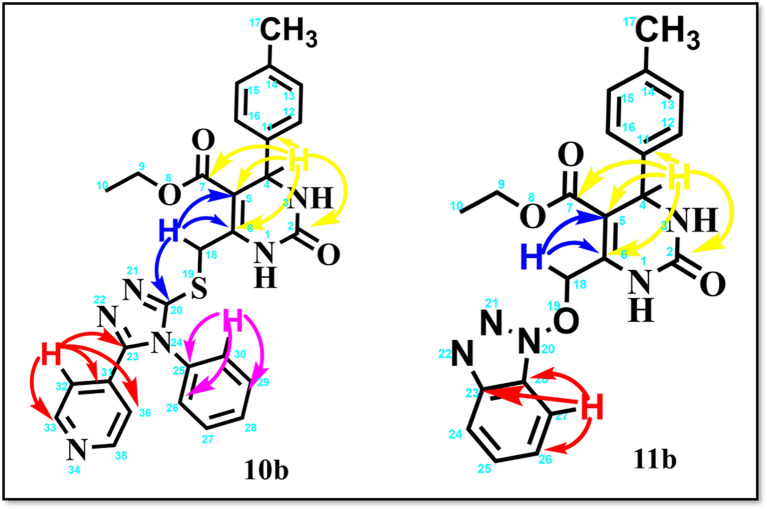
Key HMBC interactions were observed for the representative 1,2,4-triazole derivative 10b and benzotriazole derivative 11b. The highlighted long-range ^1^H and ^13^C correlations confirm the connectivity of the DHPM scaffold with the respective azole moieties and substituted aromatic rings, providing spectroscopic evidence for the successful synthesis and structural assignment of the target hybrid compounds.

The key carbon assignments of compound 10b were further established using Heteronuclear Multiple Bond Coherence (HMBC) spectroscopy (Fig. 4). The pyridine proton H-32 (*δ*_H_ 7.29 ppm) showed HMBC correlations with C-23 (*δ*_C_ 153.05 ppm), C-31 (*δ*_C_ 134.36 ppm), C-33 (*δ*_C_ 153.66 ppm), and C-36 (*δ*_C_ 121.94). Similarly, H-30 (*δ*_H_ 7.48 ppm) showed HMBC correlations with C-25 (*δ*_C_ 134.09 ppm), C-26 (*δ*_C_ 130.94 ppm), and C-29 (*δ*_C_ 130.09 pm). Proton H-18 displayed correlations with C-5 (*δ*_C_ 101.63 ppm), C-6 (*δ*_C_ 147.11 ppm), and C-20 (*δ*_C_ 152.80 ppm). In addition, proton H-4 showed HMBC correlations with multiple carbons, namely C-2 (*δ*_C_ 152.26 ppm), C-5 (*δ*_C_ 101.63 ppm), C-6 (*δ*_C_ 147.11 ppm), C-7 (*δ*_C_ 165.15 ppm), and C-11 (*δ*_C_ 141.72 ppm) (Fig. 4), thereby supporting the proposed structure of compound 10b.

In series 11, the IR spectrum of compound 11b showed four characteristic absorption bands: at *ν* 3236 and 3126 cm^−1^ corresponding to N–H stretching, and at 1702 and 1657 cm^−1^ attributed to the carbonyl groups (C=O) of ester and amide functionalities. In the ^1^H NMR spectrum, the pyrimidine ring exhibited two NH protons (NH-1 and NH-3), which appeared as downfield singlets at *δ*_H_ 7.77 and 9.76 ppm, respectively. A distinctive doublet at *δ*_H_ 5.06 ppm, corresponding to the CH-4 proton, confirmed the formation of the pyrimidine moiety. As expected, the methyl groups H-10 and H-17 resonated upfield, appearing at *δ*_H_ 0.92 ppm (triplet) and 2.25 ppm (singlet). The CH_2_ protons (H-18) were observed as two doublets at *δ*_H_ 5.48 and 5.73 ppm. In the ^13^C NMR spectrum, the pyrimidine carbon C-2 appeared at *δ*_C_ 152.47 ppm. The most upfield signals were assigned to the methyl carbons C-10 and C-17 at *δ*_C_ 14.13 and 21.11 ppm, respectively, while the carbonyl carbon C-7 resonated most downfield at *δ*_C_ 164.58 ppm. The quaternary carbons C-20 and C-23, belonging to the 1,2,3-triazole ring, were observed at *δ*_C_ 128.80 ppm and 142.05 ppm, respectively. The HRMS spectrum of compound 11b further confirmed the proposed structure, showing a molecular ion peak at *m*/*z* 430.14.

Moreover, the important carbons of compound 11b were successfully determined by HMBC (Fig. 4). The aromatic proton of benzotriazole H-27 showed HMBC correlations with C-23 (*δ*_C_ 1280.80 ppm), C-26 (*δ*_C_ 125.37 ppm), and C-28 (*δ*_C_ 142.05 ppm). Similarly, the proton H-18 showed correlations with C-5 (*δ*_C_ 105.75 ppm) and C-6 (*δ*_C_ 143.11 ppm). In addition, the proton H-4 showed HMBC correlations with multiple carbons such as C-2 (*δ*_C_ 152.47 ppm), C-5 (*δ*_C_ 105.75 ppm), C-6 (*δ*_C_ 143.11 ppm), C-7 (*δ*_C_ 164.58 ppm), and C-11 (*δ*_C_ 141.11 ppm) (Fig. 4).

### Biological studies

#### Antibacterial evaluation

All the synthesized novel compounds 10a–j and 11a–j were evaluated for antibacterial susceptibility against seven bacterial strains *viz*., three Gram-positive bacteria, *Staphylococcus aureus* (*S. aureus*, ATCC 25923), methicillin-resistant *Staphylococcus aureus* (MRSA, ATCC 33591), *Bacillus subtilis* (*B. subtilis*, ATCC 6051), and four Gram-negative, *Klebsiella pneumoniae* (*K. pneumoniae*, ATCC 700603), *Escherichia coli* (*E. coli*, ATCC 35218), *Pseudomonas aeruginosa* (*P. aeruginosa*, ATCC 27853), and *Salmonella typhi* (*S. typhi*, ATCC 8382) panel using broth microdilution assay. The moxifloxacin was used as the standard drug, and the results are displayed in Table 1 and demonstrated in a graphical presentation (Fig. S1a–d). DMSO was also taken in a control experiment, which showed no effect in the experiment. The antibacterial activities of compounds 10a–j and 11a–j were evaluated against Gram-positive and Gram-negative bacterial strains.

**Table 1:** *In vitro* antibacterial activity of the tested compounds against the Gram-positive and Gram-negative pathogen panel

Code	MIC^a^ (µg mL^−1^)							*C* log *P*^b^
	Gram +ve			Gram −ve				
	*B. subtilis*	*S. aureus*	MRSA	*S. typhi*	*E. coli*	*P. aeruginosa*	*K. pneumoniae*	
10a	50	>100	50	12.5	>100	50	>100	4.13
10b	**3.12**	**6.25**	>100	**3.12**	12.5	>100	>100	4.80
10c	>100	**6.25**	25	**0.39**	12.5	>100	>100	4.80
10d	25	**3.12**	**3.12**	100	**0.19**	50	>100	5.76
10e	>100	**6.25**	>100	**0.39**	**0.39**	25	>100	4.25
10f	>100	12.5	>100	>100	12.5	>100	>100	4.25
10g	>100	**6.25**	>100	>100	>100	>100	>100	4.47
10h	>100	**6.25**	12.5	**0.78**	12.5	>100	>100	4.47
10i	12.5	**3.12**	>100	**0.78**	**0.39**	50	>100	4.60
10j	>100	12.5	>100	>100	**3.12**	>100	>100	5.04
11a	12.5	12.5	>100	12.5	50	>100	>100	3.45
11b	**1.56**	**3.12**	**0.78**	12.5	25	25	>100	3.95
11c	50	>100	**6.25**	100	12.5	50	>100	3.95
11d	>100	12.5	>100	>100	12.5	50	>100	4.87
11e	**1.56**	**6.25**	50	12.5	25	50	>100	3.37
11f	50	>100	>100	>100	>100	50	>100	3.37
11g	50	>100	50	12.5	>100	50	>100	3.59
11h	50	>100	100	100	12.5	50	>100	3.59
11i	**1.56**	**1.56**	>100	12.5	**6.25**	50	>100	3.73
11j	50	>100	25	>100	12.5	50	>100	4.16
Moxifloxacin	**0.19**	**0.19**	**1.56**	**0.39**	**0.19**	**0.19**	**0.19**	**1.68**

a Minimum inhibitory concentration.

b Calculated with ChemDraw Ultra 16.0.

Among the Gram-positive bacteria, series 10 showed strong activity against *S. aureus*, with most compounds exhibiting MIC values of 3.12–12.50 µg mL^−1^. Compounds 10d and 10i were the most potent (MIC = 3.12 µg mL^−1^), while 10d also displayed the broadest spectrum of activity against *B. subtilis* (MIC = 25 µg mL^−1^) and MRSA (MIC = 3.12 µg mL^−1^), emerging as the lead compound of the series. In contrast, most derivatives showed limited activity against *B. subtilis* and MRSA.

Series 11 generally demonstrated superior Gram-positive antibacterial activity. Compounds 11b and 11i were highly active against *S. aureus* (MIC = 3.12 and 1.56 µg mL^−1^, respectively), whereas 11b, 11e, and 11i exhibited the strongest activity against *B. subtilis* (MIC = 1.56 µg mL^−1^). Notably, compound 11b showed exceptional potency against MRSA (MIC = 0.78 µg mL^−1^), surpassing the standard drug by 2-fold, and emerged as the most promising derivative of the series due to its excellent broad-spectrum activity against all Gram-positive strains tested.

Against Gram-negative bacteria, series 10 displayed notable activity against *S. typhi* and *E. coli*. Compounds 10c and 10e matched the standard drug against *S. typhi* (MIC = 0.39 µg mL^−1^), while compounds 10d, 10e, and 10i exhibited excellent activity against *E. coli* (MIC = 0.19–0.39 µg mL^−1^). However, only moderate activity was observed against *P. aeruginosa*, and all compounds were inactive against *K. pneumoniae* (MIC >100 µg mL^−1^). Hence, compounds 10e and 10i were identified as the most promising Gram-negative antibacterial agents in series 10.

In series 11, moderate activity was observed against *S. typhi* (MIC = 12.50 µg mL^−1^ for compounds 11a, 11b, 11e, 11g, and 11i) and *E. coli* (MIC = 6.25–50 µg mL^−1^ for most compounds). The derivatives also exhibited moderate activity against *P. aeruginosa* (MIC = 25–50 µg mL^−1^), whereas all compounds were inactive against *K. pneumoniae* (MIC >100 µg mL^−1^). In conclusion, series 11 demonstrated stronger activity against Gram-positive than Gram-negative bacterial strains.

#### Structure–activity relationship

The structure–activity relationship (SAR) of the synthesized compounds was based on bioisosteric modifications, which are widely employed in drug design to optimize pharmacological activity, improve target selectivity, and reduce undesirable physicochemical properties. In the present study, the antibacterial activity was found to depend on a combination of electronic effects, steric factors, substituent position, lipophilicity (log *P*), and the nature of the triazole bioisostere. These structural parameters collectively influence membrane permeability, molecular conformation, and the ability of the compounds to establish favorable hydrophobic and hydrogen-bonding interactions with putative bacterial targets. The different bioisosteres, electronic effects, steric effects, positional effects of substituents, calculated log *P* values, and MIC data are summarized in Table 1. For clarity, the SAR discussion has been organized according to the compound series, bacterial type, and individual bacterial strains.

In series 10, against the Gram-positive strain *B. subtilis*, the unsubstituted phenyl analogue 10a (log *P* = 4.13) exhibited moderate activity (MIC = 50 µg mL^−1^). Introduction of electron donating (ED) an alkyl substituent (4-CH_3_, 4-*i*Pr) markedly enhanced the potency, as observed for 10b (4-CH_3_; MIC = 3.12 µg mL^−1^, log *P* = 4.80), reflecting a 16-fold improvement, while 10d (4-*i*Pr; MIC = 25 µg mL^−1^) enhanced the antibacterial activity 2-fold. The enhanced activity of 10b is likely attributed to the increased lipophilicity imparted by the methyl group, which may facilitate penetration through the Gram-positive bacterial cell wall while maintaining minimal steric hindrance for productive target interactions. Although the isopropyl substituent is also ED, its larger steric bulk may partially restrict optimal binding, resulting in lower activity than 10b despite its higher hydrophobicity. The methoxy derivative (10e, 4-OMe) showed negligible activity (MIC > 100 µg mL^−1^). Although methoxy is also ED substituent, its stronger electron-releasing resonance effect than the alkyl group, together with increased polarity, which may alter the electronic distribution of the aromatic ring and reduce favorable hydrophobic interactions with the bacterial target. In contrast, electron-withdrawing (EW) groups, such as fluoro (10g, 4-F), resulted in substantial loss of activity (MIC > 100 µg mL^−1^), suggesting that EW substituents are generally unfavorable for antibacterial activity in the 1,2,4-triazole series against *B. subtilis*. Positional effects were also evident; substitution at the *meta*-position with methyl (10c), methoxy (10f), fluoro (10h), and chloro (10j) groups also yielded weakly active analogues (MIC > 100 µg mL^−1^), indicating that *para*-substitution provides a more favorable orientation of the substituent for interaction with the biological target, whereas *meta*-substitution may disrupt optimal molecular recognition. Interestingly, the difluoro analogue 10i (2,4-diF) displayed improved potency (MIC = 12.5 µg mL^−1^) compared with the mono-fluorinated analogues (10g and 10h), suggesting that the combined electronic influence of dual fluorine substitution and the resulting increase in lipophilicity may partially compensate for the unfavorable effect of a single electron-withdrawing substituent.

Replacement of the 1,2,4-triazole scaffold with the fused benzotriazole moiety (series 11) led to a noticeable enhancement in activity against *B. subtilis*. This trend suggests that the benzotriazole scaffold provides a more favorable electronic environment and greater aromatic surface area, which may strengthen π–π stacking or hydrophobic interactions with bacterial targets. Furthermore, the slightly lower log *P* values observed for several benzotriazole derivatives may provide a better balance between aqueous solubility and membrane permeability. Within this series, *para*-substituted alkyl (11b) and alkoxy (11e) derivatives exhibited the highest potency (MIC = 1.56 µg mL^−1^), whereas the bulky isopropyl analogue (11d) was inactive (MIC >100 µg mL^−1^). Unlike the 1,2,4-triazole series, excessive steric bulk appears detrimental in the benzotriazole scaffold, suggesting that the fused triazole system possesses different spatial requirements for target binding. EDG-substituted compounds consistently outperformed EW group analogues at the *para* position. At the *meta* position, both EDG and EWG derivatives exhibited comparable activity (MIC = 50 µg mL^−1^). At the *meta* position, both EDG- and EWG-substituted derivatives showed only moderate activity (MIC = 50 µg mL^−1^), again highlighting the importance of substituent position. Notably, the di-substituted fluoro compound 11i (MIC = 1.56 µg mL^−1^) demonstrated higher potency than the corresponding mono-substituted analogues, indicating that multiple fluorine substitution is beneficial within the benzotriazole framework.

**Fig. 5:**
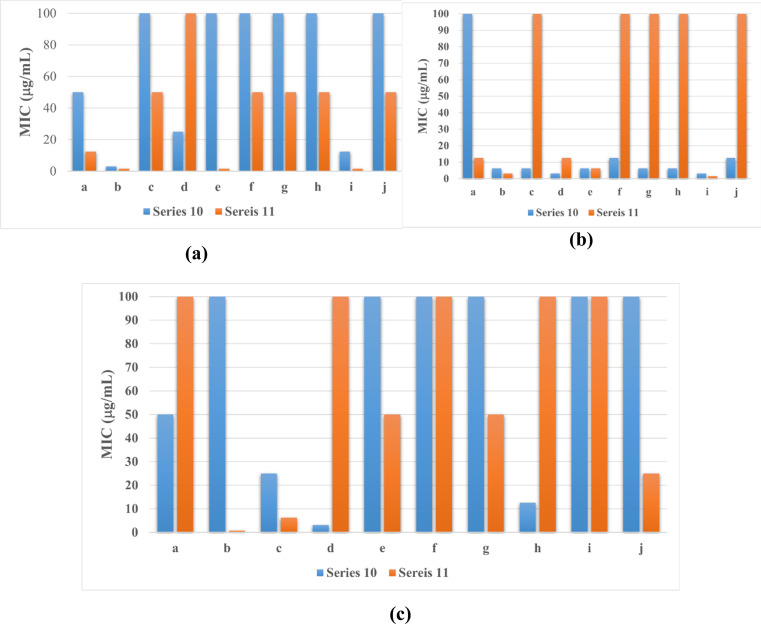
Comparative antibacterial activity of the synthesized series 10 (1,2,4-triazole hybrids) and series 11 (benzotriazole hybrids) against Gram-positive bacterial strains: (a) *B. subtilis*, (b) *S. aureus*, and (c) MRSA. The graphical comparison illustrates the variation in antibacterial potency among the synthesized derivatives, highlighting the influence of the azole scaffold and aromatic substituents on activity. The figure facilitates the visualization of SAR trends and identifies the most active compounds against each bacterial strain.

Against *S. aureus*, the unsubstituted phenyl analogue 10a showed poor activity (MIC > 100 µg mL^−1^) (Fig. 5b). Unlike the trend observed for *B. subtilis*, both ED substituents (4-CH_3_ and 4-OMe) and the EW fluoro substituent (4-F) produced comparable antibacterial activity (MIC = 6.25 µg mL^−1^), suggesting that electronic effects play a less dominant role against this organism. Instead, steric effects appear to exert a stronger influence, as demonstrated by the superior activity of the bulky *para*-isopropyl derivative 10d (MIC = 3.12 µg mL^−1^). This observation suggests that increased hydrophobic surface area may enhance interactions with the bacterial target in *S. aureus*. Among the *meta*-substituted derivatives, 10c showed greater activity than 10f, while 10h was more active than 10j, indicating that subtle differences in both electronic properties and steric size influence antibacterial potency at the *meta* position. The di-substituted fluoro derivative (10i, 2,4-diF) also exhibited improved activity compared to the corresponding mono-substituted compounds.

Scaffold modification to the benzotriazole series generally reduced activity against *S. aureus*. This indicates that the 1,2,4-triazole scaffold is more favorable for activity against this strain, possibly because its less rigid structure and different nitrogen arrangement provide improved hydrogen-bonding interactions with the bacterial target. Nevertheless, compounds 11a, 11b, and 11i retained good antibacterial activity, with 11b and 11i outperforming their corresponding 1,2,4-triazole analogues.

Against MRSA, the unsubstituted compound 10a exhibited moderate activity (MIC = 50 µg mL^−1^) (Fig. 5c). Interestingly, the SAR differed markedly from that observed for the other Gram-positive strains. *Para*-substituted derivatives containing methyl (10b), methoxy (10e), or fluoro (10g) substituents were inactive (MIC >100 µg mL^−1^), whereas the bulky *para*-isopropyl analogue 10d displayed outstanding potency (MIC = 0.78 µg mL^−1^). This finding suggests that steric bulk and increased hydrophobicity are particularly advantageous for overcoming the intrinsic resistance mechanisms of MRSA, possibly by enhancing interactions with altered bacterial proteins or improving membrane penetration. A similar trend was observed for the *meta*-substituted analogues. In contrast, the difluoro derivative 10i exhibited negligible activity, indicating that excessive EW character is unfavorable for anti-MRSA activity within the 1,2,4-triazole scaffold.

Replacement of the 1,2,4-triazole scaffold with benzotriazole resulted in a reversal of the steric preference. The bulky isopropyl derivative 11d completely lost activity (MIC >100 µg mL^−1^), whereas the smaller methyl analogue 11b exhibited excellent potency (MIC = 0.78 µg mL^−1^). These contrasting trends demonstrate that the biological activity of these hybrids depends not only on the substituent itself but also on its interplay with the triazole scaffold. The distinct electronic distribution, molecular planarity, and conformational characteristics of the 1,2,4-triazole and benzotriazole moieties likely alter the optimal steric and electronic requirements for bacterial target recognition. Overall, the results indicate that antibacterial activity is governed by a delicate balance between electronic properties, steric effects, substituent position, and scaffold architecture, with the preferred structural features varying according to the bacterial species.

**Fig. 6:**
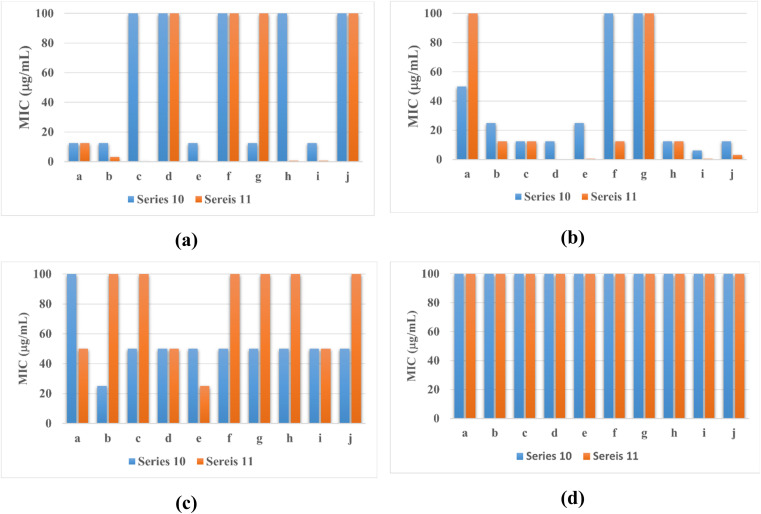
A comparative graphical representation of series 10 and 11 against (a) *S. typhi*, (b) *E. coli*, (c) *P. aeruginosa* and (d) *K. pneumoniae* strain. The figure facilitates the visualization of SAR trends and identifies the most active compounds against each bacterial strain.

For the Gram-negative strain *S. typhi*, the unsubstituted compound (10a) exhibited good activity with an MIC of 12.50 µg mL^−1^ (Fig. 6a). Introduction of ED substituents at the *para*-position significantly enhanced antibacterial activity, indicating that increasing electron density on the aromatic ring favors interactions with the bacterial target. Accordingly, the *para*-methyl (10b) and *para*-methoxy (10e) derivatives displayed MIC values of 3.12 and 0.39 µg mL^−1^, respectively. Among these, the methoxy substituent produced the greatest enhancement, suggesting that its combined ED ability and hydrogen-bond accepting character may promote stronger interactions with bacterial biomolecules. In contrast, the bulky *para*-isopropyl analogue (10d) exhibited reduced potency (MIC = 100 µg mL^−1^). This observation indicates that excessive steric bulk is unfavorable against *S. typhi*, possibly because it hinders penetration through the Gram-negative outer membrane or limits access to the bacterial target. Similarly, incorporation of the EW fluoro substituent (10g, 4-F) substantially reduced antibacterial activity, demonstrating that EW groups are generally less favorable than ED substituents in this scaffold. Positional effects were also evident; the *meta*-methyl derivative (10c) exhibited superior activity (MIC = 0.39 µg mL^−1^) compared with its *para*-analogue (10b), whereas the *meta*-methoxy derivative was less active than 10e. Similarly, the *meta*-fluoro analogue (10h, MIC = 0.78 µg mL^−1^) outperformed the corresponding *para*-fluoro compound (10g). These findings suggest that the optimal substitution position depends on the electronic nature of the substituent, with *meta*-substitution being advantageous for fluorine and methyl groups but less favorable for methoxy substitution. The difluoro analogue (10i) also demonstrated excellent activity (MIC = 0.78 µg mL^−1^), indicating that multiple fluorine substitutions can compensate for the reduced activity associated with single EW substituents, possibly through increased lipophilicity and improved molecular recognition.

Replacement of the 1,2,4-triazole scaffold with the benzotriazole moiety (series 11) generally resulted in lower antibacterial activity than the corresponding series 10 analogues, suggesting that the 1,2,4-triazole scaffold provides a more favorable balance of electronic properties and molecular flexibility for activity against *S. typhi*. Nevertheless, a similar SAR trend was observed, where *para*-substituted ED group enhanced activity, except for the bulky group (11d), which reduced it. Unlike series 10, however, both ED and EW substituted *para*-derivatives displayed comparable potency (MIC = 12.50 µg mL^−1^), indicating that electronic effects are less influential within the benzotriazole scaffold. *Meta*-substitution generally lowered activity, while the di-fluoro derivative (11i) maintained moderate potency (MIC = 12.50 µg mL^−1^).

For *E. coli*, the unsubstituted phenyl analogue (10a) exhibited poor activity (MIC > 100 µg mL^−1^) (Fig. 6b). ED substituents introduced at the *para*-position substantially enhanced antibacterial potency in the order *i*Pr > OMe > Me, demonstrating that increasing hydrophobicity together with electron donation positively influences activity. The bulky isopropyl derivative (10d) displayed the highest activity (MIC = 0.19 µg mL^−1^), followed by the methoxy (10e) and methyl (10b) analogues. These observations suggest that enhanced hydrophobic interactions and increased membrane permeability are particularly beneficial for antibacterial activity against *E. coli*. Conversely, the *para*-fluoro analogue (10g) showed no improvement in activity, indicating that EW substituents are unfavorable at this position. At the *meta* position, the methyl (10c) and methoxy (10f) analogues exhibited comparable antibacterial activity (MIC = 12.50 µg mL^−1^). Among the EW substituents, chlorine proved more beneficial than fluorine, as the *meta*-chloro analogue (10j) displayed greater potency than the corresponding fluoro derivative (10h). This may reflect the greater lipophilicity and larger steric size of chlorine, which can strengthen hydrophobic interactions with bacterial targets. Furthermore, multi-substituted derivatives, such as the di-fluoro (10i) analogue, displayed enhanced potency with an MIC of 0.39 µg mL^−1^, again highlighting the favorable effect of multiple fluorine substitution despite the generally weaker activity of mono-fluorinated derivatives.

Similar to the trend observed against *S. typhi*, replacement of the 1,2,4-triazole ring with the benzotriazole synthon (series 11) led to a general decline in activity (Fig. 6c), suggesting that the electronic distribution and conformational flexibility of the 1,2,4-triazole framework are more favorable for activity against *E. coli*. The unsubstituted compound (11a) exhibited moderate activity (MIC = 50 µg mL^−1^). *Para*-substituted EDGs (11b, 11d, and 11e) increased activity, with the bulky *i*Pr analogue (11d), which was slightly more potent than other derivatives. Unlike the 1,2,4-triazole series, however, the magnitude of improvement was considerably smaller, indicating that the benzotriazole scaffold is less responsive to substituent modification. At the *meta*-position, the methyl substitution remained beneficial, whereas the methoxy analogue (11f) lost activity completely. Both *meta*-fluoro (11h) and *meta*-chloro (11j) exhibited similar potency, while the di-fluoro derivative (11i) retained moderate activity.

For *P. aeruginosa*, generally both series displayed moderate activity with MICs ranging between 25–50 µg mL^−1^ (Fig. 6d). The relatively weak activity observed for both scaffolds is consistent with the intrinsic multidrug resistance of *P. aeruginosa*, which possesses a highly impermeable outer membrane and multiple active efflux systems. Within series 10, the unsubstituted compound (10a) showed moderate activity (MIC = 50 µg mL^−1^). Among the ED substituents, the *para*-methoxy derivative (10e) provided the greatest improvement (MIC = 25 µg mL^−1^), whereas the methyl analogue (10b) showed minimal enhancement. The bulky isopropyl analogue (10d) retained moderate activity, indicating that steric effects are less influential against *P. aeruginosa* than observed for other bacterial strains. The *para*-fluoro analogue (10g) was inactive, suggesting that neither electron-withdrawing substituents nor positional changes effectively overcome the permeability barrier of this organism. The di-fluoro compound displayed moderate potency.

Replacement of the 1,2,4-triazole scaffold with benzotriazole (series 11) produced a similar SAR profile. The *para*-methyl derivative (11b) completely lost activity, whereas the *para*-isopropyl (11d) and *para*-methoxy (11e) analogues retained moderate antibacterial potency. Overall, both ED and EW substituents produced only modest changes in activity, indicating that scaffold modification has a limited influence against *P. aeruginosa*. This observation suggests that poor membrane penetration rather than intrinsic target affinity is likely the major factor limiting antibacterial efficacy. The difluoro analogue (11i) exhibited activity comparable to its corresponding 1,2,4-triazole analogue.

Both compound series exhibited weak antibacterial activity (MIC >100 µg mL^−1^) against *K. pneumoniae*. The uniformly poor activity across both scaffolds, irrespective of substituent type or position, suggests that neither electronic nor steric modification was sufficient to overcome the formidable permeability barrier and multidrug resistance mechanisms characteristic of this organism. Consequently, no clear SAR could be established for *K. pneumoniae*, indicating that additional structural optimization will be required to improve activity against this highly resistant Gram-negative pathogen.

**Fig. 7:**
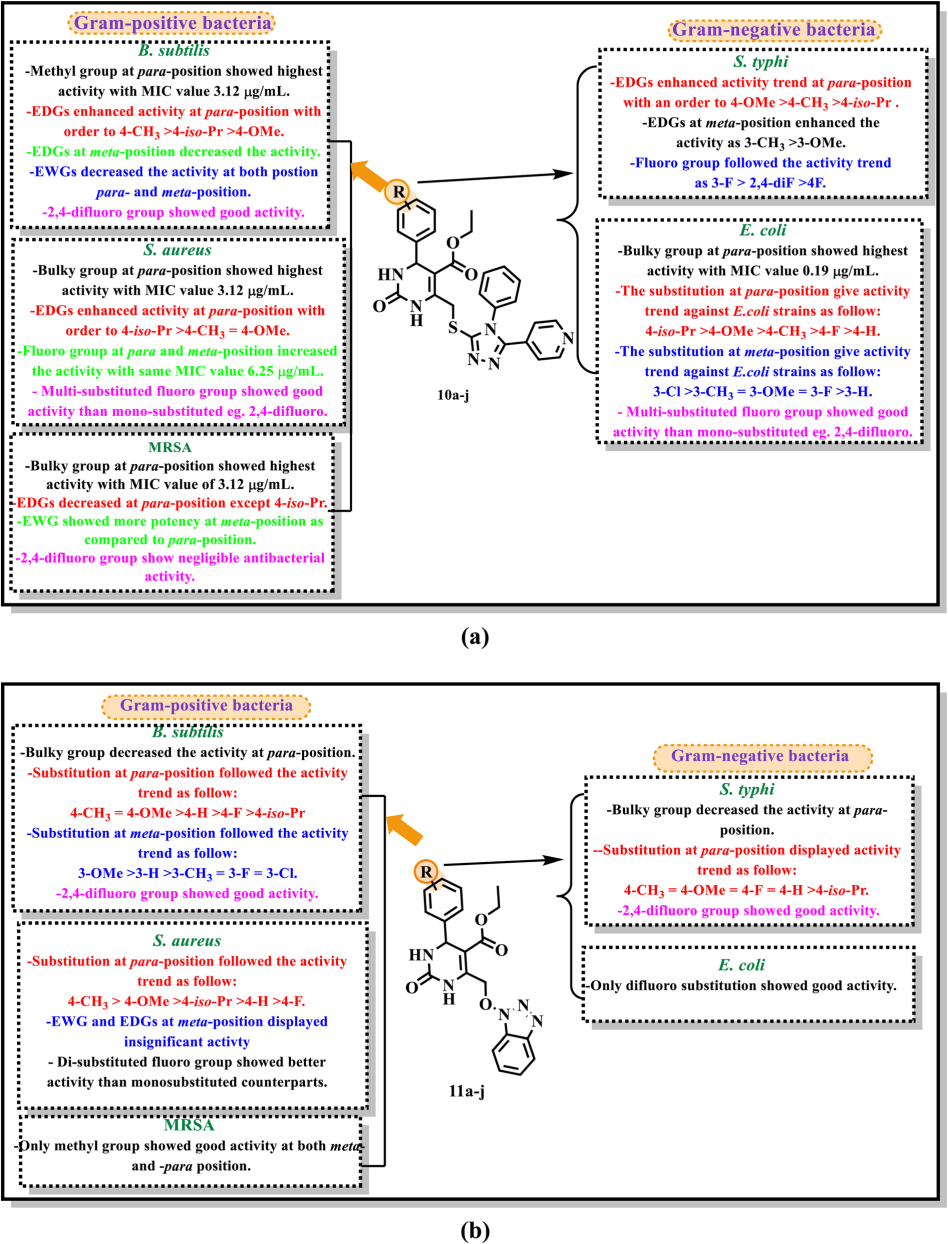
Schematic representation of the SAR analysis of the synthesized DHPM-Azole hybrids: (a) series 10a–10j containing the 1,2,4-triazole moiety and (b) series 11a–11j containing the benzotriazole moiety.

A concise overview of the principal SAR trends identified for series 10a–10j and series 11a–11j is summarized in Fig. 7a and b, respectively, highlighting the influence of electronic properties, steric factors, substituent position, and triazole scaffold on antibacterial activity across the evaluated bacterial strains.

#### Cytotoxicity and selectivity index

The cytotoxicity of the synthesized pyrimidine triazole hybrids (10a–10j and 11a–11j) was evaluated against three normal human cell lines, HaCaT, HEK293T, and FHC, to assess their safety profile and therapeutic potential. The results presented in Table 2 indicate that most of the compounds exhibited low cytotoxicity across all tested cell lines, with cell viability generally ranging from approximately 70% to over 100% at the tested concentration.

**Table 2:** The cytotoxicity study of the synthesized hybrids against three normal cell lines (HaCaT, HEK293T, and FHC)

Compounds	HaCaT	HEK293T	FHC
10a	90.2 ± 12.9	112.9 ± 4.8	99.6 ± 20.4
10b	75.5 ± 9.6	100.9 ± 2.7	109.7 ± 5.4
10c	86.8 ± 7.0	97.2 ± 16.4	96.7 ± 6.6
10d	70.9 ± 8.70	99.8 ± 14.4	94.3 ± 9.0
10e	102.5 ± 16.1	99.0 ± 10.3	98.9 ± 7.3
10f	91.4 ± 9.6	102.9 ± 6.9	94.4 ± 4.6
10g	84.0 ± 4.8	105.1 ± 3.4	97.69 ± 4.3
10h	86.4 ± 5.1	94.4 ± 7.4	69.9 ± 7.0
10i	83.5 ± 16.2	111.1 ± 7.5	93.8 ± 8.4
10j	92.8 ± 12.9	100.1 ± 8.5	100.9 ± 13.8
11a	96.0 ± 8.0	108.0 ± 4.1	90.1 ± 16.6
11b	90.6 ± 12.3	100.2 ± 6.8	90.4 ± 2.9
11c	92.7 ± 7.4	101.6 ± 4.9	91.8 ± 3.7
11d	84.6 ± 2.9	104.3 ± 7.1	87.2 ± 3.4
11e	95.8 ± 12.5	85.8 ± 3.5	92.6 ± 8.9
11f	92.4 ± 15.8	87.9 ± 3.8	88.3 ± 8.7
11g	82.4 ± 12.5	113.4 ± 20.6	89.5 ± 9.6
11h	90.0 ± 8.0	90.5 ± 15.4	90.2 ± 9.9
11i	88.3 ± 5.4	84.6 ± 5.9	95.4 ± 8.7
11j	86.5 ± 16.1	93.1 ± 3.4	90.4 ± 2.5

In series 10, the majority of compounds maintained high viability in all three cell lines, suggesting minimal toxicity toward normal cells. Compounds such as 10a, 10c, 10f, and 10j consistently showed cell viability above 90%, indicating good biocompatibility. A slight reduction in viability was observed for compound 10d in HaCaT cells and for compound 10h in FHC cells, where values were around 70%. However, these effects were moderate and did not indicate significant cytotoxicity. Overall, the cytotoxicity profile of series 10 suggests that these compounds are well tolerated in normal cell systems.

A similar trend was observed for series 11, where most compounds displayed cell viability values between 85% and 110% across the three cell lines. Compounds 11a, 11b, and 11c exhibited consistently high viability, while compounds such as 11e and 11f showed slightly reduced viability in HEK293T cells. Compound 11d also showed a modest decrease in HaCaT cells. Despite these minor variations, none of the compounds in this series demonstrated marked toxicity, indicating a favorable safety profile comparable to that of series 10.

To further evaluate the therapeutic relevance of these compounds, the selectivity index (SI) was calculated by comparing cytotoxicity toward normal cells with antibacterial activity. The SI values presented in Table 3 and 4 highlight clear differences in selectivity among the compounds.

**Table 3:** The selectivity index for Gram-positive bacterial strains

Code	*B. subtilis*			*S. aureus*			MRSA		
	HaCaT	HEK293T	FHC	HaCaT	HEK293T	FHC	HaCaT	HEK293T	FHC
10a	1.8	2.2	1.9	1.0	1.1	1.0	1.8	2.2	1.9
10b	24.1	32.3	35.1	12.0	16.1	17.5	0.7	1.0	1.0
10c	1.0	1.0	1.0	13.8	15.5	15.4	3.4	3.8	3.8
10d	2.8	3.9	3.7	22.7	31.9	30.2	22.5	31.6	29.9
10e	1.0	1.0	1.0	16.4	15.8	15.8	1.0	1.0	1.0
10f	1.0	1.0	1.0	7.3	8.2	7.5	1.0	1.0	1.0
10g	1.0	1.0	1.0	13.4	16.8	15.6	1.0	1.0	1.0
10h	1.0	1.0	0.6	13.8	15.1	11.1	6.9	7.5	5.5
10i	6.6	8.8	7.5	26.7	35.6	30.0	1.0	1.0	1.0
10j	1.0	1.0	1.0	7.4	8.0	8.0	1.0	1.0	1.0
11a	7.6	8.6	7.2	7.6	8.6	7.2	1.0	1.0	1.0
11b	58.0	64.2	57.9	29.0	32.1	28.9	116.1	128.4	115.8
11c	1.8	2.0	1.8	1.0	1.0	0.9	14.8	16.2	14.6
11d	1.0	1.0	1.0	6.7	8.3	6.9	1.0	1.0	1.0
11e	61	55.0	59.3	15.3	13.7	14.8	1.9	1.7	1.8
11f	1.8	1.7	1.7	1.0	1.0	1.0	1.0	1.0	1.0
11g	1.6	2.2	1.7	1.0	1.0	1.0	1.6	2.2	1.7
11h	1.8	1.8	1.8	1.0	1.0	1.0	1.0	1.0	1.0
11i	56.6	54.2	61.1	56.6	54.2	61.1	1.0	1.0	1.0
11j	1.7	1.8	1.8	1.0	1.0	1.0	3.4	3.7	3.6

**Table 4:** The selectivity index for Gram-negative bacterial strains

Code	*S. typhi*			*E. coli*			*P. aeruginosa*			*K. pneumoniae*		
	HaCaT	HEK293T	FHC	HaCaT	HEK293T	FHC	HaCaT	HEK293T	FHC	HaCaT	HEK293T	FHC
10a	7.2	9.0	7.9	0.9	1.1	1.0	1.8	2.2	1.9	1.0	1.1	1.0
10b	24.1	32.3	35.1	6.0	8.0	8.7	0.7	1.0	1.0	0.7	1.0	1.0
10c	222.5	249.2	247.9	6.9	7.7	7.7	1.0	1.0	1.0	1.0	1.0	1.0
10d	0.7	1.0	1.0	373.1	525.2	496.3	1.4	1.9	1.8	0.7	1.0	1.0
10e	262.8	253.8	253.5	262.8	253.8	253.5	4.1	3.9	3.9	1.0	1.0	1.0
10f	1.0	1.0	1.0	7.3	8.2	7.5	1.0	1.0	1.0	1.0	1.0	1.0
10g	1.0	1.0	1.0	1.0	1.0	1.0	1.0	1.0	1.0	1.0	1.0	1.0
10h	110.7	121.0	89.6	6.9	7.5	5.5	1.0	1.0	0.6	1.0	1.0	0.6
10i	107.0	142.4	120.2	214.1	284.8	240.5	1.6	2.2	1.8	1.0	1.1	1.0
10j	1.0	1.0	1.0	29.7	32.0	32.3	0.9	1.0	1.0	1.0	1.0	1.0
11a	7.6	8.6	7.2	1.9	2.1	1.8	0.9	1.0	0.9	1.0	1.0	1.0
11b	7.2	8.0	7.2	3.6	4.0	3.6	3.6	4.0	3.6	1.0	1.0	1.0
11c	1.0	1.0	1.0	7.4	8.1	7.3	1.8	2.0	1.8	1.0	1.0	1.0
11d	1.0	1.0	1.0	6.7	8.3	6.9	1.6	2.0	1.7	1.0	1.0	1.0
11e	7.6	6.8	7.4	3.8	3.4	3.7	1.9	1.7	1.8	1.0	0.8	1.0
11f	1.0	1.0	1.0	1.0	1.0	1.0	1.8	1.7	1.7	1.0	0.8	1.0
11g	6.5	9.0	7.1	0.8	1.1	0.8	1.6	2.2	1.7	1.0	1.1	1.0
11h	1.0	1.0	1.0	7.2	7.2	7.2	1.8	1.8	1.8	1.0	0.9	1.0
11i	7.0	6.7	7.6	14.1	13.5	15.2	1.7	1.6	1.9	1.0	0.8	1.0
11j	1.0	1.0	1.0	6.9	7.4	7.2	1.7	1.8	1.8	1.0	0.9	1.0

Among the series 11 derivatives, compound 11b emerged as the most promising candidate, showing exceptionally high SI values against MRSA, with values exceeding 100 across all three cell lines. This indicates strong antibacterial activity combined with minimal toxicity toward normal cells. In addition, compounds 11e and 11i demonstrated good selectivity against *Bacillus subtilis*, with SI values greater than 50, and moderate selectivity against *Staphylococcus aureus*. However, their selectivity against MRSA was comparatively lower, suggesting that activity against susceptible strains does not always translate to resistant pathogens.

In series 10, compound 10b exhibited good selectivity toward Gram-positive bacteria, particularly *B. subtilis* and *S. aureus*, with SI values above 15, although its selectivity against MRSA remained limited. Compounds 10d and 10i showed improved selectivity against *S. aureus*, and notably, compound 10d also demonstrated meaningful selectivity against MRSA, supporting its potential as a lead scaffold for further optimization.

For Gram-negative bacteria, compounds 10d, 10e, and 10i displayed remarkably high SI values, especially against *E. coli*, where values exceeded 200 in several cases. These findings indicate that these compounds combine strong antibacterial potency with low cytotoxicity. Additionally, compounds such as 10c, 10h, and 10i showed good selectivity against *S. typhi*, further supporting their potential as leads for Gram-negative infections. In contrast, most compounds from series 11 exhibited relatively low SI values against Gram-negative strains, indicating limited selectivity.

Overall, the integration of antibacterial activity and selectivity index data reveals that the synthesized hybrids possess a favorable safety profile, with several compounds demonstrating a clear therapeutic window. In particular, compound 11b for Gram-positive bacteria and compounds 10d, 10e, and 10i for Gram-negative bacteria stand out as promising candidates for further development. These findings support the potential of pyrimidine triazole hybrids as safe and effective antibacterial agents while providing a basis for future structural optimization.

The selectivity index (SI) was calculated by dividing the IC_50_ of normal cells by the MIC of bacterial strains, and data presented in Table 3 demonstrate differences between compounds and bacterial strains. Compound 11b significantly displayed SI values against MRSA (116.1, 128.4, and 115.8 for HaCaT, HEK293T, and FHC, respectively), indicating potent antibacterial activity combined with low cytotoxicity. The finding suggested 11b as the most promising MRSA lead within the series. In contrast, compounds 11e and 11i showed excellent selectivity towards *B. subtilis* with SI value >50 and, against *S. aureus*, 11i displayed SI > 50 and 11e showed >25, but both displayed moderate selectivity against MRSA (SI = 1.0–2.2). These results highlight that activity against susceptible Gram-positive species does not necessarily correlate with efficacy against resistant strains.

Among the series 10 derivatives, compound 10b demonstrated good selectivity (SI > 15) against *B. subtilis* and *S. aureus* but showed poor to moderate selectivity toward MRSA (SI = 0.7–1.0). Conversely, 10d and 10i maintained favorable SI values for both *S. aureus*, but against MRSA, only 10d showed good selectivity (up to 31.9), suggesting their potential as scaffolds for *S. aureus*.

Further, SI was calculated against Gram-negative strains and the results are summarized in Table 4, which demonstrates significant variability in the activity of series 10 and 11 compounds. In series 10, compounds 10d, 10e, and 10i exhibited remarkably high SI values, particularly against *E. coli* (10d = 373.1–525.2, 10e = 253.5–262.8, 10i = 214.1–284.8), indicating potent antibacterial efficacy combined with low cytotoxicity. Compounds 10c, 10h, and 10i also showed notable selectivity against *S. typhi* (SI > 100), highlighting their potential as promising leads for Gram-negative infections. However, most series 10 compounds displayed poor selectivity (SI < 4) against *P. aeruginosa* and *K. pneumoniae*.

In contrast, series 11 compounds generally exhibited lower SI values across all Gram-negative strains, indicating limited selectivity. Exceptions include 11i, which showed moderate selectivity toward *E. coli* (SI = 13–15), and 11g and 11h, which demonstrated slightly improved activity against *S. typhi* (SI up to 9). Other derivatives of the series, such as 11b, 11d, and 11f, remained below SI values of 4.

### ADME/T prediction and correlation with antibacterial activity

The predicted ADME/T properties of the synthesized compounds 10a–10j and 11a–11j were evaluated using SwissADME and pkCSM to assess their drug-like characteristics and to establish a possible relationship between physicochemical properties and antibacterial performance (Table S1 & S2). The calculated molecular weights of compounds 10a–10j ranged from 512 to 554 Da, whereas compounds 11a–11j exhibited lower molecular weights (393–435 Da). Similarly, the series-11 derivatives showed reduced topological polar surface area (*t*PSA, 107–116 Å^2^) compared with the series-10 compounds (136–145 Å^2^), suggesting potentially improved membrane permeability and drug-like properties. The hydrogen-bonding characteristics of all compounds remained within acceptable ranges, with 6–8 hydrogen bond acceptors and 2 hydrogen bond donors, indicating that the compounds retain sufficient capacity for molecular interactions with biological targets.

The calculated lipophilicity values (*C* log *P* = 3.37–5.76) indicated that all compounds possess moderate to high hydrophobicity, which may contribute to enhanced interaction with bacterial membranes and improved cellular penetration (Table 1). However, antibacterial activity did not correlate solely with lipophilicity, suggesting that an optimal balance between hydrophobicity, molecular size, and polarity is required for effective antibacterial action. For example, compound 10d exhibited the highest *C* log *P* value (5.76) and showed excellent antibacterial activity against *E. coli* (MIC = 0.19 µg mL^−1^), *S. aureus* (MIC = 3.12 µg mL^−1^), and MRSA (MIC = 3.12 µg mL^−1^). In contrast, compounds with comparable or higher lipophilicity did not always demonstrate similar potency, indicating that other structural factors contribute to antibacterial activity.

The predicted absorption parameters revealed favorable intestinal absorption profiles for most compounds. The series-10 derivatives showed 100% predicted intestinal absorption, whereas the series-11 compounds exhibited good absorption potential (84.79–89.59%). Although the series-10 compounds demonstrated higher predicted absorption, their larger molecular size and higher polar surface area may negatively influence permeability. In comparison, the lower molecular weight and reduced polarity of the series-11 compounds suggest a more balanced physicochemical profile that may facilitate bacterial penetration. The predicted water solubility values (−3.9 to −4.7 log mol L^−1^) indicated limited aqueous solubility, which is frequently observed for hydrophobic antibacterial scaffolds and may require further optimization.

The distribution and toxicity predictions further supported the drug-like potential of the synthesized derivatives. All compounds showed negative predicted BBB permeability values, suggesting limited central nervous system exposure and reduced risk of CNS-related toxicity. Furthermore, none of the compounds showed predicted AMES toxicity, indicating a favorable preliminary safety profile. Metabolic prediction analysis revealed that all compounds were non-substrates of CYP2D6, while differences were observed in CYP3A4, CYP2C9, and CYP2C19 interactions. The series-11 compounds generally showed fewer predicted CYP interactions compared with the series-10 derivatives, suggesting a potentially improved metabolic profile.

Integration of the ADME predictions with antibacterial results indicated that compounds possessing a balanced physicochemical profile and strong antibacterial activity represent the most promising candidates. Among the tested compounds, compound 11b displayed the most favorable overall profile, exhibiting potent activity against *B. subtilis* (MIC = 1.56 µg mL^−1^), *S. aureus* (MIC = 3.12 µg mL^−1^), and MRSA (MIC = 0.78 µg mL^−1^), together with moderate lipophilicity (*C* log *P* = 3.95), lower molecular weight (407 Da), acceptable intestinal absorption, reduced predicted metabolic liability, and no AMES toxicity. Compound 11i also demonstrated promising antibacterial activity against *B. subtilis* and *S. aureus* (MIC = 1.56 µg mL^−1^ each) with a favorable ADME profile. Although compound 10d showed remarkable antibacterial potency, particularly against *E. coli*, its higher molecular weight, increased lipophilicity, and greater CYP interaction potential suggest that further structural optimization may be required.

Overall, the SwissADME analysis supports the biological findings by demonstrating that antibacterial potency is associated with a balance between physicochemical properties and ADMET characteristics rather than a single molecular descriptor. Compounds 11b and 11i, in particular, represent promising lead candidates due to their combination of potent antibacterial activity, favorable drug-like properties, and predicted safety profiles, while compound 10d provides a valuable scaffold for further optimization.

### Possible mechanism of antibacterial action

Although the precise antibacterial mechanism of the synthesized pyrimidine-based *N*-heterocyclic hybrids was not experimentally investigated in the present study, previous studies provide insight into plausible molecular targets. Pyrimidine and 1,2,4-triazole scaffolds are well-established pharmacophores in medicinal chemistry and have been widely reported to exhibit antibacterial activity through inhibition of bacterial DNA gyrase and topoisomerase IV, two essential type II topoisomerases involved in DNA replication, transcription, and chromosome segregation. In several studies, triazolopyrimidine derivatives displayed potent antibacterial activity together with significant DNA gyrase inhibitory activity, and molecular docking confirmed binding interactions comparable to those of ciprofloxacin.^57^ More recently, triazolopyrimidine analogues have also been reported as dual inhibitors of bacterial DNA gyrase and dihydrofolate reductase (DHFR), further supporting these enzymes as attractive targets for nitrogen-rich heterocyclic antibacterial agents.

The synthesized compounds in the present study combine three pharmacologically important heterocyclic motifs, namely pyrimidine, 1,2,4-triazole, and benzotriazole, which may capable of forming hydrogen-bonding and π–π interactions within enzyme active sites. The observed antibacterial activity against both Gram-positive and Gram-negative bacteria, including MRSA, suggests that these hybrid molecules may interfere with conserved bacterial enzymes rather than acting through species-specific mechanisms. In particular, the potent activity of compounds 10d, 11b, and 11i against *S. aureus*, MRSA, *B. subtilis*, and *E. coli* is consistent with the antibacterial profiles reported for DNA gyrase inhibitors containing triazole- and pyrimidine-based pharmacophores. However, because no target-based biological assays or molecular docking studies were performed, this proposed mechanism remains hypothetical.

Furthermore, the SwissADME analysis showed that the most active compounds possess balanced physicochemical and pharmacokinetic properties, including moderate lipophilicity, acceptable predicted intestinal absorption, and the absence of predicted mutagenicity. Such properties are compatible with efficient bacterial cell penetration and intracellular access to enzyme targets. Among the synthesized derivatives, compounds 11b and 11i combine potent antibacterial activity with favorable predicted drug-like properties, whereas compound 10d demonstrates exceptional antibacterial potency despite its comparatively higher molecular weight and lipophilicity. These findings indicate that antibacterial activity is likely governed by a combination of target affinity and favorable physicochemical properties rather than by any single molecular descriptor. Future studies involving molecular docking, enzyme inhibition assays, and target validation will be necessary to confirm their precise mechanism of antibacterial action.

## Conclusion

In this study, a novel series of pyrimidine-based *N*-heterocyclic hybrid compounds incorporating 1,2,4-triazole and benzotriazole moieties was successfully synthesized and fully characterized using spectroscopic techniques. The biological evaluation revealed that several compounds exhibited promising antimicrobial activity against both Gram-positive and Gram-negative bacterial strains. Compound 10d demonstrated outstanding anti-MRSA activity together with acceptable cytotoxicity and favorable drug-like characteristics, while 11b and 11i combined potent Gram-positive antibacterial activity with low cytotoxicity and suitable predicted ADME properties. Other compounds, such as 10e and 10i, also showed promising antibacterial activity; however, differences in activity spectrum and overall pharmacokinetic profile made 10d, 11b, and 11i the most balanced lead candidates for further optimization. Nevertheless, the present study is limited by the absence of detailed mechanistic investigations and advanced biological assays such as time-kill kinetics, antibiofilm activity, minimum bactericidal concentration (MBC), and resistance development studies. Future work will focus on elucidating the molecular targets of these hybrids through enzyme inhibition assays, molecular docking, and target validation studies, together with pharmacokinetic evaluation and lead optimization to support the development of novel antibacterial agents.

## Experimental section

### Materials and methods

#### Chemicals and reagents

All the chemicals used for the synthesis of molecular hybrids and their precursors were purchased from Merck, CRD, Sigma-Aldrich and were used without further purification. TLC 60 F254 silica gel plates were used to monitor the progress of the reaction. ^1^H and ^13^C NMR and 2D NMR spectroscopy were applied to characterize the compounds, which were recorded using a Bruker Avance-III spectrometer (600 MHz). The Fourier transform infrared (FTIR) spectra were obtained using the ATR technique over the range of 400–4000 cm^−1^ on a PerkinElmer FTIR Spectrum II instrument. Chemical shifts are reported in parts per million (ppm), using deuterated dimethyl sulfoxide (DMSO-*d*_6_) as the solvent (*δ*_H_ 2.50 ppm and *δ*_C_ 39.50 ppm), with tetramethylsilane (TMS, *δ*_H_ = 0) as the internal reference. Signal multiplicities are described as singlet (s), doublet (d), triplet (t), quartet (q), multiplet (m), doublet of triplets (dt), doublet of doublets (dd), and coupling constants (*J*) are expressed in Hertz (Hz). High-resolution mass spectra were recorded using a Waters Micromass LCT Premier TOF-MS spectrometer.

#### General procedures for the preparation of intermediate 4

The compound 4 was synthesized as per the reported method.^54^ A mixture of phenyl isothiocynate 1 (1.0 eq.) and isoniazid 2 (1.0 eq.) was taken in a round-bottom flask containing EtOH (10 ml), refluxed for 5–10 minutes. The precipitate was formed 3, filter-out it along with washing with a small amount of ethanol when the reaction reached room temperature. Then this precipitate 3 was treated with 4 N NaOH solution (20 ml) and refluxed for 2–5 hours. After the completion of the reaction, put it at room temperature and neutralized with glacial acetic acid in the pH range of 5 to 6. The crystals were formed after some time and filtered. The filtrate was recrystallized using ethanol to get pure compound 4.

#### General procedure for the preparation of intermediate 6

The intermediate 6 has been prepared by using the reported document.^55^ 2-nitrochlorobenzene 5 (1.0 eq.) treated with hydrazine hydrate (5.0 eq.) in the presence of 1-heptanol at 110–120 °C to form intermediate 6.

#### General procedures for the synthesis of compounds 9a–9j

A series of compounds 9a–9j was synthesised according to the literature.^56^ Aldehyde 8a–8j (1.0 eq.), 4-chloroethylacetoacetate (1.0 eq.), and urea (2.0 eq.) were stirred for two hours at 100 °C under reflux in a round-bottom flask. Conc. HCl was added to the mixture to catalyze the reaction. The resulting solution was allowed to reach room temperature once the reaction was finished, and then it was quenched with water. The solid was filtered out and then recrystallized in ethanol to purify the product 9a–9j.

#### General procedures for the synthesis of compounds 10a–10j

A solution of 8a–8j (1.0 eq.) and intermediate 4 (1.0 eq.) and CH_3_CN (10 ml) was stirred at 80 °C. The activated K_2_CO_3_ (2.5 eq.) was added to the reaction mixture and refluxed for 1.5 hours. TLC was used to monitor the progress of the reaction. The reaction mixture was cooled at room temperature and quenched with water after finishing the reaction. The precipitates were formed, filtered out using a vacuum and purified by using column chromatography (30% hexane/EtOAc). The final compounds 10a–10j were recrystallized using ethanol.

#### General procedures for the synthesis of compounds 11a–11j

First, the K_2_CO_3_ was activated in a microwave for half an hour, then 2.5 equivalent was added into the solution of 9a–9j (1.0 eq.) and intermediate 6 (1.0 eq.) and CH_3_CN (10 ml), refluxed for 30 minutes. The progress of the reaction was monitored by TLC using 20% hexane/EtOAc solution. The reaction mixture was cooled at room temperature and quenched with water after finishing the reaction. The precipitates were formed, filtered out using a vacuum and purified by recrystallization using ethanol.

#### Biological studies

##### Antibacterial assay

The antibacterial screening of all the final compounds was performed against *B.subtilis* (ATCC 6051), *S. Typhi*, MRSA (ATCC 33591), *S. aureus* (ATCC 25923), *E. Coli* (ATCC 35218), *K. Pneumoniae* (ATCC 700603), and *P. aeruginosa* (ATCC 27853) using the micro-broth dilution method as previously published.^58,59^ All strains were cultured and subcultured one day prior to testing at the Department of Microbiology, Inkosi Albert Luthuli Hospital, Durban, South Africa. Moxifloxacin was employed as a positive control, while DMSO was the negative control. All the experiments were carried out in triplicate. The lowest concentration inhibiting bacterial growth was considered the MIC value.

##### Cell culture

Human transformed non-cancerous cell lines HaCaT (immortalized human keratinocytes), HEK293T (human embryonic kidney cells), and FHC (normal human fetal colon epithelial cells) were obtained from the American Type Culture Collection (ATCC, Manassas, VA, USA). HaCaT, and HEK293T cells were maintained in high-glucose Dulbecco's Modified Eagle Medium (DMEM; Thermo Fisher Scientific, USA) supplemented with 10% fetal bovine serum (HyClone®, Logan, UT, USA), 4 mmol L^−1^l-glutamine, and 1% penicillin-streptomycin solution (MP Biomedicals, Solon, OH, USA). FHC cells were maintained in the growth factors supplemented media as recommended by ATCC. Cultures were incubated at 37 °C in a humidified atmosphere without CO_2_. Stock solutions of the test compounds were prepared in dimethyl sulfoxide (DMSO) and diluted to the desired concentrations prior to treatment.

##### Cell viability assay

For the viability assay, HaCaT, HEK293T, and FHC cells were seeded at a density of 5 × 10^3^ cells per well in 96-well plates. After 24 h, cells were treated with varying concentrations of the respective compounds for 48 h in triplicate. Following treatment, the medium was replaced with fresh medium containing 20 µL of MTS reagent per well ([3-(4,5-dimethylthiazol-2-yl)-5-(3-carboxymethoxyphenyl)-2-(4-sulfophenyl)-2*H*-tetrazolium]; Promega, WI, USA). Absorbance was measured at 490 nm (ref. 630 nm) using a microplate reader (MRX Revelation; Dynex Technologies, Chantilly, VA, USA). Cell viability was expressed as a percentage of control, and IC_50_ values were determined by nonlinear regression analysis.

##### ADME predictions

ADME predictions were studied for molecular hybrids using online web portal SwissADME (https://www.swissadme.ch/index.php) and Molinspiration (https://www.molinspiration.com) to evaluate the physicochemical properties, pharmacokinetic parameters, and drug-likeness of the synthesized compounds.^60,61^

## Conflicts of interest

The authors declare no competing interests.

## Supplementary Material

RA-OLF-D6RA05427G-s001

## Data Availability

The authors declare that all the data generated in this work has either been provided in the manuscript or the supplementary information (SI) data file. Also, all the information used from the literature has been appropriately cited. Supplementary information is available. See DOI: https://doi.org/10.1039/d6ra05427g.
