# Supplementary material for: Exploring DHPM-azole hybrid molecules for antibacterial drug discovery: synthesis and biological assessment
Source: RSC Adv. 2026 Aug 5;16(39):44701–18. doi: 10.1039/d6ra05427g (PMC13438781; doi:10.1039/d6ra05427g)
Supplement: Supplementary file 1 [file RA-016-D6RA05427G-s001.pdf]

Supplementary Information (SI) for RSC Advances.
This journal is © The Royal Society of Chemistry 2026

Exploring DHPM-Azole Hybrid Molecules for Antibacterial Drug Discovery: Synthesis and Biological Assessment

Gobind Kumar[a], Safiyah Ghumran[a], Neha Manhas[a], Pule Seboletswe[a], Afsana Kajee[b], Asif Raza[c], Arun K. Sharma[c*], Parvesh Singh[a*]

[a]Discipline of Chemistry, University of KwaZulu-Natal, P/Bag X54001, Westville, Durban 4000, South Africa
[b]Department of Microbiology, National Health Laboratory Services (NHLS), Inkosi Albert Luthuli Central Hospital, Durban, South Africa
[c]Department of Pharmacology; Penn State Cancer Institute, CH72; Penn State College of Medicine, 500 University Drive, Hershey, PA 17033, USA.

*Correspondence: asharma1@pennstatehealth.psu.edu, singhp4@ukzn.ac.za

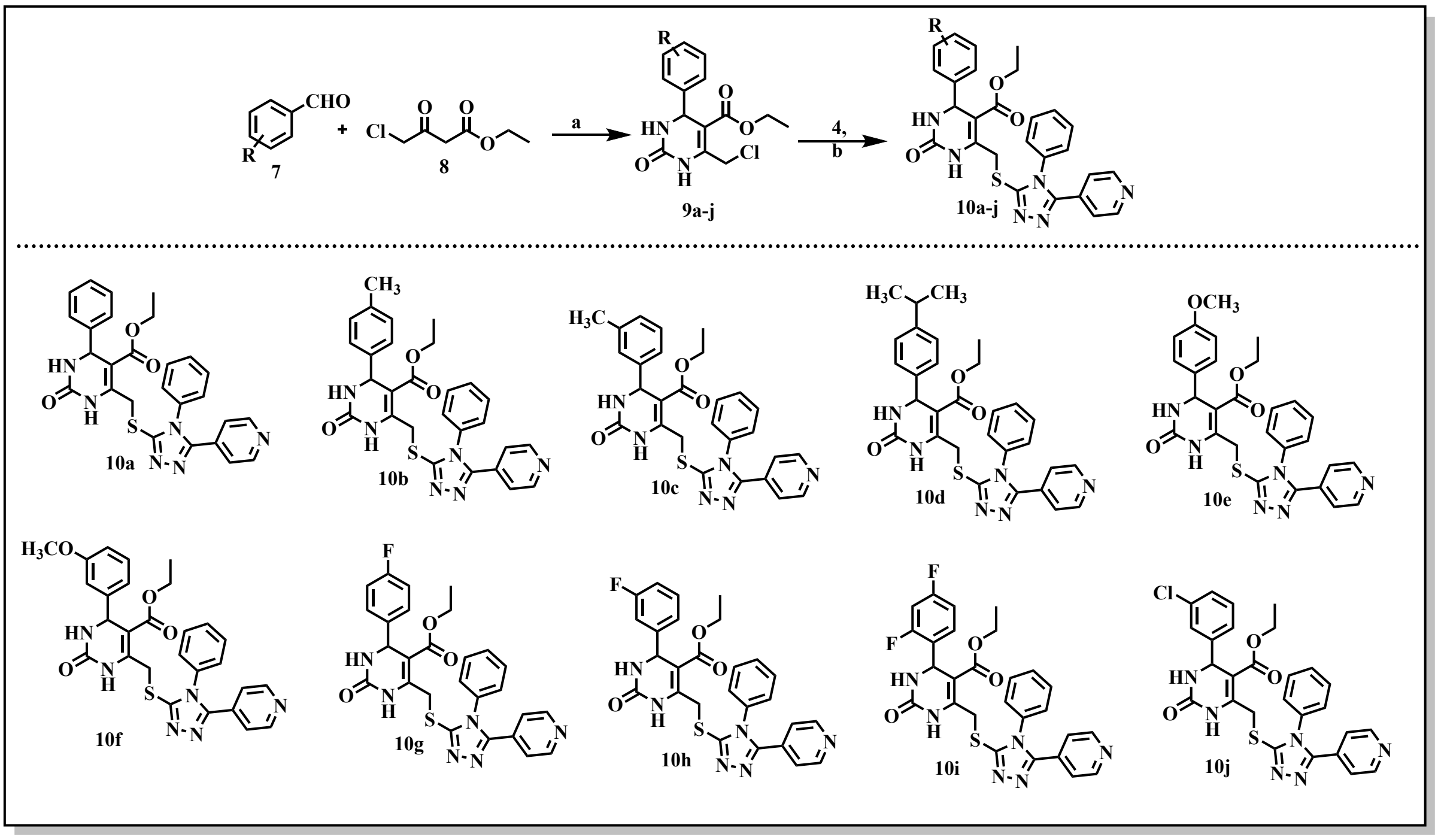

Scheme S1. Illustration of the synthesis of molecular hybrids **10a-j**. Conditions: (a) Urea (2 eq.) and conc. HCl 2-3 drops, heated at 100 ºC for 2 hours; (b) anhydrous K_2CO_3 (2.5 eq.), CH_3CN, refluxed at 80 ºC for 30 minutes.

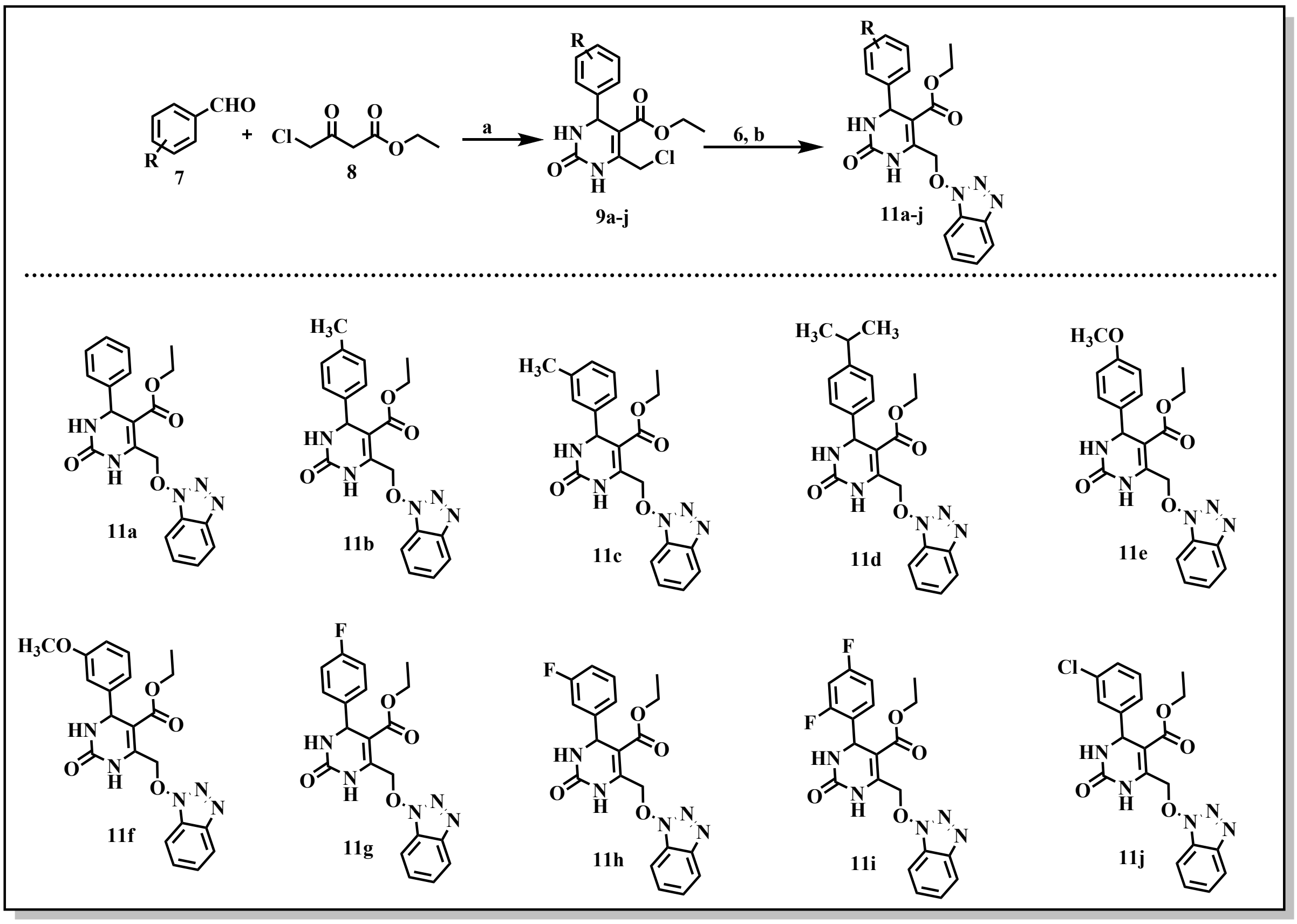

Scheme S2. Illustration of the synthesis of molecular hybrids **11a-j**. Conditions: (a) Urea (2 eq.) and conc. HCl 2-3 drops, heated at 100 ºC for 2 hours; (b) anhydrous K_2CO_3 (2.5 eq.), CH_3CN, refluxed at 80 ºC for 30 minutes.

Antibacterial analysis

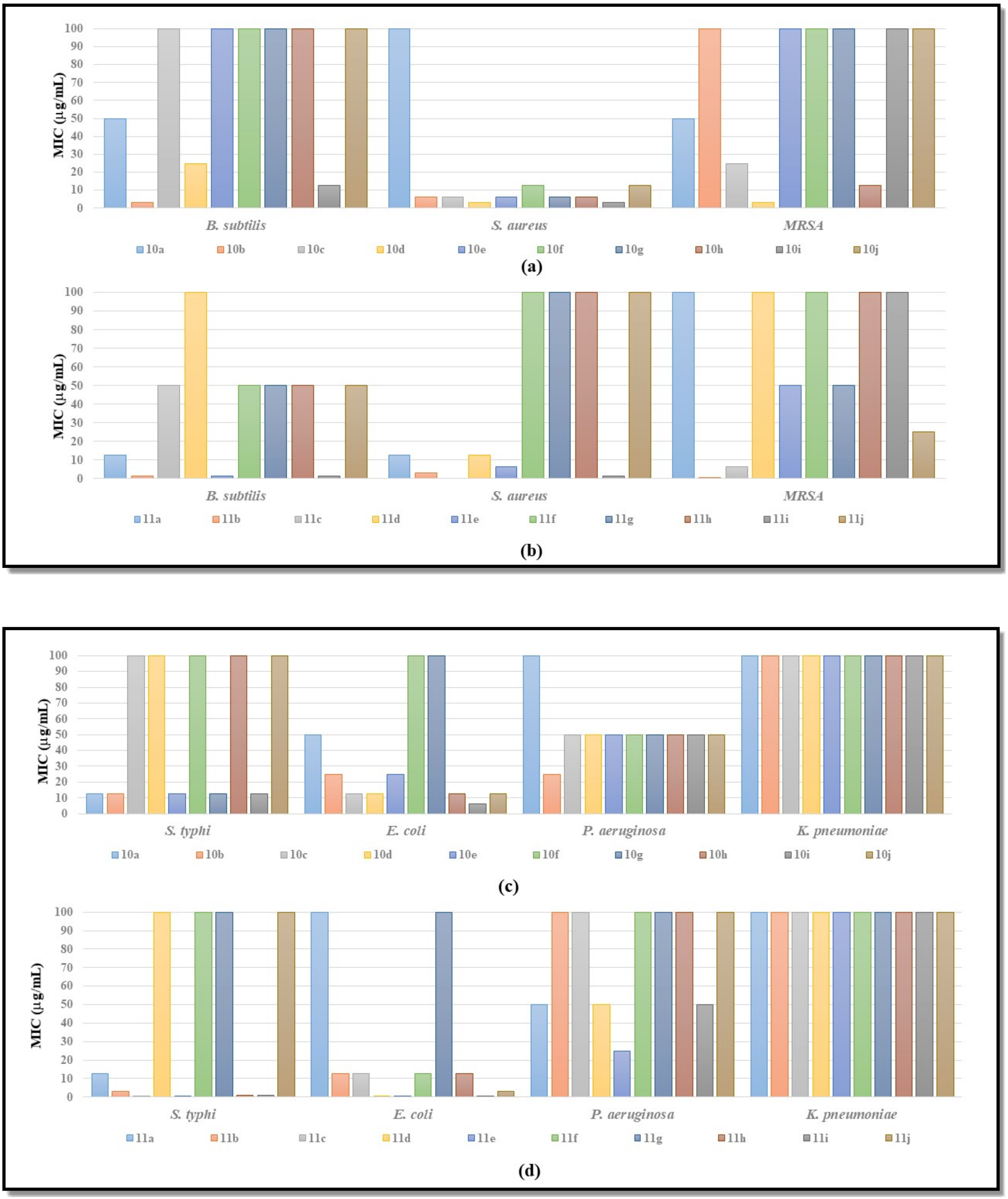

Figure S1. The graphical presentation of antibacterial activity of both series against (a and b) gram-positive and (c and d) gram-negative strains.

Physicochemical properties and ADMET prediction

Table S1. The physicochemical properties of compounds **10a-10j** and **11a-11j**.

Compound	Physicochemical properties[a]				
	M.W	HBA	HBD	^{rot}BOND	^{t}PSA (Å^2)
**10a**	512	6	2	9	136
**10b**	536	6	2	9	136
**10c**	536	6	2	9	136
**10d**	554	6	2	10	136
**10e**	542	7	2	10	145
**10f**	542	7	2	10	145
**10g**	530	7	2	9	136
**10h**	530	7	2	9	136
**10i**	548	8	2	9	136
**10j**	547	6	2	9	136
**11a**	393	6	2	7	107
**11b**	407	6	2	7	107
**11c**	407	6	2	7	107
**11d**	435	6	2	8	107
**11e**	423	7	2	8	116
**11f**	423	7	2	8	116
**11g**	411	7	2	7	107
**11h**	411	7	2	7	107
**11i**	429	8	2	7	107
**11j**	427	6	2	7	107
**Standard range**	<500 [49, 50]	0-20 [50]	0-6 [50]	0-11 [51]	0-140 [50]

[a]M.F= Molecular formula; M.W = molecular weight; HBD = no. of hydrogen bond donor; HBA = no. of

hydrogen bond acceptor; rotBOND = no. of rotatable bond; tPSA = total polar surface area. [b]Reference.

Table S2. ADMET prediction using pkCSM for compounds **10a-j** and **11a-j** along with the standard drug moxifloxacin.

Name	Absorption		Distribution	Metabolism							Excretion	Toxicity
	Water solubility	Intestinal absorption	BBB	CyP							Total clearance	AMES toxicity
				2D6 substrate	3A4 substrate	1A2 inhibitor	2C19	2C9	2D6	3A4		
	Log mol/L	Numeric (% absorbed)	(logBB)	Categorical (yes/no)							Numeric (log ml/min/kg)	(Yes/No)
**10a**	-3.955	100	-1.175	no	yes	no	yes	yes	no	yes	0.087	no
**10b**	-3.952	100	-1.187	no	yes	no	yes	yes	no	yes	0.030	no
**10c**	-3.983	100	-1.161	no	yes	no	yes	yes	no	yes	0.037	no
**10d**	-4.136	100	-0.834	no	yes	no	yes	yes	no	yes	0.015	no
**10e**	-3.928	100	-1.442	no	yes	no	yes	yes	no	yes	0.125	no
**10f**	-3.969	100	-1.386	no	yes	no	yes	yes	no	yes	0.131	no
**10g**	-3.916	100	-1.395	no	yes	no	yes	yes	no	yes	-0.056	no
**10h**	-3.987	100	-1.369	no	yes	no	yes	yes	no	yes	-0.050	no
**10i**	-3.942	100	-1.596	no	yes	no	yes	yes	no	yes	0.041	no

**10j**	-3.982	100	-1.336	no	yes	no	yes	yes	no	yes	-0.038	no
**11a**	-4.201	85.54	-0.271	no	yes	no	no	yes	no	yes	0.295	no
**11b**	-4.187	85.95	-0.275	no	yes	no	no	yes	no	yes	0.298	no
**11c**	-4.202	85.94	-0.251	no	yes	no	no	yes	no	yes	0.307	no
**11d**	-4.707	87.52	-0.231	no	yes	no	no	yes	no	yes	0.289	no
**11e**	-4.332	84.79	-0.484	no	yes	no	no	yes	no	yes	0.275	no
**11f**	-4.260	87.63	-0.438	no	yes	no	no	yes	no	yes	0.273	no
**11g**	-4.360	86.70	-0.441	no	yes	no	no	yes	no	yes	0.106	no
**11h**	-4.332	89.59	-0.417	no	yes	no	no	yes	no	yes	0.104	no
**11i**	-4.442	88.58	-0.592	no	yes	no	no	yes	no	yes	0.042	no
**11j**	-4.241	86.65	-0.371	no	yes	no	no	yes	no	yes	-0.007	no
**Moxifloxacin**	-3.489	95.77	-0.901	no	no	no	no	no	no	no	0.537	no

Analytical data of compound 10a-j

Ethyl 2-oxo-4-phenyl-6-(((4-phenyl-5-(pyridin-4-yl)-4H-1,2,4-triazol-3-yl)thio)methyl)-1,2,3,4-tetrahydropyrimidine-5-carboxylate (10a)

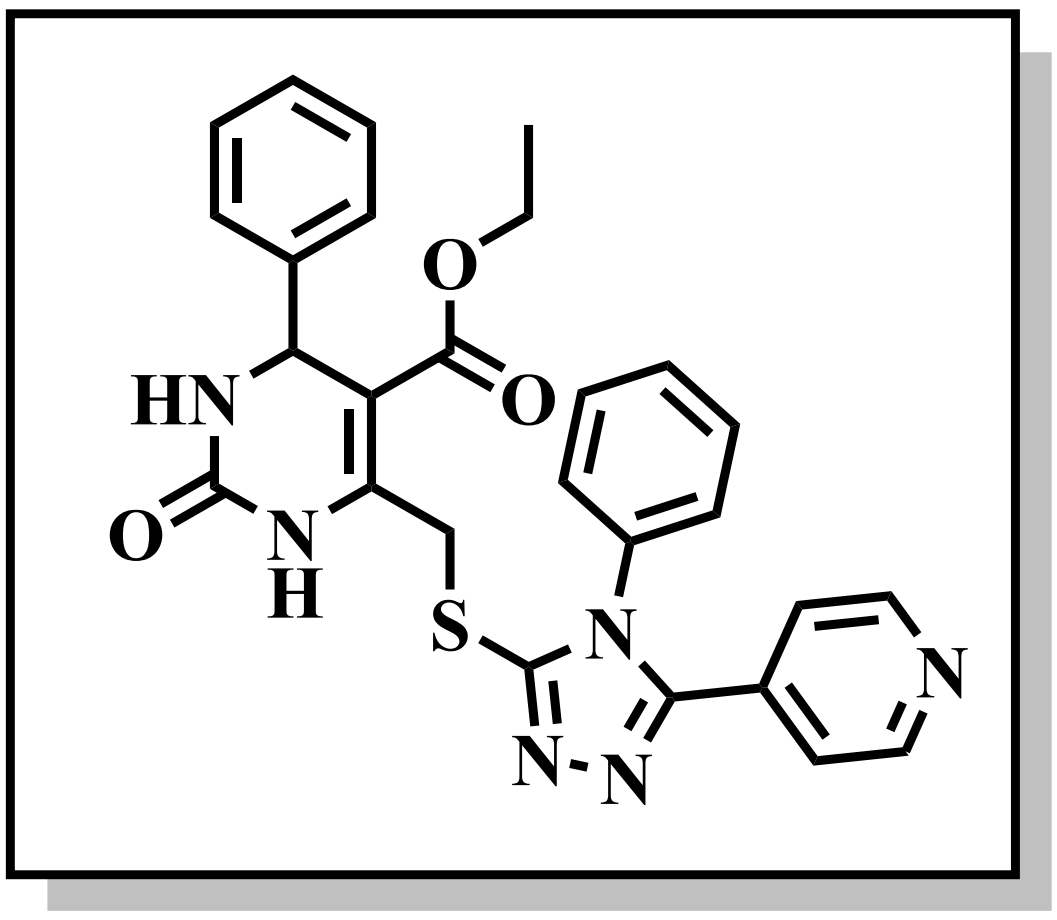

R*f* value: 0.37 (EtOAc, 100%)

Appearance: White solid

IR (APT): 1603, 1633, 1688, 2815, 2390, 3056, 3206 CM^{-1}.

^{1}H NMR (DMSO-d6, 600 MHz): 1.01 (t, *J* = 7.05 Hz, 3H), 3.89 (q, *J* = 7.11 Hz, 2H), 4.17 (d, *J* = 13.08 Hz, 1H), 4.40 (d, *J* = 13.08 Hz, 1H), 5.12 (d, *J* = 3.11 Hz, 1H), 7.23 (pseudo t, *J* = 7.71 Hz, 3H), 7.29 (dd, *J* = 4.12 Hz, 4H), 7.48 (d, *J* = 7.03 Hz, 2H), 7.58 (dd, *J* = 8.23 Hz, 3H), 7.84 (s, 1H), 8.57 (d, *J* = 5.60 Hz, 2H), 9.25 (s, 1H).

^{13}C APT NMR (DMSO-d6, 150 MHz): δ (ppm) 14.53 (1C), 32.53 (1C), 54.55 (1C), 60.20 1C), 63.24 (1C), 101.52 (1C), 121.97 (2C), 126.86 (2C), 127.96 (2C), 128.15 (2C), 128.92 (1C), 130.55 (1C), 130.94 (1C), 134.06 (1C), 134.36 (2C), 144.28 (2C), 147.28 (1C), 150.63 (1C), 152.22 (1C), 152.83 (1C), 153.08 (1C), 165.13 (1C).

HRMS: (ESI^+-MS, m/z) $[M + Na]^+$ calcd for $[C_{28}H_{26}N_6O_3]$: 535.15; found: 535.15.

Ethyl 2-oxo-6-(((4-phenyl-5-(pyridin-4-yl)-4H-1,2,4-triazol-3-yl)thio)methyl)-4-(p-tolyl)-1,2,3,4-tetrahydropyrimidine-5-carboxylate (10b)

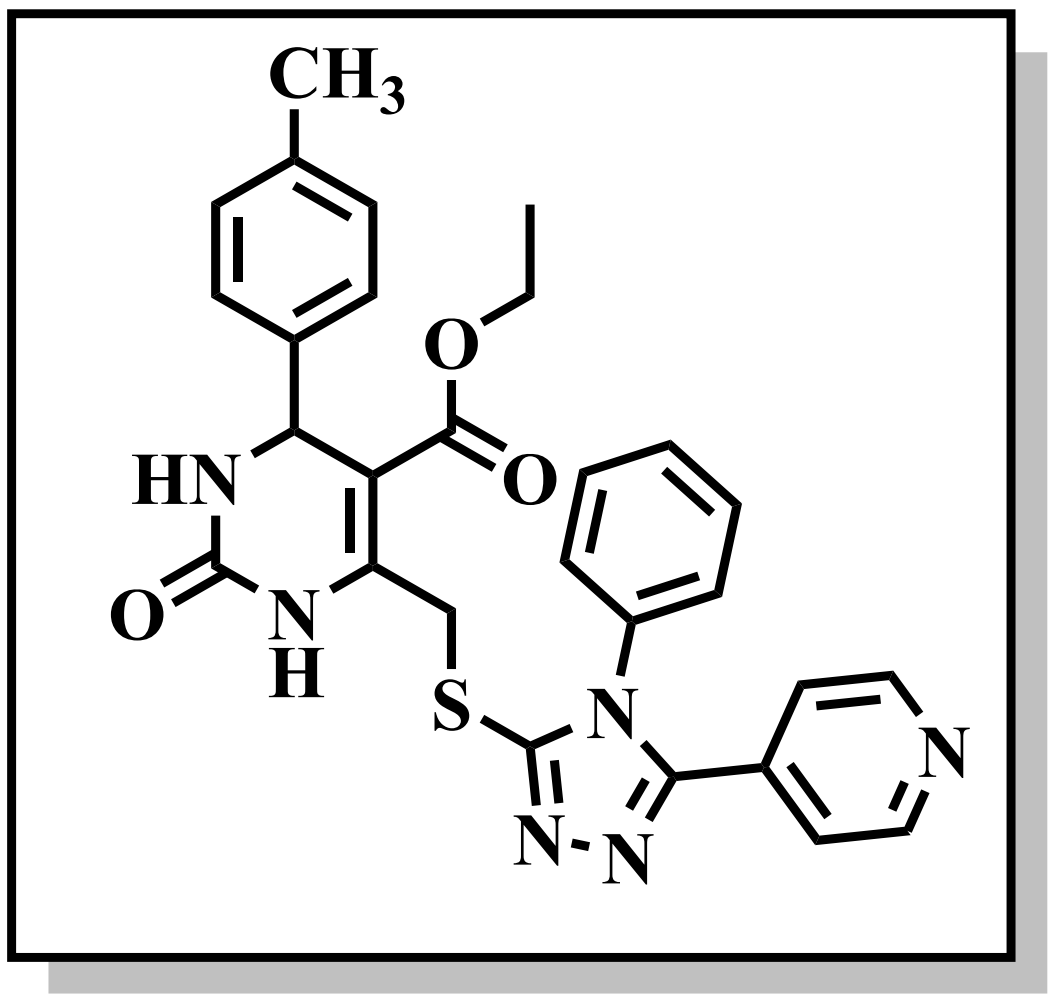

R*f* value: 0.45 (EtOAc, 100%)

Appearance: White solid

IR (APT): 1603 (C=C), 1636 (C=O), 1690 (C=O), 3360 (N-H) cm^{-1}.

^{1}H NMR (DMSO-d6, 600 MHz): δ (ppm) 1.03 (t, *J* = 6.98 Hz, 3H), 2.25 (s, 3H), 3.89-3.94 (m, 2H), 5.06 (d, *J* = 13.13 Hz, 1H), 5.48 (d, *J* = 13.13 Hz, 1H), 5.73 (d, *J* = 3.15 Hz, 1H), 7.05 (dd, *J* = 8.38 Hz, 4H), 7.29 (d, *J* = 5.98 Hz, 2H), 7.48 (d, *J* = 6.99 Hz, 2H), 7.58 (dd, *J* = 8.45 Hz, 3H), 7.79 (s, 1H), 8.58 (d, *J* = 5.92 Hz, 2H), 9.21 (s, 1H).

^{13}C NMR (DMSO-d6, 150 MHz): δ (ppm) 14.35 (1C), 21.11 (1C), 32.51 (1C), 54.20 (1C), 60.17 (1C), 101.63 (1C), 121.94 (2C), 126.76 (2C), 128.17 (2C), 129.44 (2C), 130.56 (1C), 130.94 (2C), 134.09 (1C), 134.36 (2C), 137.10 (1C), 141.72 (2C), 147.11 (1C), 150.66 (1C), 152.26 (1C), 152.80 (1C), 153.05 (1C), 165.15 (1C).

HRMS: (ESI^{+}-MS, m/z) $[M + Na]^{+}$ calcd for $[C_{29}H_{28}N_6O_3]$: 549.16; found: 549.16.

Ethyl 2-oxo-6-(((4-phenyl-5-(pyridin-4-yl)-4H-1,2,4-triazol-3-yl)thio)methyl)-4-(m-tolyl)-1,2,3,4-tetrahydropyrimidine-5-carboxylate (10c)

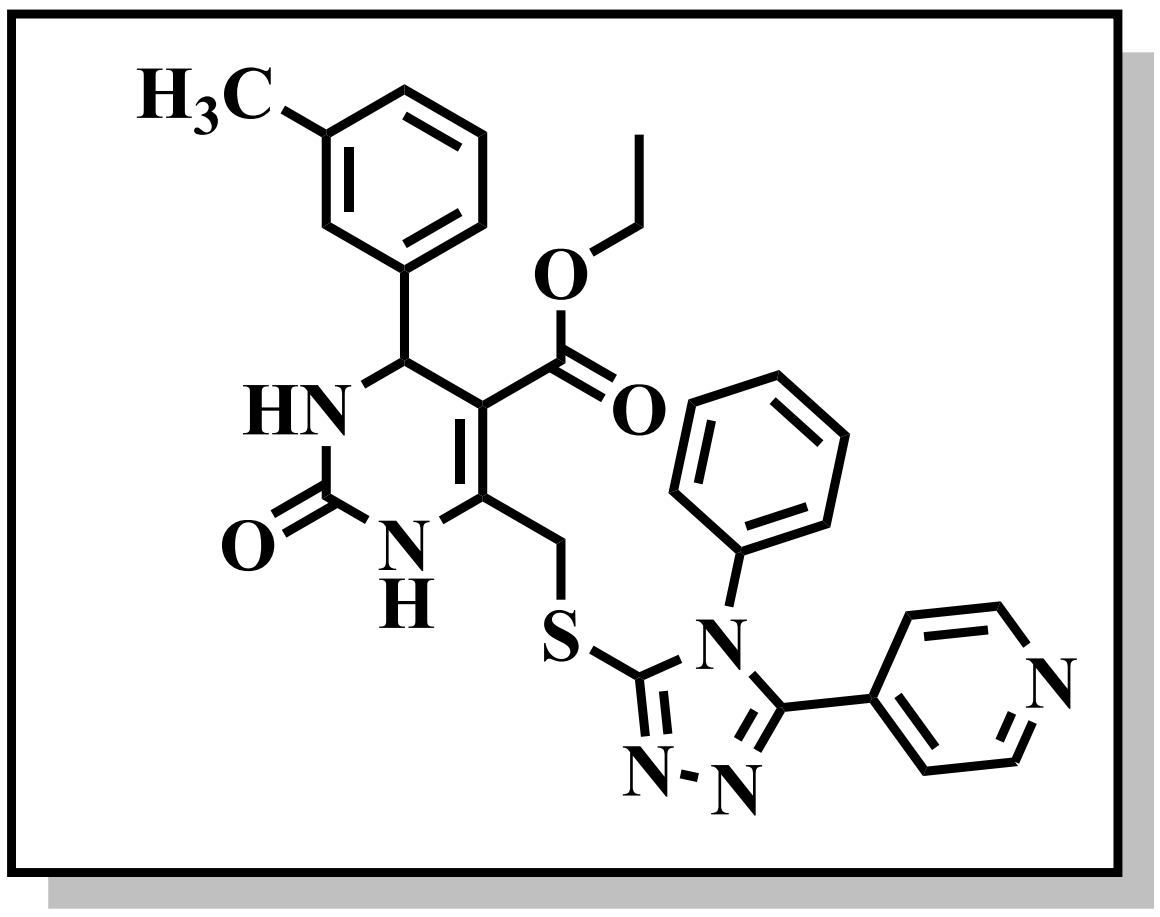

R*f* value: 0.42 (EtOAc, 100%)

Appearance: Yellow solid

IR (APT):1607, 1630, 1687, 2812, 2911, 3077, 3216 cm^{-1}.

^{1}H NMR (DMSO-d6, 600 MHz): δ (ppm) 1.03 (t, *J* = 6.90 Hz, 3H), 2.27 (s, 3H), 3.90 (q, *J* = 6.90 Hz, 2H), 4.05 (d, *J* = 13.12 Hz, 1H), 4.39 (d, *J* = 13.12 Hz, 1H), 5.11 (d, *J* = 2.57 Hz, 1H), 7.02 (dtd, *J* = 7.47 Hz, 3H), 7.18 (pseudo t, *J* = 7.48 Hz, 1H), 7.29 (d, *J* = 4.74 Hz, 2H), 7.49 (d, *J* = 6.81 Hz, 2H), 7.60 (d, *J* = 7.46 Hz, 3H), 7.79 (s, 1H), 8.58 (d, *J* = 5.94 Hz, 2H), 9.21 (s, 1H).

^{13}C NMR (DMSO-d6, 150 MHz): 14.33 (1C), 21.54 (1C), 32.47 (1C), 54.52 (1C), 60.15 (1C), 101.48 (1C), 121.95 (1C), 123.98 (1C), 127.49 (1C), 128.16 (2C), 128.57 (2C), 128.83 (1C), 130.55 (1C), 130.93 (2C), 134.09 (1C), 134.37 (2C), 137.95 (1C), 144.56 (2C), 147.22 (1C), 150.65 (1C), 152.21(1C), 152.76 (1C), 153.08 (1C), 165.13 (1C).

HRMS: (ESI$^+$-MS, m/z) $[M + Na]^+$ calcd for $[C_{29}H_{28}N_6O_3]$: 549.16; found: 549.28.

Ethyl 4-(4-isopropylphenyl)-2-oxo-6-(((4-phenyl-5-(pyridin-4-yl)-4H-1,2,4-triazol-3-yl)thio)methyl)-1,2,3,4-tetrahydropyrimidine-5-carboxylate (10d)

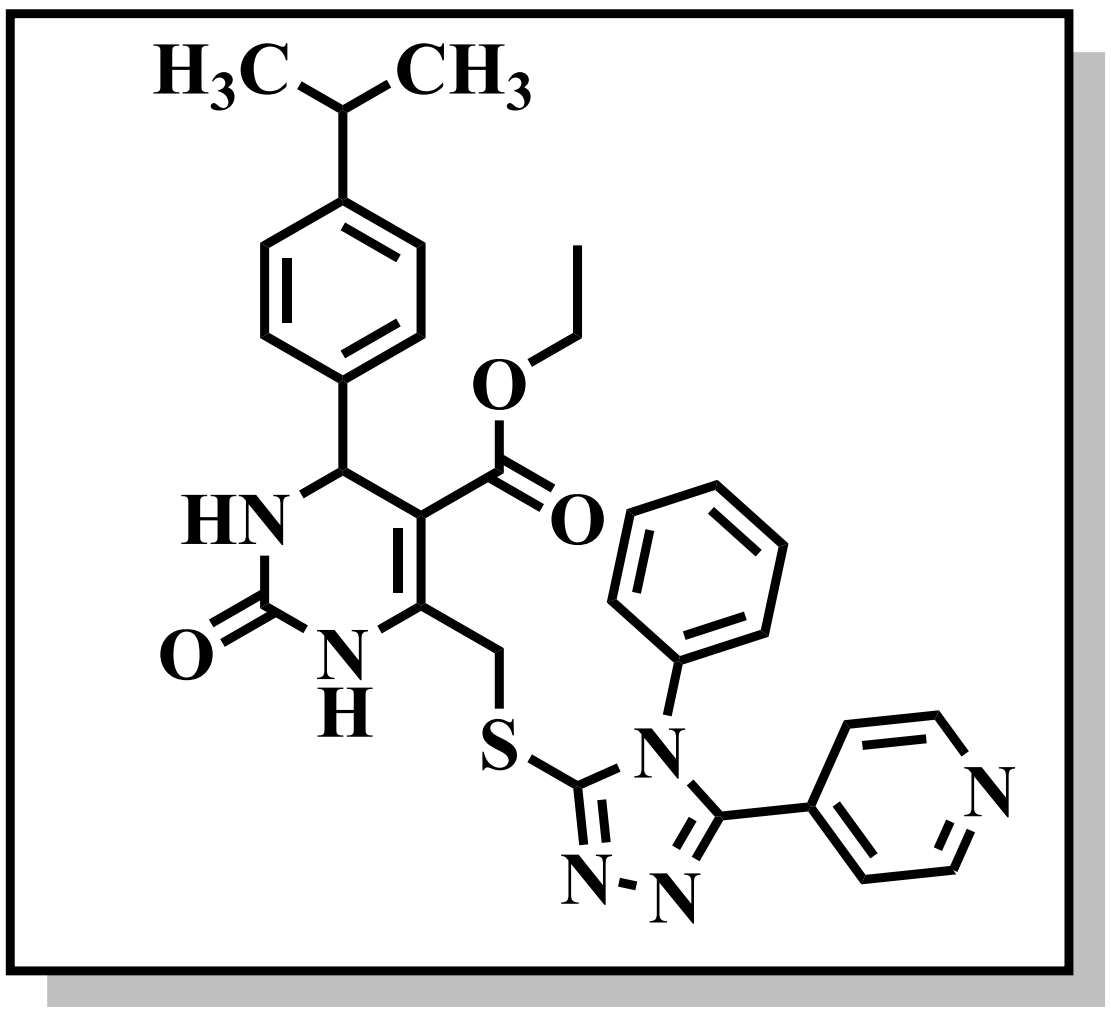

R*f* value: 0.42 (EtOAc, 100%)

Appearance: Yellow solid

IR (APT): 1601, 1643, 1692, 2866, 2957, 3077, 3228 cm^{-1}.

^{1}H NMR (DMSO-d6, 600 MHz): δ (ppm) 1.04 (t, *J* = 7.11 Hz, 3H), 1.15 (d, *J* = 6.91 Hz, 6H), 2.79 (sept, 1H), 3.90 (m, 2H), 4.06 (d, *J* = 13.02Hz, 1H), 4.40 (d, *J* = 13.02 Hz, 1H), 5.11 (d, *J* = 3.08 Hz, 1H),7.14 (m, 4H), 7.30 (d, *J* = 5.58 Hz, 2H), 7.45 (d, *J* = 7.11 Hz, 2H), 7.59 (m, 3H), 7.78 (s, 1H), 8.58 (d, *J* = 5.77 Hz, 2H), 9.22 (s, 1H).

^{13}C NMR (DMSO-d6, 150 MHz): δ (ppm) 14.34 (1C), 24.31 (2C), 33.55 (1C), 54.16 (1C), 60.20 (1C), 63.26 (1C), 101.65 (1C), 121.93 (2C), 126.79 (2C), 128.16 (2C), 130.55 (2C), 130.93 (2C), 134.09 (2C), 134.35 (2C), 142.11 (2C), 147.16 (1C), 148.08 (1C), 150.65 (1C), 152.33 (1C), 152.86 (1C), 153.04 (1C), 165.17 (1C).

HRMS: (ESI^{+}-MS, m/z) $[M + Na]^{+}$ calcd for $[C_{31}H_{32}N_{6}O_{3}]$: 577.19; found: 577.25.

Ethyl 4-(4-methoxyphenyl)-2-oxo-6-(((4-phenyl-5-(pyridin-4-yl)-4H-1,2,4-triazol-3-yl)thio)methyl)-1,2,3,4-tetrahydropyrimidine-5-carboxylate (10e)

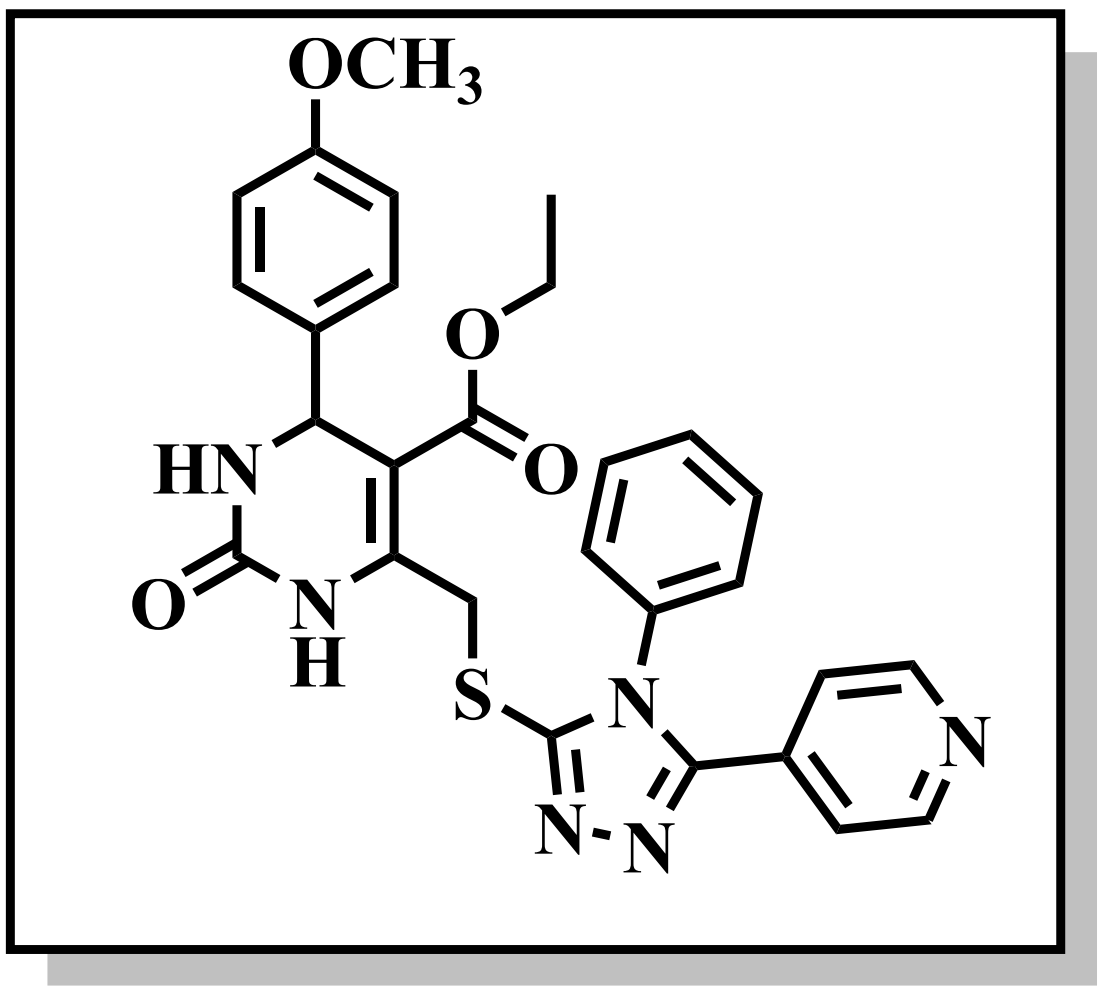

R*f* value: 0.32 (EtOAc, 100%)

Appearance: Pale yellow solid

IR (APT): 1602, 1638, 1689, 2962, 3109, 3217, 3356 cm^{-1}.

^{1}H NMR (DMSO-d6, 600 MHz): δ (ppm) 1.03 (t, *J* = 6.96 Hz, 3H), 3.71 (s, 3H), 3.90-3.93 (m, 2H), 4.06 (d, *J* = 13.13 Hz, 1H), 4.40 (d, *J* = 13.13 Hz, 1H), 5.10 (d, *J* = 3.05 Hz, 1H), 6.85 (d, *J* = 8.53 Hz, 2H),7.15 (d, *J* = 8.58 Hz, 2H), 7.29 (d, *J* = 5.69 Hz, 2H), 7.49 (d, *J* = 6.84 Hz, 2H), 7.59 (dd, *J* = 6.18 Hz, 3H), 7.77 (s, 1H), 8.58 (d, *J* = 5.65 Hz, 2H), 9.21 (s, 1H).

^{13}C NMR (DMSO-d6, 150 MHz): δ (ppm) 14.36 (1C), 19.02 (1C), 32.55 (1C), 53.92 (1C), 55.52 (1C), 60.16 (1C), 101.78 (1C), 114.23 (2C), 121.95 (2C), 128.05 (2C), 128.17 (2C), 130.55 (1C), 130.93 (1C), 134.09 (2C), 134.36 (1C), 136.80 (1C), 146.97 (2C), 150.65 (1C), 152.23 (1C), 152.85 (1C), 153.06 (1C), 159.05 (1C), 165.17 (1C).

HRMS: (ESI^{+}-MS, m/z) [M + Na]$^{+}$ calcd for [$C_{29}H_{28}N_6O_4$]: 565.16; found: 565.16.

Ethyl 4-(3-methoxyphenyl)-2-oxo-6-(((4-phenyl-5-(pyridin-4-yl)-4H-1,2,4-triazol-3-yl)thio)methyl)-1,2,3,4-tetrahydropyrimidine-5-carboxylate (10f)

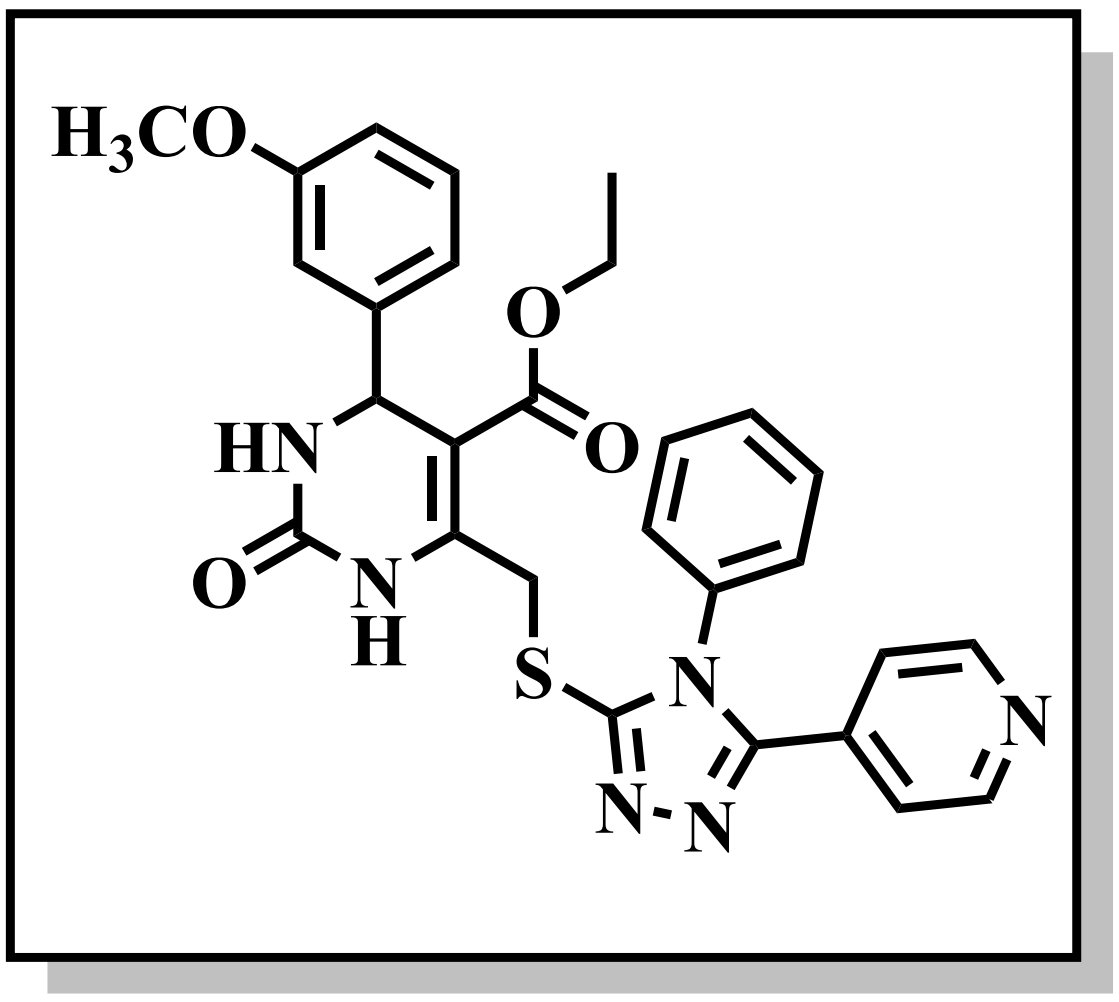

R*f* value: 0.37 (EtOAc, 100%)

Appearance: Light yellow solid

IR (APT): 1603, 1636, 1683, 2846, 2919, 3063, 3203 cm^{-1}.

^{1}**H NMR** (DMSO-d6, 600 MHz): δ (ppm) 1.04 (t, *J* = 7.19 Hz, 3H), 3.70 (s, 3H), 3.92 (q, *J* = 6.95 Hz, 2H), 4.10 (d, *J* = 13.04 Hz, 1H), 4.38 (d, *J* = 13.04 Hz, 1H), 5.13 (d, *J* = 3.12 Hz, 1H), 7.22 (pseudo t, *J* = 7.86 Hz, 2H), 7.29 (d, *J* = 5.65 Hz, 2H), 7.49 (d, *J* = 7.03 Hz, 2H), 7.59 (dd, *J* = 7.11 Hz, 3H), 7.83 (s, 1H), 8.58 (d, *J* = 5.64 Hz, 2H), 9.25 (s, 1H).

^{13}**C NMR** (DMSO-d6, 150 MHz): 14.36 (1C), 19.02 (1C), 32.43 (1C), 54.33 (1C), 55.43 (1C), 56.49 (1C), 60.21 (1C), 101.25 (1C), 112.61 (1C), 113.13 (1C), 118.93 (1C), 121.95 (2C), 128.15 (2C), 130.09 (1C), 130.57 (1C), 130.94 (1C), 134.07 (1C), 134.34 (1C), 146.05 (2C), 147.47 (1C), 150.66 (1C), 152.27 (1C), 152.78 (1C), 153.07 (1C), 159.72 (1C), 165.12 (1C).

HRMS: (ESI^{+}-MS, m/z) $[M + Na]^{+}$ calcd for $[C_{29}H_{28}N_6O_4]$: 565.16; found: 565.16.

Ethyl 4-(4-fluorophenyl)-2-oxo-6-(((4-phenyl-5-(pyridin-4-yl)-4H-1,2,4-triazol-3-yl)thio)methyl)-1,2,3,4-tetrahydropyrimidine-5-carboxylate (10g)

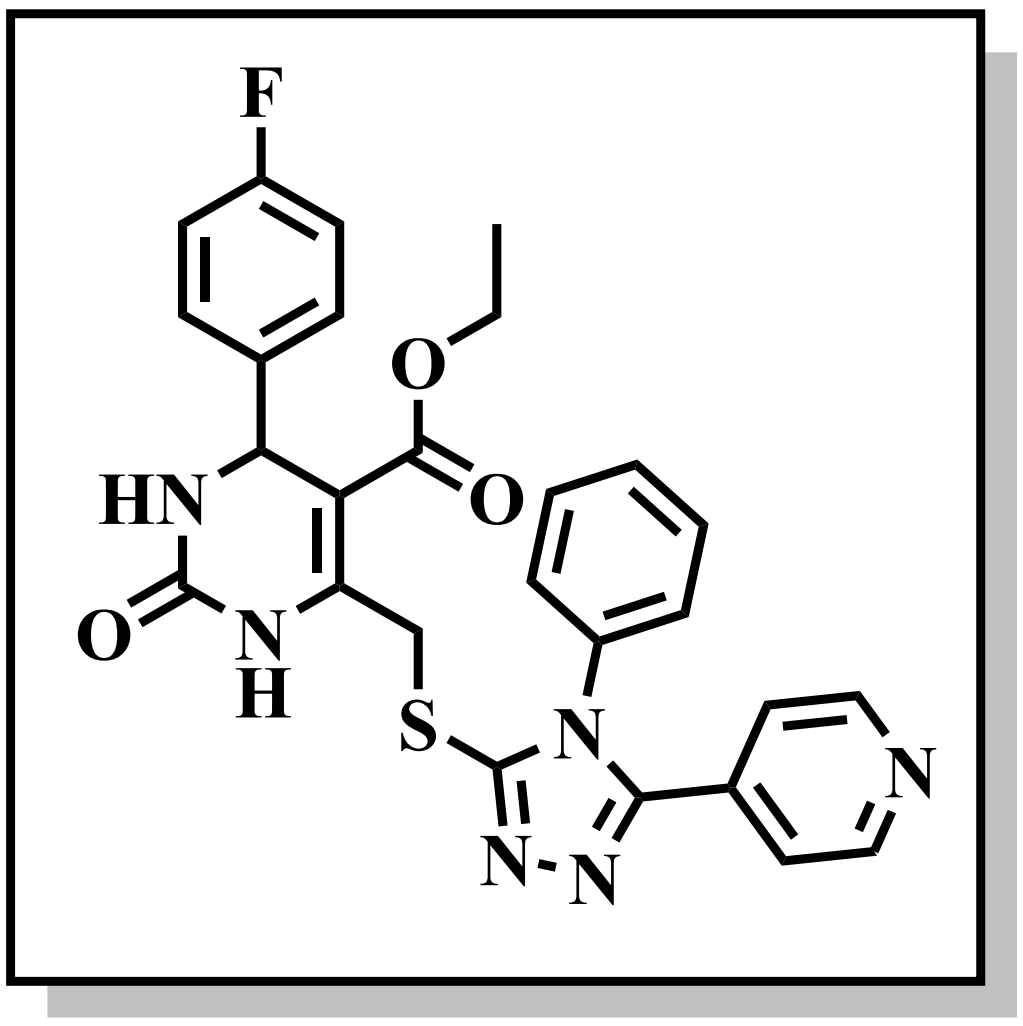

R*f* value: 0.34 (EtOAc, 100%)

Appearance: Light brown solid

IR (APT): 1600, 1636, 1691, 2925, 3117, 3225, 3346 cm^{-1}.

^{1}**H NMR** (DMSO-d6, 600 MHz): δ (ppm) 1.01 (t, *J* = 7.12 Hz, 3H), 3.89 (q, *J* = 6.98 Hz, 2H), 4.05 (d, *J* = 13.16 Hz, 1H), 4.37 (d, *J* = 13.16 Hz, 1H), 5.16 (d, *J* = 3.11 Hz, 1H), 7.13 (pseudo t, *J* = 8.77 Hz, 2H), 7.27 (dtd, *J* = 5.57 Hz, 4H), 7.49 (d, *J* = 6.77 Hz, 2H), 7.59 (dd, *J* = 7.57 Hz, 3H), 7.84 (s, 1H), 8.58 (d, *J* = 5.87 Hz, 2H), 9.28 (s, 1H).

^{13}**C NMR** (DMSO-d6, 150 MHz): δ (ppm) 14.32 (1C), 14.38 (1C), 32.56 (1C), 53.91 (1C), 60.20 (1C), 63.26 (1C), 101.25 (1C), 115.58 ($J_{C\text{-}F}$ = 30 Hz, 2C), 121.94 (1C), 128.18 ($J_{C\text{-}F}$ = 90.50 Hz, 1C), 128.90 ($J_{C\text{-}F}$ = 9.0 Hz, 2C), 130.54 (1C), 130.93 (1C), 134.11 (1C), 134.36 (1C), 140.90 ($J_{C\text{-}F}$ = 15 Hz, 1C), 147.55 (1C), 150.66 (1C), 152.05 (1C) 152.31 (1C), 152.40 (1C), 152.69 (1C), 153.09 (1C), 161.08 (1C), 162.669 (1C), 165.02 (1C).

HRMS: (ESI^{+}-MS, m/z) $[M + Na]^{+}$ calcd for [$C_{28}H_{25}FN_6O_3$]: 553.14; found: 553.27.

Ethyl 4-(3-fluorophenyl)-2-oxo-6-(((4-phenyl-5-(pyridin-4-yl)-4H-1,2,4-triazol-3-yl)thio)methyl)-1,2,3,4-tetrahydropyrimidine-5-carboxylate (10h)

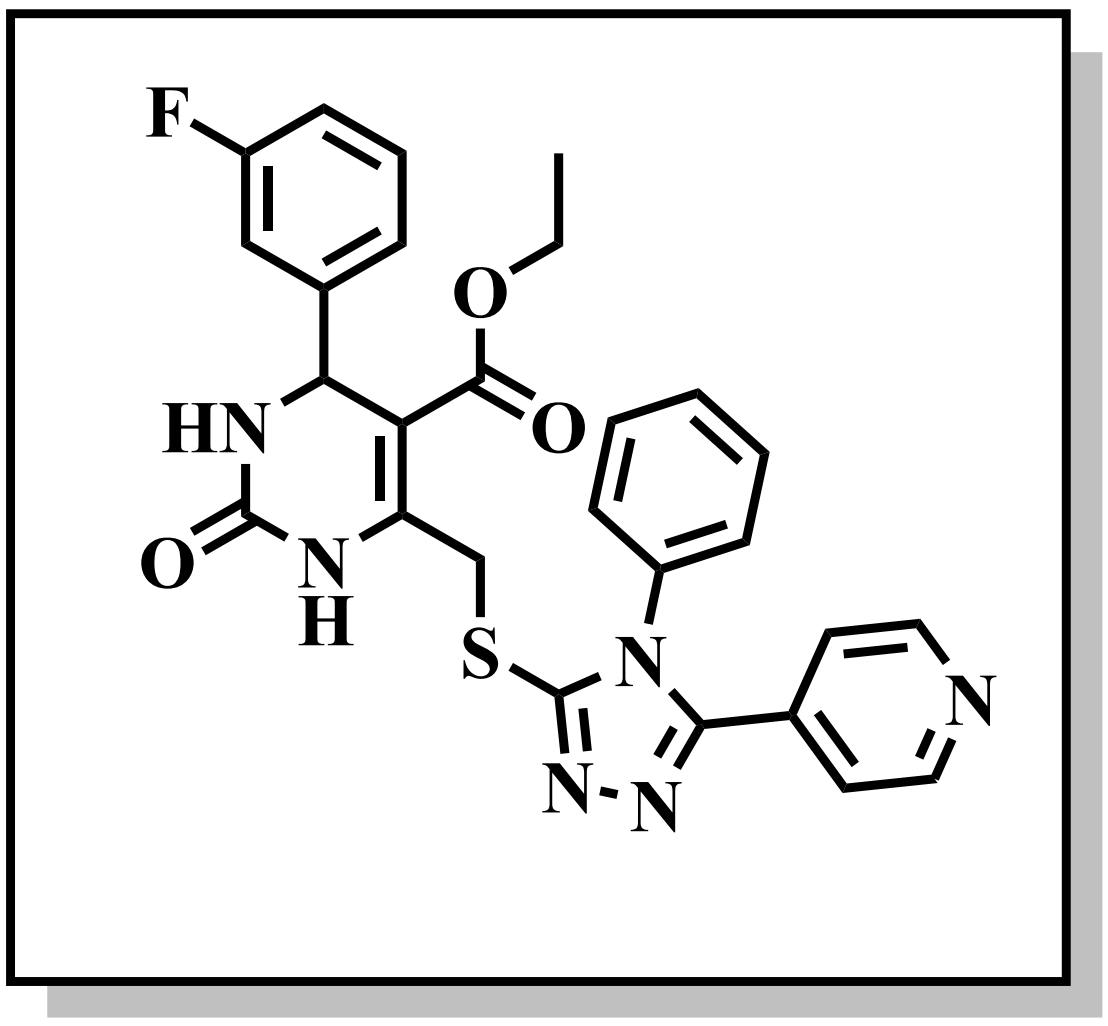

R*f* value: 0.48 (EtOAc, 100%)

Appearance: White solid

IR (APT): 1605, 1632, 1680, 2809, 2923, 3059, 3217 cm^{-1}.

^{1}H NMR (DMSO-d6, 600 MHz): δ (ppm) 1.02 (t, *J* = 7.08 Hz, 3H), 3.91 (q, *J* = 6.85 Hz, 2H), 4.08 (d, *J* = 13.08 Hz, 1H), 4.36 (d, *J* = 13.08 Hz, 1H), 5.18 (d, *J* = 3.09 Hz, 1H), 7.03 (ddd, *J* = 8.14 Hz, 3H),7.29 (d, *J* = 5.78 Hz, 2H), 7.35 (dd, *J* = 7.86 Hz, 1H), 7.49 (d, *J* = 6.78 Hz, 2H), 7.59 (dd, *J* = 6.96 Hz, 3H), 7.89 (s, 1H), 8.58 (d, *J* = 5.62 Hz, 2H), 9.32 (s, 1H).

^{13}C NMR (DMSO-d6, 150 MHz): δ (ppm) 14.32 (1C), 32.52 (1C), 54.07 (1C), 60.25 (1C), 100.75 (1C), 113.57 (J_{C-F} = 21.0 Hz, 1C), 114.69 (J_{C-F} = 21.0 Hz, 1C), 121.95 (1C), 122.94 (1C), 128.17 (1C), 130.55 (2C), 130.93 (2C), 130.97 (2C), 131.03 (1C), 134.11(J_{C-F} = 39.0 Hz 1C), 147.33 (1C), 147.37 (1C), 148.02 (1C), 150.65 (1C), 152.08 (1C), 152.65 (1C), 153.10 (1C), 161.79 (1C), 163.40 (1C), 164.99 (1C).

HRMS: (ESI^+-MS, m/z) $[M + Na]^+$ calcd for [$C_{28}H_{25}FN_6O_3$]: 553.14; found: 553.27.

Ethyl 4-(2,4-difluorophenyl)-2-oxo-6-(((4-phenyl-5-(pyridin-4-yl)-4H-1,2,4-triazol-3-yl)thio)methyl)-1,2,3,4-tetrahydropyrimidine-5-carboxylate (10i)

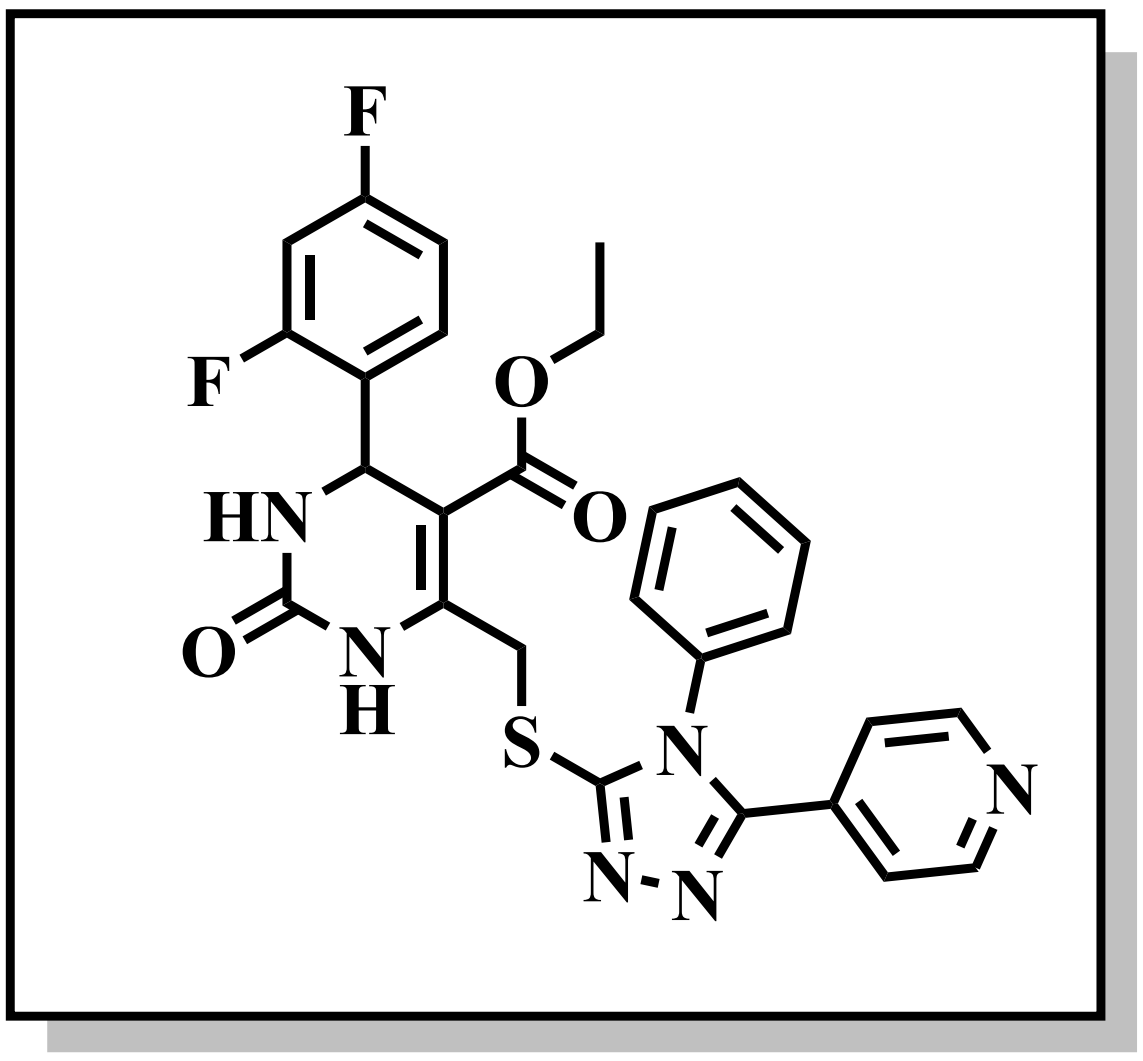

R*f* value: 0.34 (EtOAc, 100%)

Appearance: Light brown solid

IR (APT): 1602, 1638, 1695, 2847, 2923, 3106, 3202, 3355 cm^{-1}.

^{1}H NMR (DMSO-d6, 600 MHz): δ (ppm) 0.97 (t, *J* = 7.10 Hz, 3H), 3.85 (q, *J* = 6.84 Hz, 2H), 4.07 (d, *J* = 13.10 Hz, 1H), 4.34 (d, *J* = 13.10 Hz, 1H), 5.44 (d, *J* = 2.66 Hz, 1H), 7.02 (ddd, *J*= 6.65 Hz, 1H), 7.18 (ddd, *J* = 2.13 Hz, *J* = 11.21 Hz, 1H),7.29 (d, *J* = 5.97 Hz, 2H), 7.35 (dd, *J* = 8.52 Hz, 1H), 7.51 (d, *J* = 6.56 Hz, 2H), 7.59 (m, 3H), 7.81 (s, 1H), 8.58 (d, *J* = 5.87 Hz, 2H), 9.33 (s, 1H).

^{13}C NMR (DMSO-d6, 150 MHz): δ (ppm) 14.15 (1C), 32.49 (1C), 48.67 (1C), 60.12 (1C), 63.27 (1C), 99.52 (1C), 104.22 (1C), 112.04 (J_{C-F} = 21.0 Hz, 2C), , 121.95 (2C), 128.19 (2C), 130.54 (2C), 130.93 (2C), 134.12 (2C), 134.37 (2C), 148.16 (2C), 150.65 (1), 152.59 (1), 152.66 (1C), 153.12 (1C), 164.72 (1C).

HRMS: (ESI^{+}-MS, m/z) $[M + Na]^{+}$ calcd for $[C_{28}H_{24}F2N_{6}O_{3}]$: 571.13; found: 571.25.

Ethyl 4-(3-chlorophenyl)-2-oxo-6-(((4-phenyl-5-(pyridin-4-yl)-4H-1,2,4-triazol-3-yl)thio)methyl)-1,2,3,4-tetrahydropyrimidine-5-carboxylate (10j)

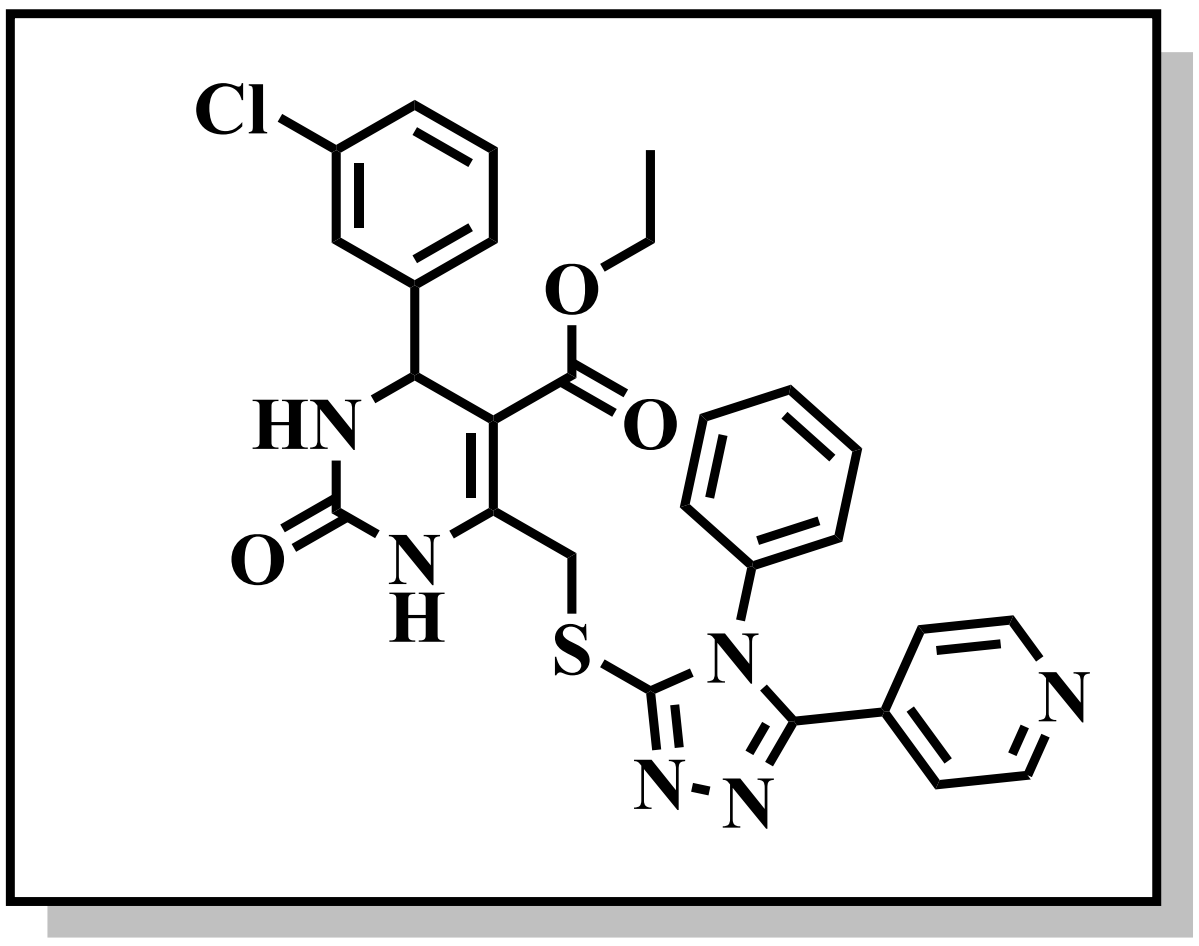

R*f* value: 0.48 (EtOAc, 100%)

Appearance: Yellow solid

IR (APT): 1609, 1633, 1690, 2919, 3058, 3232 cm^{-1}.

^{1}H NMR (DMSO-d6, 600 MHz): δ (ppm) 1.03 (t, *J* = 7.01 Hz, 3H), 3.90-3.96 (m, 2H), 4.09 (d, *J* = 13.12 Hz, 1H), 4.38 (d, *J* = 13.12 Hz, 1H), 5.17 (d, *J* = 3.20 Hz, 1H), 7.22 (d, *J* = 7.52 Hz, 1H), 7.28 (pseudo t, *J* = 6.00 Hz, 3H), 7.29 (dtd, *J* = 7.85 Hz, 2H), 7.49 (d, *J* = 6.69 Hz, 2H), 7.59 (Dd, *J* = 6.56 Hz, 3H), 7.88 (s, 1H), 8.58 (d, *J* = 5.95 Hz, 2H), 9.31 (s, 1H).

^{13}C NMR (DMSO-d6, 150 MHz): 14.30 (1C), 32.46 (1C), 54.17 (1C), 60.26 (1C), 100.75 (1C), 121.95 (2C), 125.57 (1C), 126.89 (2C), 127.92 (1C), 128.14 (2C), 130.55 (2C), 130.93 (1C), 130.96 (1C), 133.45 (1C), 134.08 (1C), 134.37 (1C), 146.94 (2C), 148.00 (1C), 150.64 (1C), 152.00 (1C), 152.71 (1C), 153.09 (1C), 164.95 (1C).

HRMS: (ESI^{+}-MS, m/z) $[M + Na]^{+}$ calcd for [$C_{28}H_{25}ClN_6O_3$]: 569.11; found: 569.25.

Analytical data of compound 11a-j

Ethyl 6-(((1H-benzo[d][1,2,3]triazol-1-yl)oxy)methyl)-2-oxo-4-phenyl-1,2,3,4-tetrahydropyrimidine-5-carboxylate (11a)

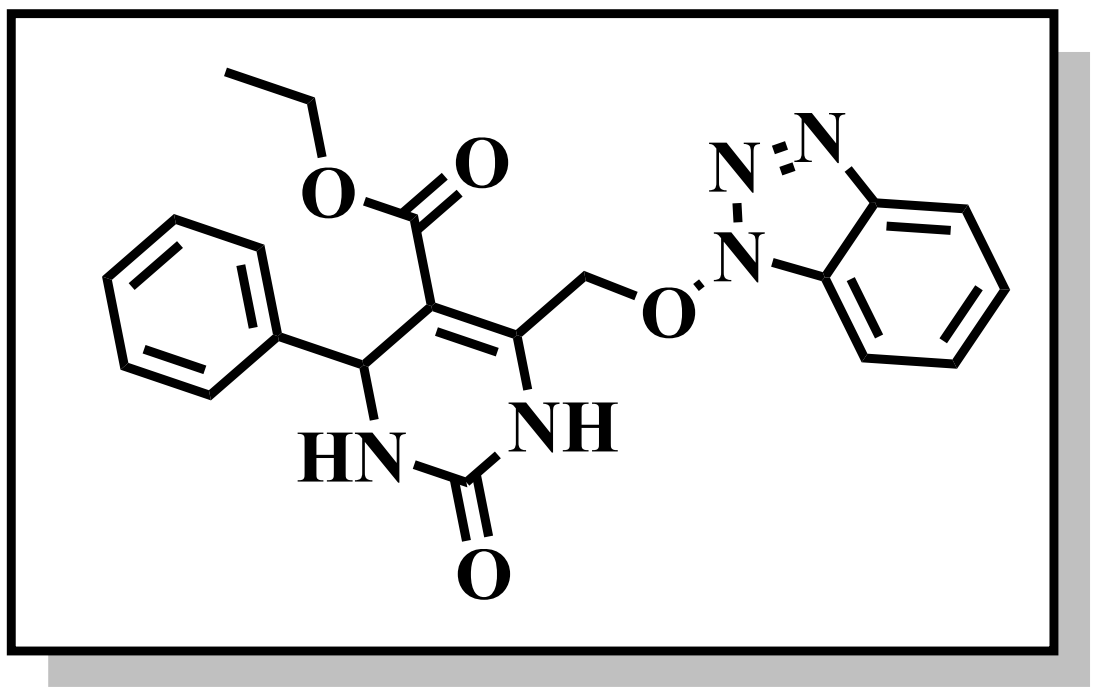

R*f* value: 0.13 (Hex/EtOAc, 40%)

Appearance: Light yellow solid

IR (APT): 1654, 1701, 2832, 2935, 3086, 3210 cm^{-1}.

^{1}H NMR (DMSO-d_6, 600 MHz): δ (ppm) 0.91 (t, J = 7.04 Hz, 3H), 3.75 (m, 2H), 5.11 (d, J = 3.18 Hz, 1H), 5.50 (d, J = 11.88 Hz, 1H), 5.74 (d, J = 11.88 Hz, 1H), 7.17 (d, J = 7.20 Hz, 2H), 7.24–7.30 (m, 3H), 7.45 (t, J = 7.50 Hz, J = 15.21 Hz, 1H), 7.59 (t, J = 7.75 Hz, J = 15.17 Hz, 1H), 7.77 (d, J = 8.23 Hz, 1H), 7.82 (d, J = 2.31 Hz, 1H), 8.05 (d, J = 8.58 Hz, 1H), 9.80 (s, 1H).

^{13}C CPD NMR (DMSO-d_6, 150 MHz): δ (ppm) 14.11 (1C), 54.67 (1C), 60.44 (1C), 75.19 (1C), 105.57 (1C), 109.69 (1C), 120.09 (2C), 125.38 (1C), 126.77 (2C), 128.03 (2C), 128.80 (1C), 128.92 (1C), 142.27 (1C), 143.12 (1C), 144.02 (1C), 152.43 (1C), 164.55 (1C).

^{13}C APT NMR (DMSO-d_6, 150 MHz): δ (ppm) 14.11 (1C), 54.67 (1C), 60.44 (1C), 75.19 (1C), 105.57 (1C), 109.69 (1C), 120.09 (2C), 125.38 (1C), 126.77 (2C), 128.03 (2C), 128.80 (1C), 128.92 (1C), 142.27 (1C), 143.12 (1C), 144.02 (1C), 152.43 (1C), 164.55 (1C).

HRMS: (ESI$^+$-MS, m/z) $[M + Na]^+$ calcd for $[C_{20}H_{19}N_5O_4]$: 416.14; found: 416.13.

Ethyl 6-(((1H-benzo[d][1,2,3]triazol-1-yl)oxy)methyl)-2-oxo-4-(p-tolyl)-1,2,3,4-tetrahydropyrimidine-5-carboxylate (11b)

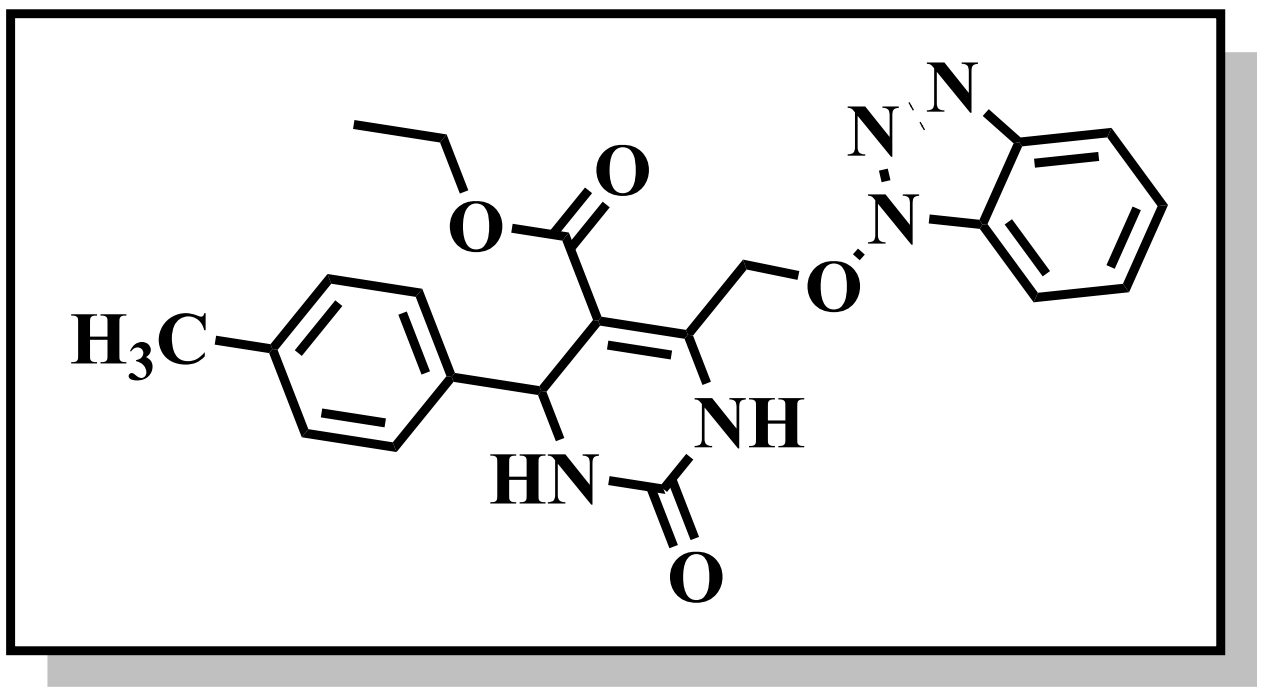

R*f* value: 0.17 (Hex/EtOAc, 40%)

Appearance: White solid

IR (APT): 1657, 1702, 2821, 2956, 3126, 3236 cm^{-1}.

^{1}**H NMR** (DMSO-d_6, 600 MHz): δ (ppm) 0.92 (t, *J* = 6.96 Hz, 3H), 2.25 (s, 3H), 3.74 (m, 2H), 5.06 (d, *J* = 3.09 Hz, 1H), 5.48 (d, *J* = 11.88 Hz, 1H), 5.73 (d, *J* = 11.88 Hz, 1H), 7.05(q, *J* = 8.07 Hz, 4H), 7.45 (t, *J* = 7.52 Hz, *J* = 15.20 Hz, 1H), 7.59 (t, *J* = 7.74 Hz, *J* = 15.22 Hz, 1H), 7.76 (d, *J* = 8.60 Hz, 1H), 7.77 (merge s, 1H), 8.05 (d, *J* = 8.44 Hz, 1H), 9.76 (s, 1H).

^{13}**C NMR** (DMSO-d_6, 150 MHz): δ (ppm) 14.13 (1C), 21.11 (1C), 54.33 (1C), 60.43 (1C), 75.19 (1C), 105.75 (1C), 109.96 (1C), 120.08 (2C), 125.37 (1C), 126.66 (2C), 127.73 (1C), 128.80 (2C), 129.43 (1C), 137.22 (1C), 141.11 (1C), 142.05 (1C), 143.11 (1C), 152.47 (1C), 164.58 (1C).

HRMS: (ESI^+-MS, m/z) $[M + Na]^+$ calcd for [$C_{21}H_{21}N_5O_4$]: 430.15; found: 430.14.

Ethyl 6-(((1H-benzo[d][1,2,3]triazol-1-yl)oxy)methyl)-2-oxo-4-(m-tolyl)-1,2,3,4-tetrahydropyrimidine-5-carboxylate (11c)

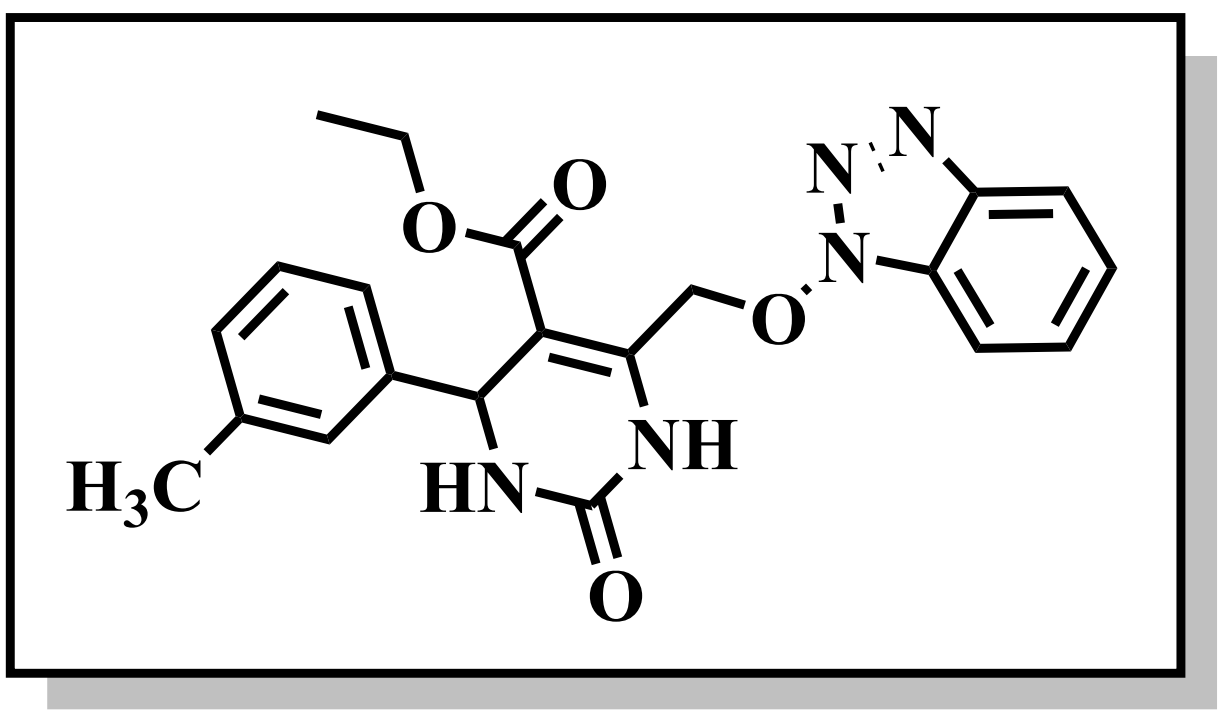

R*f* value: 0.11 (Hex/EtOAc, 40%)

Appearance: White solid

IR (APT): 1654, 1701, 2835, 3942, 3091, 3218 cm^{-1}.

^{1}H NMR (DMSO-d_6, 600 MHz): δ (ppm) 0.91 (t, *J* = 7.01 Hz, 3H), 2.26 (s, 3H), 3.75 (m, 2H), 5.08 (d, *J* = 2.47 Hz, 1H), 5.50 (d, *J* = 11.82 Hz, 1H), 5.73 (d, *J* = 11.82 Hz, 1H), 6.98 (d, *J* = 7.52 Hz, 1H), 7.06 (s, 2H), 7.15 (t, *J* = 7.61 Hz, *J* = 15.35 Hz, 1H), 7.45 (t, *J* = 7.51 Hz, *J* = 15.13 Hz, 1H), 7.77 (t, *J* = 7.73 Hz, *J* = 15.13 Hz,1H), 7.82 (t, *J* = 8.34 Hz, 2H), 8.05 (d, *J* = 8.34 Hz, 1H), 9.78 (s, 1H).

^{13}C NMR (DMSO-d_6, 150 MHz): 14.10 (1C), 21.53 (1C), 54.69 (1C), 60.41 (1C), 75.20 (1C), 105.56 (1C), 109.67 (1C), 120.09 (1C), 123.90 (1C), 125.37 (1C), 127.42 (1C), 127.73 (1C), 128.68 (1C), 128.79 (1C), 128.83 (1C), 138.03 (1C), 142.19 (1C), 143.12 (1C), 144.03 (1C), 152.44 (1C), 164.58 (1C).

HRMS: (ESI^+-MS, m/z) $[M + Na]^+$ calcd for $[C_{21}H_{21}N_5O_4]$: 430.14; found: 430.14.

Ethyl 6-(((1H-benzo[d][1,2,3]triazol-1-yl)oxy)methyl)-4-(4-isopropylphenyl)-2-oxo-1,2,3,4-tetrahydropyrimidine-5-carboxylate (11d)

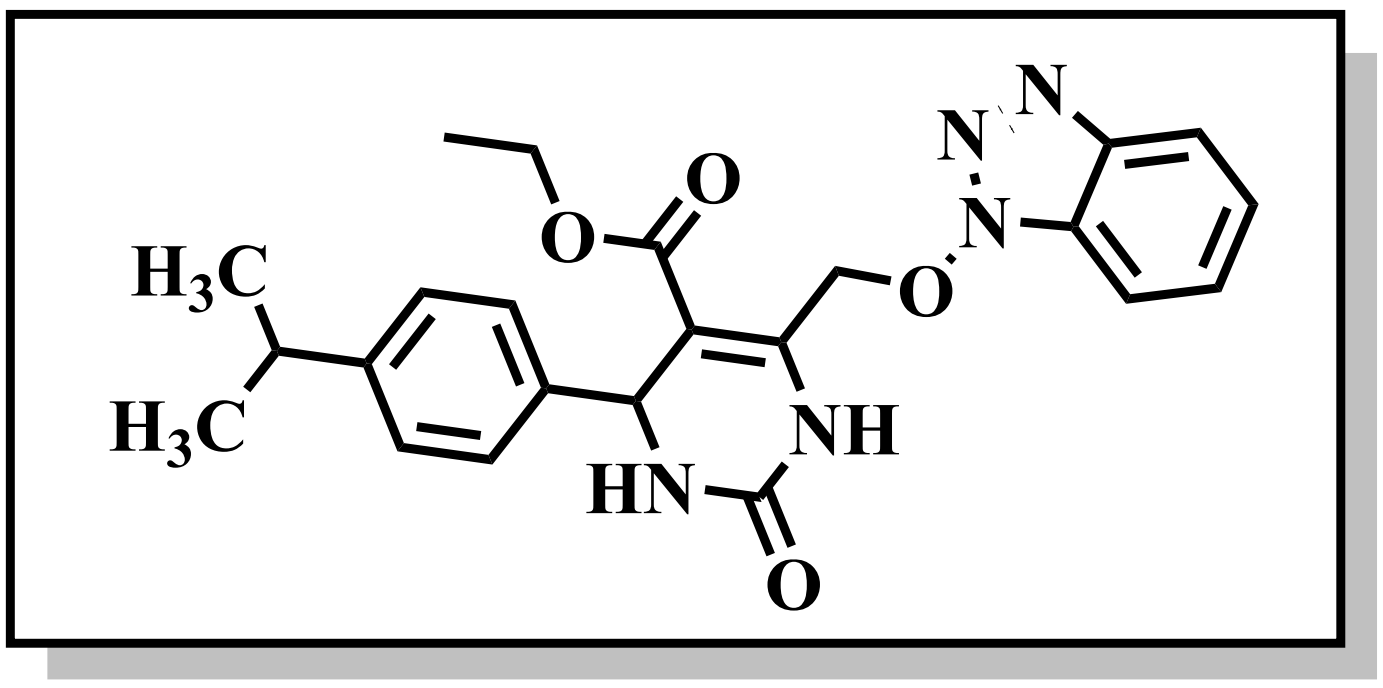

R*f* value: 0.23 (Hex/EtOAc, 40%)

Appearance: Yellow solid

IR (APT): 1655, 1702, 1735, 2969, 3097, 3223 cm^{-1}.

^{1}H NMR (DMSO-d_6, 600 MHz): δ (ppm) 0.92 (t, J = 7.16 Hz, 3H), 1.15 (d, J = 6.83 Hz, 6H), 2.81 (m, 1H), 3.73 (m, 2H), 5.07 (d, J = 3.20 Hz, 1H), 5.48 (d, J = 11.91 Hz, 1H), 5.74 (d, J = 11.91 Hz, 1H),7.08(d, J = 8.31 Hz, 4H), 7.17 (d, J = 8.10 Hz, 1H), 7.45 (t, J = 7.67 Hz, J = 15.34 Hz, 1H), 7.59 (t, J = 7.80 Hz, J = 15.24 Hz, 1H), 7.77 (m, 2H), 8.05 (d, J = 8.36 Hz, 1H), 9.80 (s, 1H).

^{13}C NMR (DMSO-d_6, 150 MHz): δ (ppm) 14.13 (1C), 24.32 (2C), 33.54 (1C), 54.28 (1C), 60.47 (1C), 75.22 (1C), 105.75 (1C), 109.71 (1C), 120.08 (2C), 125.38 (1C), 126.69 (2C), 126.82 (1C), 127.73 (1C), 128.80 (1C), 141.50 (1C), 142.10 (1C), 143.11 (1C), 148.20 (1C), 152.56 (1C), 164.60 (1C).

HRMS: (ESI$^+$-MS, m/z) $[M + Na]^+$ calcd for $[C_{23}H_{25}N_5O_4]$: 458.18; found: 458.18.

Ethyl 6-(((1H-benzo[d][1,2,3]triazol-1-yl)oxy)methyl)-4-(4-methoxyphenyl)-2-oxo-1,2,3,4-tetrahydropyrimidine-5-carboxylate (11e)

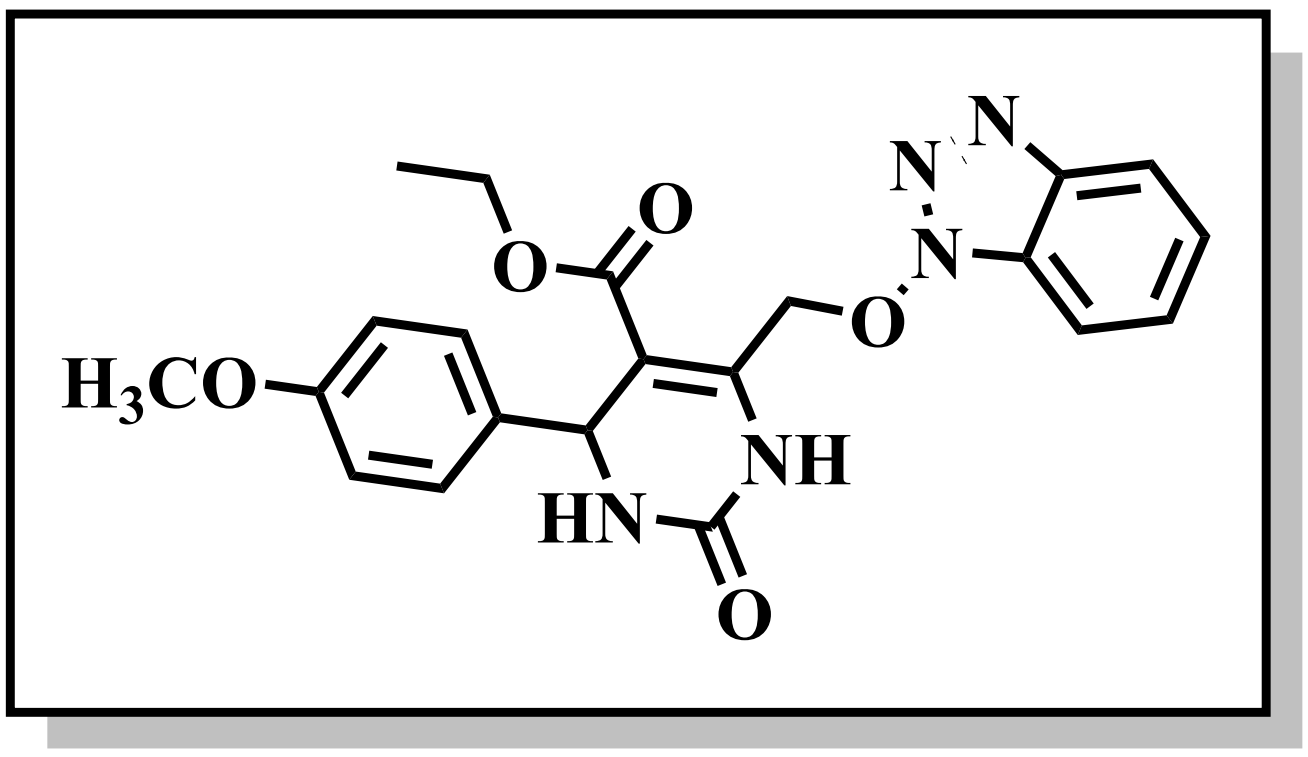

R*f* value: 0.13 (Hex/EtOAc, 40%)

Appearance: Light yellow solid

IR (APT): 1631, 1680, 2828, 2964, 3152, 3212, 3349 cm^{-1}.

^{1}H NMR (DMSO-d_6, 600 MHz): δ (ppm) 0.92 (t, J = 7.10 Hz, J = 14.21 Hz, 3H), 3.71 (s, 3H), 3.74 (m, 2H), 5.06 (d, J = 2.77 Hz, 1H), 5.50 (d, J = 11.87 Hz, 1H), 5.73 (d, J = 11.87 Hz, 1H), 6.81 (d, J = 8.55 Hz, 2H), 7.08 (d, J = 8.48 Hz, 2H), 7.45 (t, J = 7.66 Hz, J = 15.30 Hz, 1H), 7.59 (t, J = 7.78 Hz, J = 15.21 Hz, 1H), 7.76 (m, 2H), 8.05 (d, J = 8.39 Hz, 1H), 9.76 (s, 1H).

^{13}C NMR (DMSO-d_6, 150 MHz): δ (ppm) 14.14 (1C), 54.05 (1C), 55.53 (1C), 60.42 (1C), 75.20 (1C), 105.90 (1C), 109.71 (1C), 114.22 (2C), 120.09 (1C), 125.37 (1C), 127.73 (1C), 127.96 (1C), 128.142 (1C), 128.80 (1C), 136.20 (1C), 141.90 (1C), 143.11 (1C), 152.46 (1C), 159.10 (1C), 164.59 (1C).

HRMS: (ESI$^+$-MS, m/z) [M + Na]$^+$ calcd for [$C_{21}H_{21}N_5O_5$]: 446.15; found: 446.28.

Ethyl 6-(((1H-benzo[d][1,2,3]triazol-1-yl)oxy)methyl)-4-(3-methoxyphenyl)-2-oxo-1,2,3,4-tetrahydropyrimidine-5-carboxylate (11f)

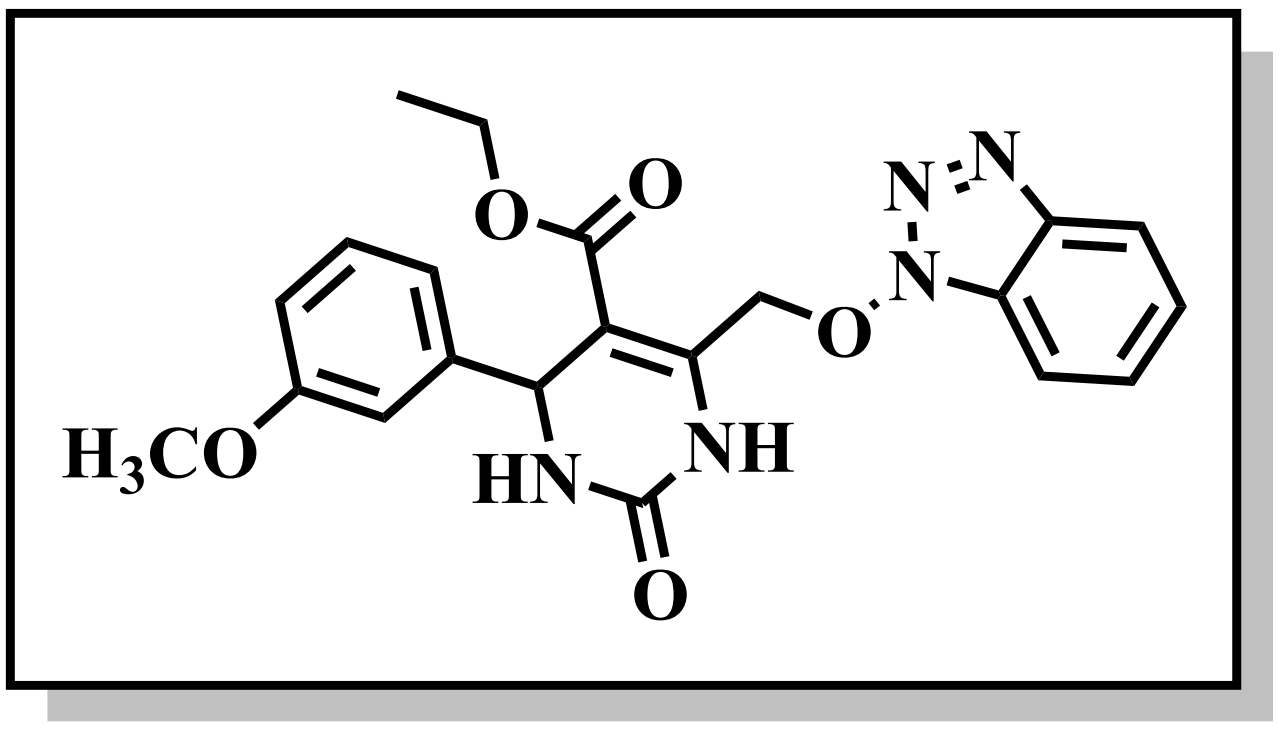

R*f* value: 0.13 (Hex/EtOAc, 40%)

Appearance: White solid

IR (APT): 1655, 1701, 2835, 2952, 3096, 3216 cm^{-1}.

^{1}H NMR (DMSO-d_6, 600 MHz): δ (ppm) 0.91 (t, *J* = 7.01 Hz, 3H), 3.71 (s, 3H), 3.75 (m, 2H), 5.09 (d, *J* = 2.71 Hz, 1H), 5.46 (d, *J* = 11.88 Hz, 1H), 5.74 (d, *J* = 11.88 Hz, 1H), 6.74 (m, 2H), 6.82 (m, 1H), 7.19 (t, *J* = 7.91 Hz, *J* = 15.81 Hz, 1H), 7.45 (t, *J* = 7.41 Hz, *J* = 15.25 Hz, 1H), 7.58 (t, *J* = 7.84 Hz, *J* = 15.25 Hz,1H), 7.75 (d, *J* = 8.28 Hz, 1H), 7.85 (s, 1H), 8.05 (d, *J* = 8.70 Hz, 1H), 9.82 (s, 1H).

^{13}C NMR (DMSO-d_6, 150 MHz): 14.13 (1C), 54.45 (1C), 55.45 (1C), 60.49 (1C), 75.22 (1C), 105.42 (1C), 109.65 (1C), 112.79 (1C), 113.08 (1C), 118.72 (1C), 120.08 (1C), 125.39 (1C), 127.69 (1C), 128.81 (1C), 130.19 (1C), 142.36 (1C), 143.10 (1C), 145.47 (1C), 152.54 (1C), 159.73 (1C), 164.57 (1C).

HRMS: (ESI$^+$-MS, m/z) [M + Na]$^+$ calcd for [$C_{21}H_{21}N_5O_5$]: 446.14; found: 446.14.

Ethyl 6-(((1H-benzo[d][1,2,3]triazol-1-yl)oxy)methyl)-4-(4-fluorophenyl)-2-oxo-1,2,3,4-tetrahydropyrimidine-5-carboxylate (11g)

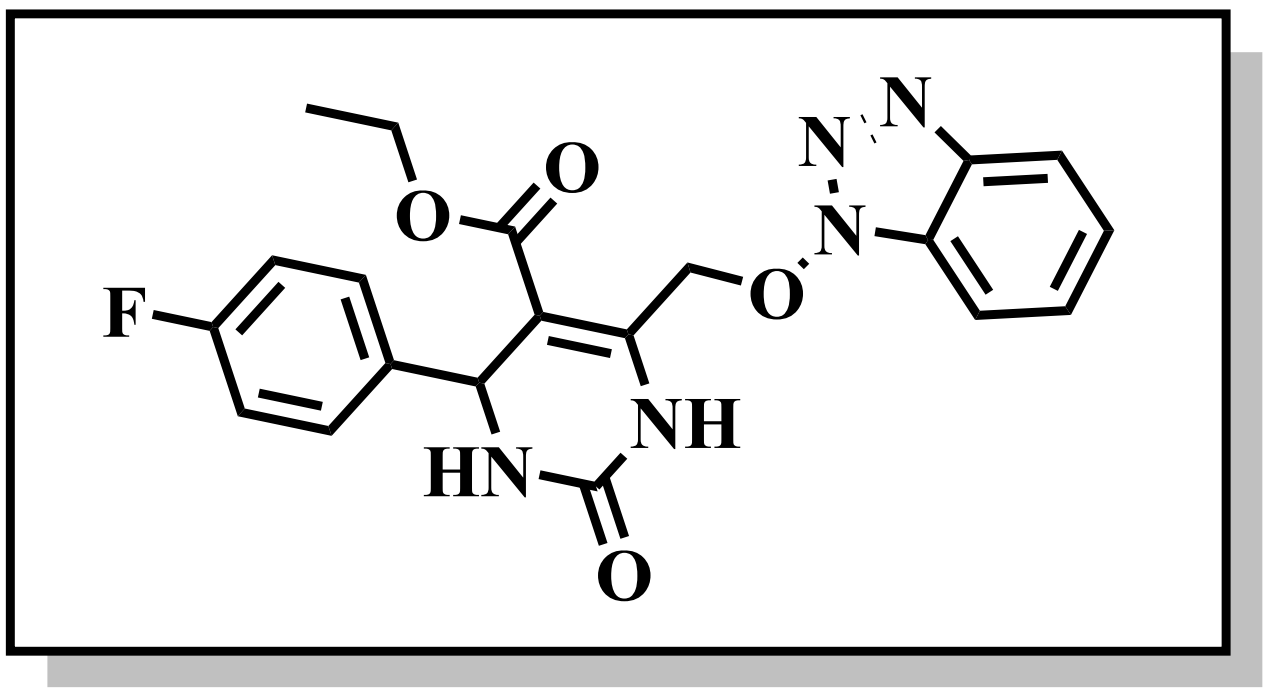

R*f* value: 0.15 (Hex/EtOAc, 40%)

Appearance: White solid

IR (APT): 1659, 1701, 2811, 2972, 3107, 3205 cm^{-1}.

^{1}H NMR (DMSO-d_6, 600 MHz): δ (ppm) 0.89 (t, J = 7.07 Hz, 3H), 3.75 (m, 2H), 5.11 (d, J = 3.08 Hz, 1H), 5.50 (d, J = 11.85 Hz, 1H), 5.72 (d, J = 11.85 Hz, 1H),7.09 (t, J = 8.89 Hz, 4H), 7.19 (m, 2H), 7.45 (t, J = 7.61 Hz, J = 15.23 Hz, 1H), 7.60 (t, J = 8.65 Hz, J = 15.19 Hz, 1H), 7.77 (d, J = 8.31 Hz, 1H), 7.78 (d, J = 2.33 Hz, 1H), 8.05 (d, J = 8.54 Hz, 1H), 9.87 (s, 1H).

^{13}C NMR (DMSO-d_6, 150 MHz): δ (ppm) 14.10 (1C), 54.01 (1C), 60.48 (1C), 75.16 (1C), 105.37 (1C), 109.68 (1C), 115.59 (J_{C-F} = 21 Hz, 2C), 120.10 (1C), 125.39 (1C), 127.70 (1C), 128.81 (J_{C-F} = 9.0 Hz, 2C), 140.31 (J_{C-F} = 4.5 Hz, 1C), 142.45 (1C), 143.10 (1C), 152.30 (1C), 161.10 (1C), 162.72 (1C), 164.46 (1C).

HRMS: (ESI^+-MS, m/z) $[M + Na]^+$ calcd for [$C_{20}H_{18}FN_5O_4$]: 434.12; found: 434.12.

Ethyl 6-(((1H-benzo[d][1,2,3]triazol-1-yl)oxy)methyl)-4-(3-fluorophenyl)-2-oxo-1,2,3,4-tetrahydropyrimidine-5-carboxylate (11h)

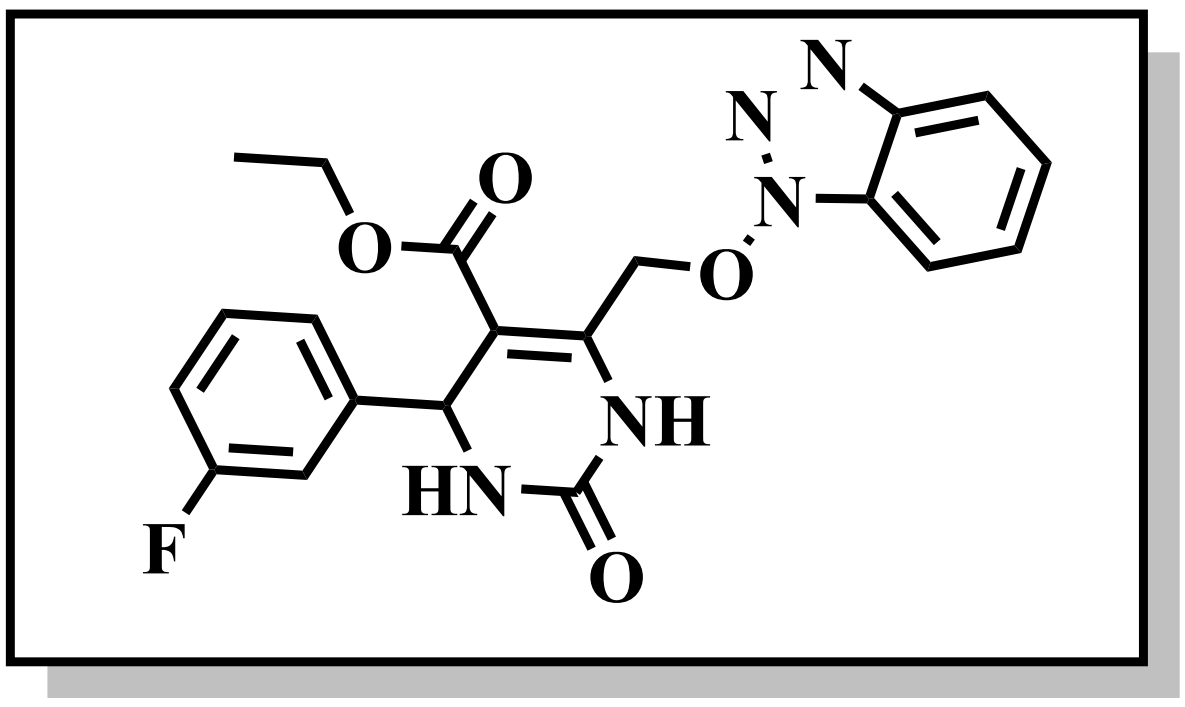

R*f* value: 0.17 (Hex/EtOAc, 40%)

Appearance: White solid

IR (APT): 1655, 1701, 2817, 2899, 3087, 3211 cm^{-1}.

^{1}H NMR (DMSO-d_6, 600 MHz): δ (ppm) 0.90 (t, *J* = 7.12 Hz, 3H), 3.76 (m, 2H), 5.14 (d, *J* = 3.10 Hz, 1H), 5.50 (d, *J* = 11.91 Hz, 1H), 5.73 (d, *J* = 11.91 Hz, 1H), 6.99-7.03 (m, 2H), 7.08-7.11 (m, 1H), 7.32 (q, *J* = 7.74 Hz, 1H), 7.45 (t, *J* = 7.59 Hz, *J* = 15.34 Hz, 1H), 7.59 (t, *J* = 7.76 Hz, *J* = 15.14 Hz, 1H), 7.77 (d, *J* = 8.24 Hz, 1H), 7.90 (d, *J* = 2.83 Hz, 1H), 8.05 (d, *J* = 8.58 Hz, 1H), 9.87 (s, 1H).

^{13}C NMR (DMSO-d_6, 150 MHz): δ (ppm) 14.09 (1C), 54.18 (1C), 60.52 (1C), 75.18 (1C), 104.78 (1C), 109.64 (1C), 113.53 (J_{C-F} = 21 Hz, 1C), 114.81 (J_{C-F} = 21 Hz, 1C), 120.11 (1C), 122.81 (1C), 125.39 (1C), 127.69 (1C), 128.81 (1C), 130.98 (1C), 131.04 (1C), 142.96 (1C), 143.11 (1C), 146.78 (1C), 152.31 (1C), 164.44 (1C).

HRMS: (ESI^+-MS, m/z) $[M + Na]^+$ calcd for [$C_{20}H_{18}FN_5O_4$]: 434.12; found: 434.12.

Ethyl 6-(((1H-benzo[d][1,2,3]triazol-1-yl)oxy)methyl)-4-(2,4-difluorophenyl)-2-oxo-1,2,3,4-tetrahydropyrimidine-5-carboxylate (11i)

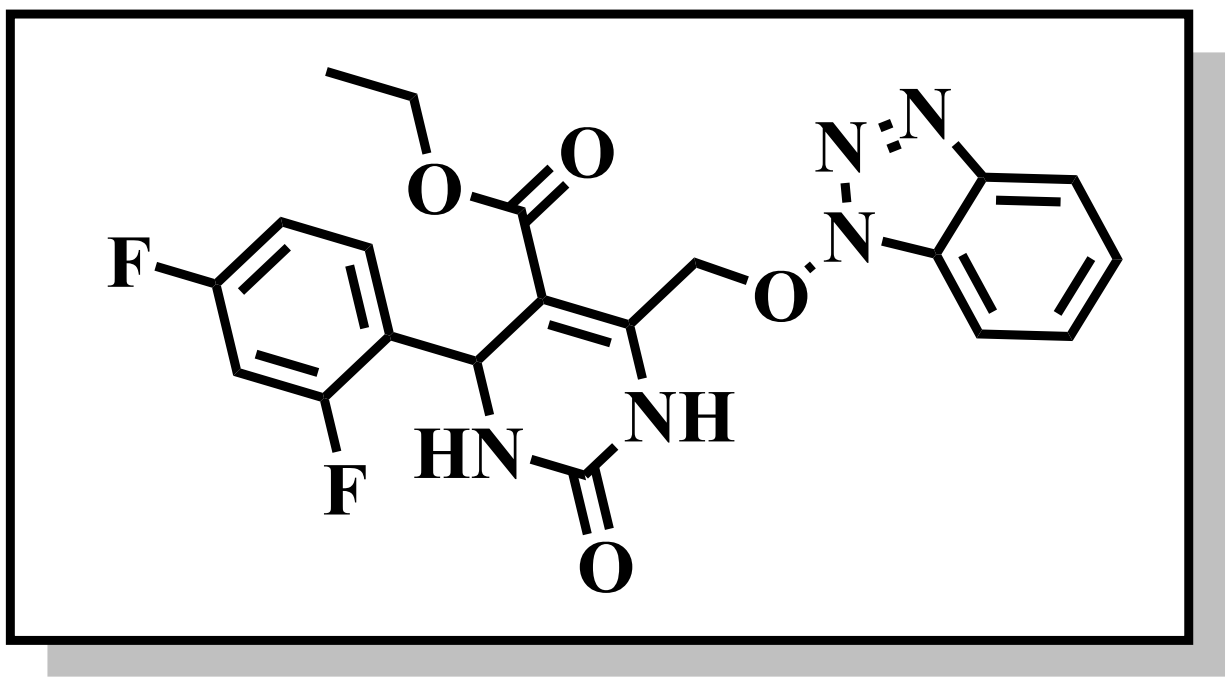

R*f* value: 0.20 (Hex/EtOAc, 40%)

Appearance: White solid

IR (APT): 1657, 1705, 2827, 2941, 3093, 3220 cm^{-1}.

^{1}H NMR (DMSO-d_6, 600 MHz): δ (ppm) 0.86 (t, J = 7.11 Hz, 3H), 3.71 (m, 2H), 5.38 (d, J = 2.74 Hz, 1H), 5.51 (d, J = 11.87 Hz, 1H), 5.74 (d, J = 11.87 Hz, 1H), 6.97-7.00 (m, 1H), 7.17-7.21 (m, 1H), 7.25 (dd, J = 8.57 Hz, J = 6.83 Hz, 1H), 7.46 (t, J = 7.58 Hz, J = 15.32 Hz, 1H), 7.62 (d, J = 7.74 Hz, J = 15.18 Hz, 1H), 7.83 (d, J = 8.34 Hz, 1H), 8.06 (d, J = 8.35 Hz, 1H), 9.91 (s, 1H).

^{13}C NMR (DMSO-d_6, 150 MHz): δ (ppm) 13.96 (1C), 48.79 (1C), 60.40 (1C), 75.09 (1C), 103.54 (J_{C-F} = 87.0 Hz, 1C), 104.30 (J_{C-F} = 25.5 Hz, 1C), 109.72 (1C), 112.04 (1C), 112.18 (1C), 120.12 (1C), 125.42 (1C), 127.65 (1C), 127.70 (1C), 128.85 (2C), 130.61 (1C), 143.11 (1C), 151.81 (1C), 164.14 (1C).

HRMS: (ESI^{+}-MS, m/z) $[M + Na]^{+}$ calcd for $[C_{20}H_{17}F_2N_5O_4]$: 452.11; found: 452.11.

Ethyl 6-(((1H-benzo[d][1,2,3]triazol-1-yl)oxy)methyl)-4-(3-chlorophenyl)-2-oxo-1,2,3,4-tetrahydropyrimidine-5-carboxylate (11j)

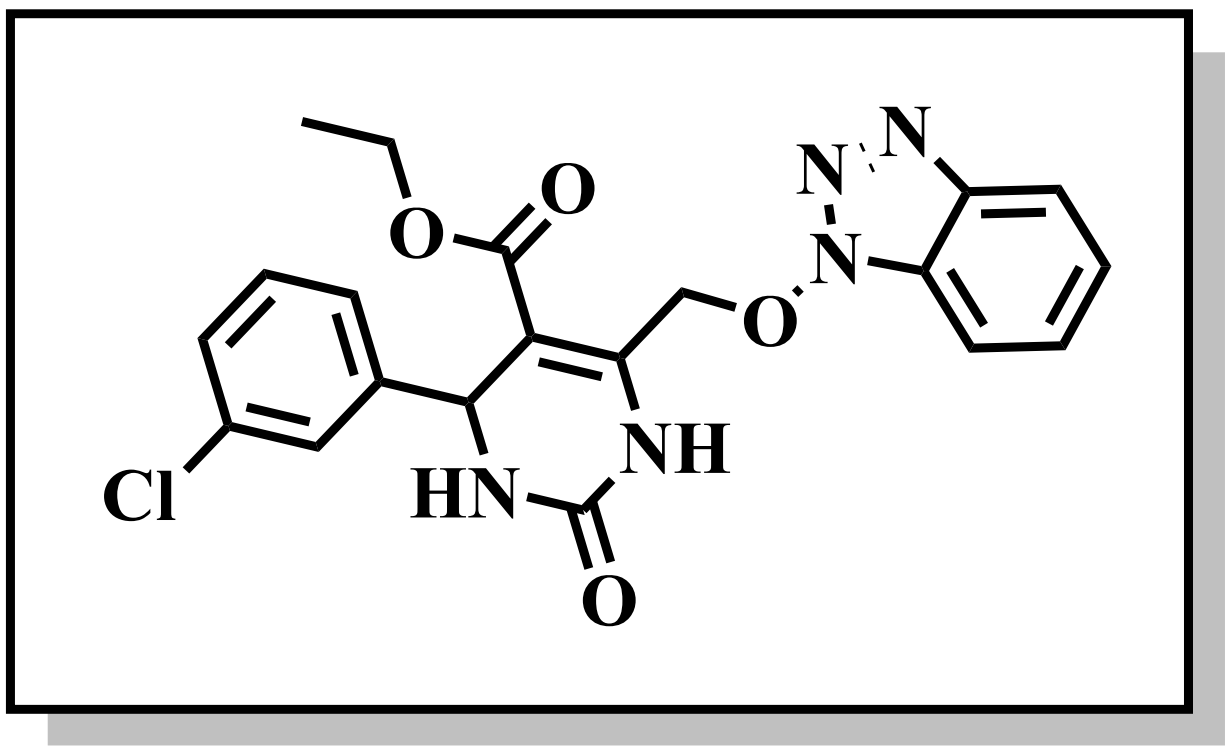

R*f* value: 0.20 (Hex/EtOAc, 40%)

Appearance: White solid

IR (APT): 1656, 1702, 2817, 2917, 3096, 3227 cm^{-1}.

^{1}H NMR (DMSO-d_6, 600 MHz): δ (ppm) 0.90 (t, *J* = 7.12 Hz, 3H), 3.75 (m, 2H), 5.13 (d, *J* = 3.09 Hz, 1H), 5.49 (d, *J* = 11.88 Hz, 1H), 5.73 (d, *J* = 11.88 Hz, 1H), 7.13 (m, 1H), 7.27 (s, 1H), 7.32 (m, 2H), 7.45 (t, *J* = 7.47 Hz, *J* = 15.21 Hz, 1H), 7.59 (t, *J* = 7.69 Hz, *J* = 15.27 Hz, 1H), 7.77 (d, *J* = 8.39 Hz,1H), 7.91 (s, 1H), 8.05 (d, *J* = 8.39 Hz, 1H), 9.90 (s, 1H).

^{13}C NMR (DMSO-d_6, 150 MHz): 14.08 (1C), 60.54 (1C), 75.17 (1C), 104.69 (1C), 109.62 (1C), 120.11 (1C), 125.40 (1C), 125.45 (1C), 126.84 (1C), 127.68 (1C), 128.06 (1C), 128.82 (1C), 130.97 (1C), 133.51 (1C), 143.01 (1C), 143.10 (1C), 146.38 (1C), 152.26 (1C), 164.40 (1C).

HRMS: (ESI^+-MS, m/z) $[M + Na]^+$ calcd for [$C_{20}H_{18}ClN_5O_4$]: 450.09; found: 450.09.

Spectral data of compound 10a-j

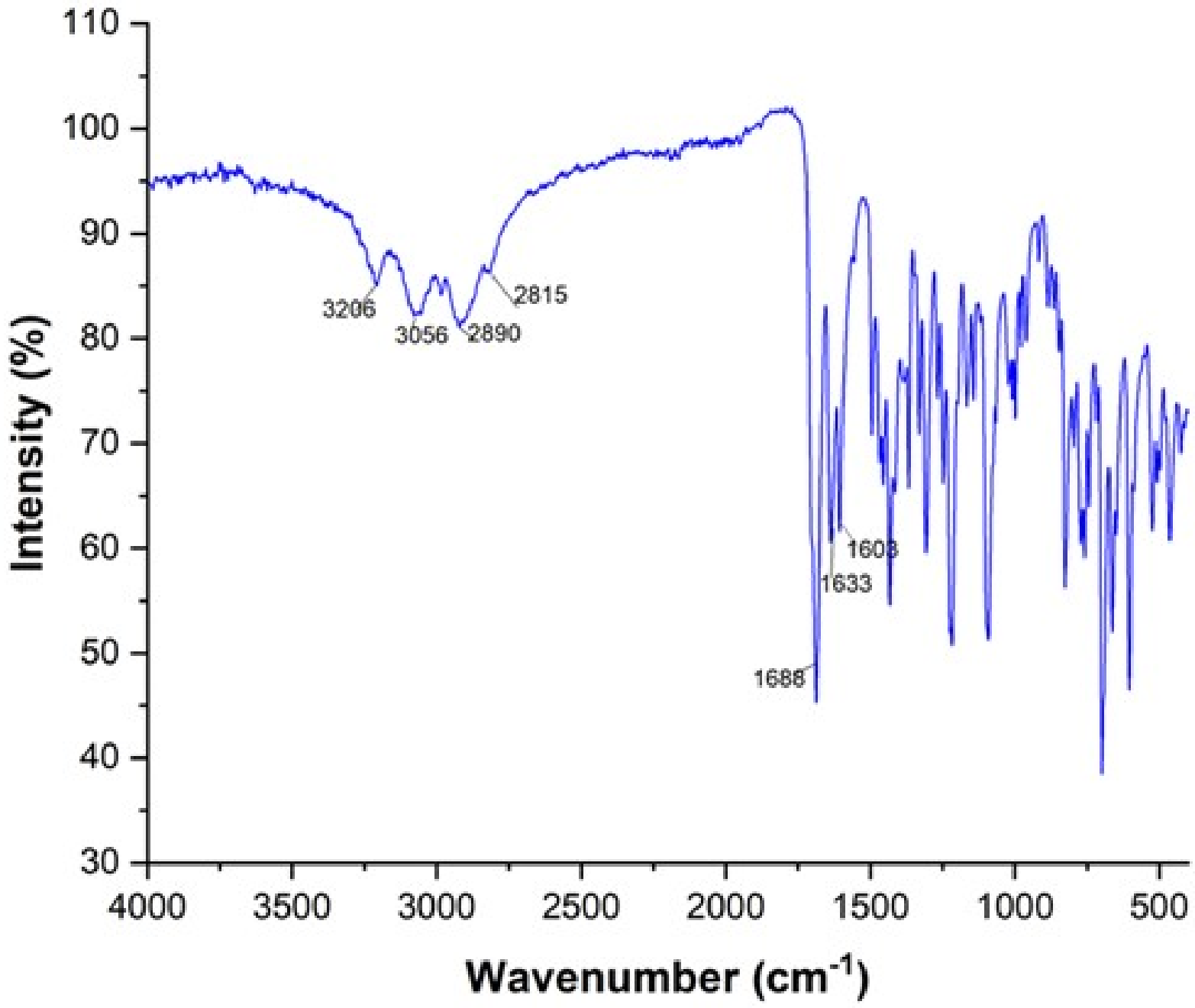

IR spectra of compound **10a**.

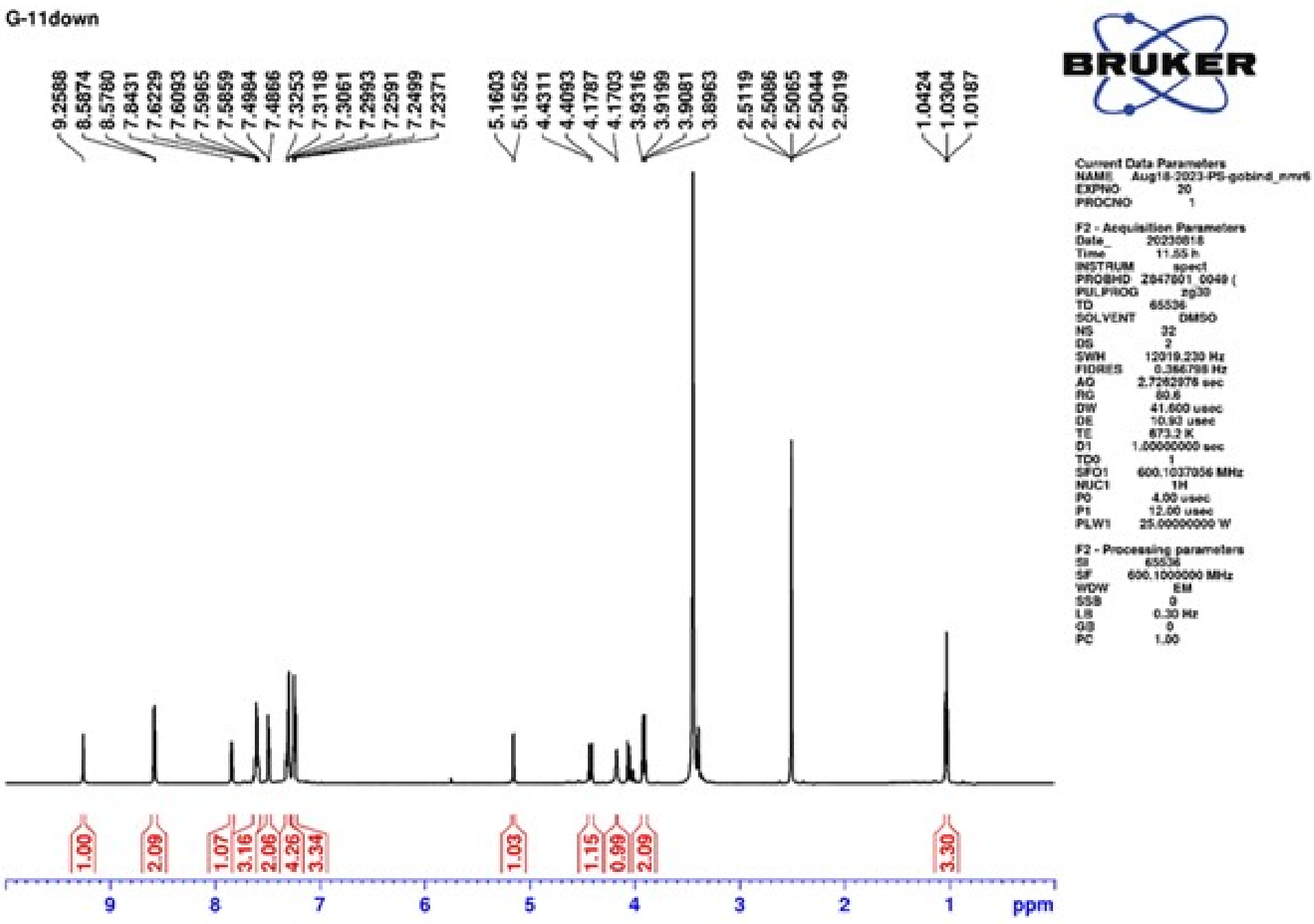

^{1}H NMR of Compound **10a**.

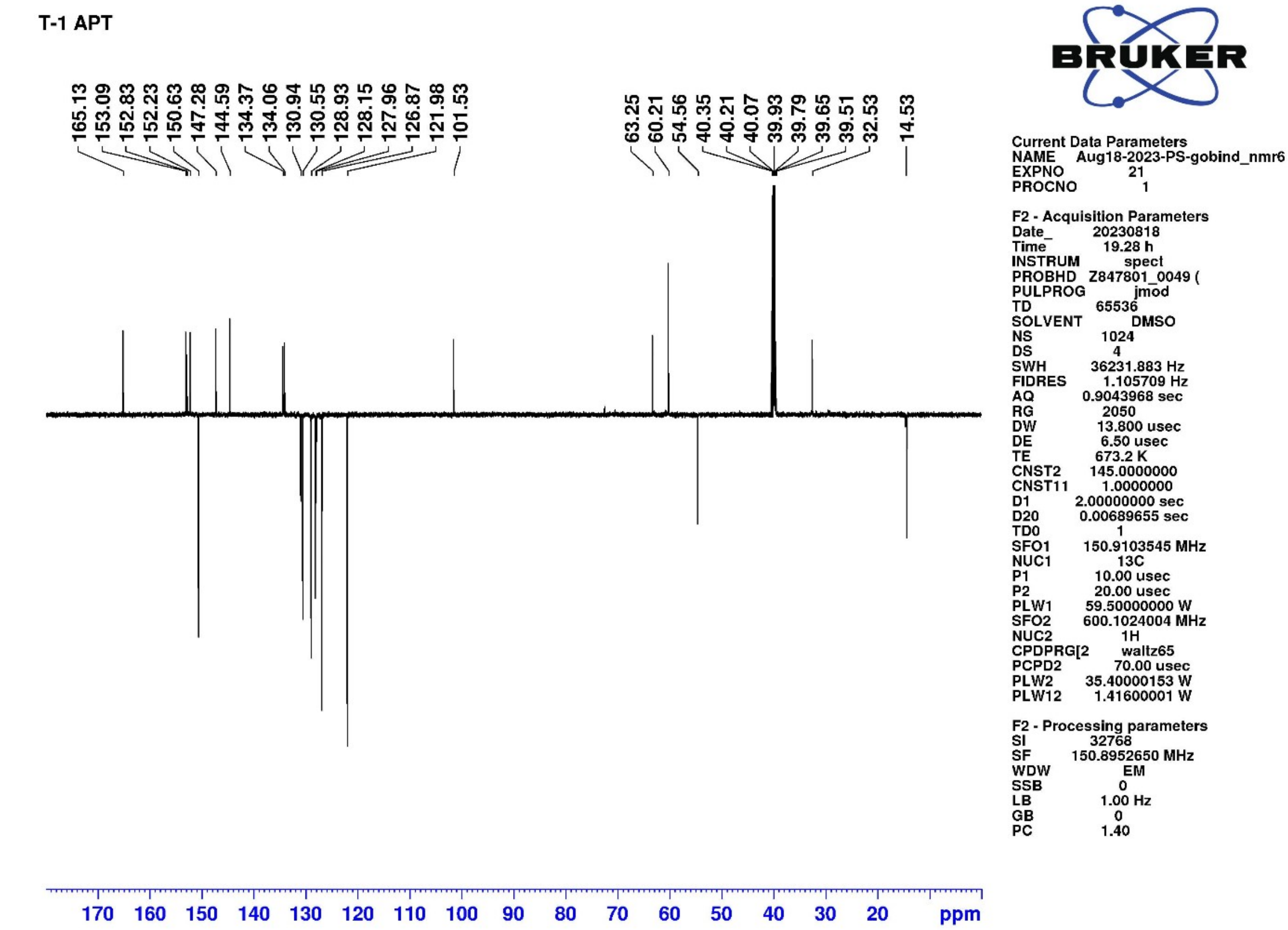

^{13}C NMR APT of Compound **10a**.

Single Mass Analysis
Tolerance = 5.0 PPM / DBE: min = -1.5, max = 50.0
Element prediction: Off
Number of isotope peaks used for i-FIT = 3

Monoisotopic Mass, Even Electron Ions
242 formula(e) evaluated with 1 results within limits (up to 50 closest results for each mass)
Elements Used:
C: 10-50 H: 10-100 N: 1-10 O: 1-5 Na: 1-1 S: 1-1
T-1 1 (0.024) Cm (1:230)
TOF MS ES+

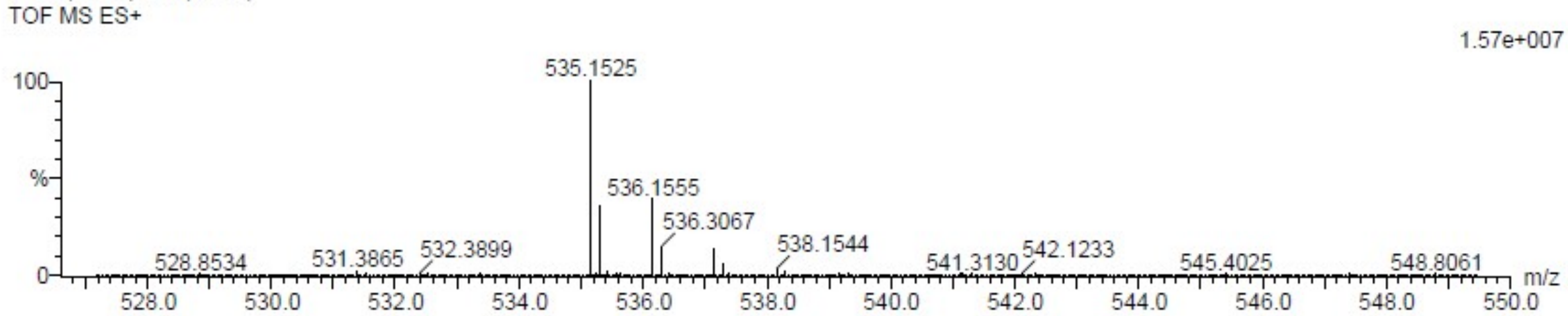

Minimum:				-1.5				
Maximum:		2.0	5.0	50.0				
Mass	Calc. Mass	mDa	PPM	DBE	i-FIT	Norm	Conf(%)	Formula
535.1525	535.1528	-0.3	-0.6	18.5	1260.8	n/a	n/a	C27 H24 N6 O3 Na S

HRMS of Compound **10a**.

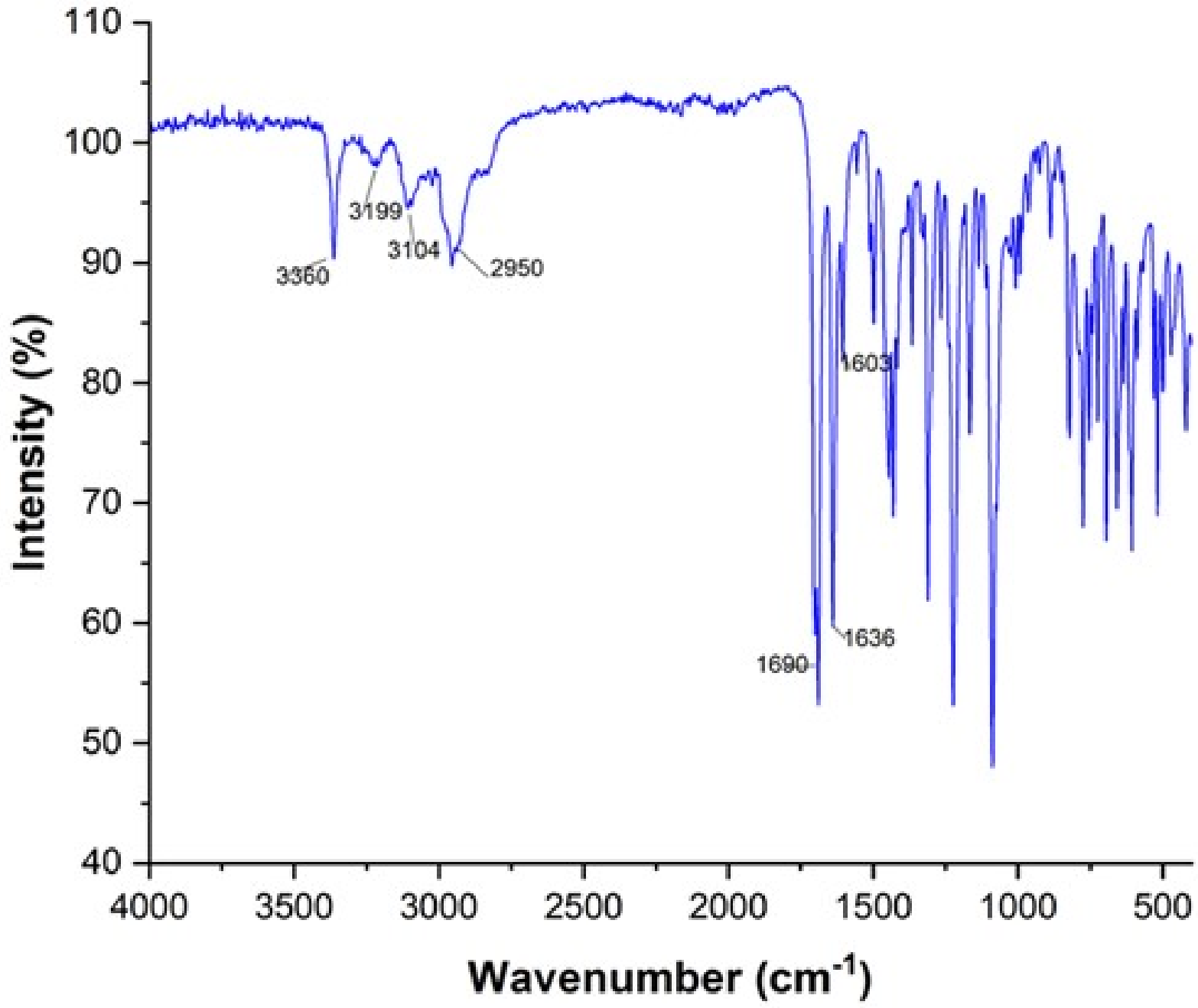

IR spectra of compound **10b**.

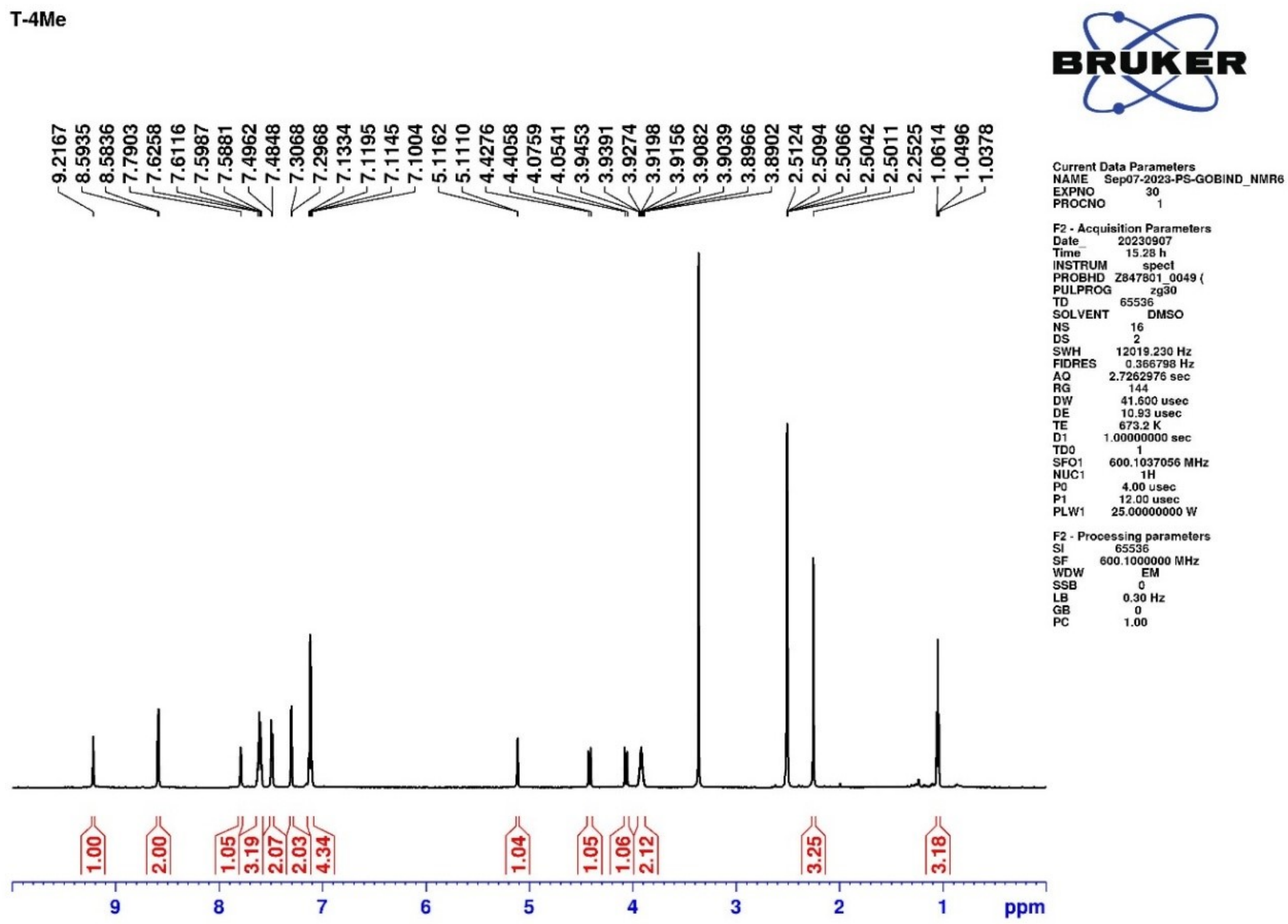

^{1}H NMR of Compound **10b**.

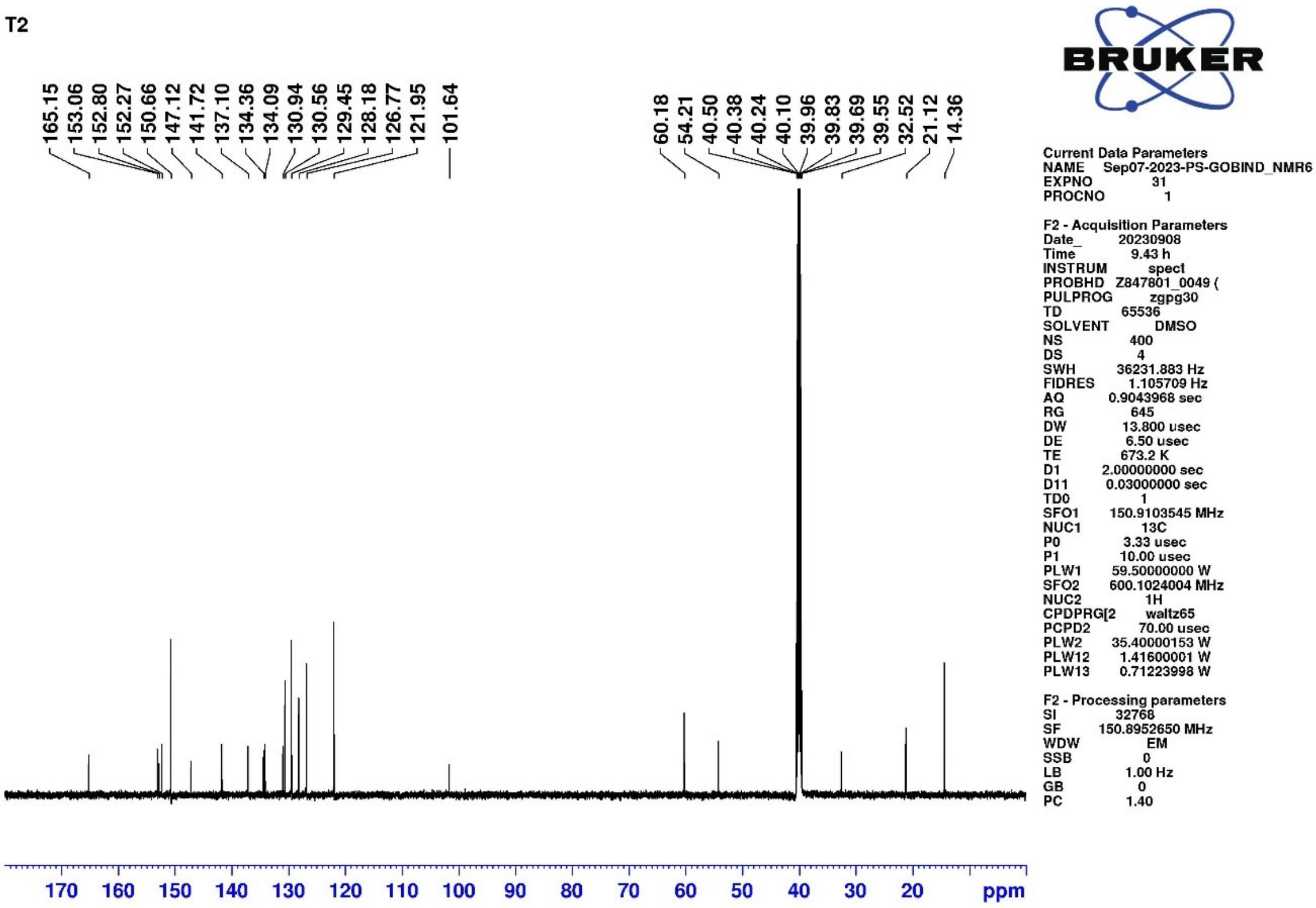

^{13}C NMR of Compound **10b**.

Single Mass Analysis
Tolerance = 5.0 PPM / DBE: min = -1.5, max = 50.0
Element prediction: Off
Number of isotope peaks used for i-FIT = 3

Monoisotopic Mass, Even Electron Ions
250 formula(e) evaluated with 2 results within limits (up to 50 closest results for each mass)
Elements Used:
C: 10-50 H: 10-100 N: 1-10 O: 1-5 Na: 1-1 S: 1-1
T-2 201 (1.739) Cm (1:230)
TOF MS ES+

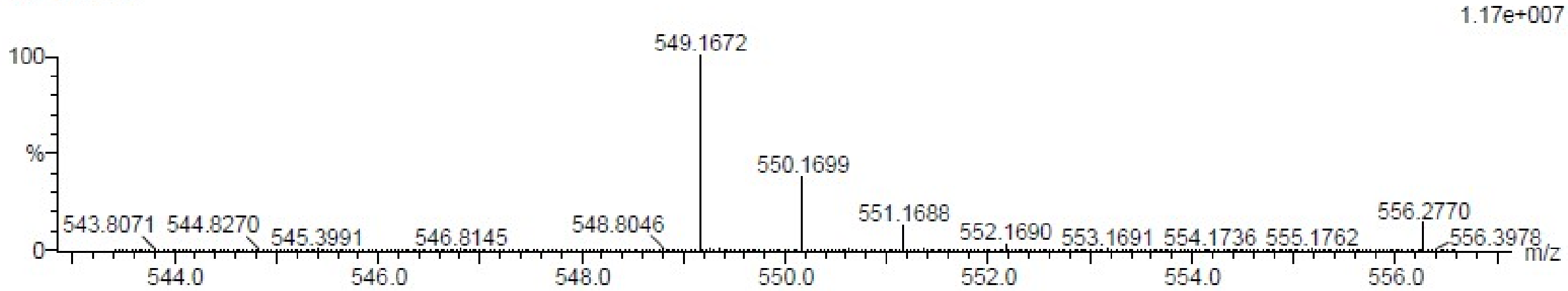

Minimum:				-1.5				
Maximum:		2.0	5.0	50.0				
Mass	Calc. Mass	mDa	PPM	DBE	i-FIT	Norm	Conf(%)	Formula
549.1672	549.1685	-1.3	-2.4	18.5	1091.8	0.003	99.73	C28 H26 N6 O3 Na S
	549.1645	2.7	4.9	14.5	1097.7	5.905	0.27	C23 H26 N8 O5 Na S

HRMS of Compound **10b**.

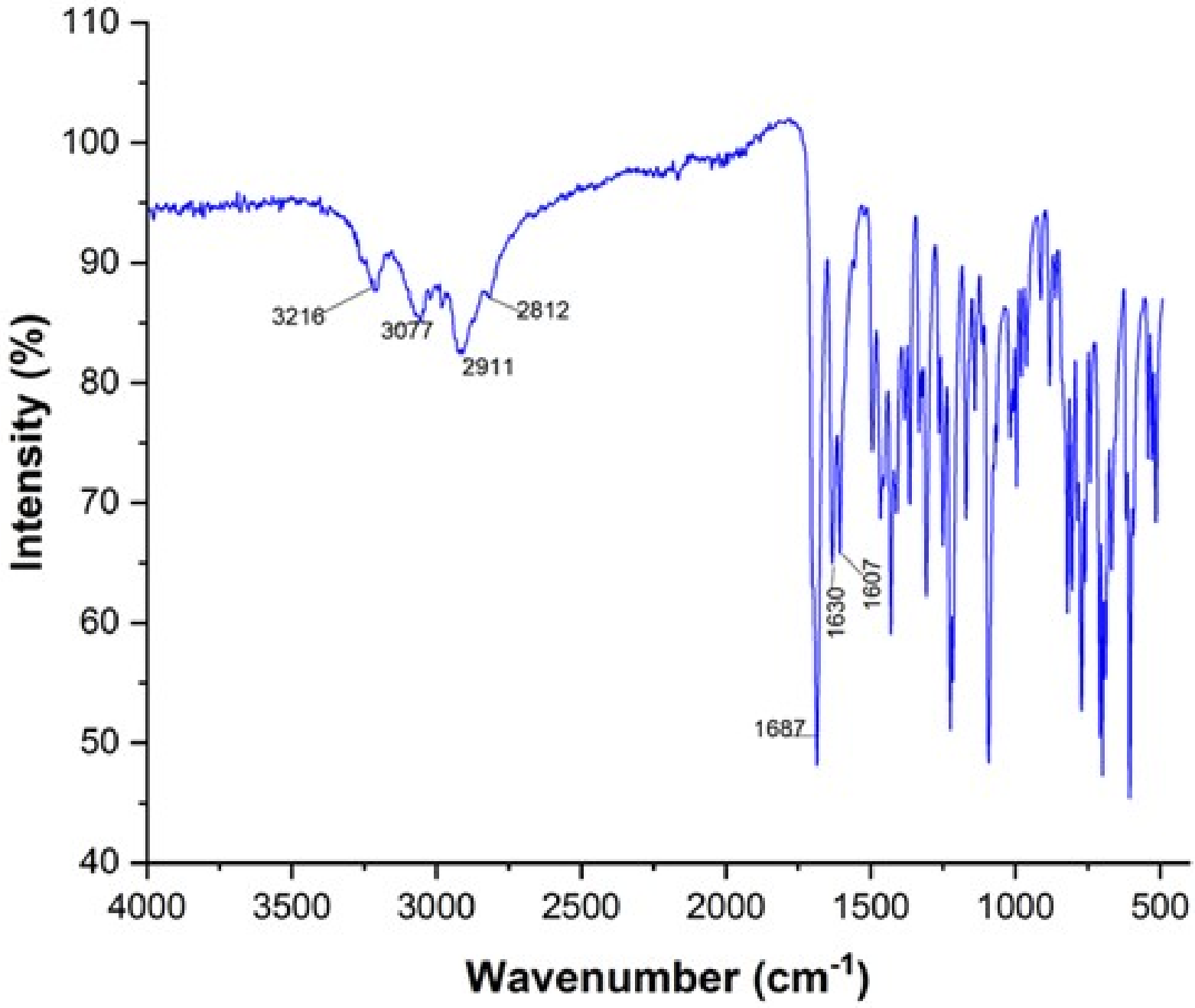

IR spectra of compound **10c**.

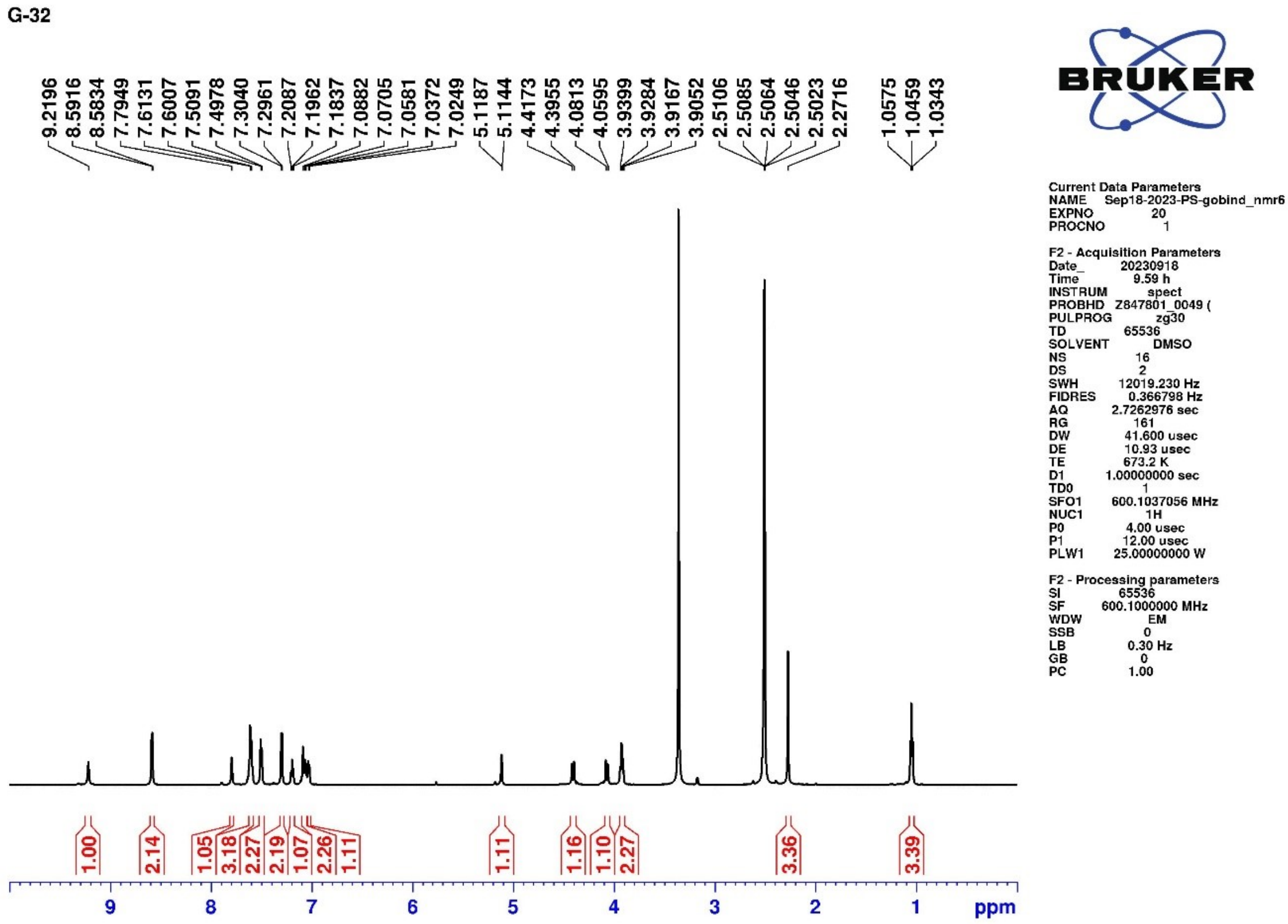

^{1}H NMR of Compound **10c**.

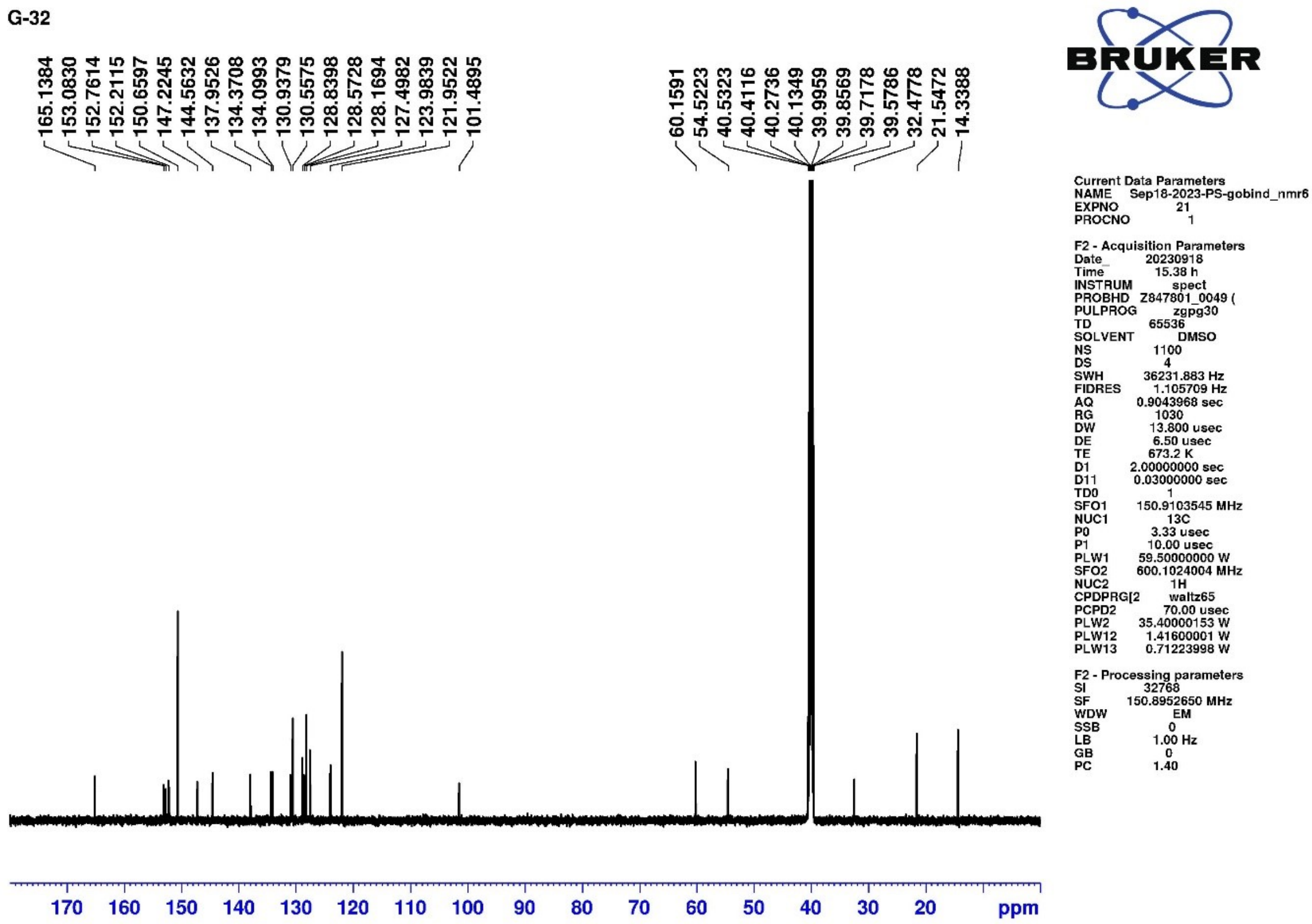

^{13}C NMR of Compound **10c**.

Single Mass Analysis
Tolerance = 5.0 PPM / DBE: min = -1.5, max = 50.0
Element prediction: Off
Number of isotope peaks used for i-FIT = 3

Monoisotopic Mass, Even Electron Ions
250 formula(e) evaluated with 2 results within limits (up to 50 closest results for each mass)
Elements Used:
C: 10-50 H: 10-100 N: 1-10 O: 1-5 Na: 1-1 S: 1-1
T-6 163 (1.413) Cm (1:230)
TOF MS ES+

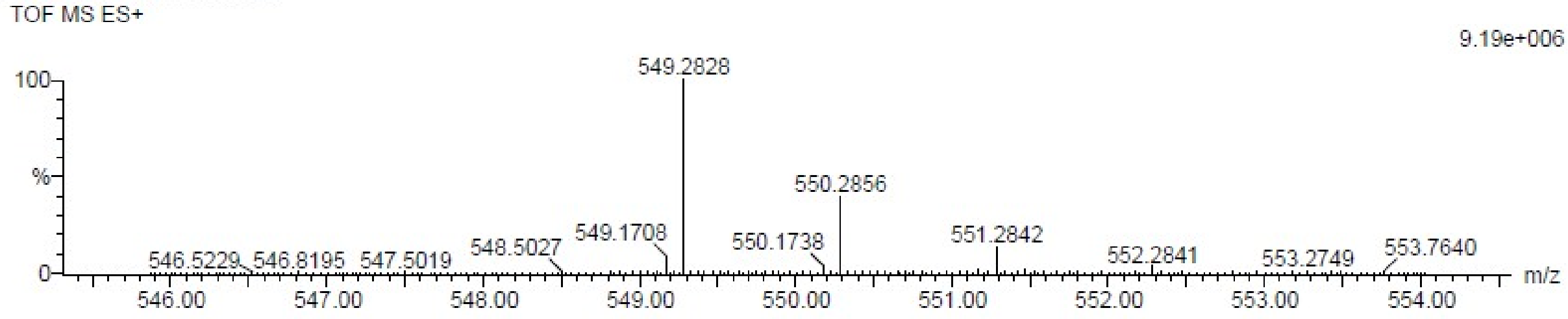

Minimum:				-1.5				
Maximum:		2.0	5.0	50.0				
Mass	Calc. Mass	mDa	PPM	DBE	i-FIT	Norm	Conf(%)	Formula
549.1708	549.1725	-1.7	-3.1	22.5	1262.1	0.316	72.93	C33 H26 N4 O Na S
	549.1685	2.3	4.2	18.5	1263.1	1.307	27.07	C28 H26 N6 O3 Na S

HRMS of Compound **10c**.

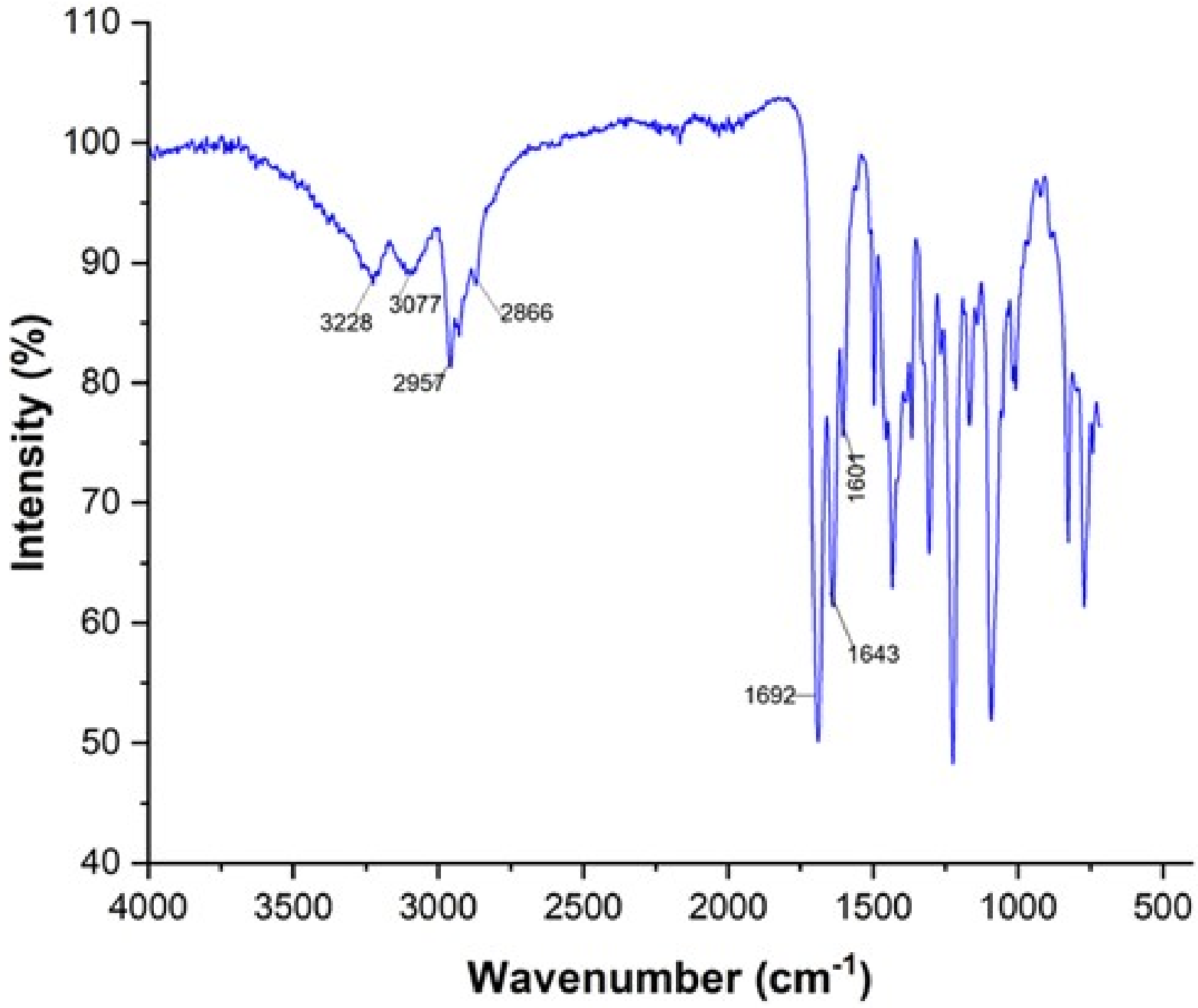

IR spectra of compound **10d**.

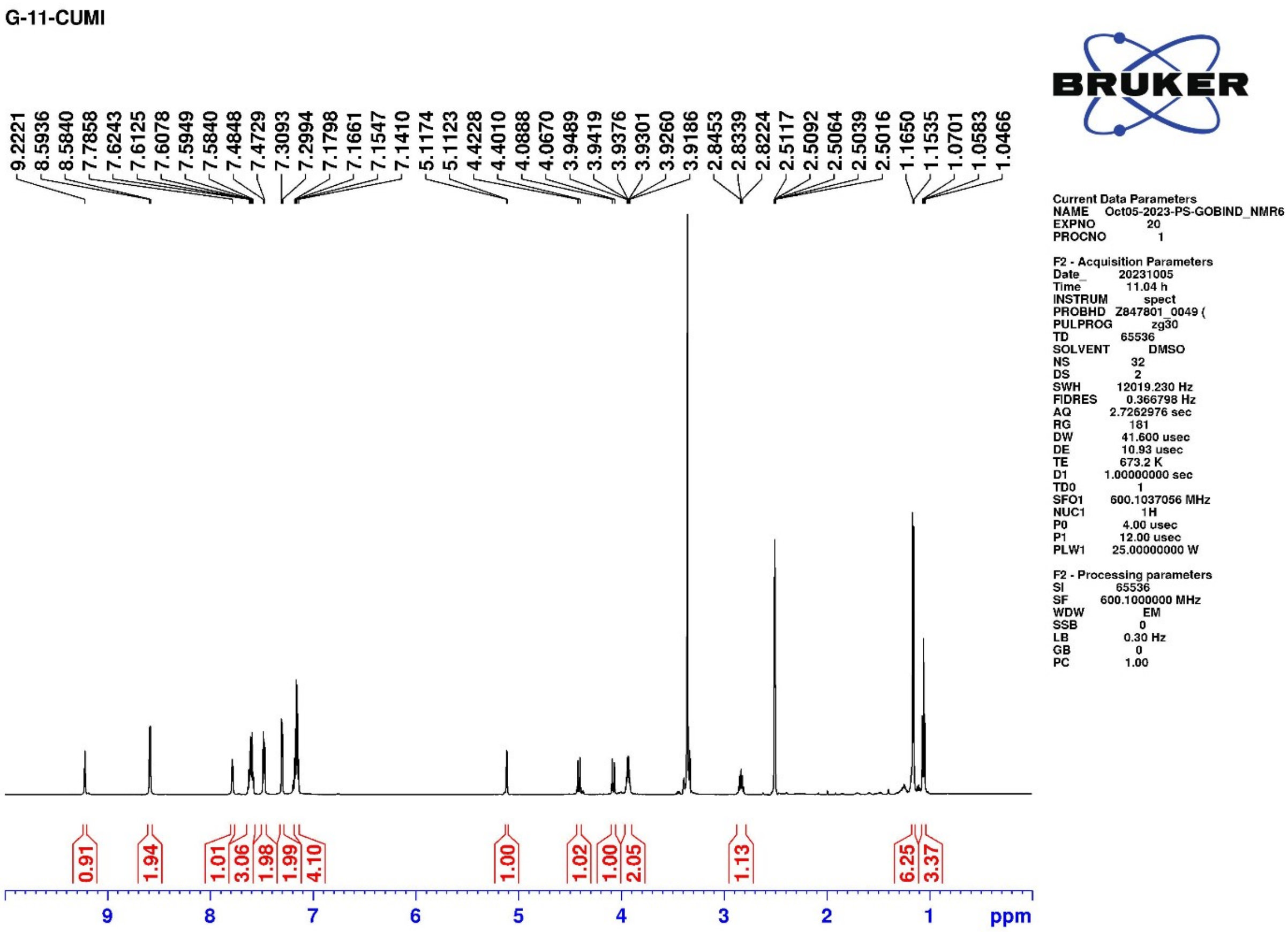

^{1}H NMR of Compound **10d**.

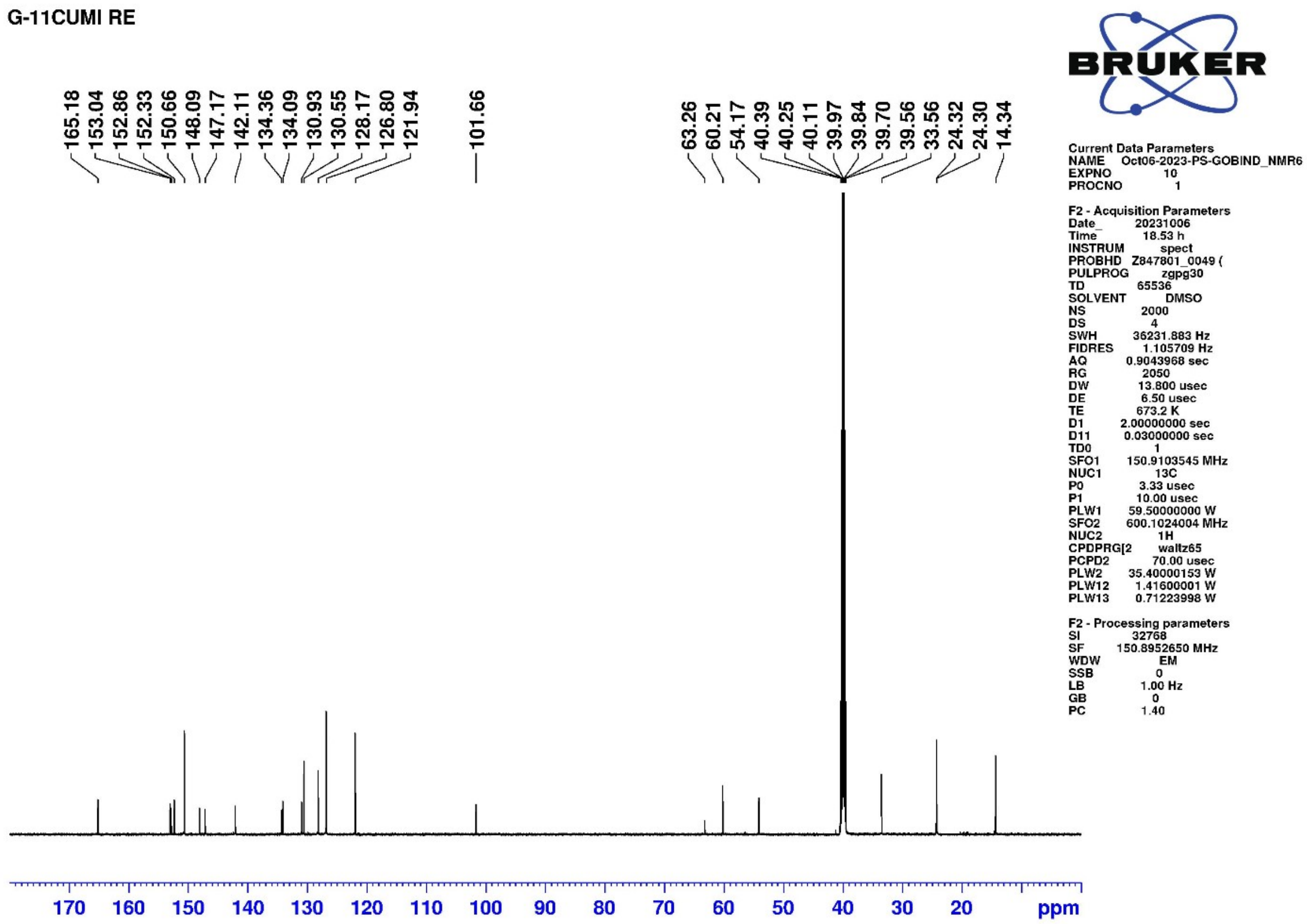

^{13}C NMR of Compound **10d**.

Single Mass Analysis
Tolerance = 5.0 PPM / DBE: min = -1.5, max = 50.0
Element prediction: Off
Number of isotope peaks used for i-FIT = 3

Monoisotopic Mass, Even Electron Ions
266 formula(e) evaluated with 2 results within limits (up to 50 closest results for each mass)
Elements Used:
C: 10-50 H: 10-100 N: 1-10 O: 1-5 Na: 1-1 S: 1-1
T-10 81 (0.710) Cm (1:230)
TOF MS ES+

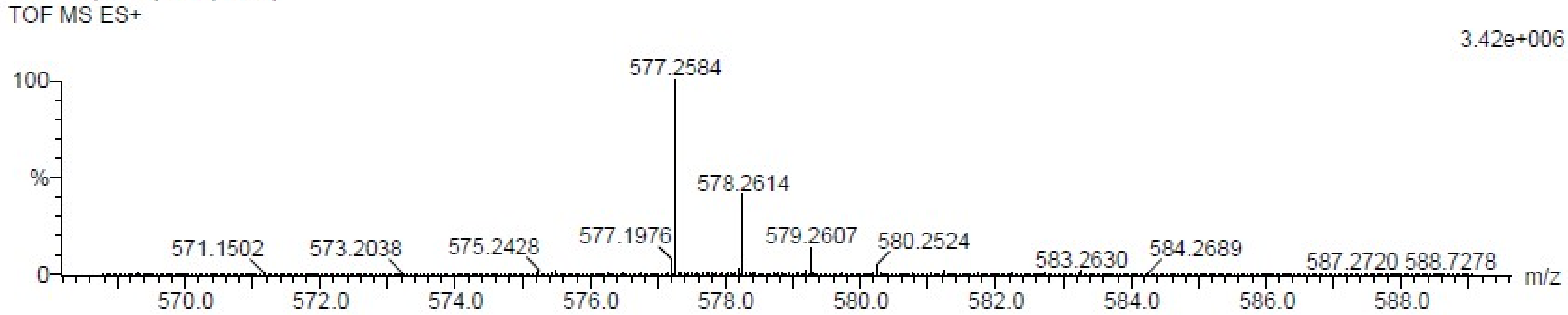

Minimum:				-1.5				
Maximum:		2.0	5.0	50.0				
Mass	Calc. Mass	mDa	PPM	DBE	i-FIT	Norm	Conf(%)	Formula
577.1976	577.1958	1.8	3.1	14.5	1101.3	3.026	4.85	C25 H30 N8 O5 Na S
	577.1998	-2.2	-3.8	18.5	1098.4	0.050	95.15	C30 H30 N6 O3 Na S

HRMS of Compound **10d**.

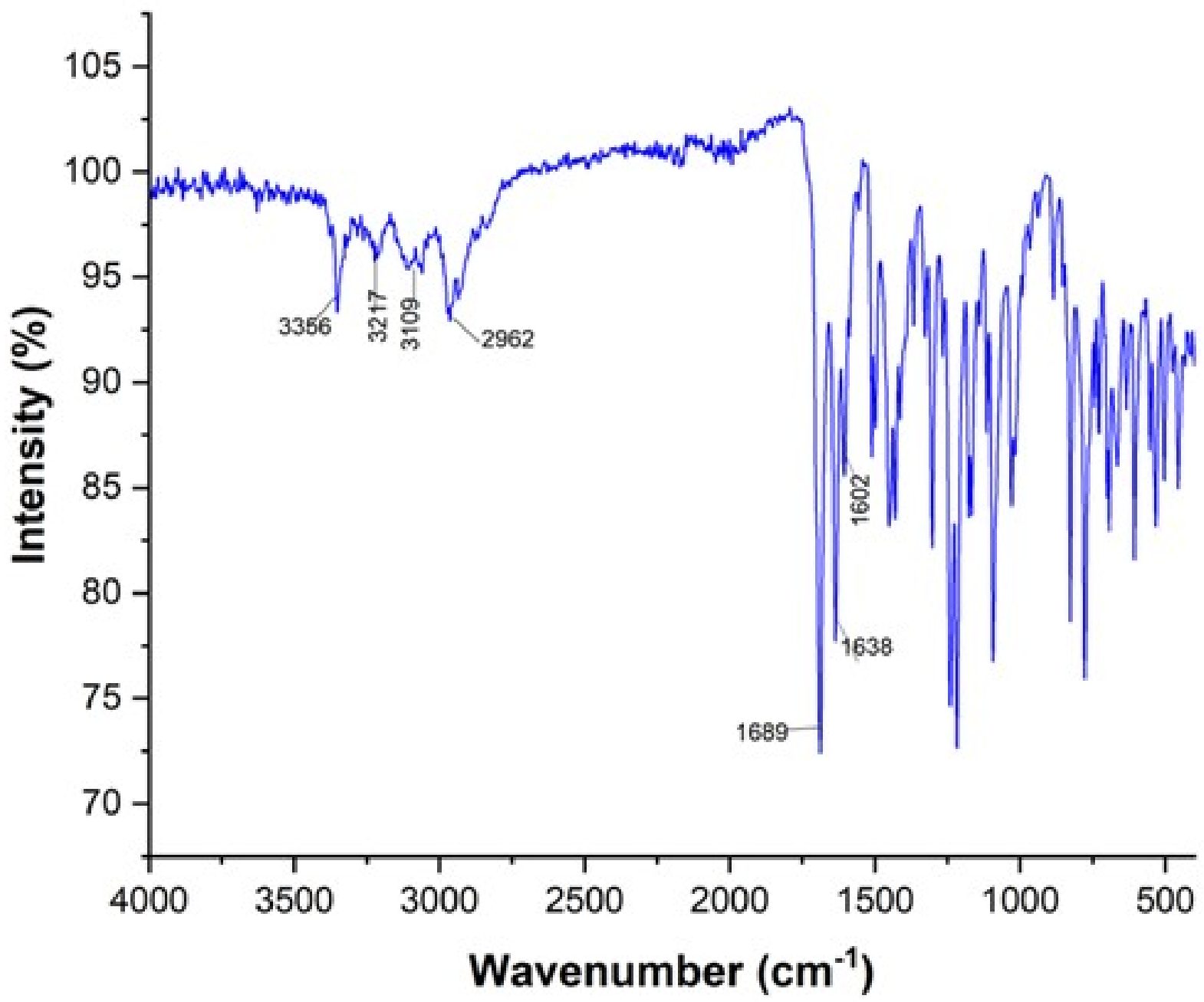

IR spectra of compound **10e**.

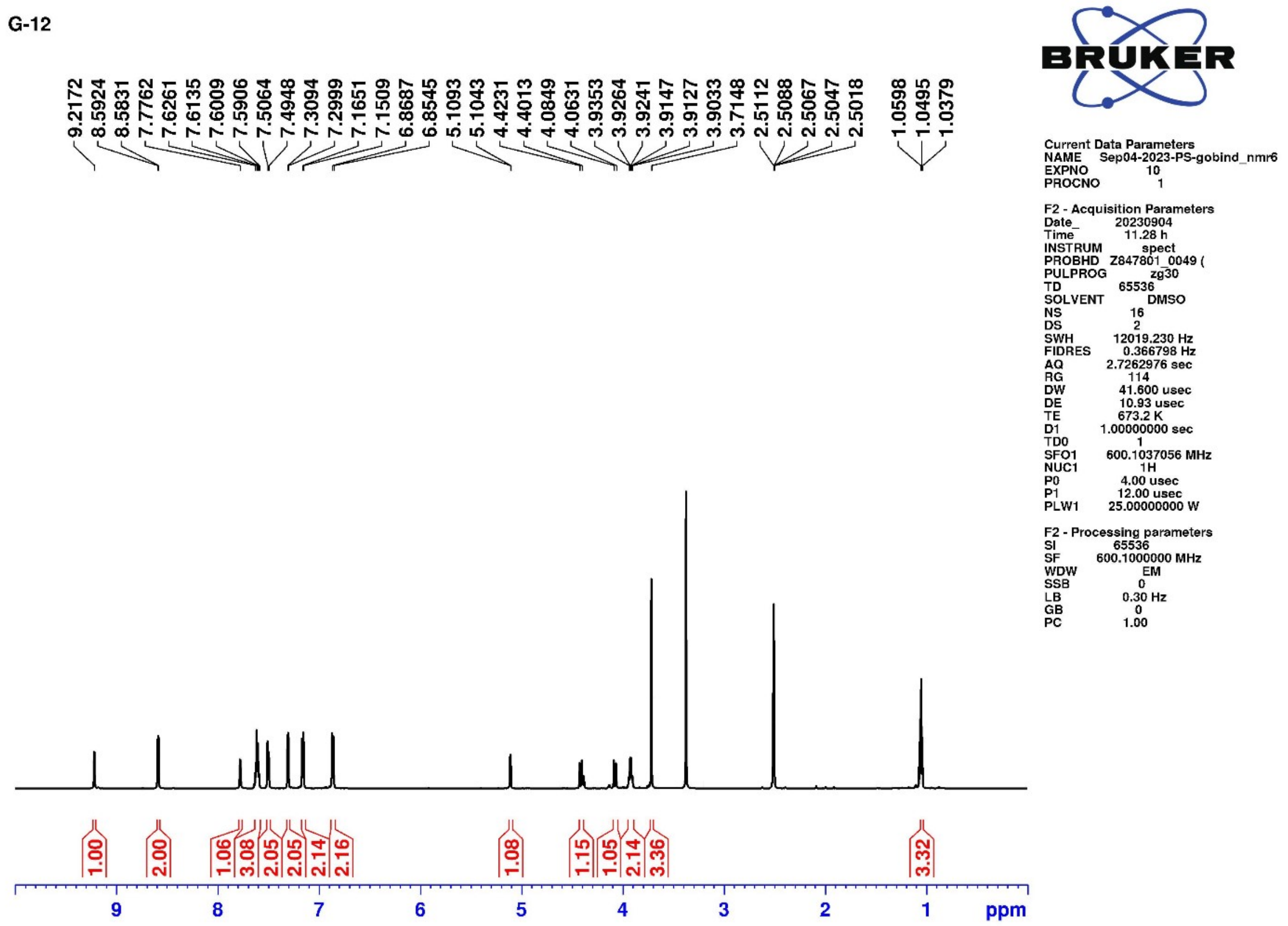

^{1}H NMR of Compound **10e**.

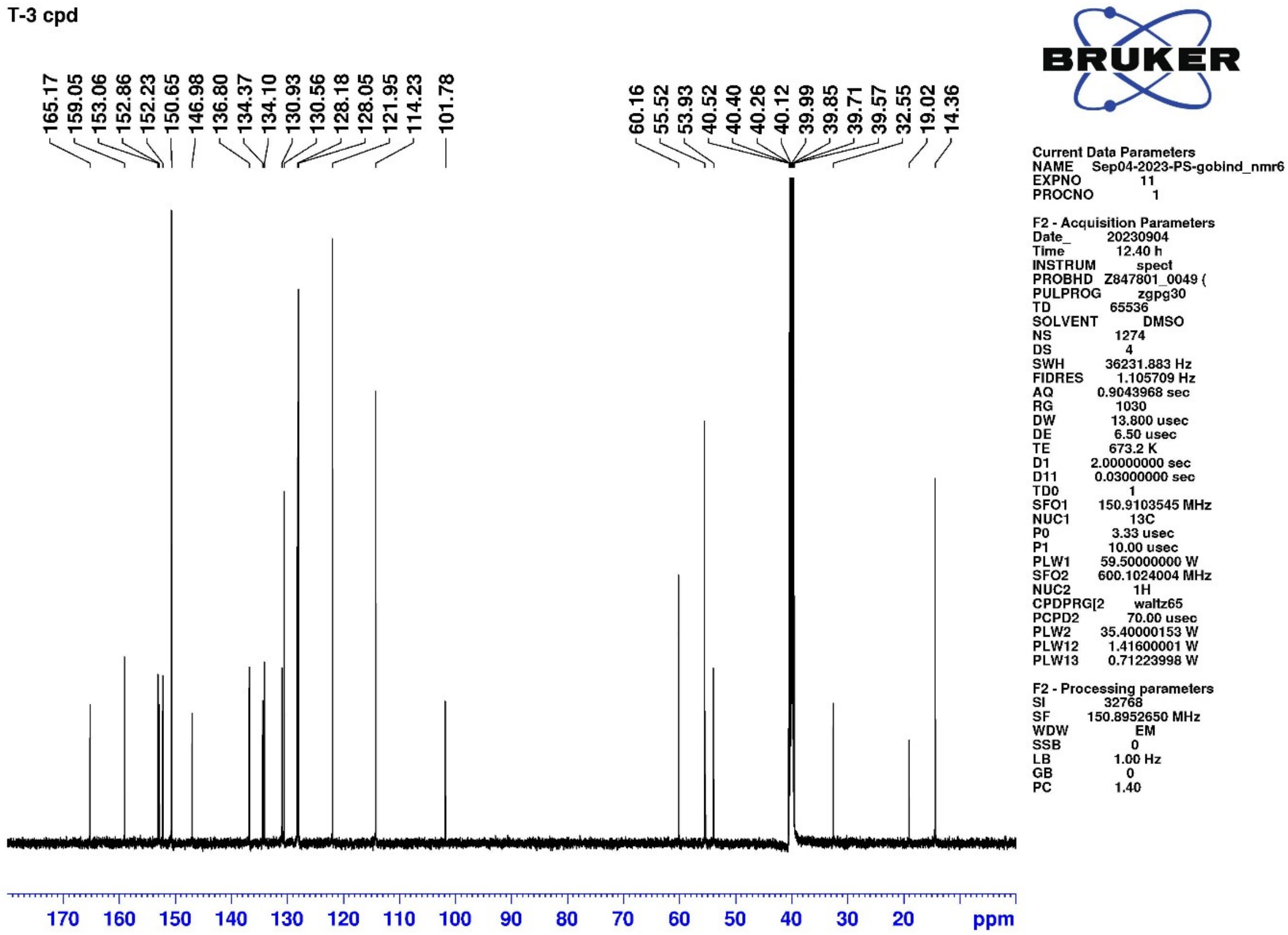

^{13}C NMR of Compound **10e**.

Single Mass Analysis
Tolerance = 5.0 PPM / DBE: min = -1.5, max = 50.0
Element prediction: Off
Number of isotope peaks used for i-FIT = 3

Monoisotopic Mass, Even Electron Ions
260 formula(e) evaluated with 1 results within limits (up to 50 closest results for each mass)
Elements Used:
C: 10-50 H: 10-100 N: 1-10 O: 1-5 Na: 1-1 S: 1-1
T-3 200 (1.730) Cm (1:230)
TOF MS ES+

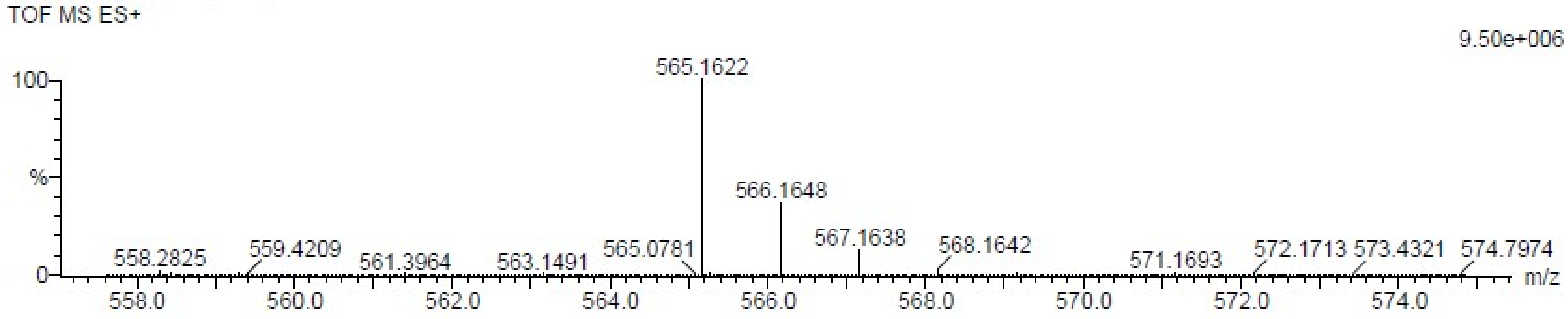

Minimum:				-1.5				
Maximum:		2.0	5.0	50.0				
Mass	Calc. Mass	mDa	PPM	DBE	i-FIT	Norm	Conf(%)	Formula
565.1622	565.1634	-1.2	-2.1	18.5	1050.9	n/a	n/a	C28 H26 N6 O4 Na S

HRMS of Compound **10e**.

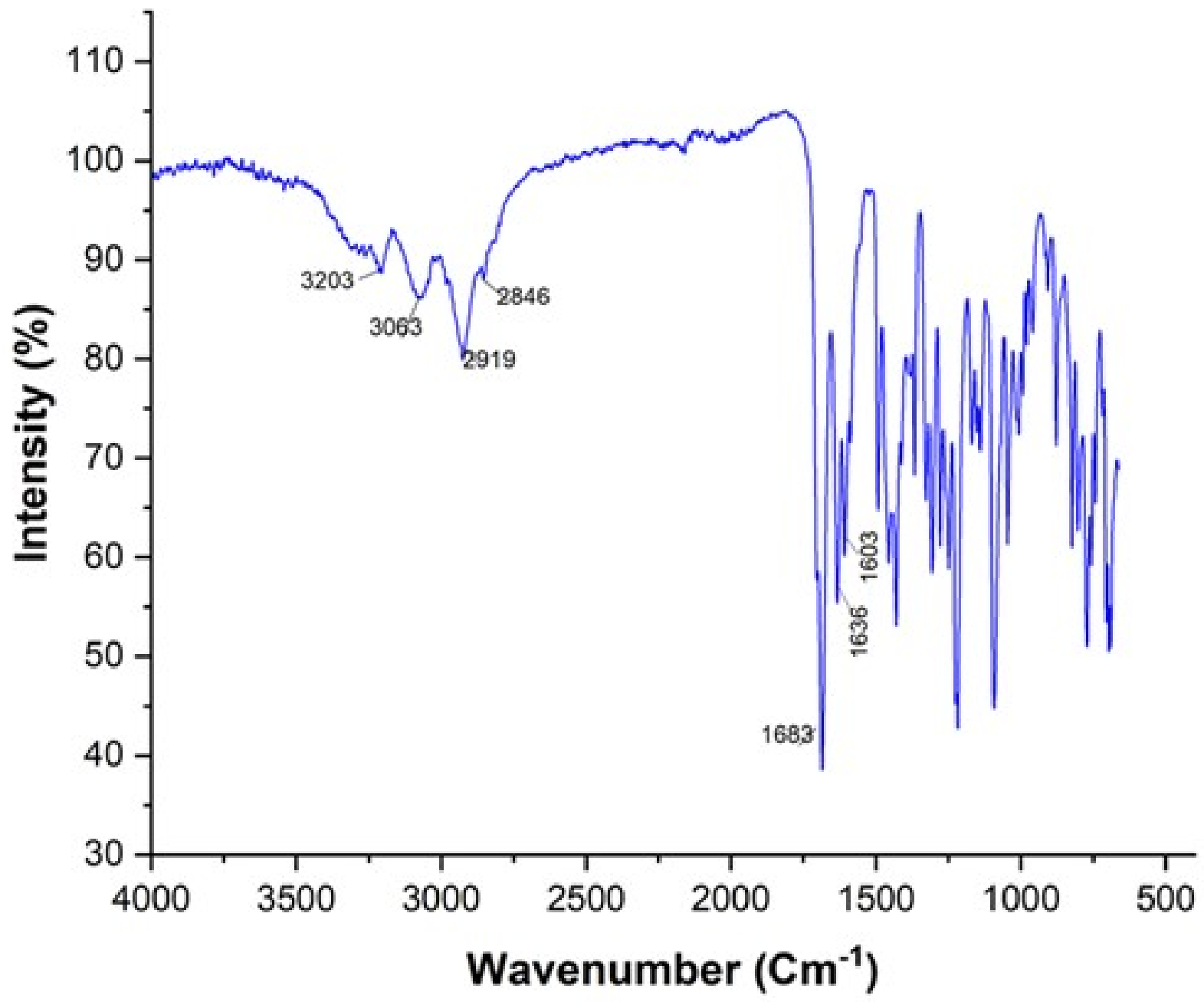

IR spectra of compound **10f**.

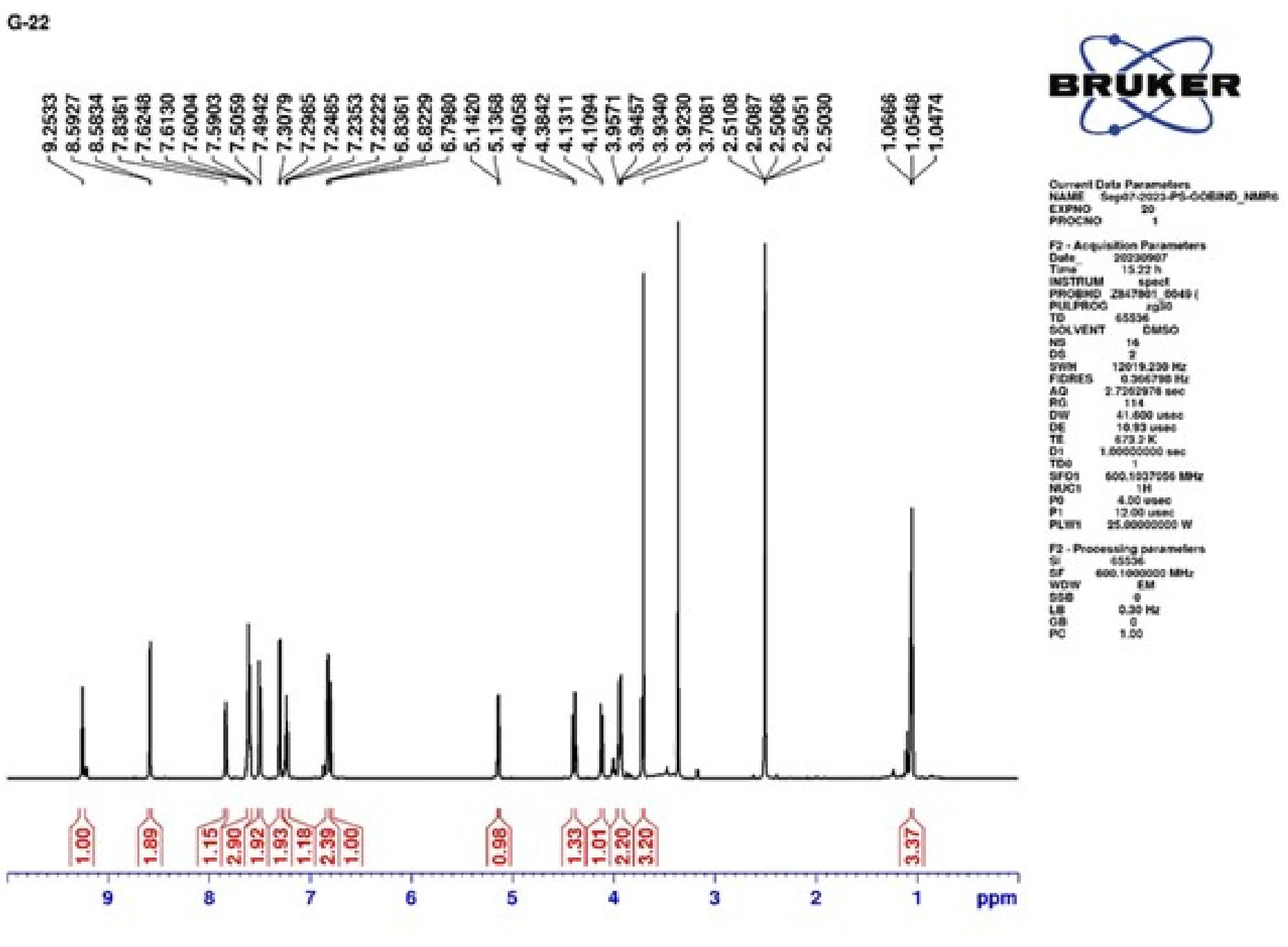

^{1}H NMR of Compound **10f**.

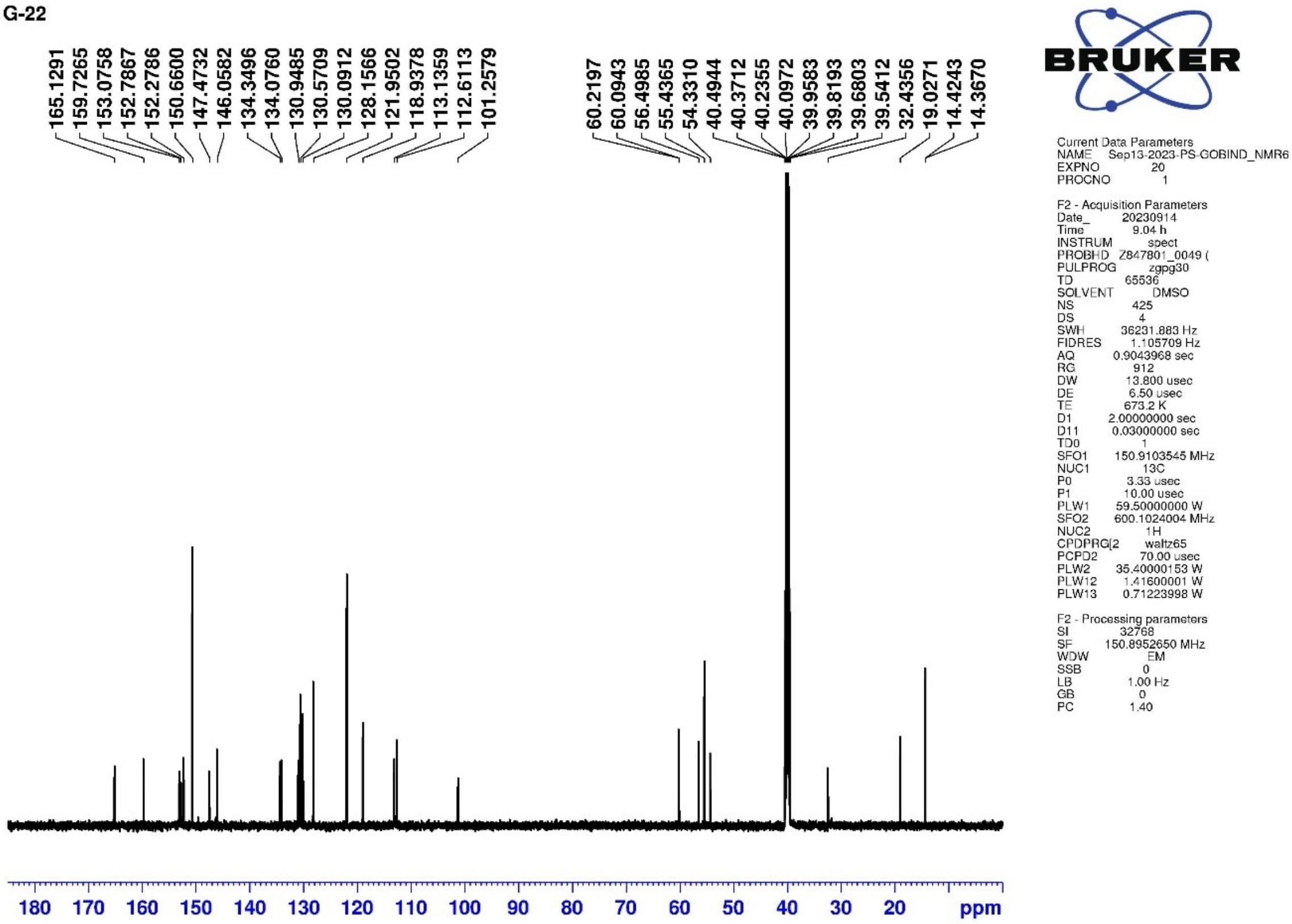

^{13}C NMR of Compound **10f**.

Single Mass Analysis
Tolerance = 5.0 PPM / DBE: min = -1.5, max = 50.0
Element prediction: Off
Number of isotope peaks used for i-FIT = 3

Monoisotopic Mass, Even Electron Ions
260 formula(e) evaluated with 1 results within limits (up to 50 closest results for each mass)
Elements Used:
C: 10-50 H: 10-100 N: 1-10 O: 1-5 Na: 1-1 S: 1-1
T-9 28 (0.257) Cm (1:230)
TOF MS ES+

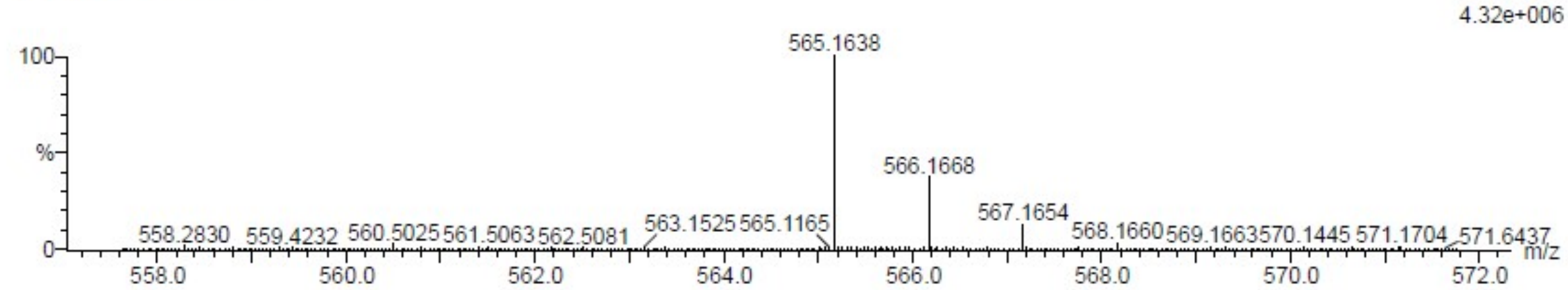

Minimum:				-1.5				
Maximum:		2.0	5.0	50.0				
Mass	Calc. Mass	mDa	PPM	DBE	i-FIT	Norm	Conf(%)	Formula
565.1638	565.1634	0.4	0.7	18.5	1012.2	n/a	n/a	C28 H26 N6 O4 Na S

HRMS of Compound **10f**.

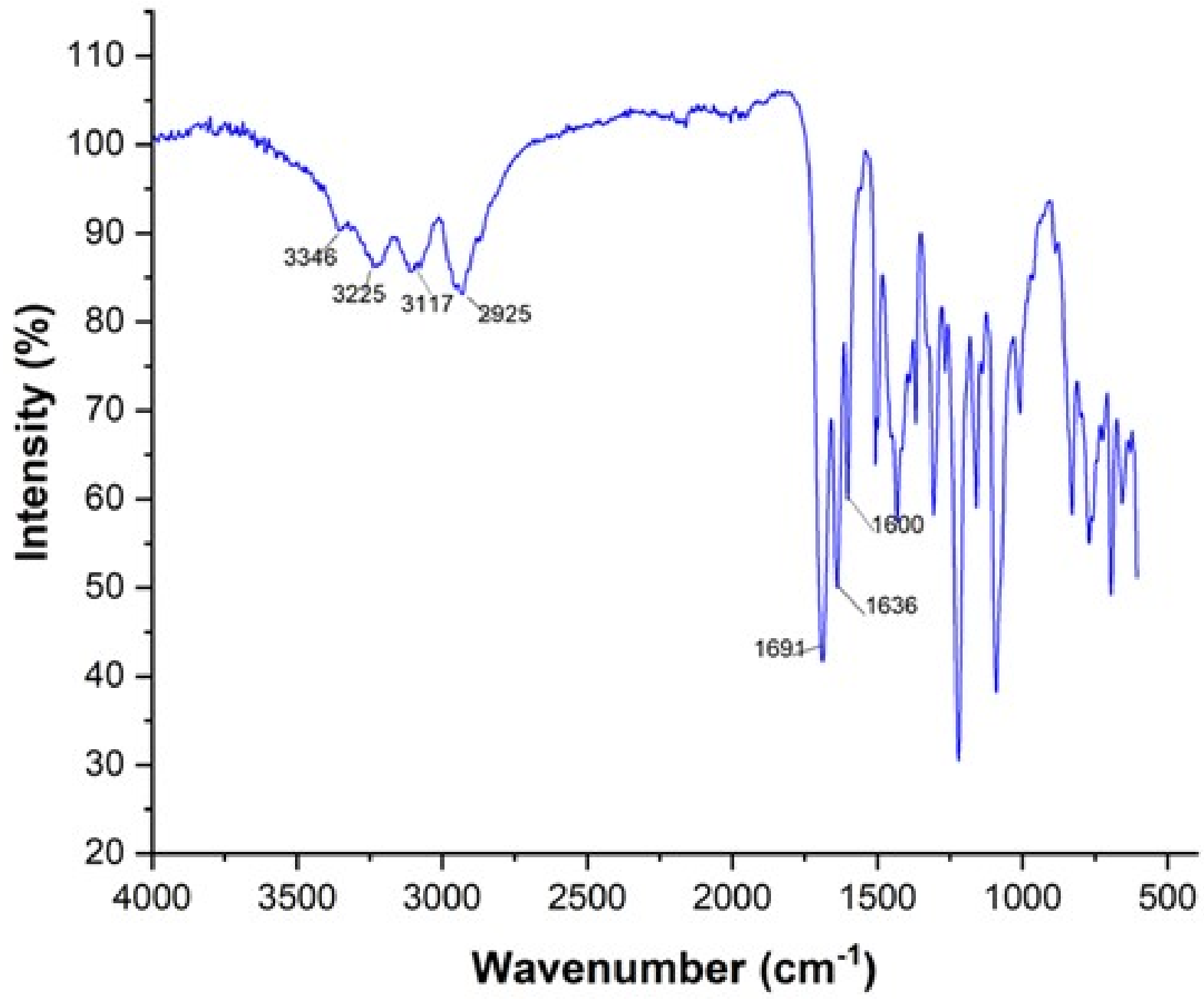

IR spectra of compound **10g**.

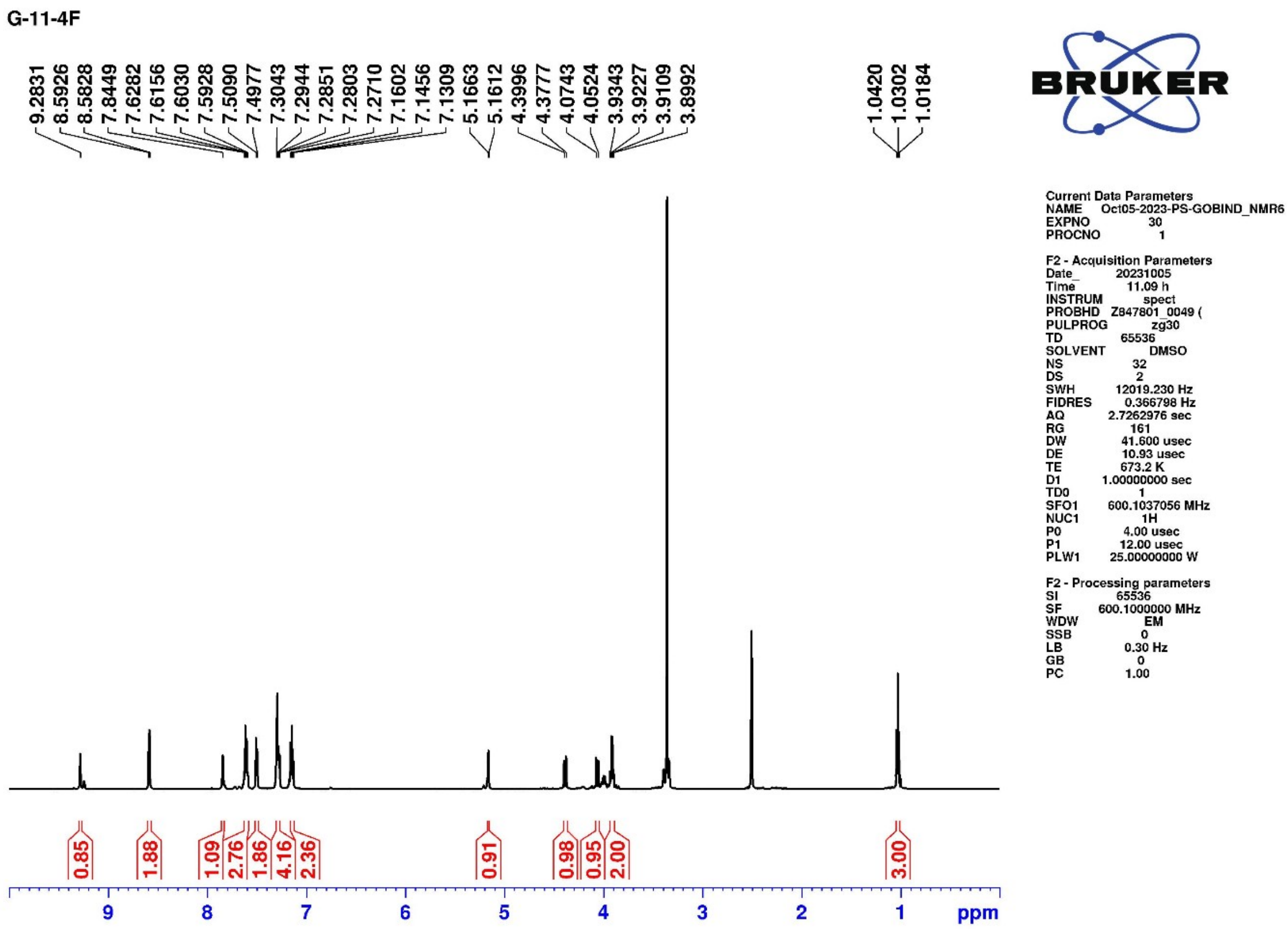

^{1}H NMR of Compound **10g**.

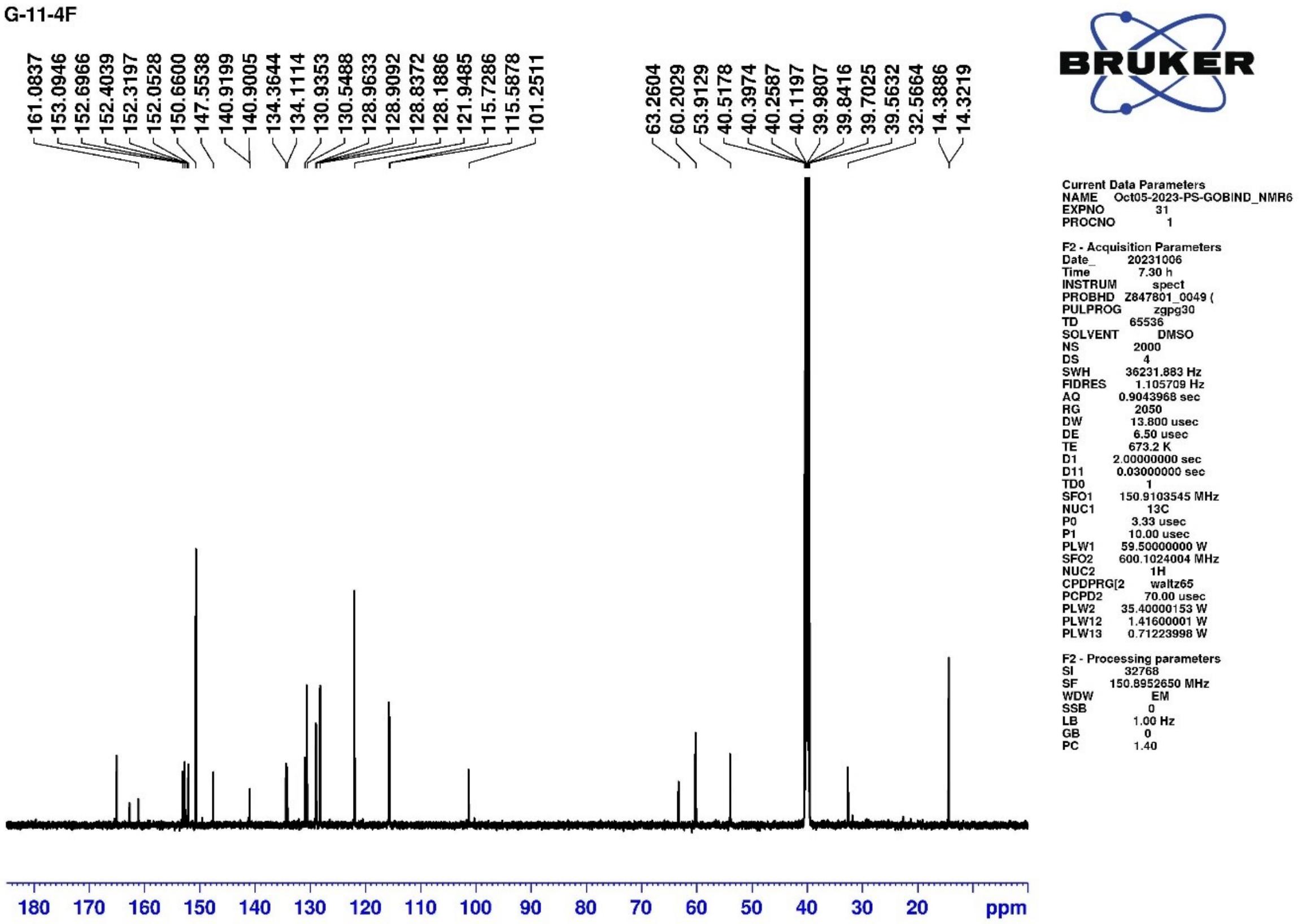

^{13}C NMR of Compound **10g**.

Single Mass Analysis
Tolerance = 5.0 PPM / DBE: min = -1.5, max = 50.0
Element prediction: Off
Number of isotope peaks used for i-FIT = 3

Monoisotopic Mass, Even Electron Ions
454 formula(e) evaluated with 2 results within limits (up to 50 closest results for each mass)
Elements Used:
C: 10-50 H: 10-100 N: 1-10 O: 1-5 Na: 1-1 S: 1-1 F: 1-2

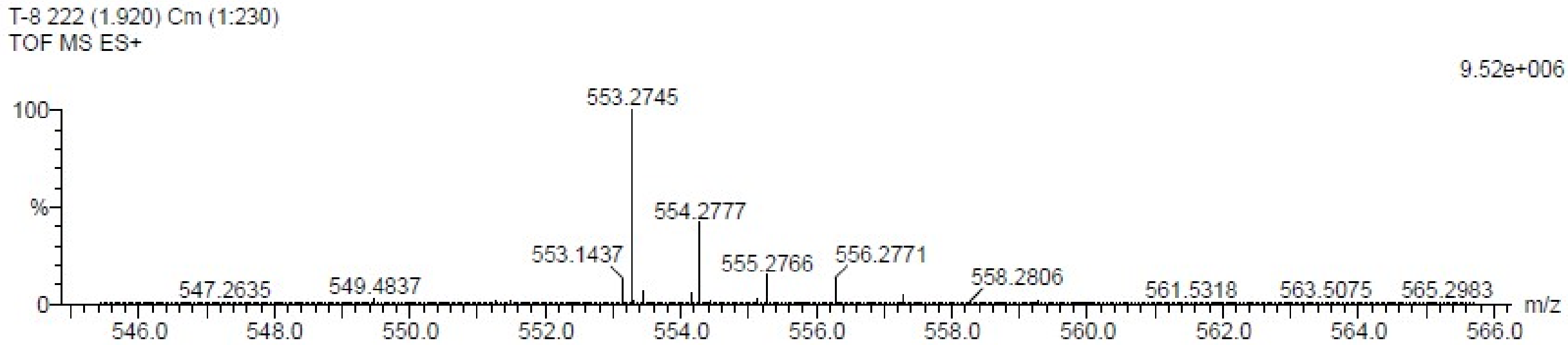

Mass	Calc. Mass	mDa	PPM	DBE	i-FIT	Norm	Conf(%)	Formula
Minimum:				-1.5				
Maximum:		2.0	5.0	50.0				
553.1437	553.1434	0.3	0.5	18.5	1319.0	0.310	73.33	C27 H23 N6 O3 Na S F
	553.1446	-0.9	-1.6	14.5	1320.0	1.322	26.67	C24 H24 N6 O4 Na S F2

HRMS of Compound **10g**.

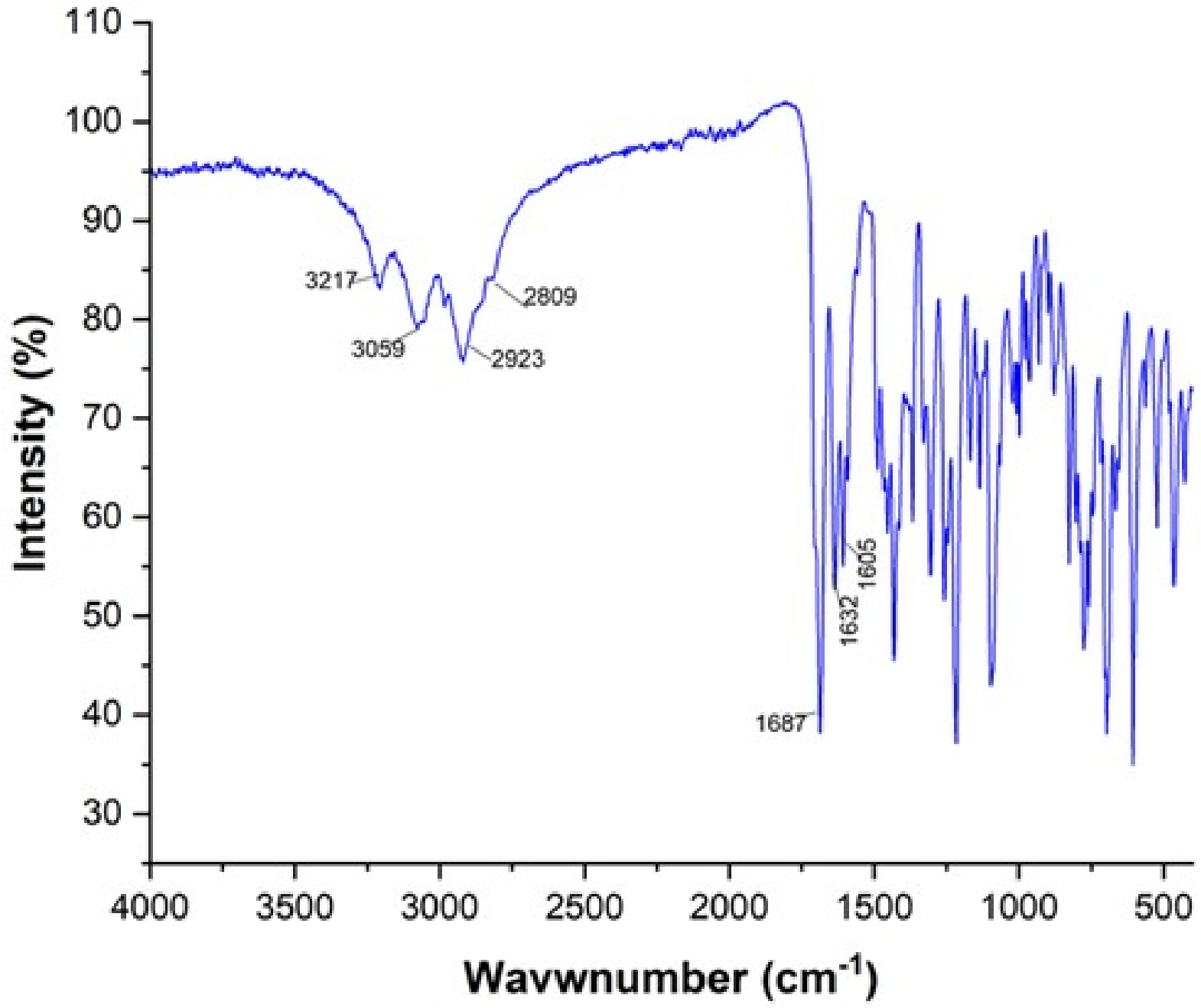

IR spectra of compound **10h**.

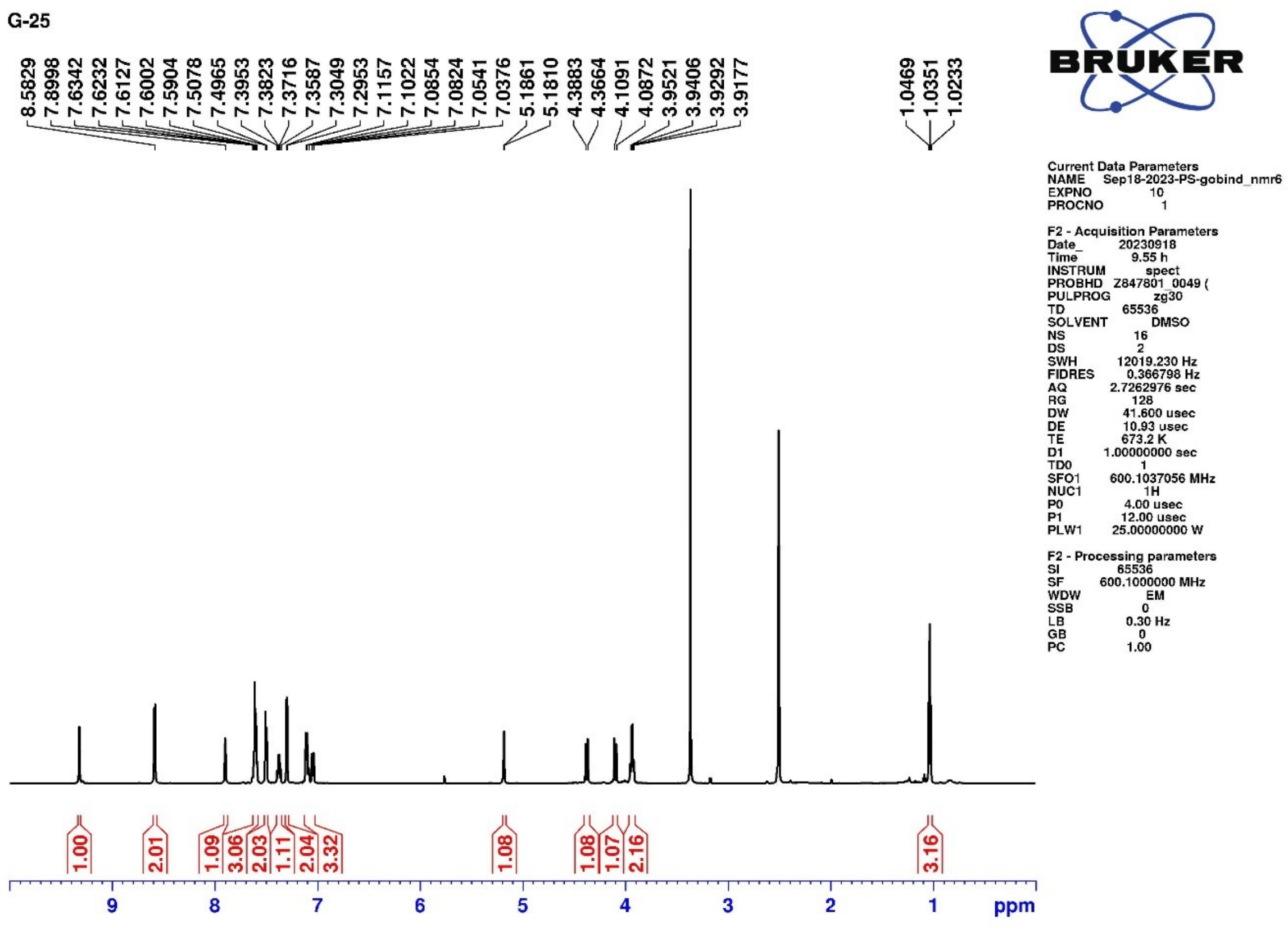

^{1}H NMR of Compound **10h**.

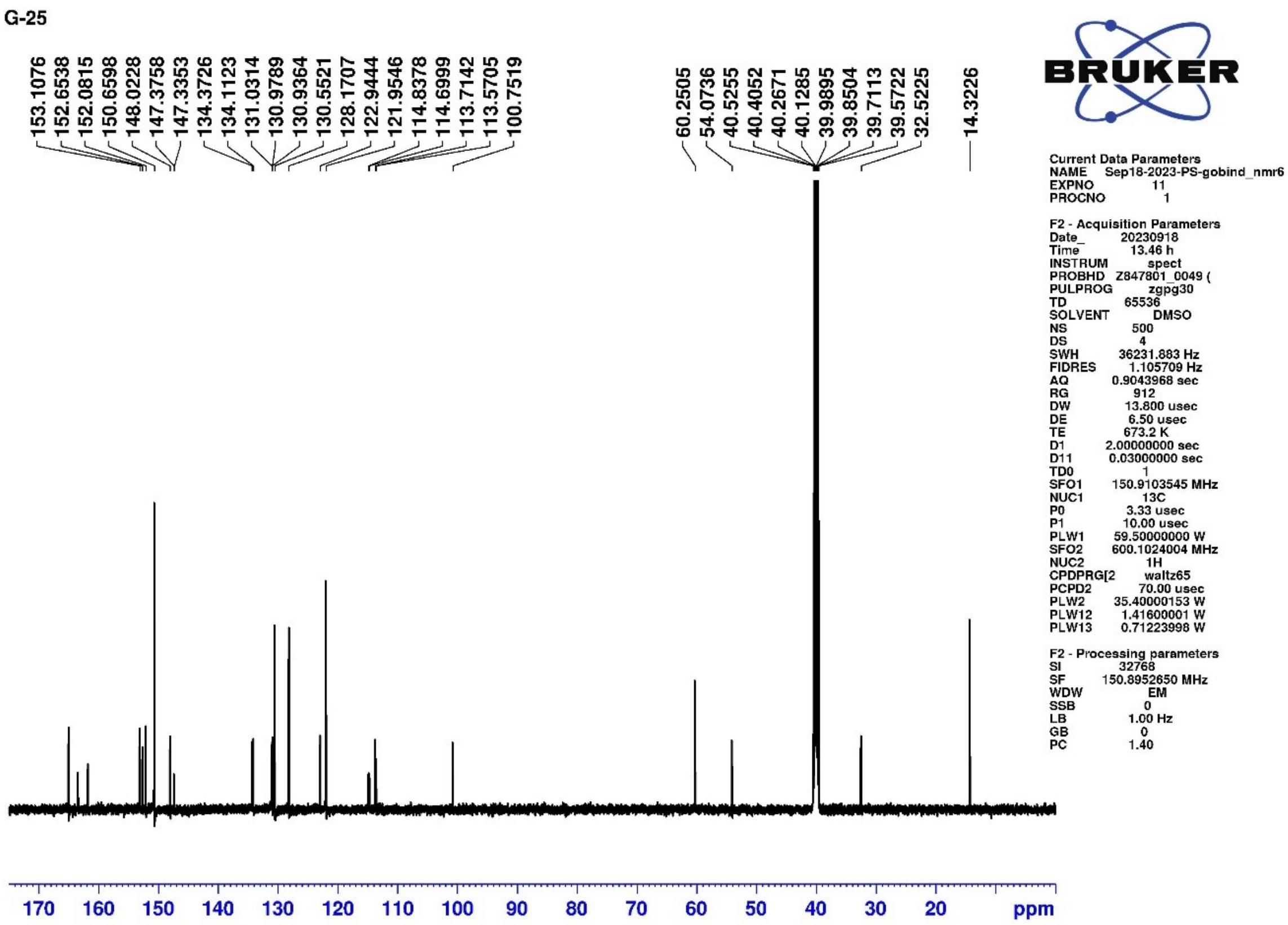

^{13}C NMR of Compound **10h**.

Single Mass Analysis
Tolerance = 5.0 PPM / DBE: min = -1.5, max = 50.0
Element prediction: Off
Number of isotope peaks used for i-FIT = 3

Monoisotopic Mass, Even Electron Ions
234 formula(e) evaluated with 1 results within limits (up to 50 closest results for each mass)
Elements Used:
C: 10-50 H: 10-100 N: 1-10 O: 1-5 F: 1-1 Na: 1-1 S: 1-1

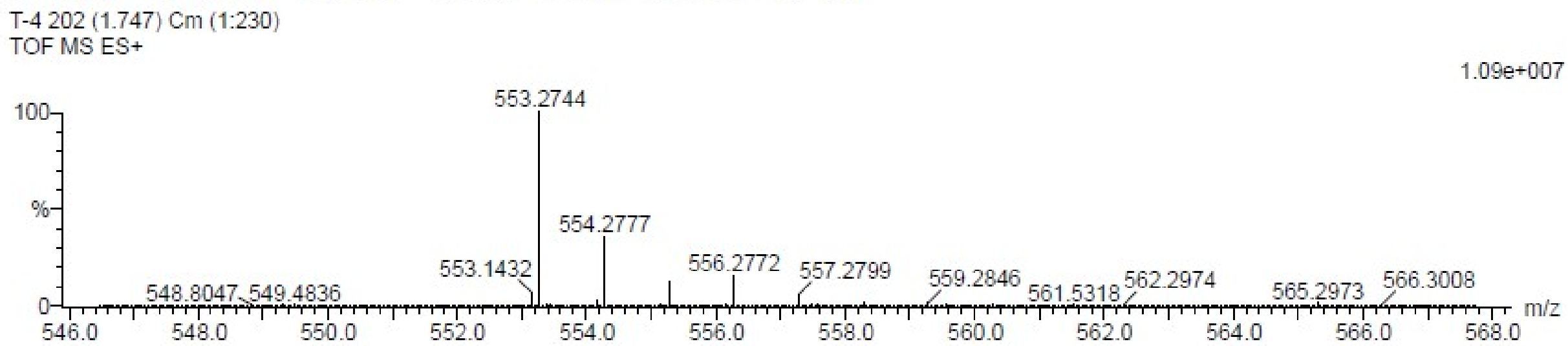

Minimum:				-1.5				
Maximum:		2.0	5.0	50.0				
Mass	Calc. Mass	mDa	PPM	DBE	i-FIT	Norm	Conf(%)	Formula
553.1432	553.1434	-0.2	-0.4	18.5	1128.0	n/a	n/a	C27 H23 N6 O3 F Na S

HRMS of Compound **10h**.

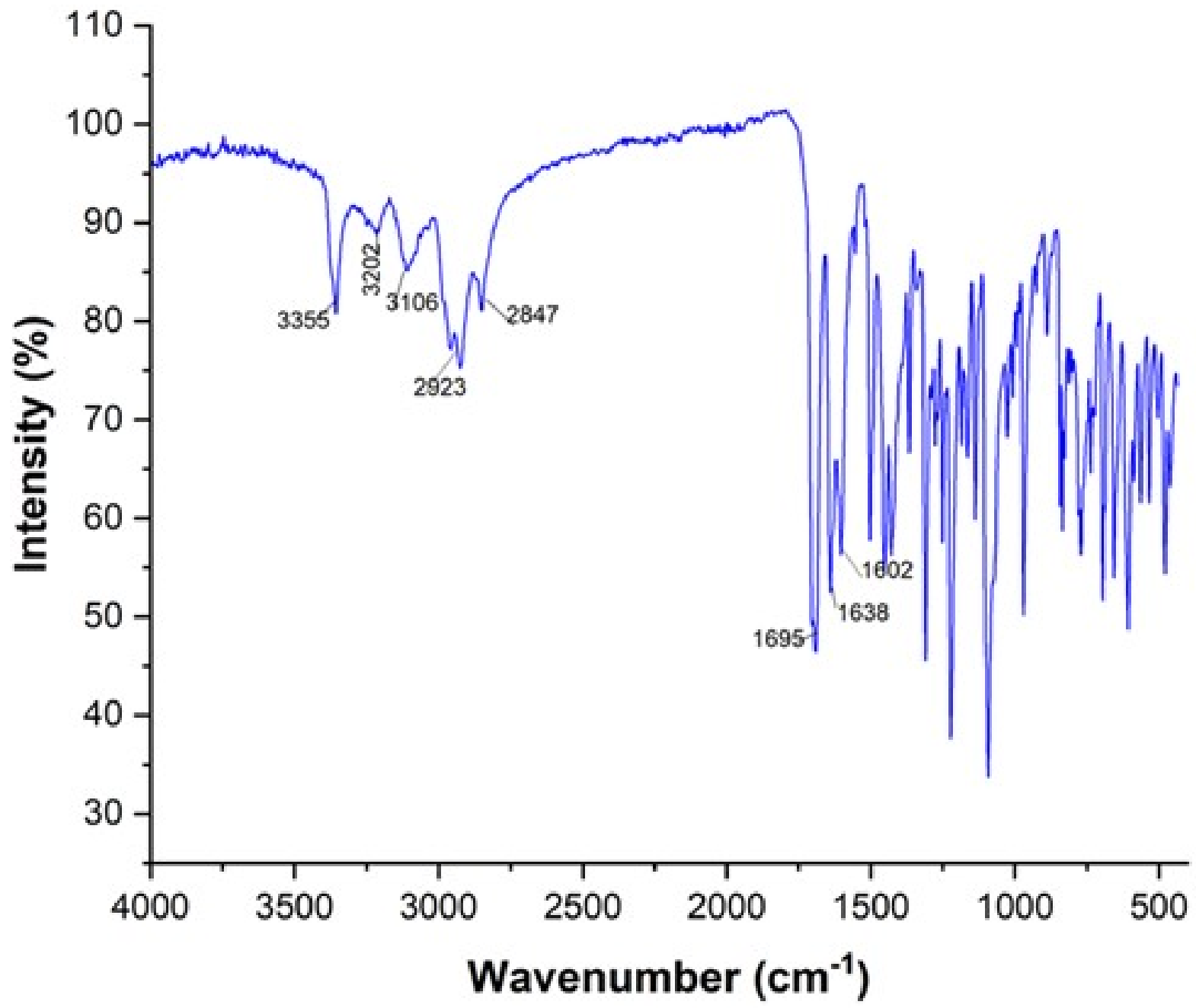

IR spectra of compound **10i**.

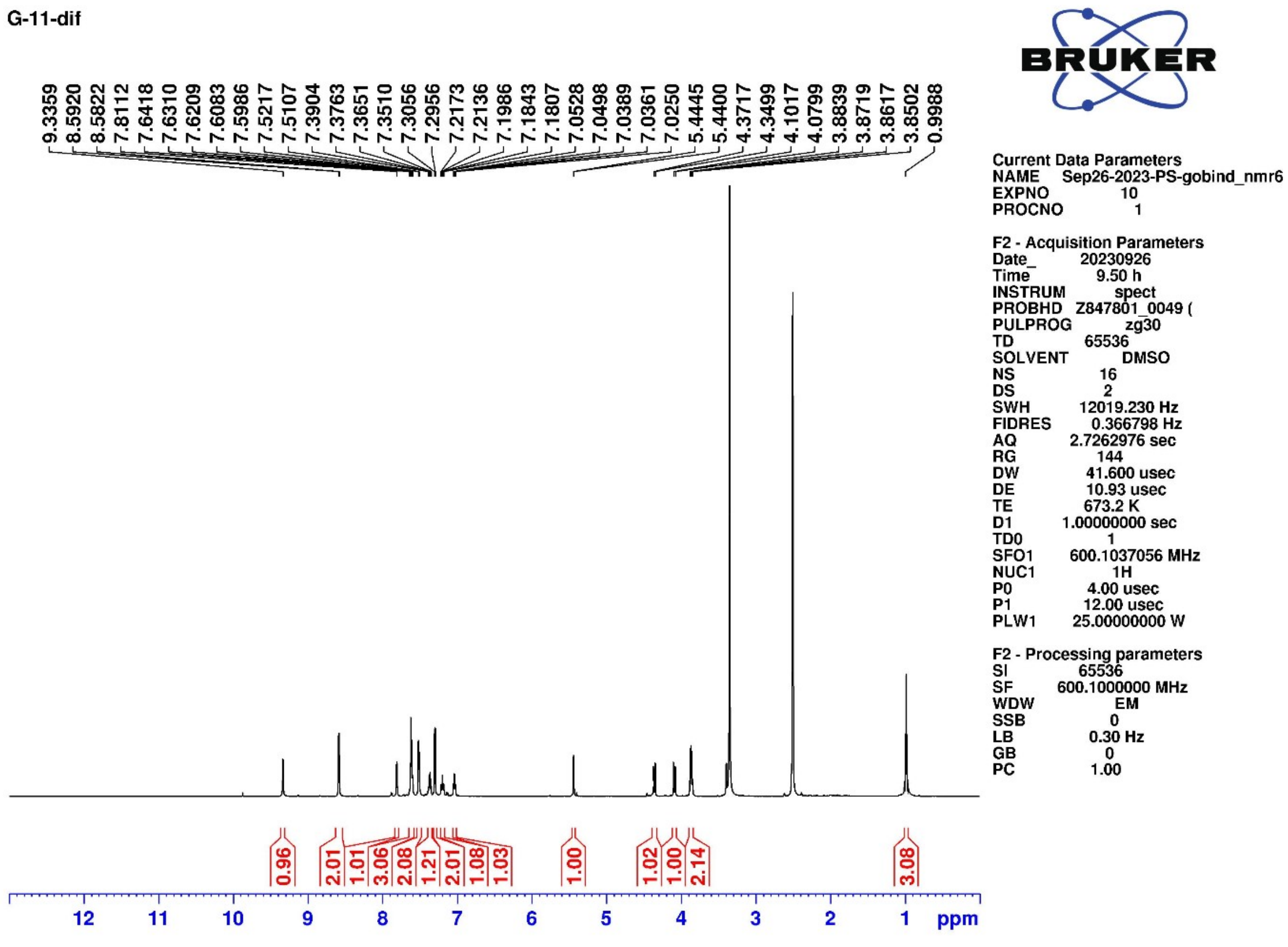

^{1}H NMR of Compound **10i**.

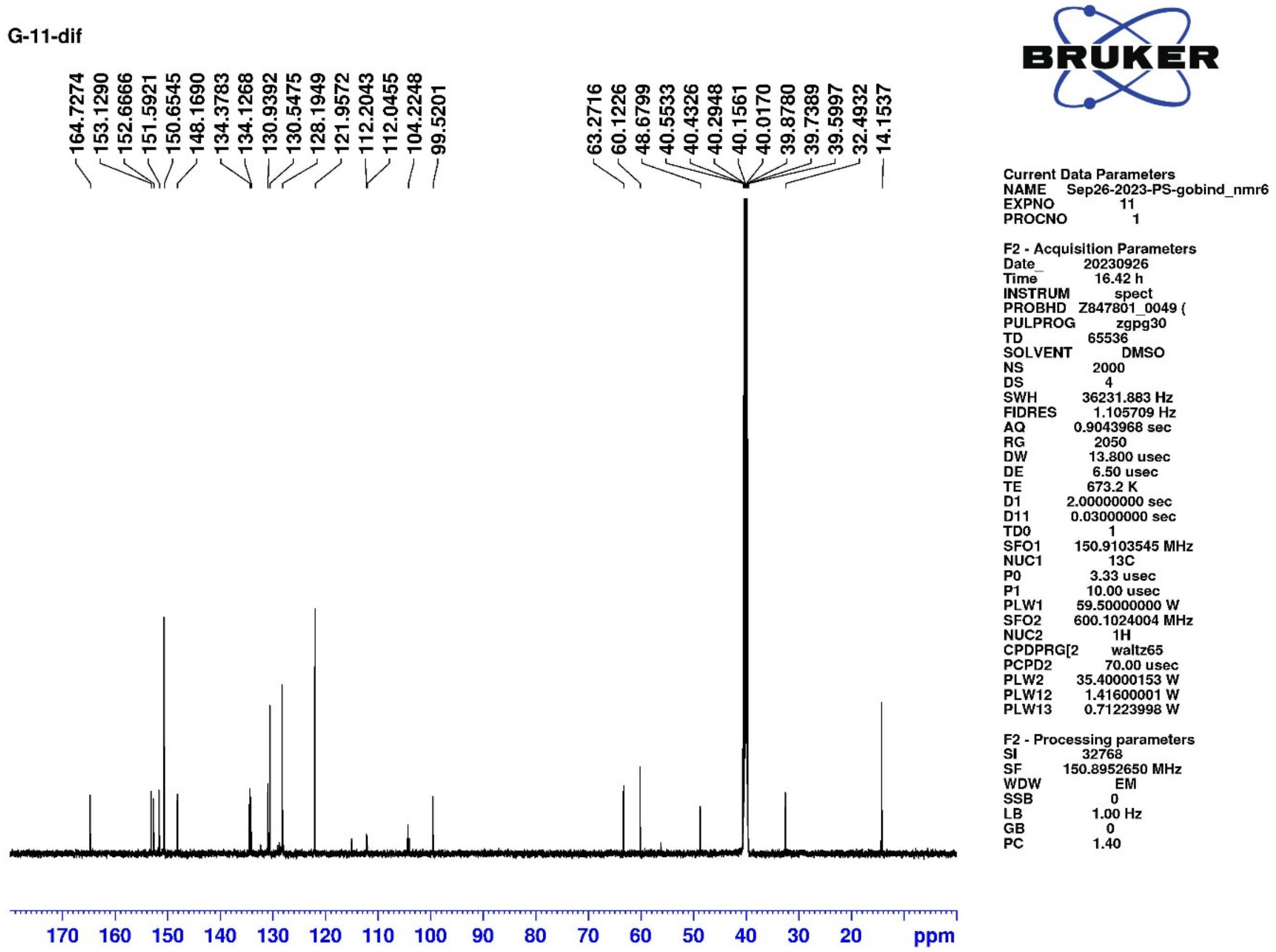

^{13}C NMR of Compound **10i**.

Single Mass Analysis
Tolerance = 5.0 PPM / DBE: min = -1.5, max = 50.0
Element prediction: Off
Number of isotope peaks used for i-FIT = 3

Monoisotopic Mass, Even Electron Ions
484 formula(e) evaluated with 2 results within limits (up to 50 closest results for each mass)
Elements Used:
C: 10-50 H: 10-100 N: 1-10 O: 1-5 F: 1-2 Na: 1-1 S: 1-1
T-5 221 (1.910) Cm (1:230)
TOF MS ES+

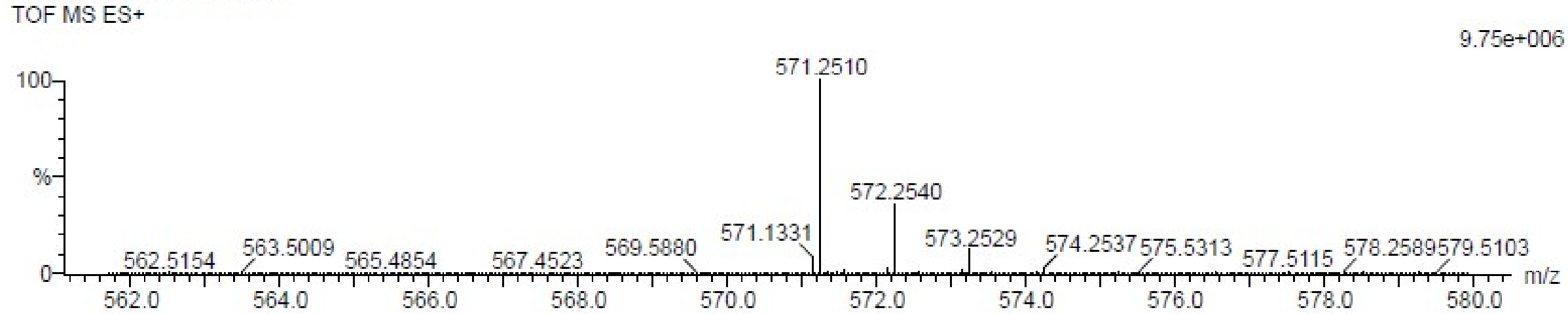

Minimum:				-1.5				
Maximum:		2.0	5.0	50.0				
Mass	Calc. Mass	mDa	PPM	DBE	i-FIT	Norm	Conf(%)	Formula
571.1331	571.1328	0.3	0.5	22.5	1163.7	0.040	96.12	C30 H21 N6 O2 F Na S
	571.1340	-0.9	-1.6	18.5	1166.9	3.250	3.88	C27 H22 N6 O3 F2 Na S

HRMS of Compound **10i**.

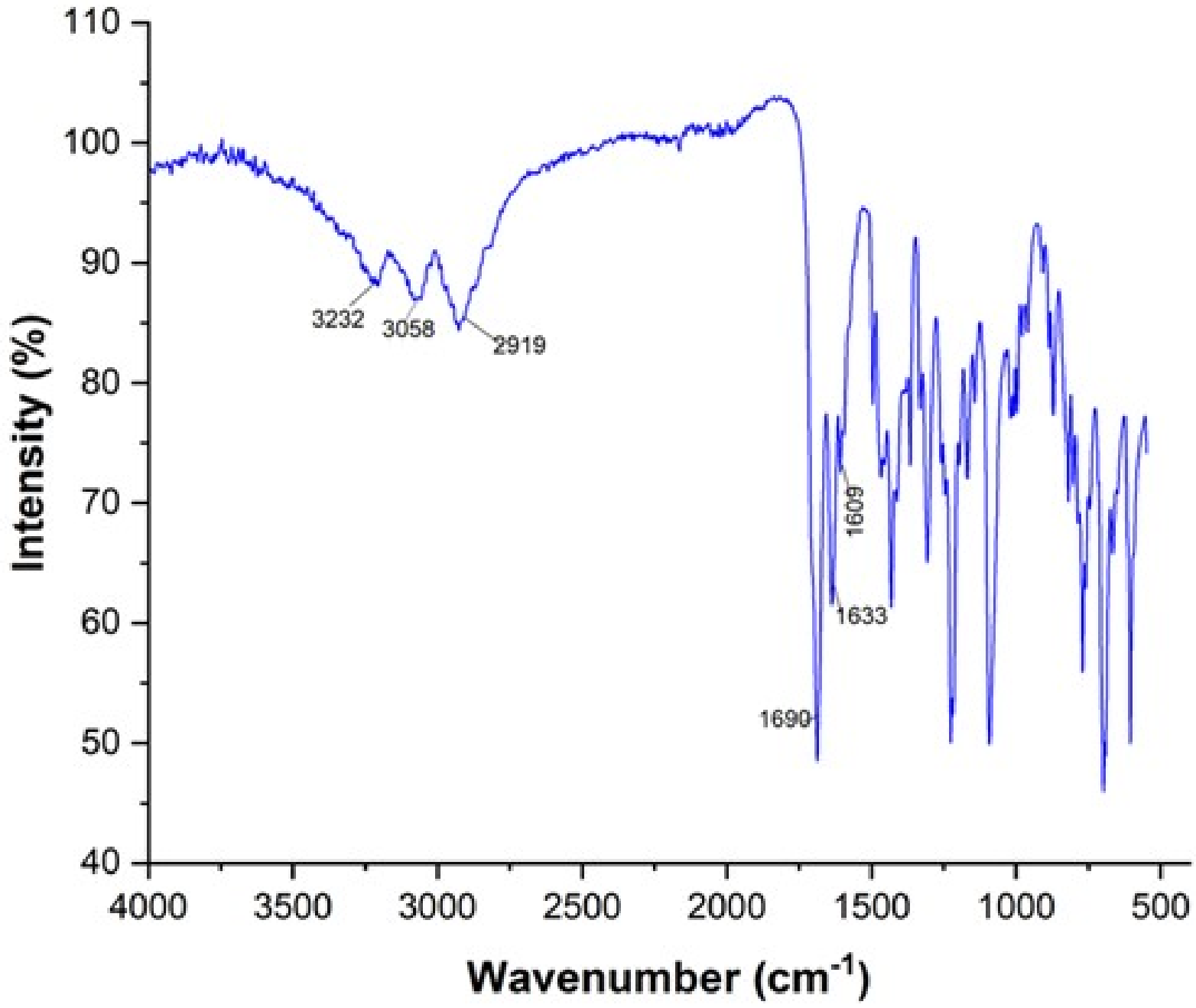

IR spectra of compound **10j**.

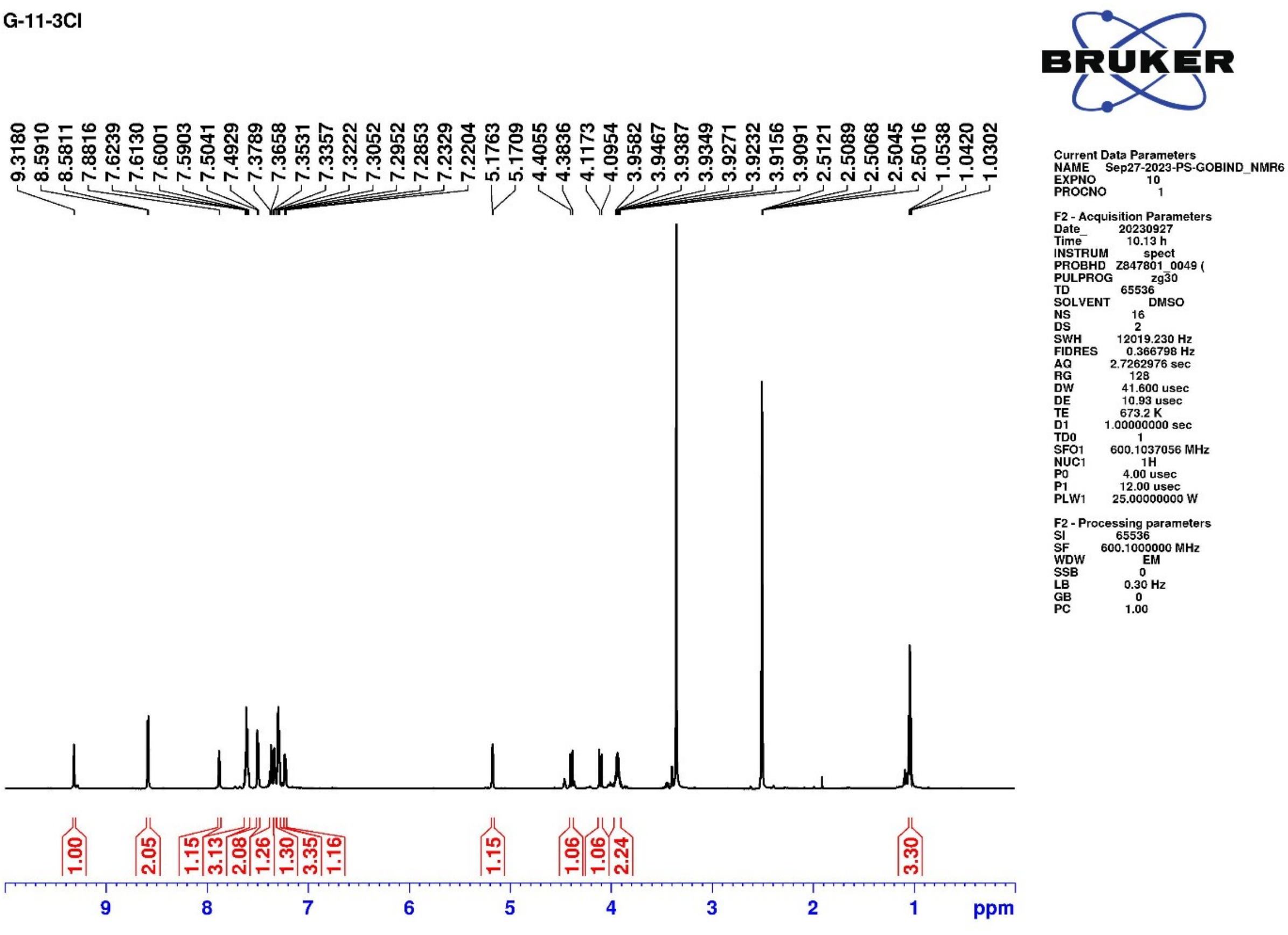

^{1}H NMR of Compound **10j**.

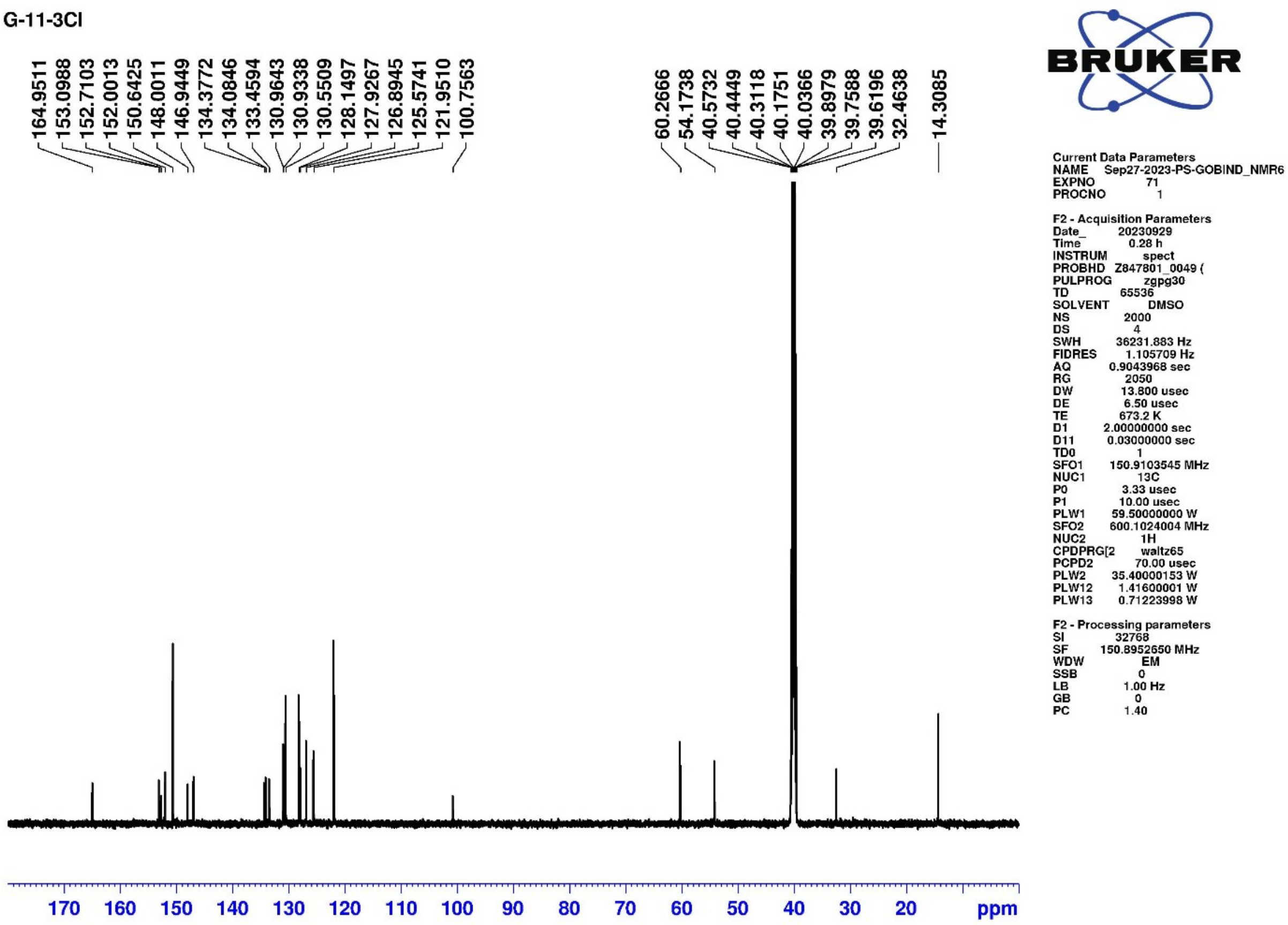

^{13}C NMR of Compound **10j**.

Single Mass Analysis
Tolerance = 5.0 PPM / DBE: min = -1.5, max = 50.0
Element prediction: Off
Number of isotope peaks used for i-FIT = 3

Monoisotopic Mass, Even Electron Ions
234 formula(e) evaluated with 1 results within limits (up to 50 closest results for each mass)
Elements Used:
C: 10-50 H: 10-100 N: 1-10 O: 1-5 Na: 1-1 S: 1-1 Cl: 1-1
T-7 201 (1.740) Cm (1:230)
TOF MS ES+

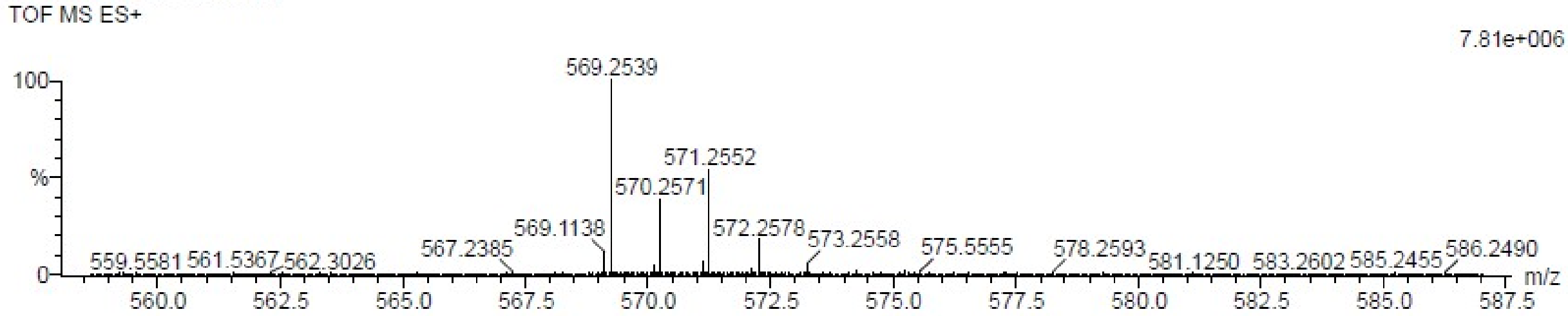

Mass	Calc. Mass	mDa	PPM	DBE	i-FIT	Norm	Conf(%)	Formula
Minimum:				-1.5				
Maximum:		2.0	5.0	50.0				
569.1138	569.1139	-0.1	-0.2	18.5	1248.8	n/a	n/a	C27 H23 N6 O3 Na S Cl

HRMS of Compound **10j**.

Spectral data of compound 11a-j

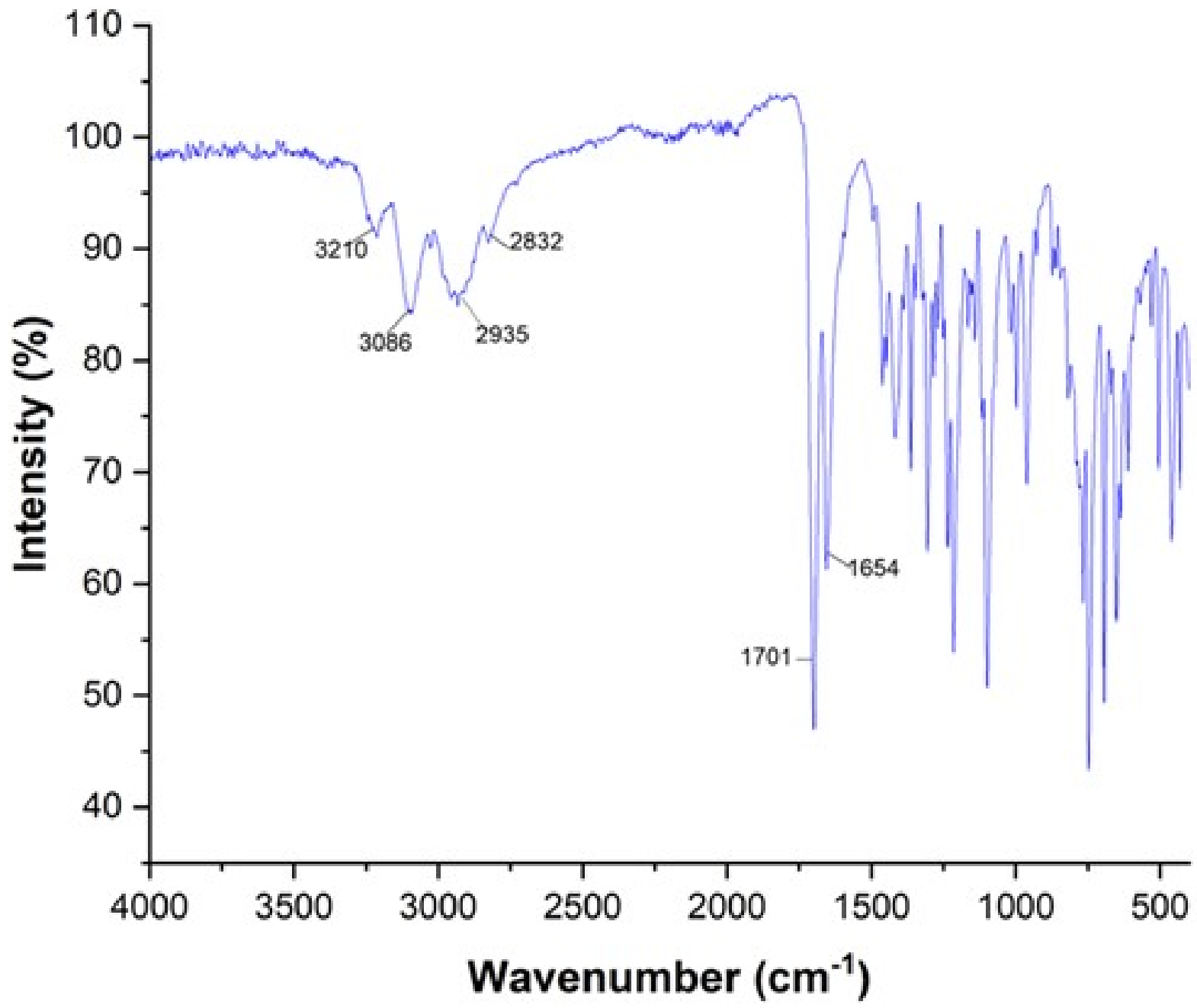

IR spectra of compound **11a.**

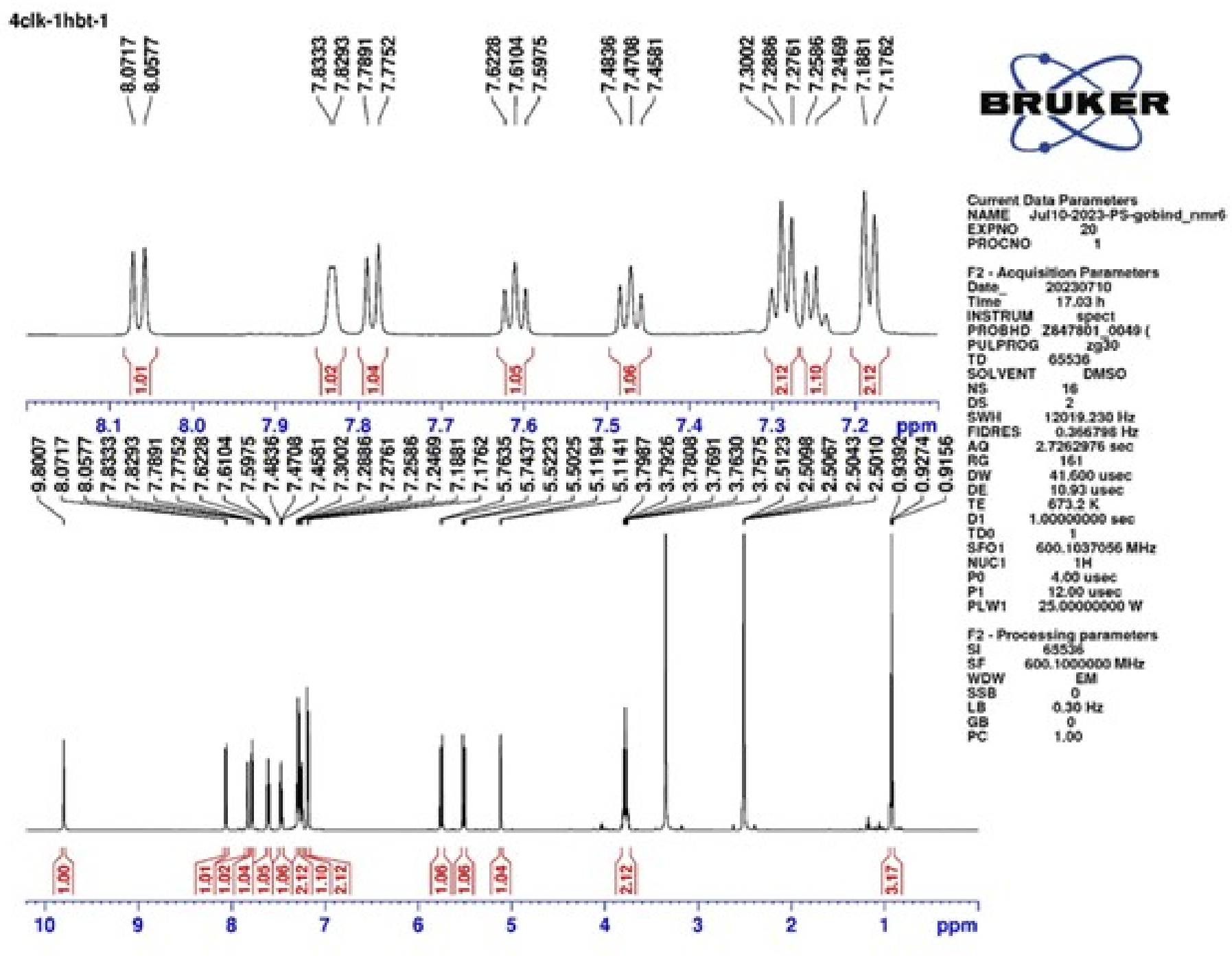

^{1}HNMR compound **11a**.

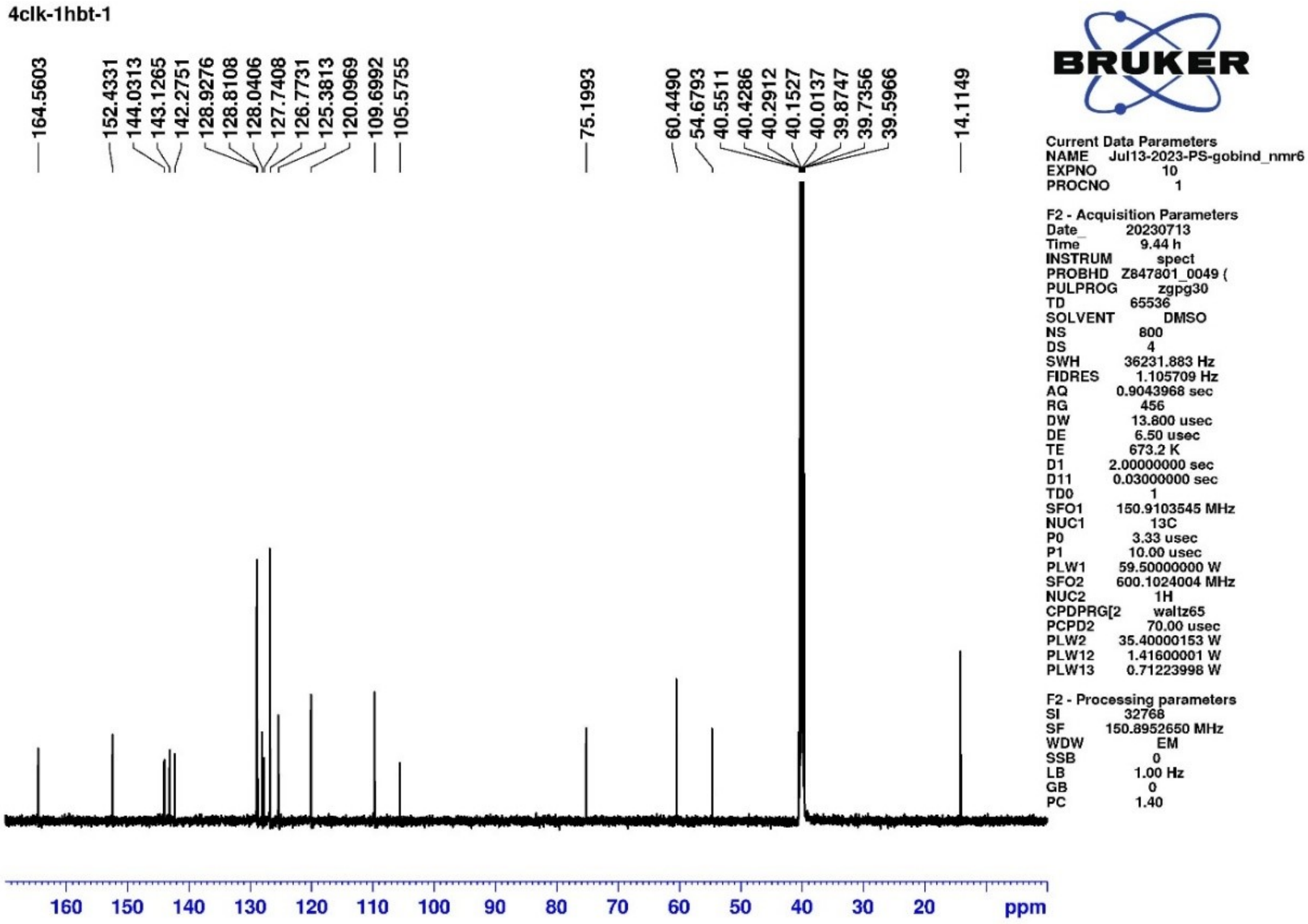

^{13}CNMR (CPD) compound **11a**.

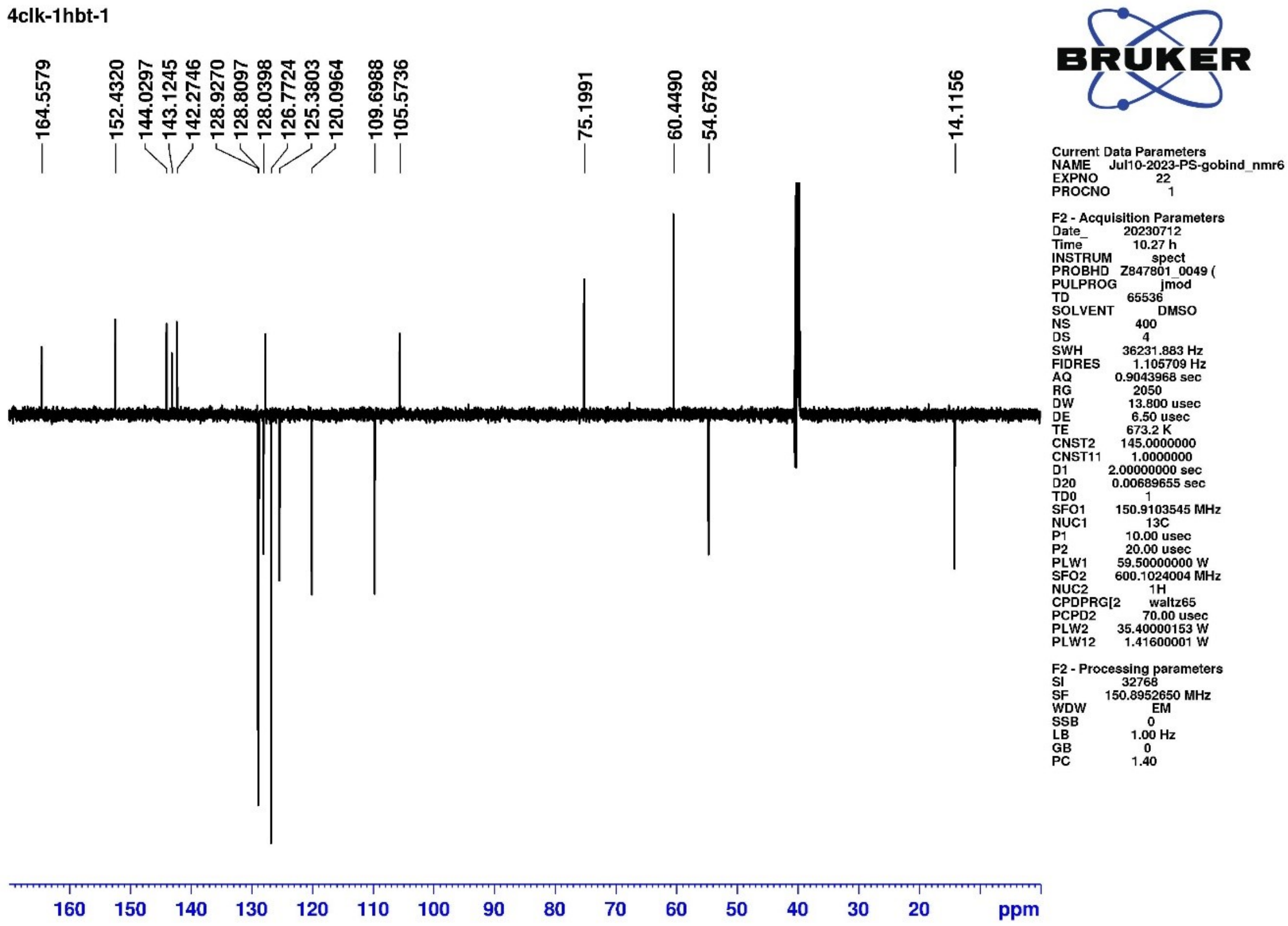

^{13}CNMR (APT) compound **11a**.

Single Mass Analysis
Tolerance = 5.0 PPM / DBE: min = -1.5, max = 50.0
Element prediction: Off
Number of isotope peaks used for i-FIT = 3

Monoisotopic Mass, Even Electron Ions
98 formula(e) evaluated with 1 results within limits (up to 50 closest results for each mass)
Elements Used:
C: 10-50 H: 10-100 N: 1-5 O: 1-5 Na: 1-1
H-1 205 (1.773) Cm (2:230)
TOF MS ES+

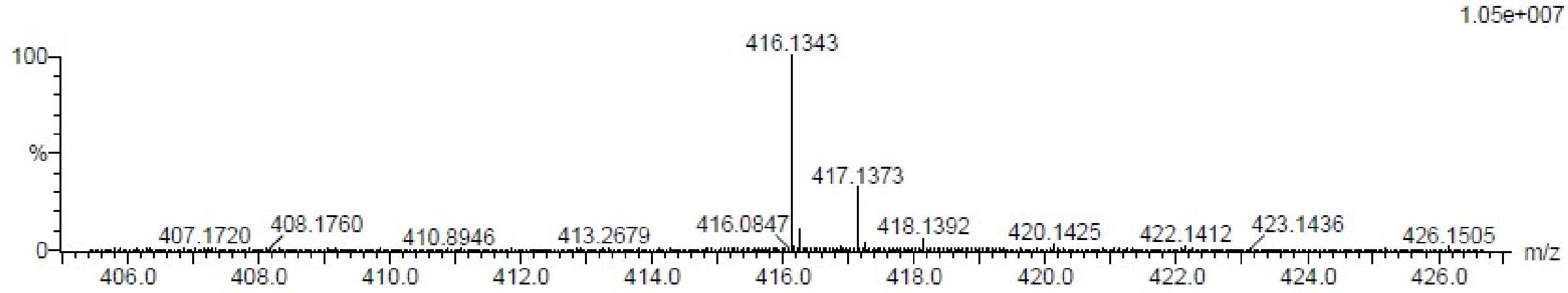

Mass	Calc. Mass	mDa	PPM	DBE	i-FIT	Norm	Conf(%)	Formula
Minimum:				-1.5				
Maximum:		10.0	5.0	50.0				
416.1343	416.1335	0.8	1.9	13.5	1297.8	n/a	n/a	C20 H19 N5 O4 Na

HRMS of compound **11a**.

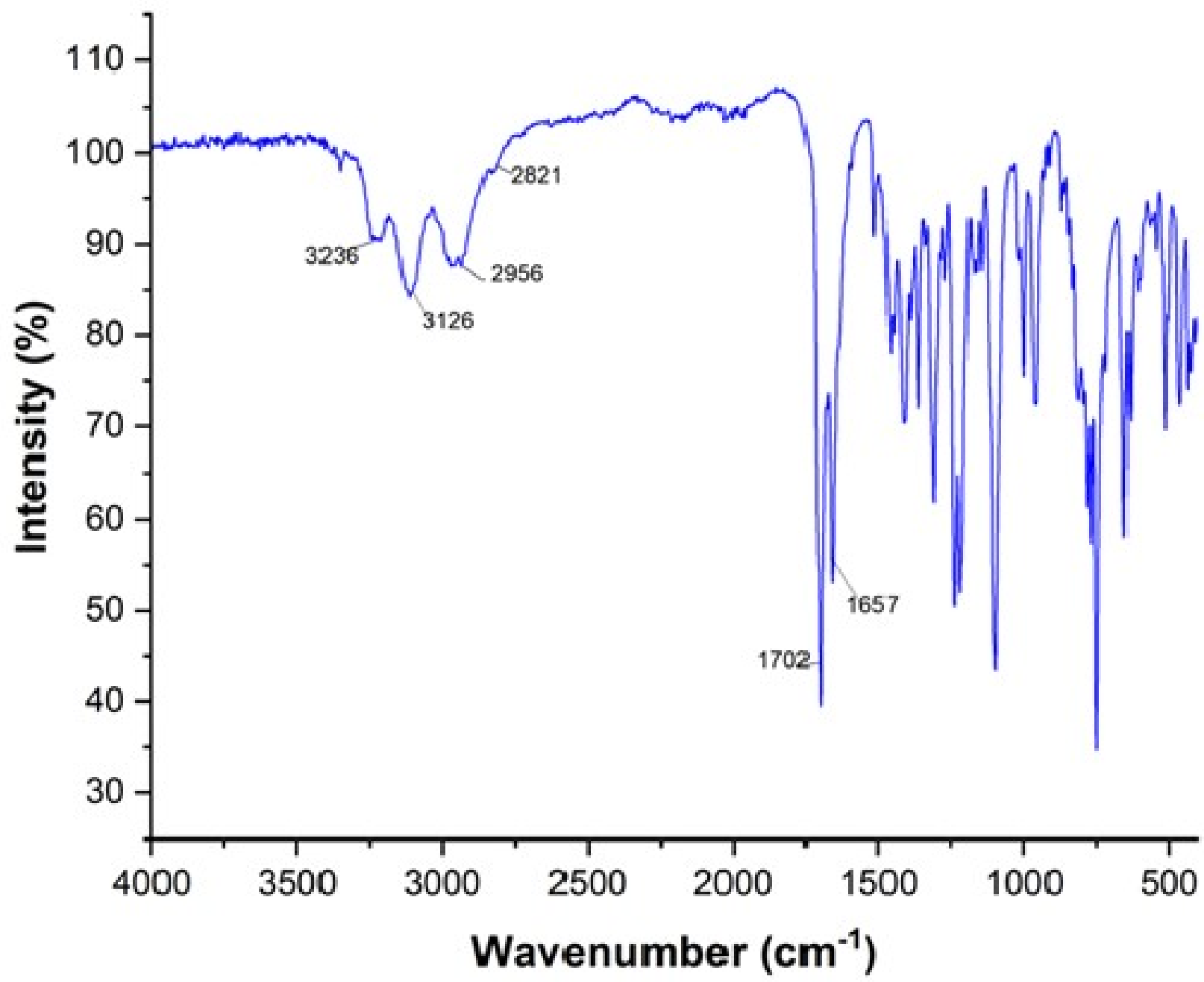

IR spectra of compound **11b.**

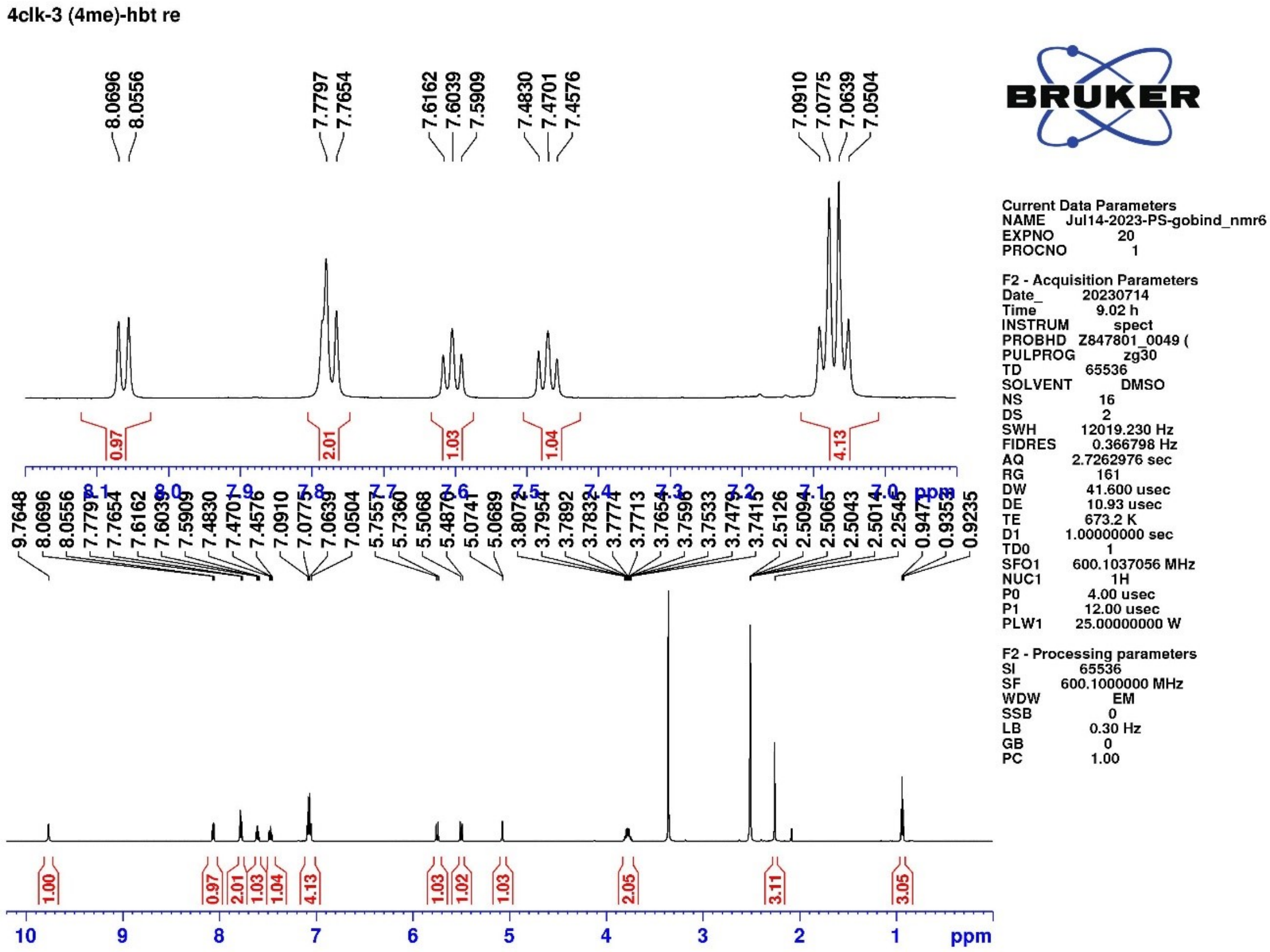

^{1}HNMR compound **11b**.

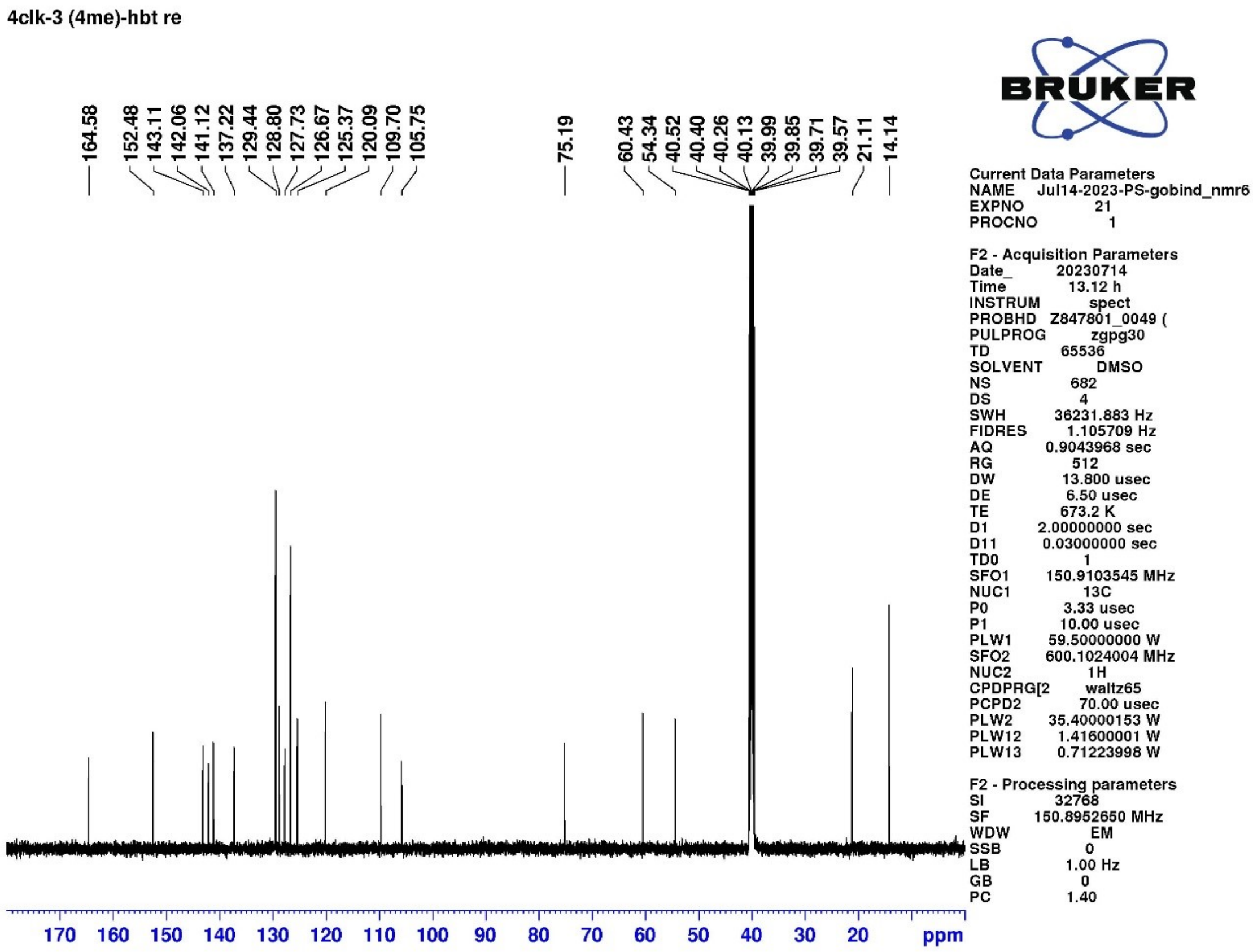

^{13}CNMR compound **11b**.

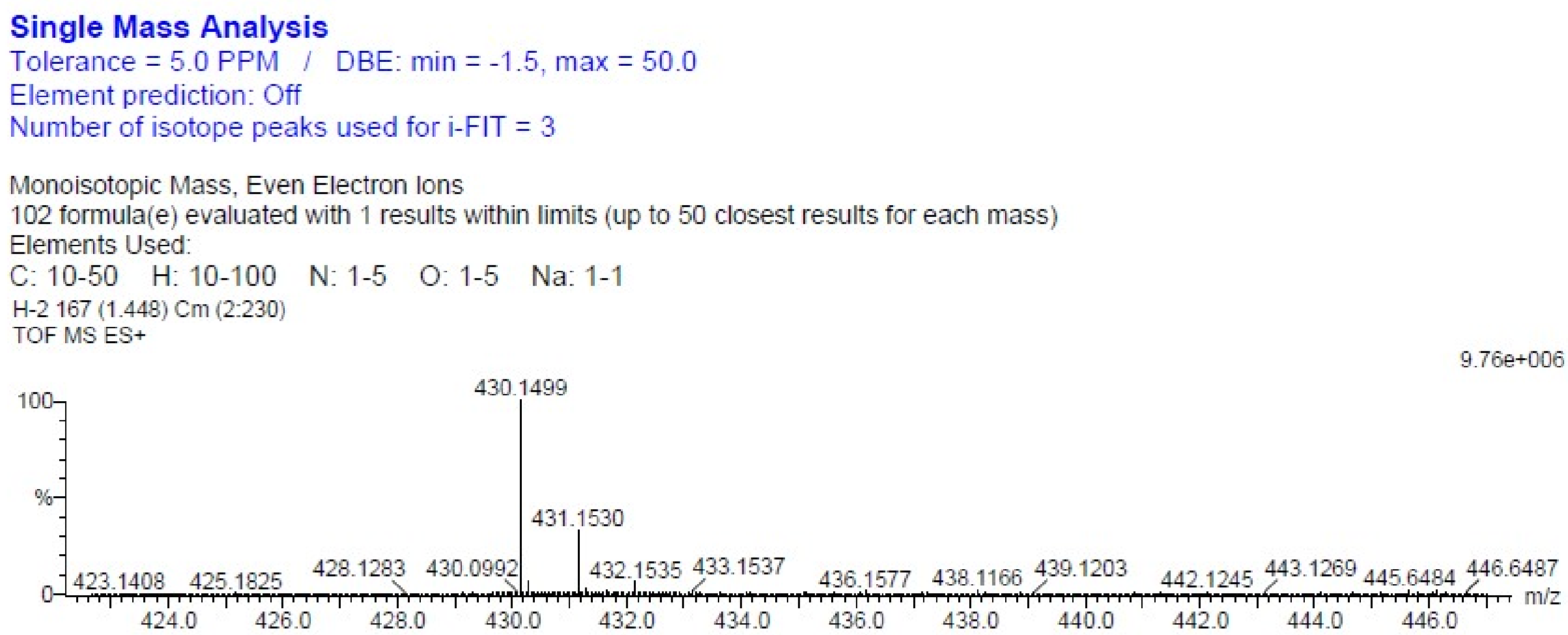

Minimum:				-1.5				
Maximum:		10.0	5.0	50.0				
Mass	Calc. Mass	mDa	PPM	DBE	i-FIT	Norm	Conf(%)	Formula
430.1499	430.1491	0.8	1.9	13.5	1284.5	n/a	n/a	C21 H21 N5 O4 Na

HRMS of compound **11b**.

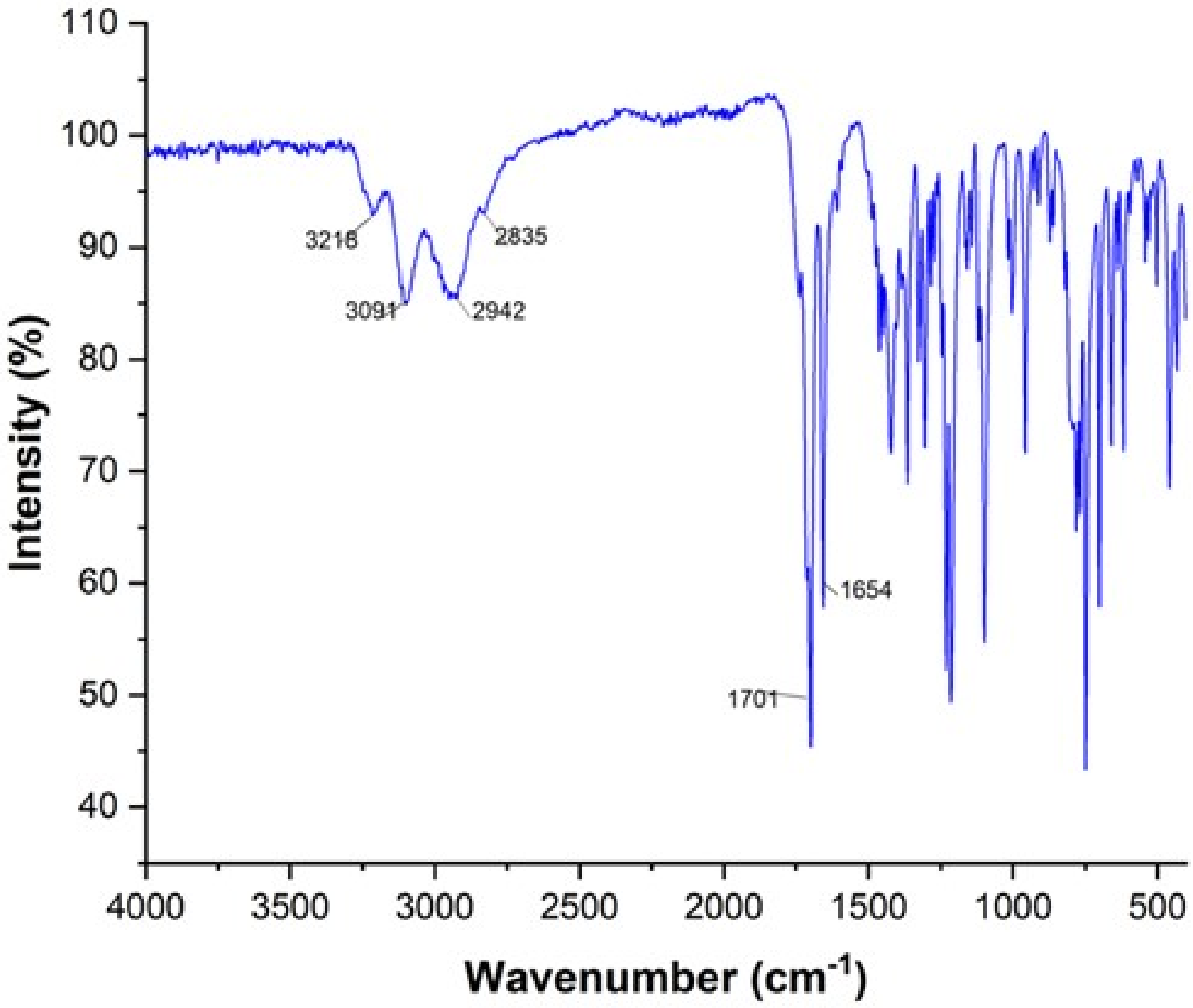

IR spectra of compound **11c.**

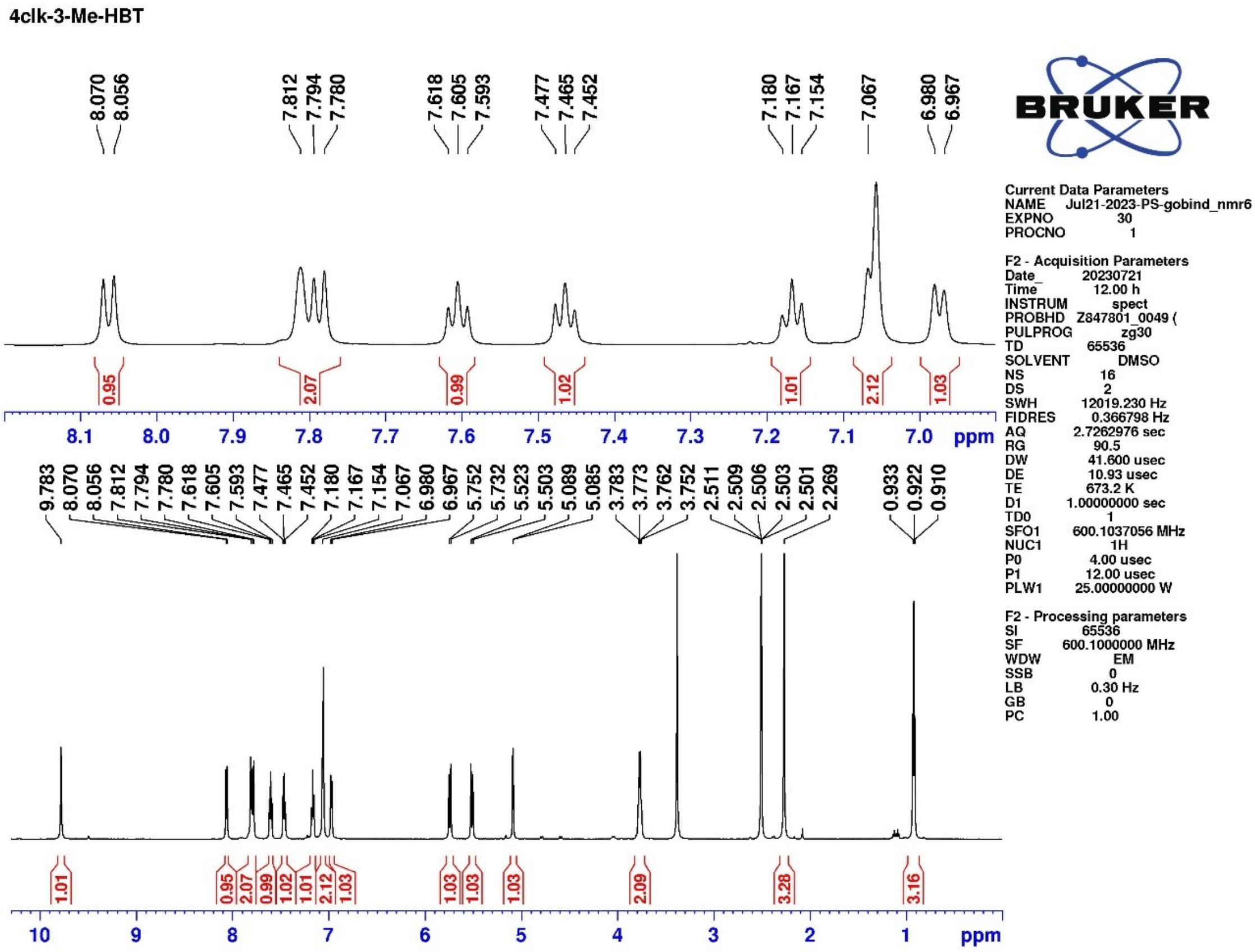

^{1}HNMR compound **11c**.

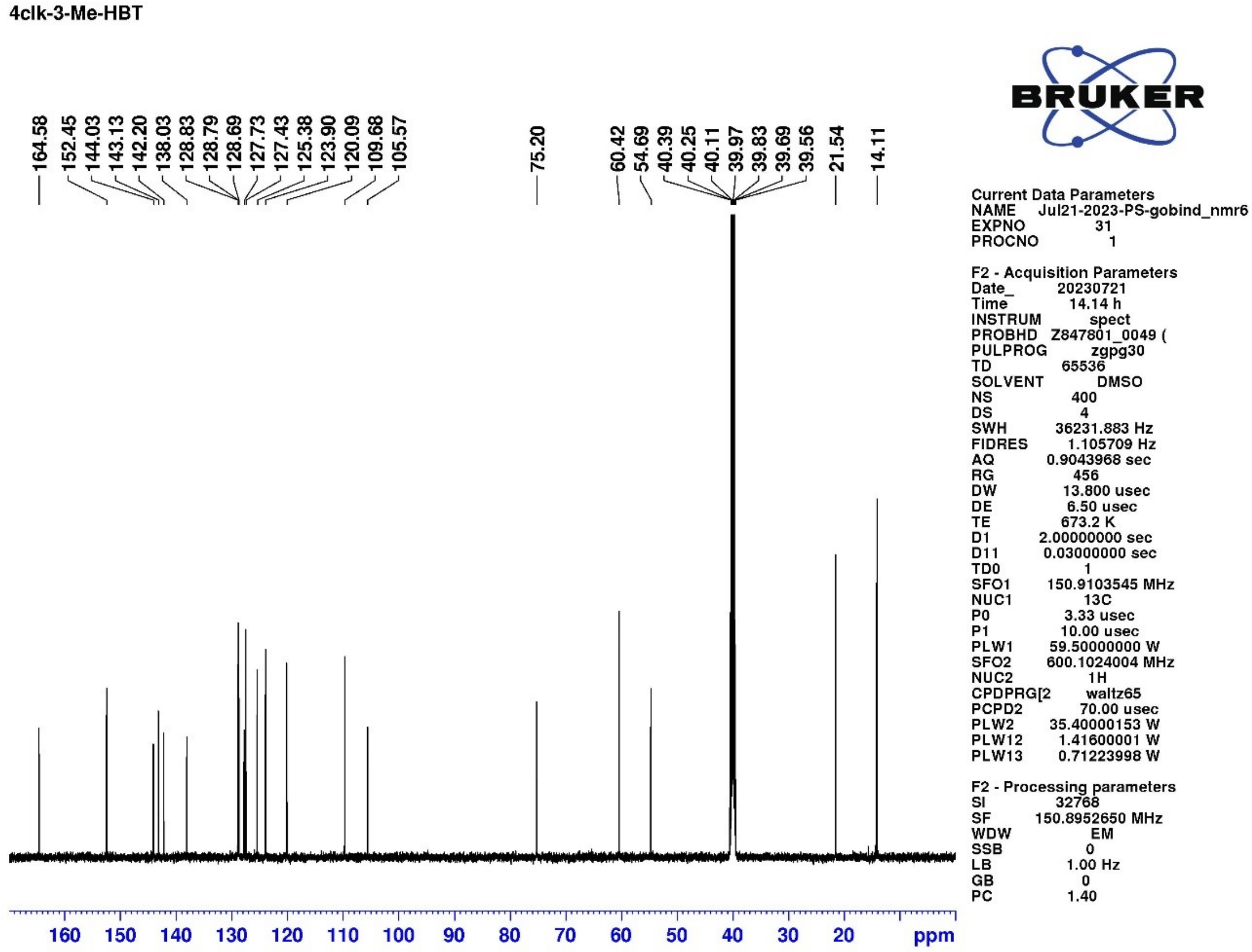

^{13}CNMR compound **11c**.

Single Mass Analysis
Tolerance = 5.0 PPM / DBE: min = -1.5, max = 50.0
Element prediction: Off
Number of isotope peaks used for i-FIT = 3

Monoisotopic Mass, Even Electron Ions
102 formula(e) evaluated with 1 results within limits (up to 50 closest results for each mass)
Elements Used:
C: 10-50 H: 10-100 N: 1-5 O: 1-5 Na: 1-1
H-6 223 (1.928) Cm (2:230)
TOF MS ES+

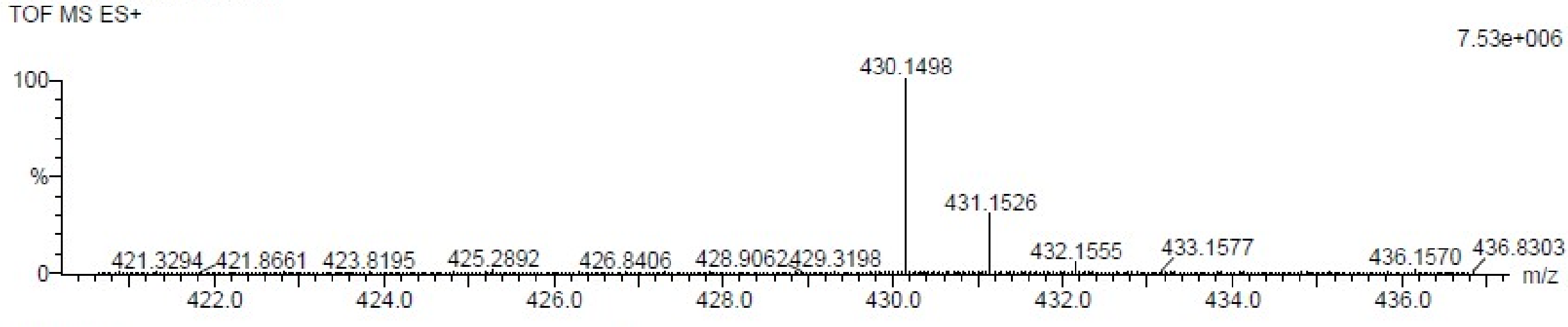

Minimum:				-1.5				
Maximum:		10.0	5.0	50.0				
Mass	Calc. Mass	mDa	PPM	DBE	i-FIT	Norm	Conf(%)	Formula
430.1498	430.1491	0.7	1.6	13.5	1270.9	n/a	n/a	C21 H21 N5 O4 Na

HRMS of compound **11c**.

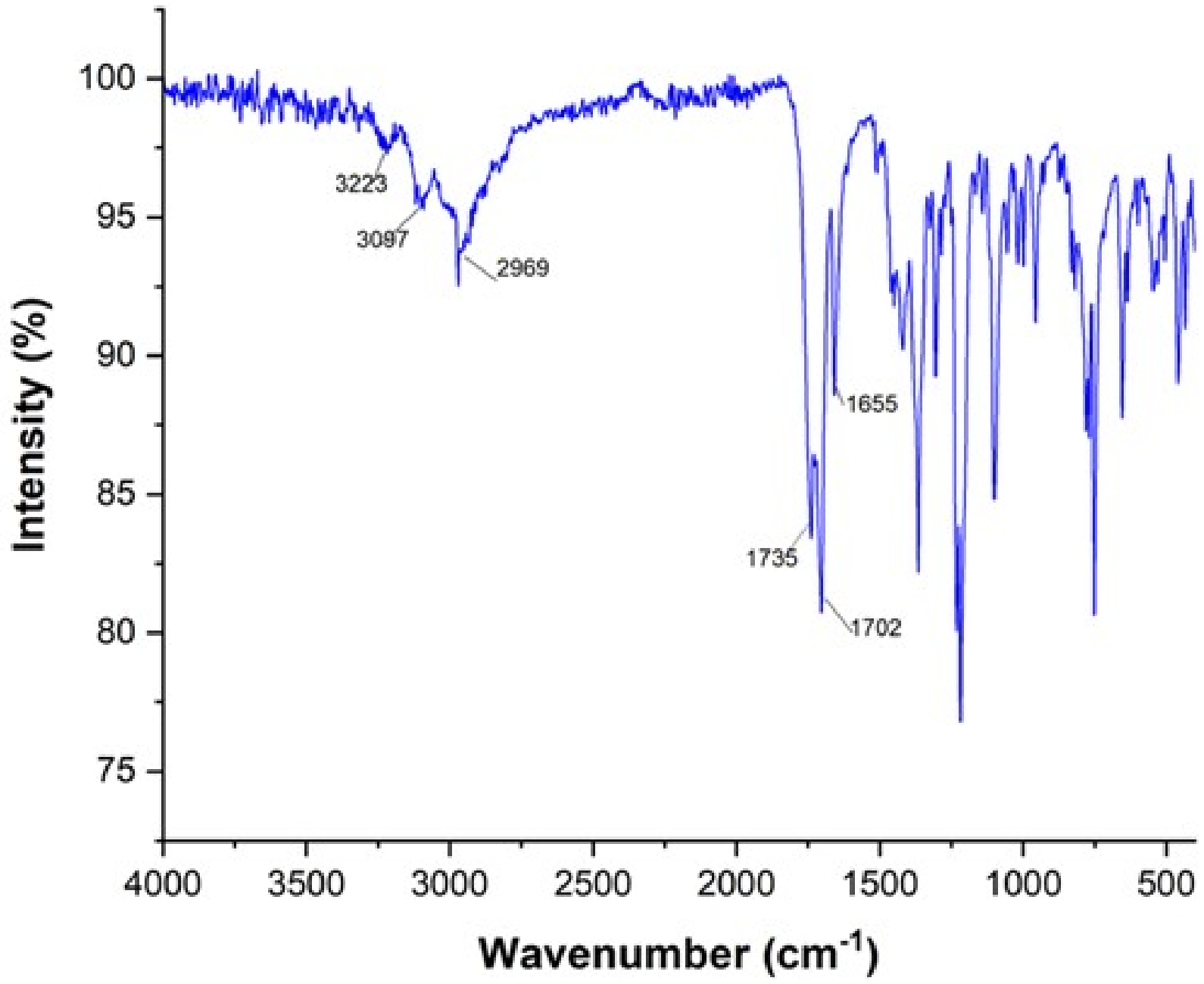

IR spectra of compound **11d.**

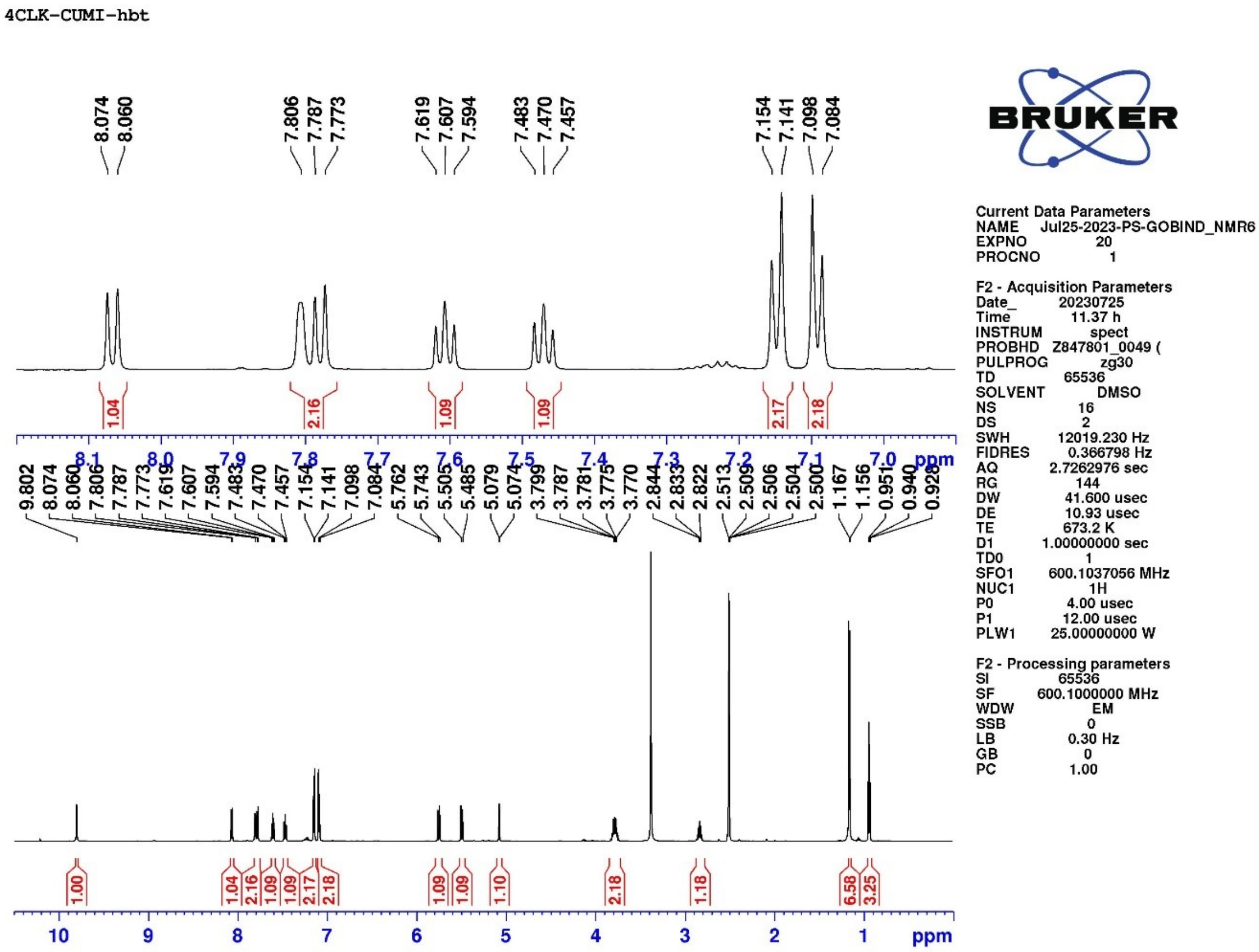

^{1}HNMR compound **11d**.

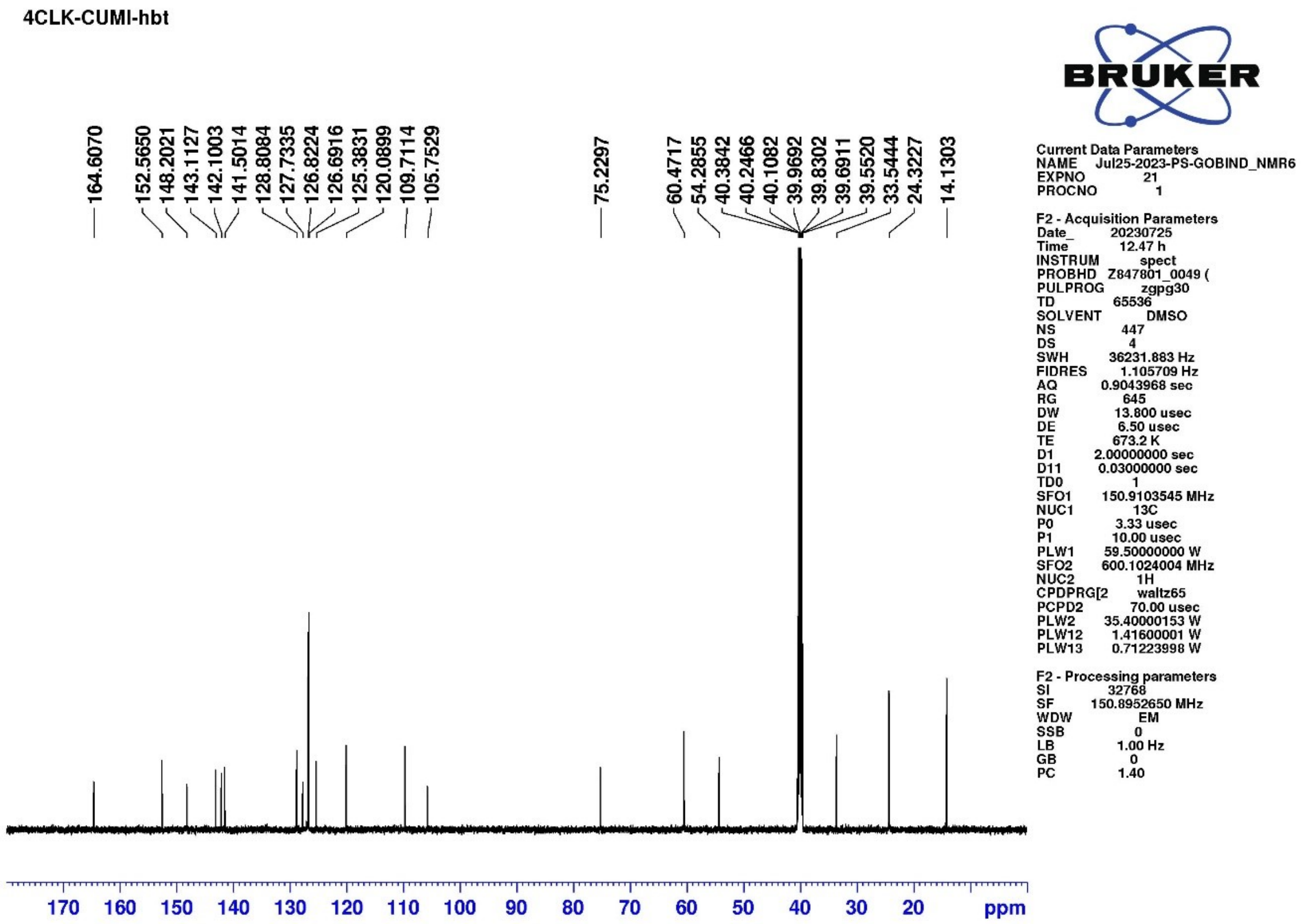

^{13}CNMR compound **11d**.

Single Mass Analysis
Tolerance = 5.0 PPM / DBE: min = -1.5, max = 50.0
Element prediction: Off
Number of isotope peaks used for i-FIT = 3

Monoisotopic Mass, Even Electron Ions
110 formula(e) evaluated with 1 results within limits (up to 50 closest results for each mass)
Elements Used:
C: 10-50 H: 10-100 N: 1-5 O: 1-5 Na: 1-1
H-10 163 (1.413) Cm (2:230)
TOF MS ES+

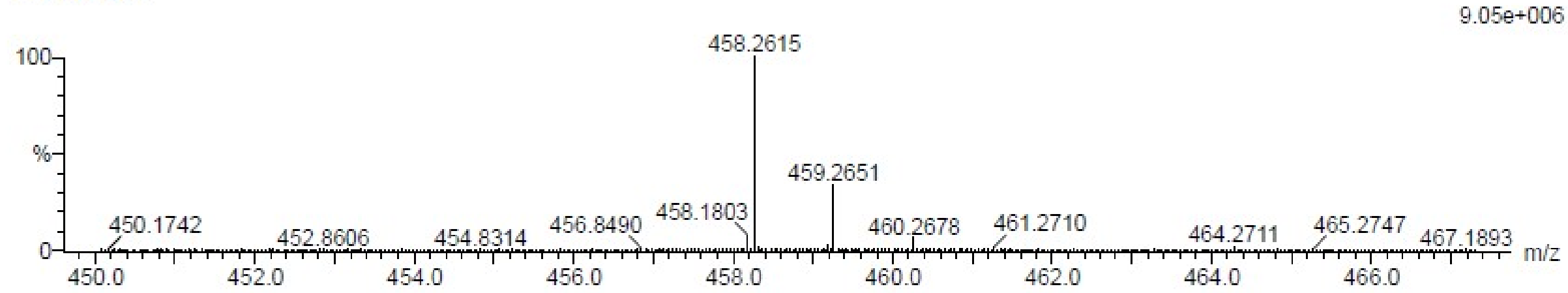

Minimum:				-1.5				
Maximum:		10.0	5.0	50.0				
Mass	Calc. Mass	mDa	PPM	DBE	i-FIT	Norm	Conf(%)	Formula
458.1803	458.1804	-0.1	-0.2	13.5	1311.7	n/a	n/a	C23 H25 N5 O4 Na

HRMS of compound **11d**.

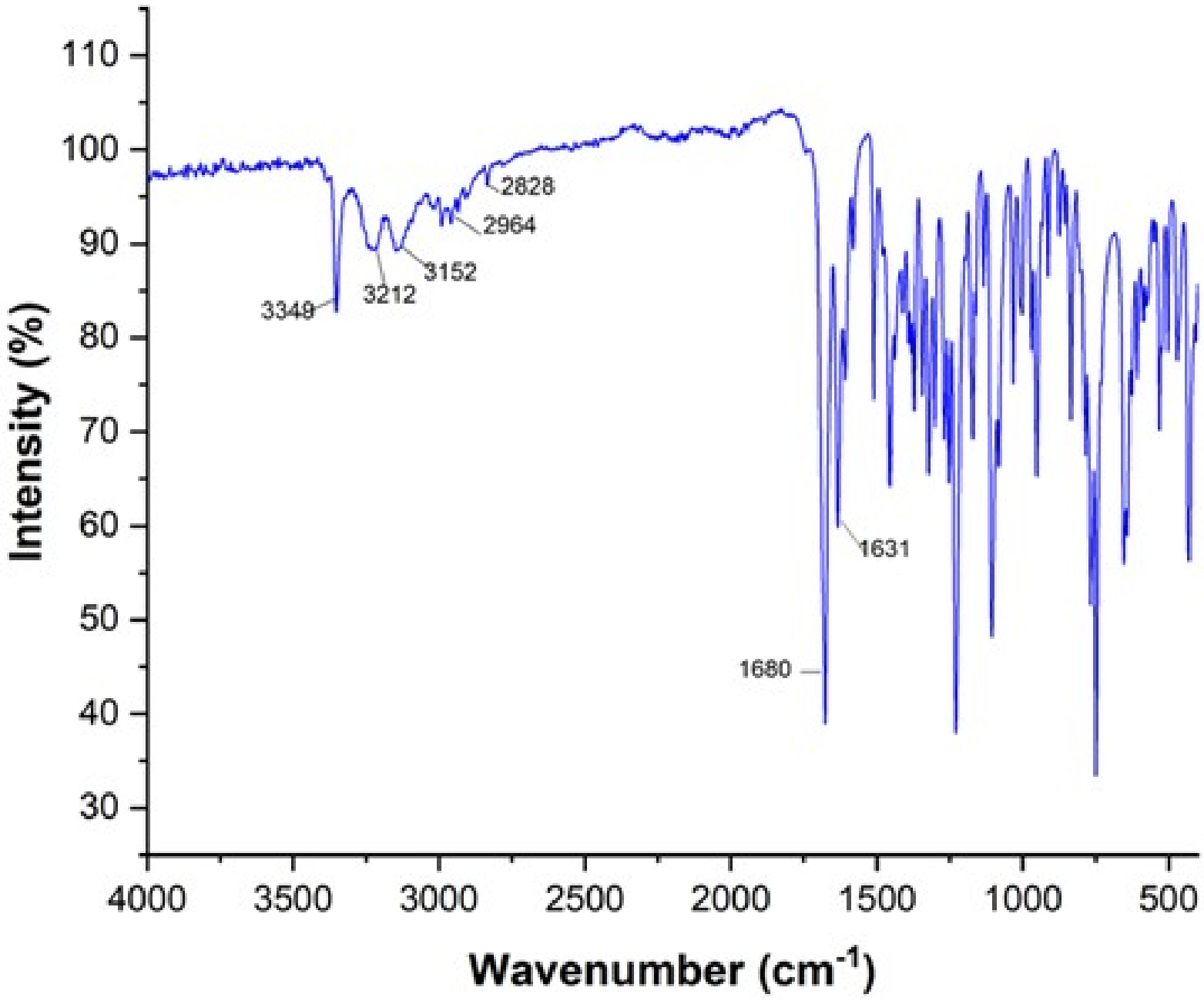

IR spectra of compound **11e.**

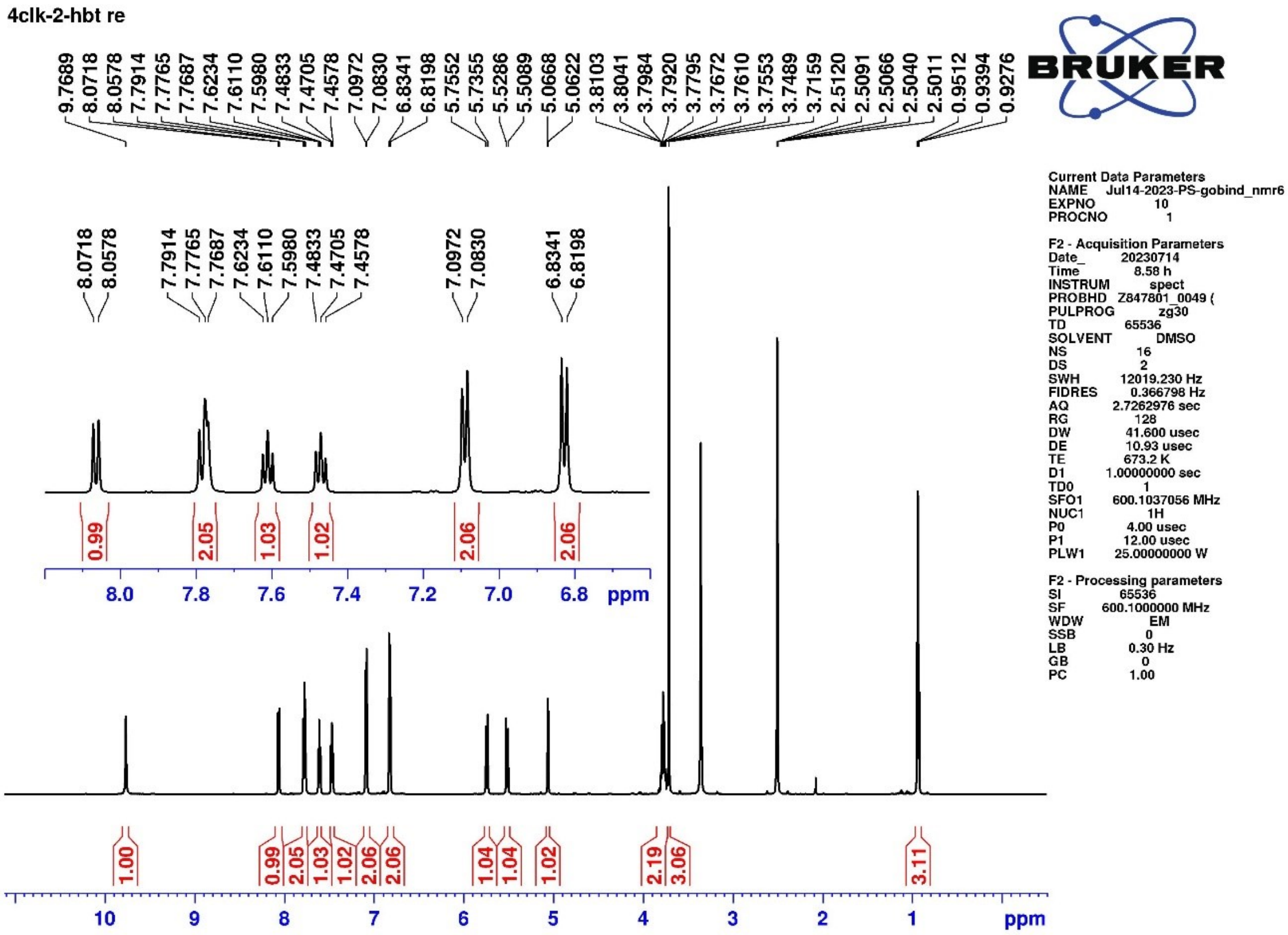

^{1}HNMR compound **11e**.

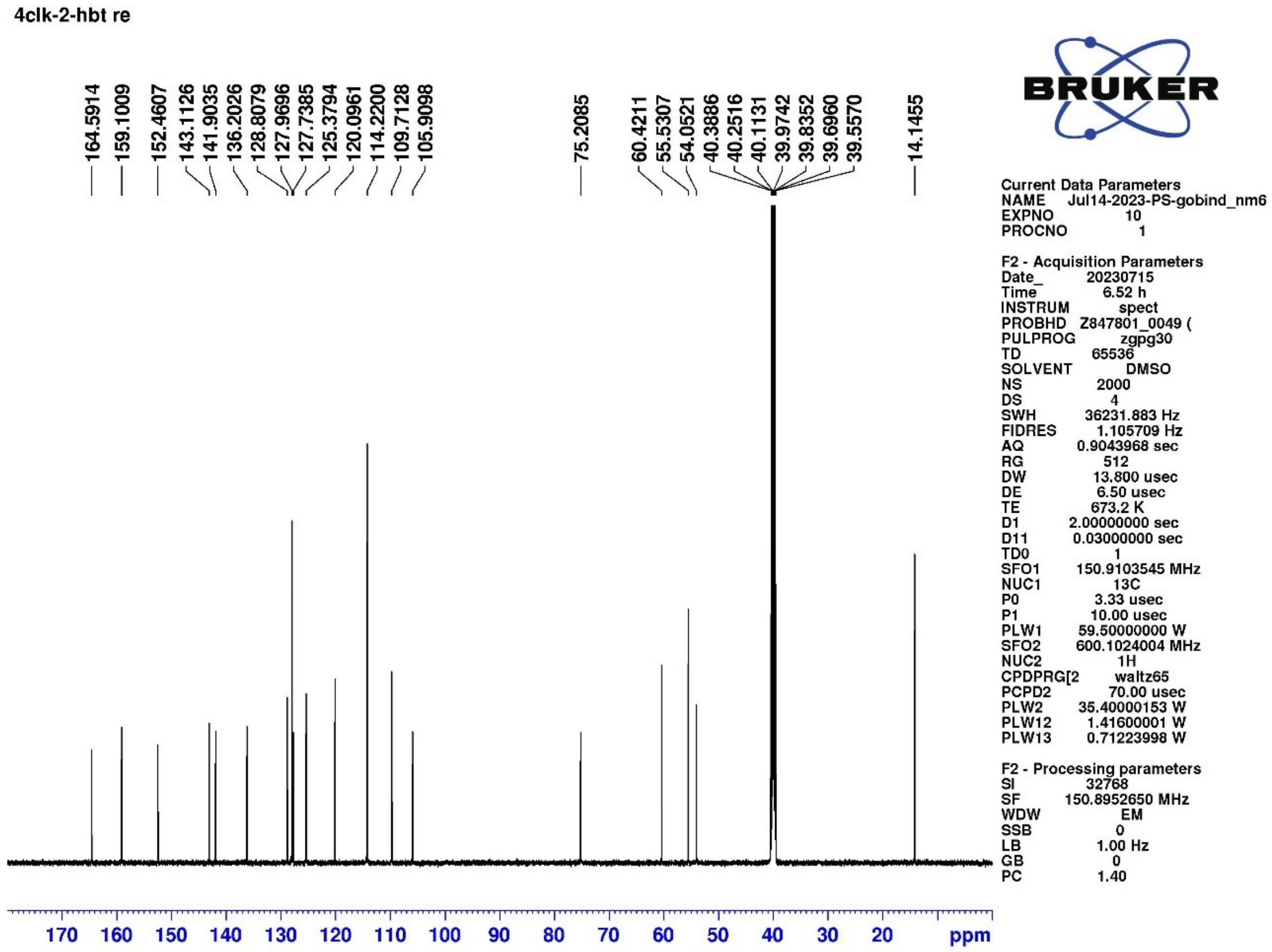

^{13}CNMR compound **11e**.

Single Mass Analysis
Tolerance = 5.0 PPM / DBE: min = -1.5, max = 50.0
Element prediction: Off
Number of isotope peaks used for i-FIT = 3

Monoisotopic Mass, Even Electron Ions
110 formula(e) evaluated with 1 results within limits (up to 50 closest results for each mass)
Elements Used:
C: 10-50 H: 10-100 N: 1-5 O: 1-5 Na: 1-1
H-3 222 (1.920) Cm (2:230)
TOF MS ES+

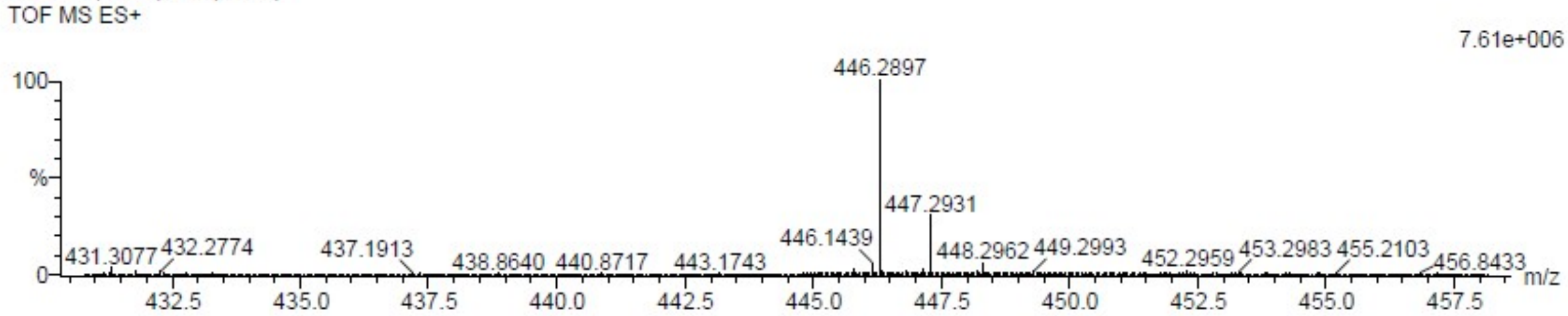

Minimum:				-1.5				
Maximum:		10.0	5.0	50.0				
Mass	Calc. Mass	mDa	PPM	DBE	i-FIT	Norm	Conf(%)	Formula
446.1439	446.1440	-0.1	-0.2	13.5	1258.7	n/a	n/a	C21 H21 N5 O5 Na

HRMS of compound **11e**.

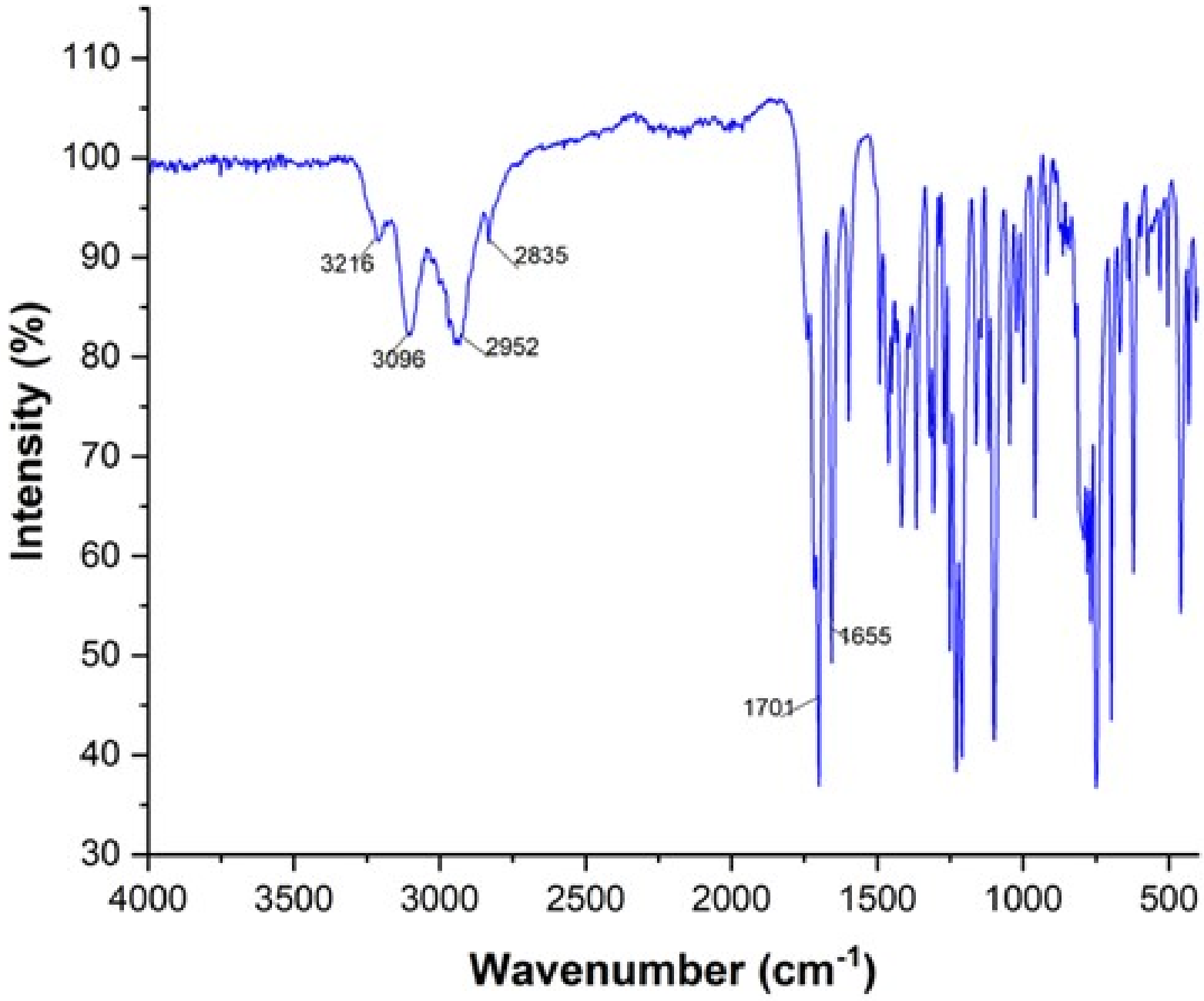

IR spectra of compound **11f.**

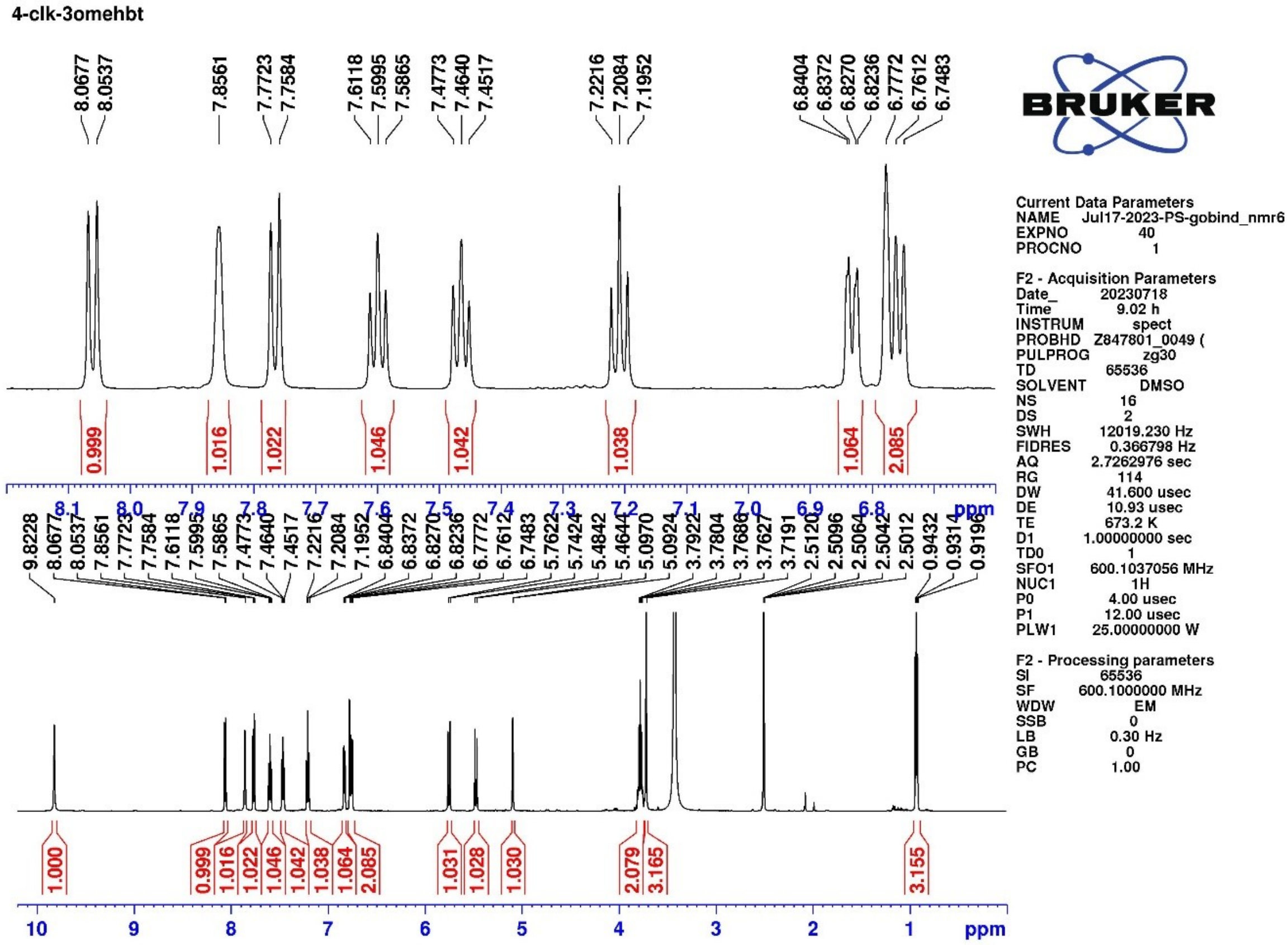

^{1}HNMR compound **11f**.

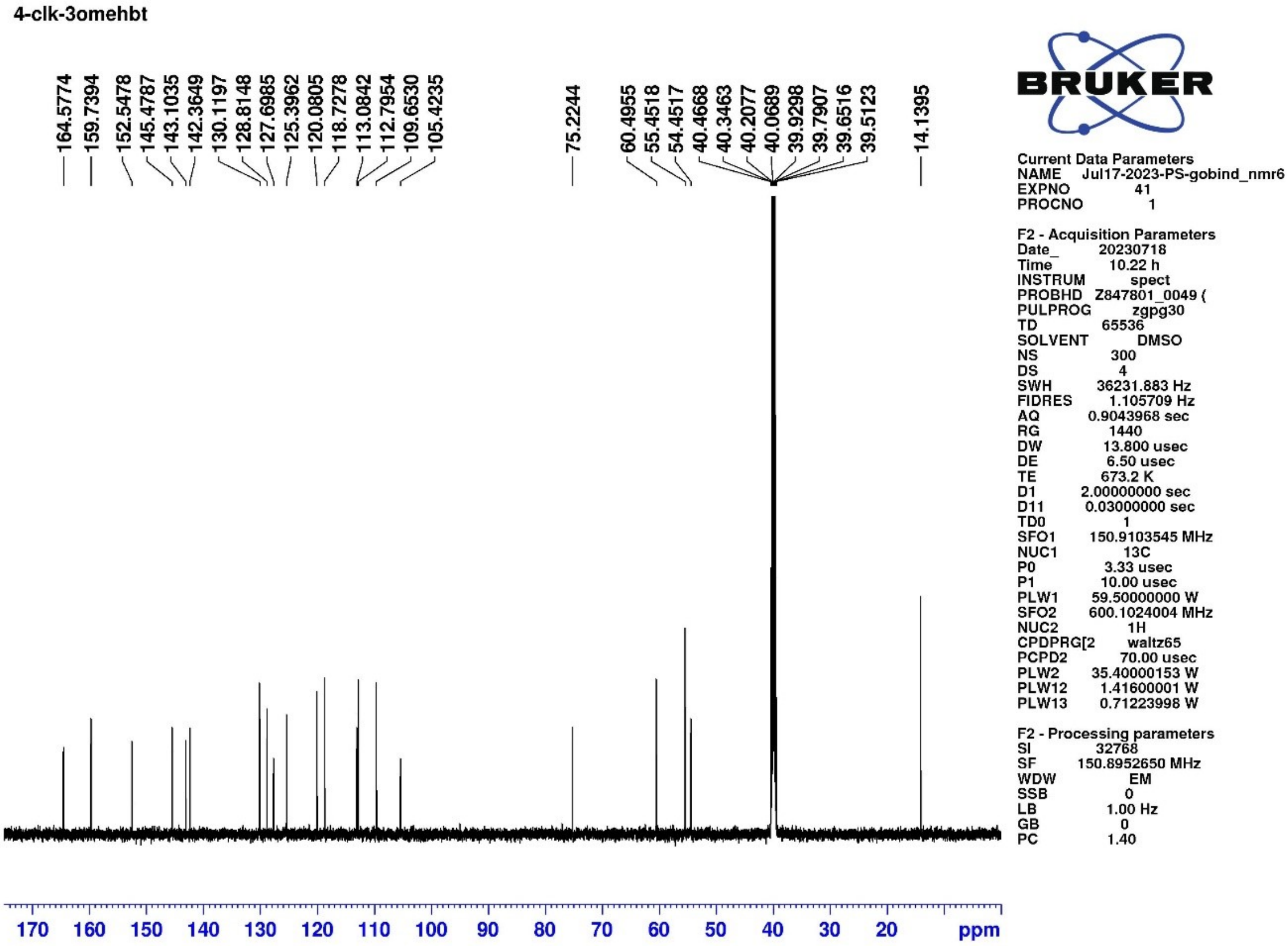

^{13}CNMR compound **11f**.

Single Mass Analysis

Tolerance = 5.0 PPM / DBE: min = -1.5, max = 50.0

Element prediction: Off

Number of isotope peaks used for i-FIT = 3

Monoisotopic Mass, Even Electron Ions

110 formula(e) evaluated with 1 results within limits (up to 50 closest results for each mass)

Elements Used:

C: 10-50 H: 10-100 N: 1-5 O: 1-5 Na: 1-1

H-9 222 (1.920) Cm (2:230)

TOF MS ES+

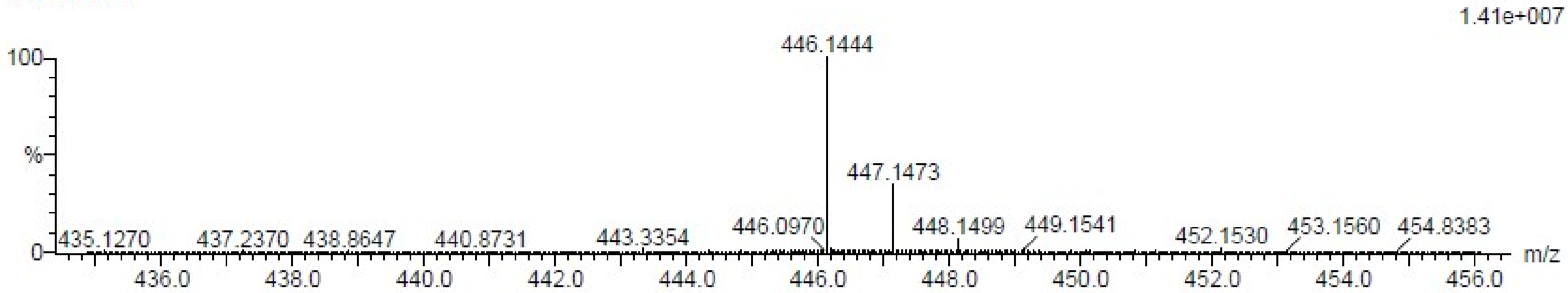

Minimum:				-1.5				
Maximum:		10.0	5.0	50.0				
Mass	Calc. Mass	mDa	PPM	DBE	i-FIT	Norm	Conf(%)	Formula
446.1444	446.1440	0.4	0.9	13.5	1315.7	n/a	n/a	C21 H21 N5 O5 Na

HRMS of compound **11f**.

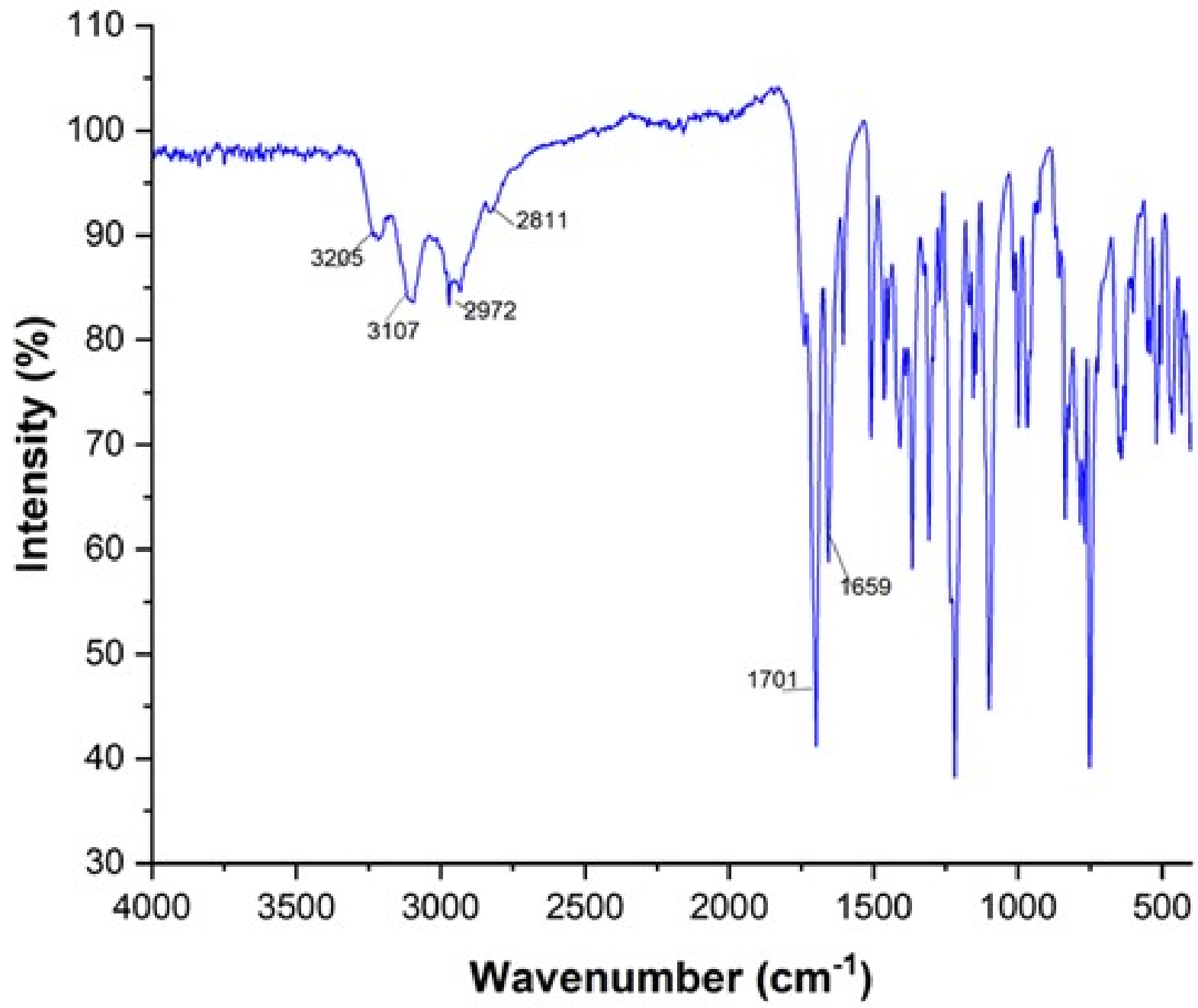

IR spectra of compound **11g**.

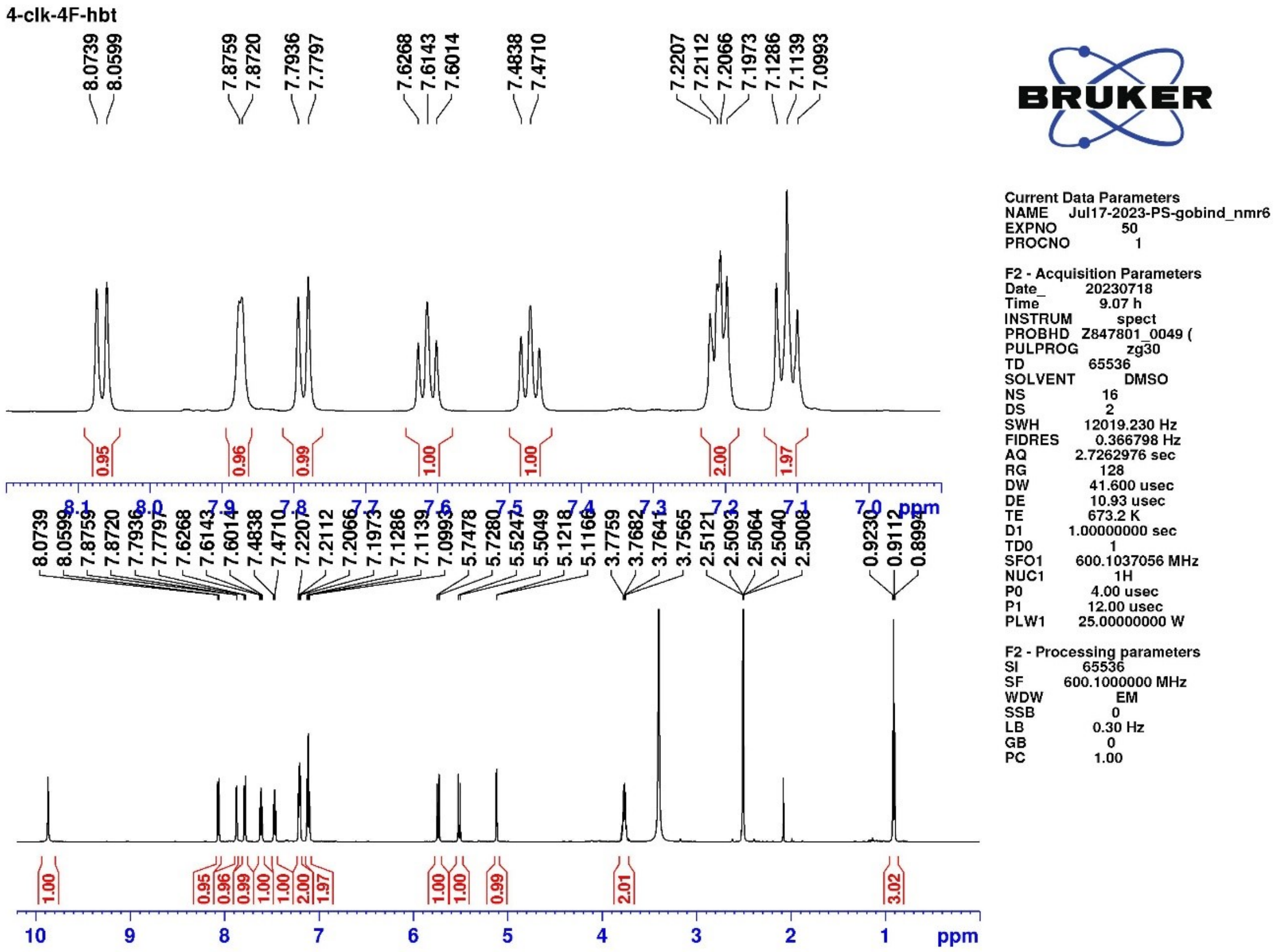

^{1}HNMR compound **11g**.

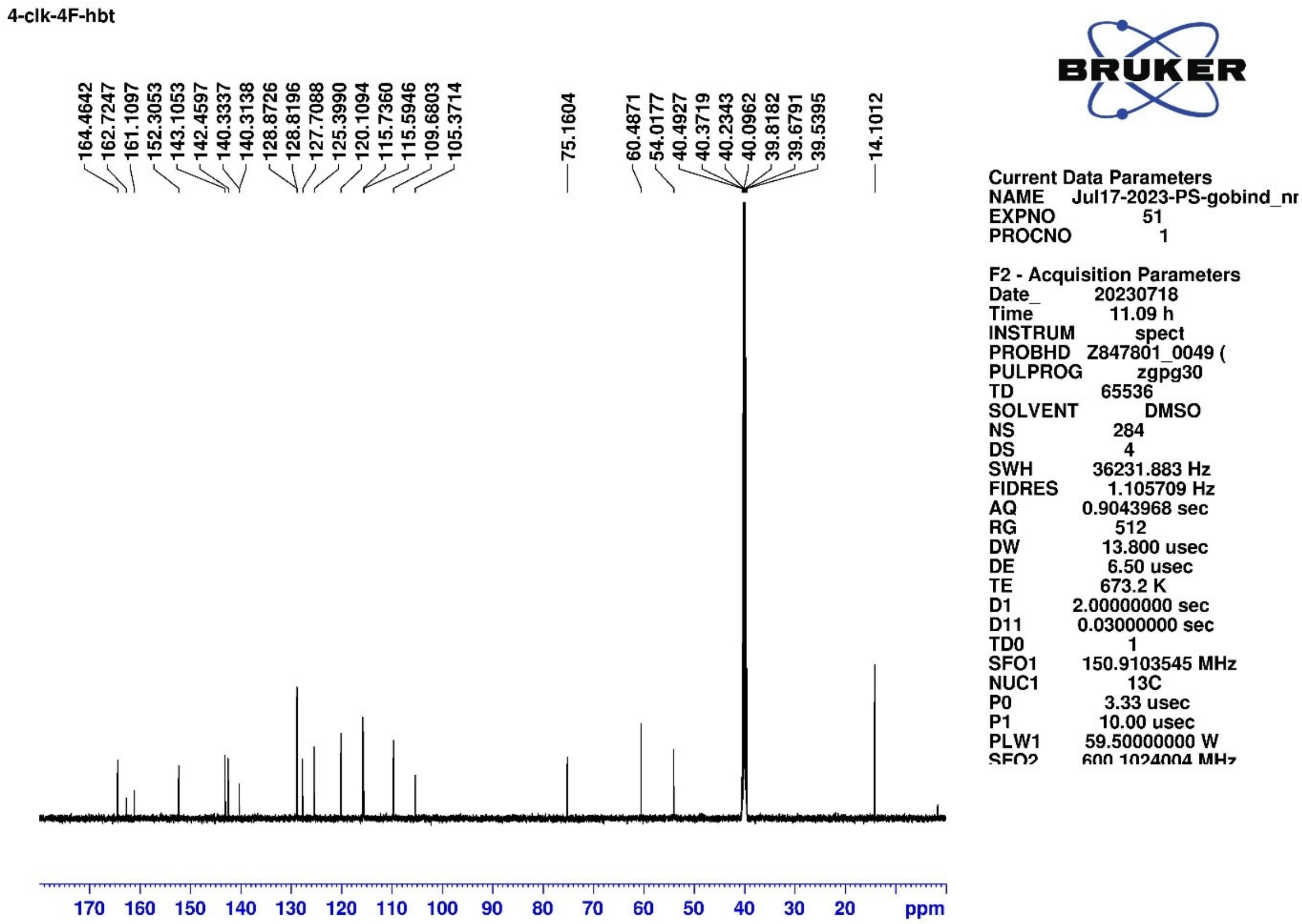

^{13}CNMR spectra of compound **11g**.

Single Mass Analysis
Tolerance = 5.0 PPM / DBE: min = -1.5, max = 50.0
Element prediction: Off
Number of isotope peaks used for i-FIT = 3

Monoisotopic Mass, Even Electron Ions
98 formula(e) evaluated with 1 results within limits (up to 50 closest results for each mass)
Elements Used:
C: 10-50 H: 10-100 N: 1-5 O: 1-5 Na: 1-1 F: 1-1

H-8 202 (1.748) Cm (2:230)
TOF MS ES+

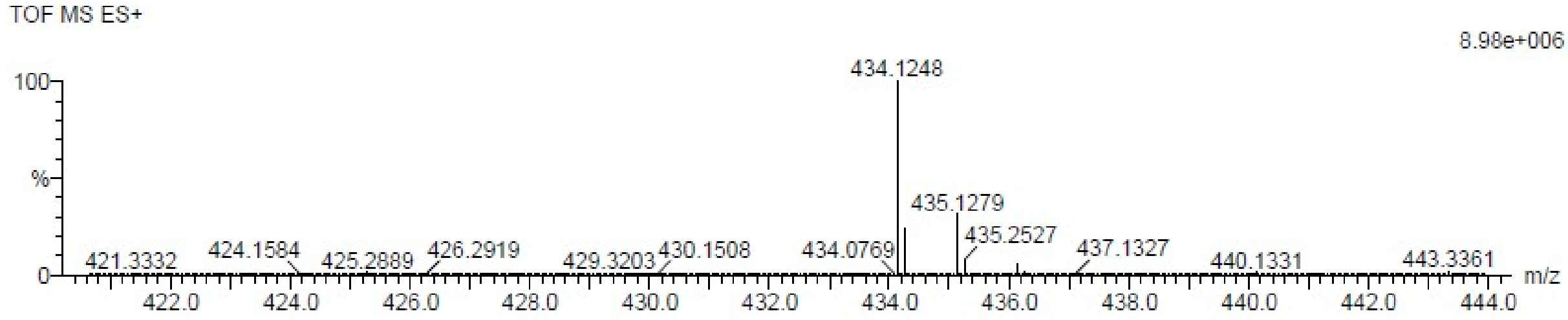

Minimum:				-1.5				
Maximum:		10.0	5.0	50.0				
Mass	Calc. Mass	mDa	PPM	DBE	i-FIT	Norm	Conf(%)	Formula
434.1248	434.1241	0.7	1.6	13.5	1225.6	n/a	n/a	C20 H18 N5 O4 Na F

HRMS of compound **11g**.

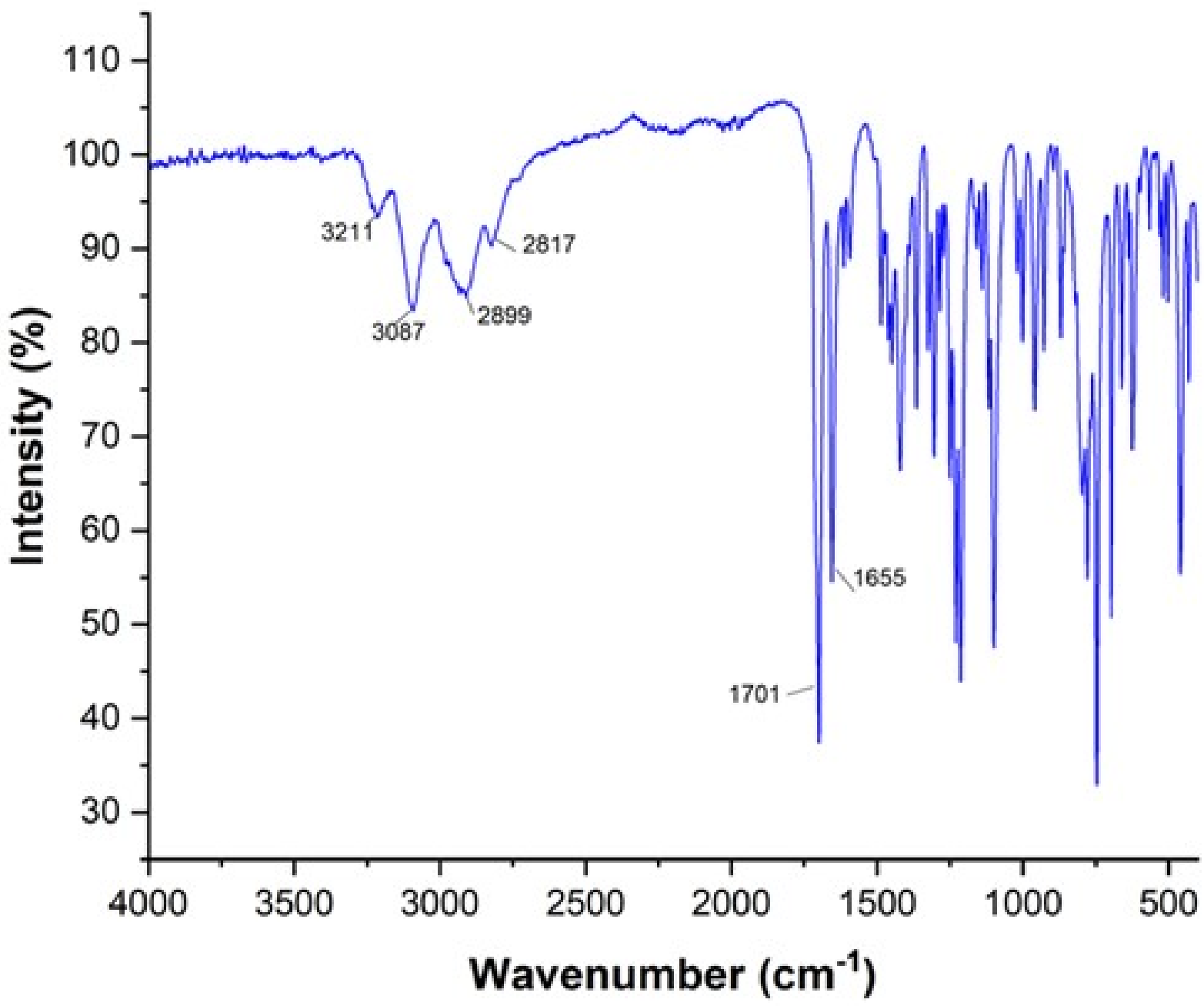

IR spectra of compound **11h**.

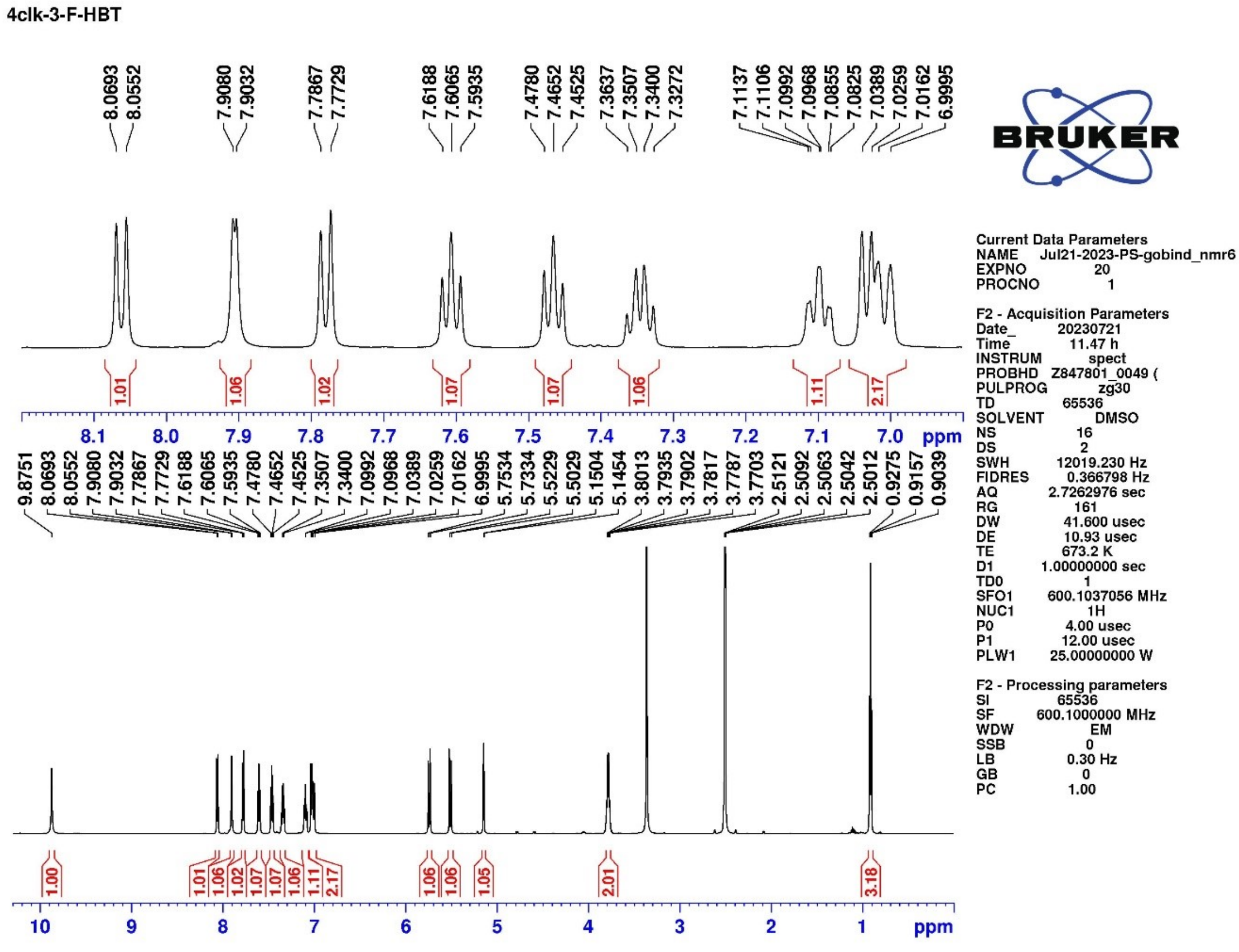

^{1}HNMR compound **11h**.

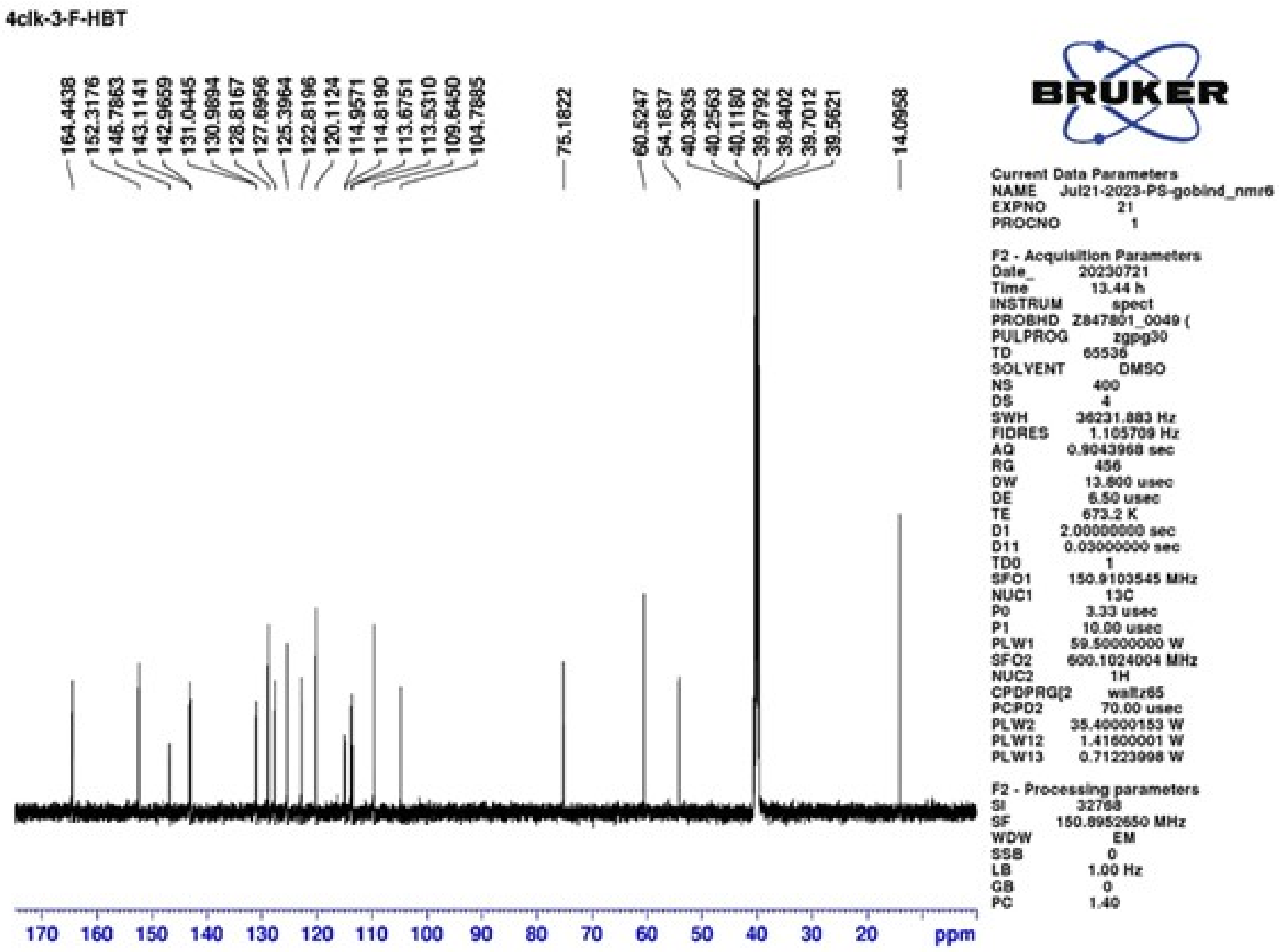

^{13}CNMR compound **11h**.

Single Mass Analysis
Tolerance = 5.0 PPM / DBE: min = -1.5, max = 50.0
Element prediction: Off
Number of isotope peaks used for i-FIT = 3

Monoisotopic Mass, Even Electron Ions
98 formula(e) evaluated with 1 results within limits (up to 50 closest results for each mass)
Elements Used:
C: 10-50 H: 10-100 N: 1-5 O: 1-5 Na: 1-1 F: 1-1

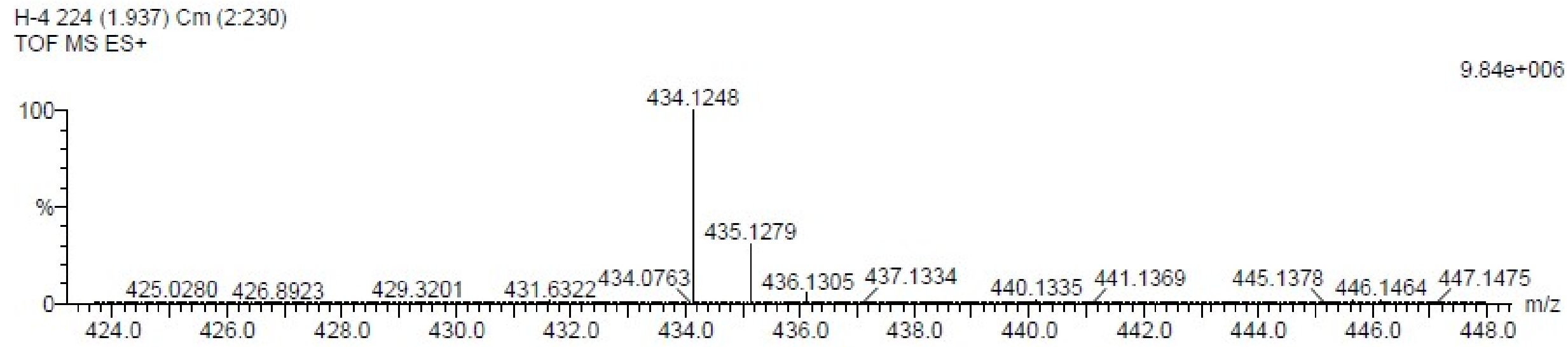

Minimum:				-1.5				
Maximum:		10.0	5.0	50.0				
Mass	Calc. Mass	mDa	PPM	DBE	i-FIT	Norm	Conf(%)	Formula
434.1248	434.1241	0.7	1.6	13.5	1228.4	n/a	n/a	C20 H18 N5 O4 Na F

HRMS of compound **11h**.

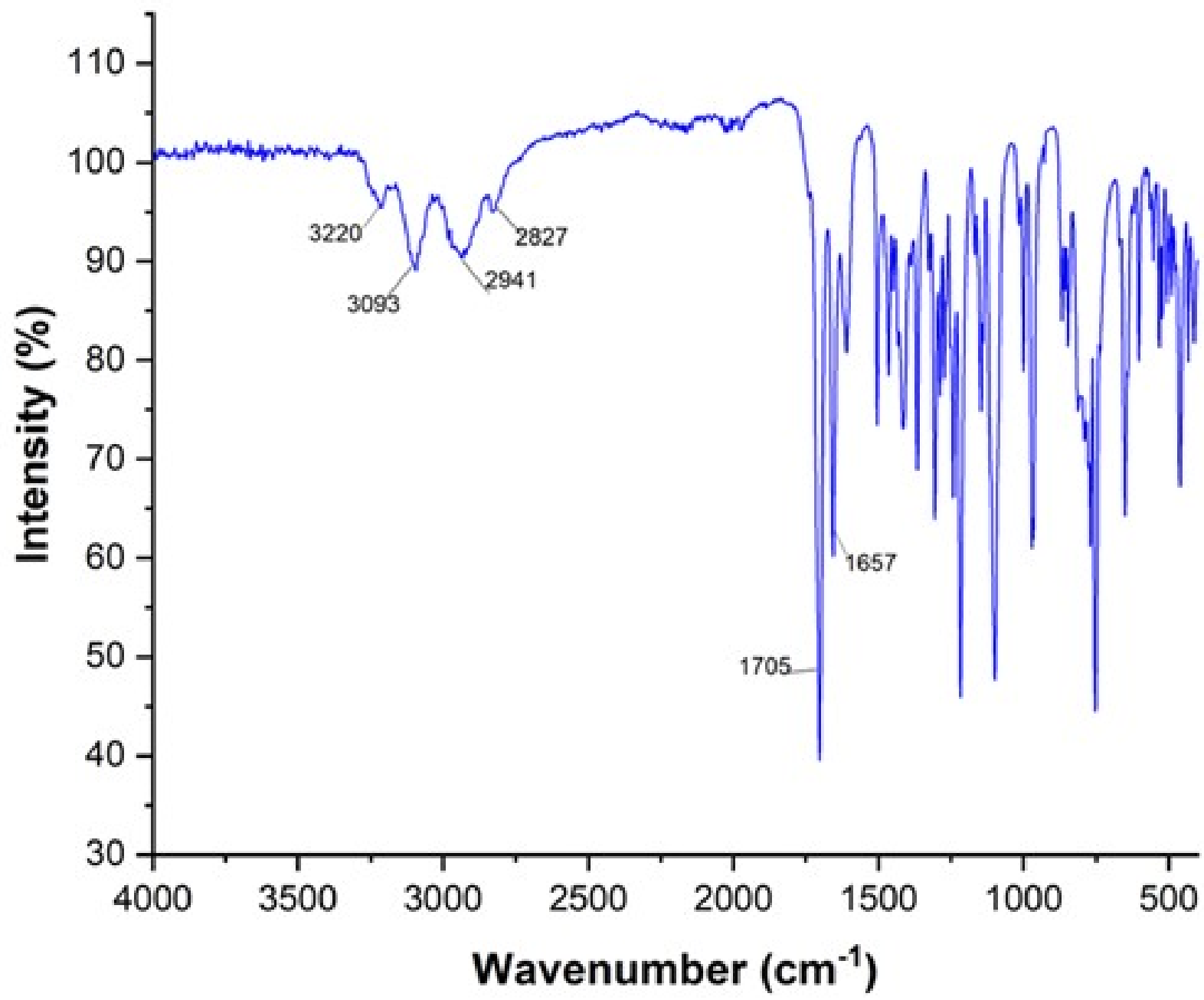

IR spectra of compound **11i.**

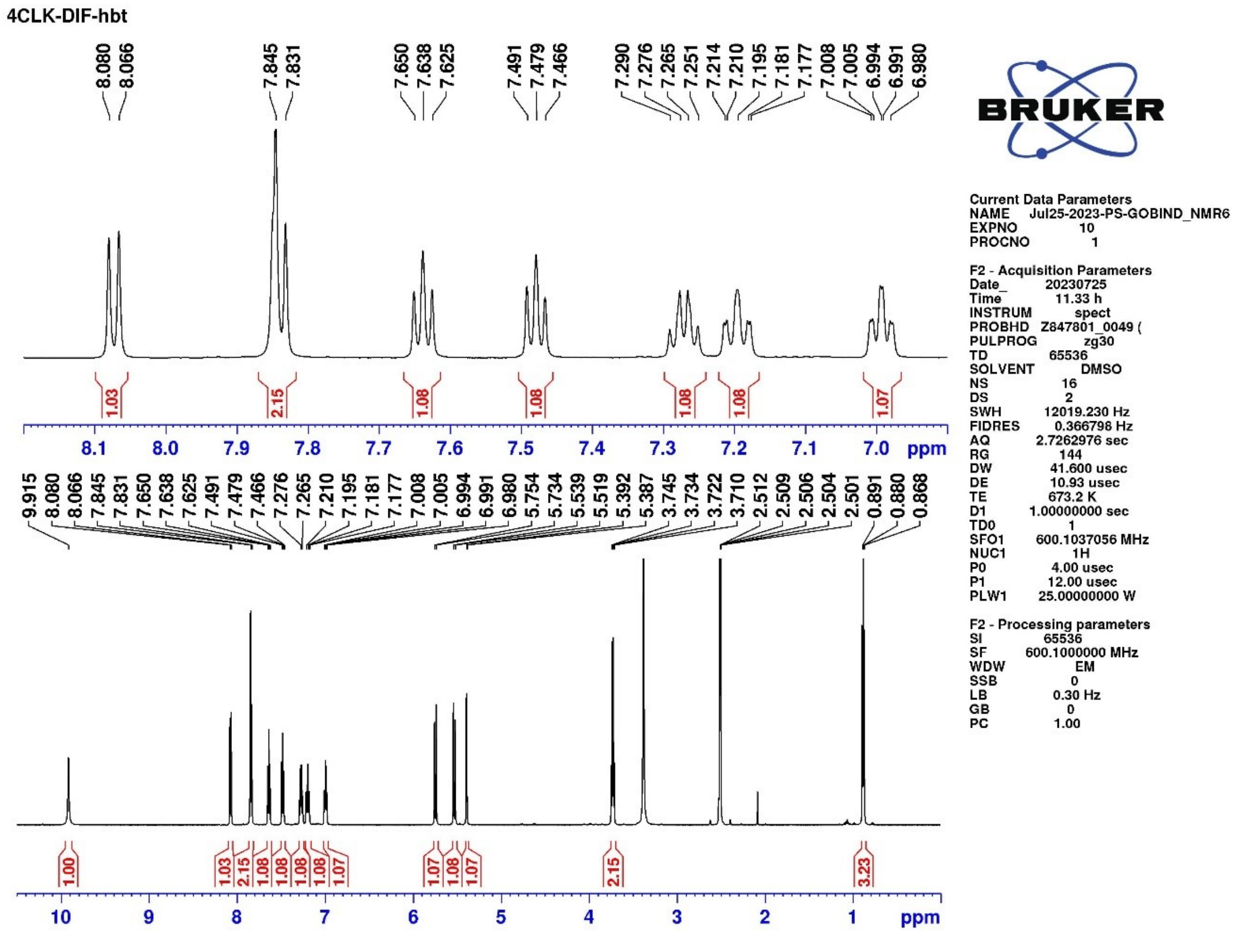

^{1}HNMR compound **11i**.

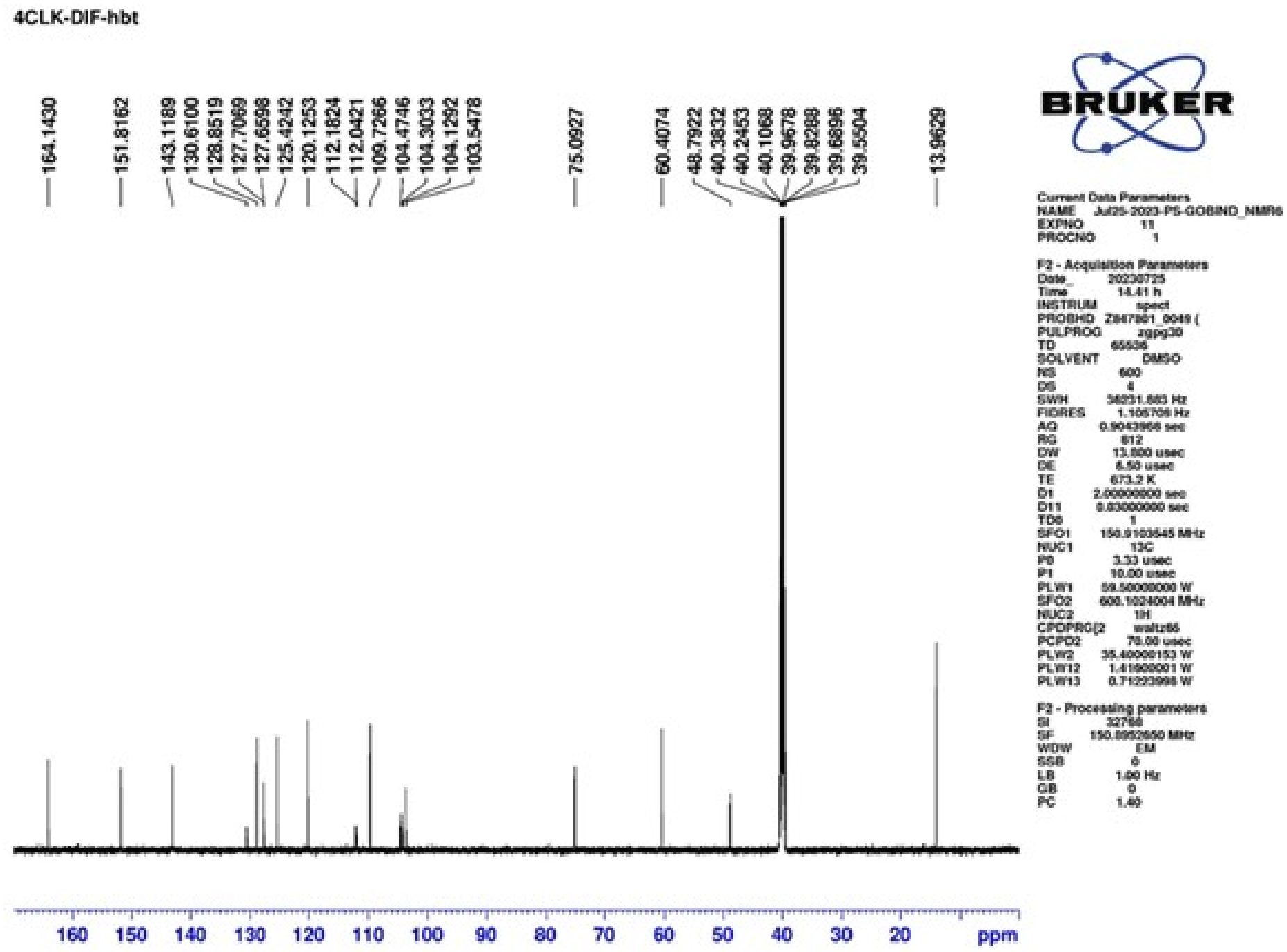

^{13}CNMR compound **11i**.

Single Mass Analysis
Tolerance = 5.0 PPM / DBE: min = -1.5, max = 50.0
Element prediction: Off
Number of isotope peaks used for i-FIT = 3

Monoisotopic Mass, Even Electron Ions
208 formula(e) evaluated with 2 results within limits (up to 50 closest results for each mass)
Elements Used:
C: 10-50 H: 10-100 N: 1-5 O: 1-5 F: 1-2 Na: 1-1
H-5 122 (1.062) Cm (2:230)
TOF MS ES+

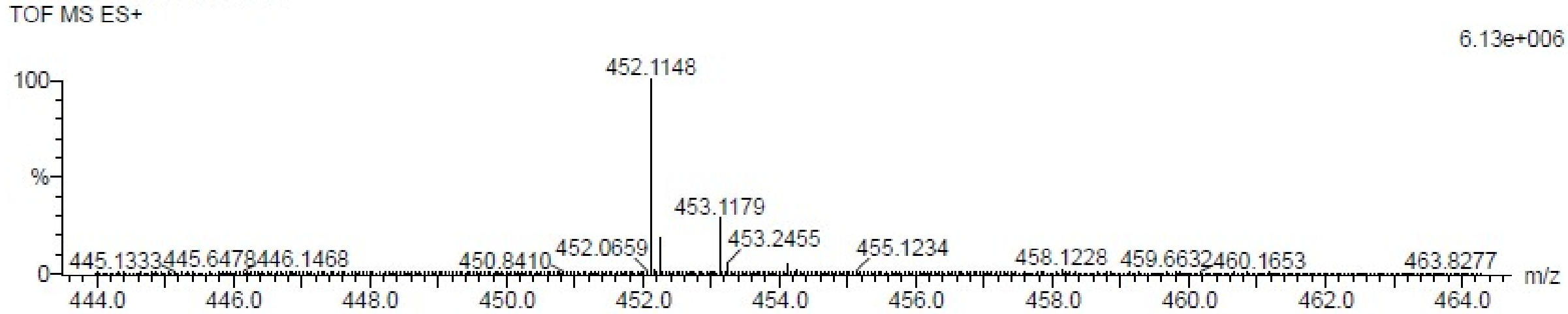

Minimum:				-1.5				
Maximum:		10.0	5.0	50.0				
Mass	Calc. Mass	mDa	PPM	DBE	i-FIT	Norm	Conf(%)	Formula
452.1148	452.1146	0.2	0.4	13.5	1286.8	2.923	5.38	C20 H17 N5 O4 F2 Na
	452.1135	1.3	2.9	17.5	1283.9	0.055	94.62	C23 H16 N5 O3 F Na

HRMS of compound **11i**.

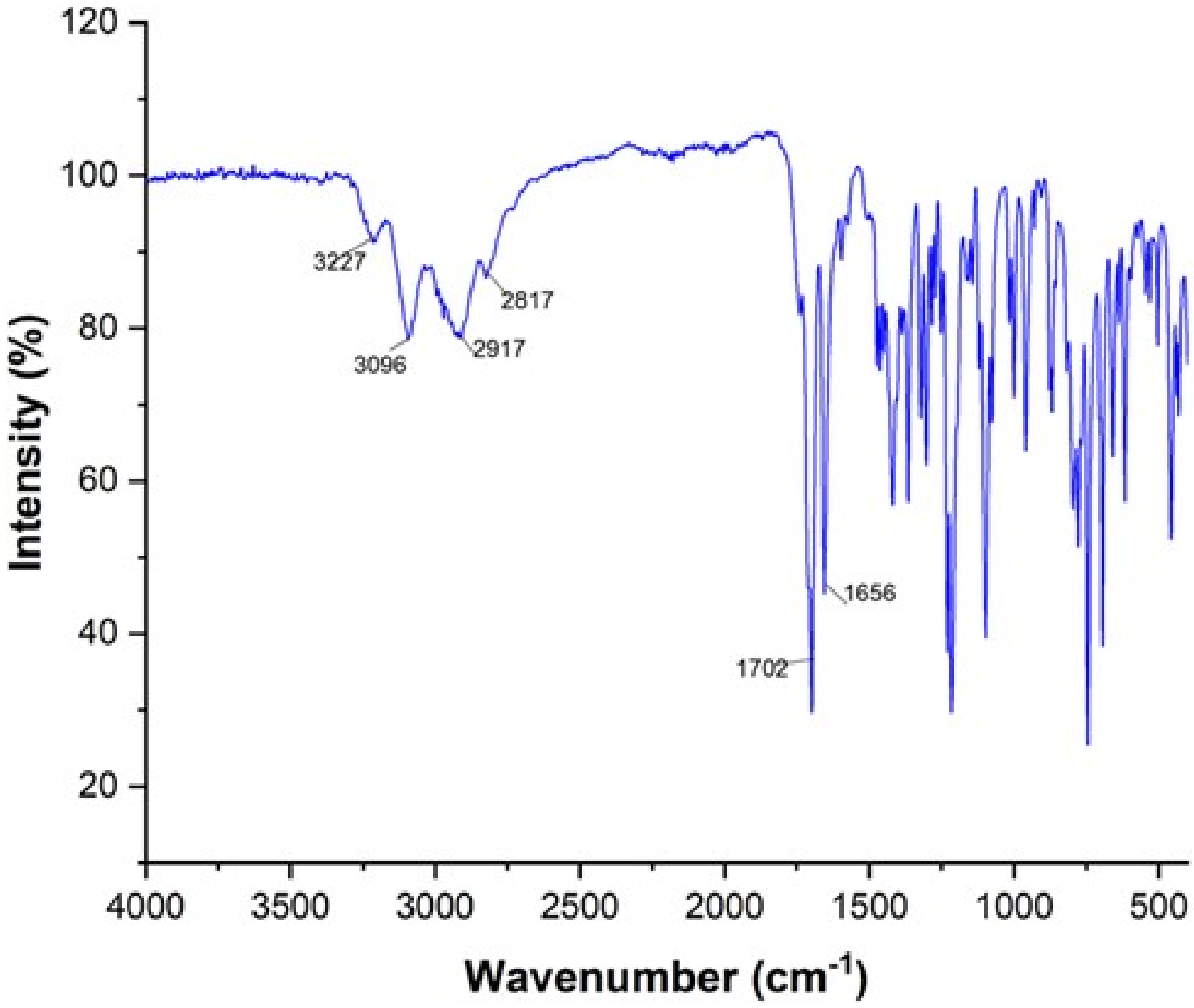

IR spectra of compound **11j.**

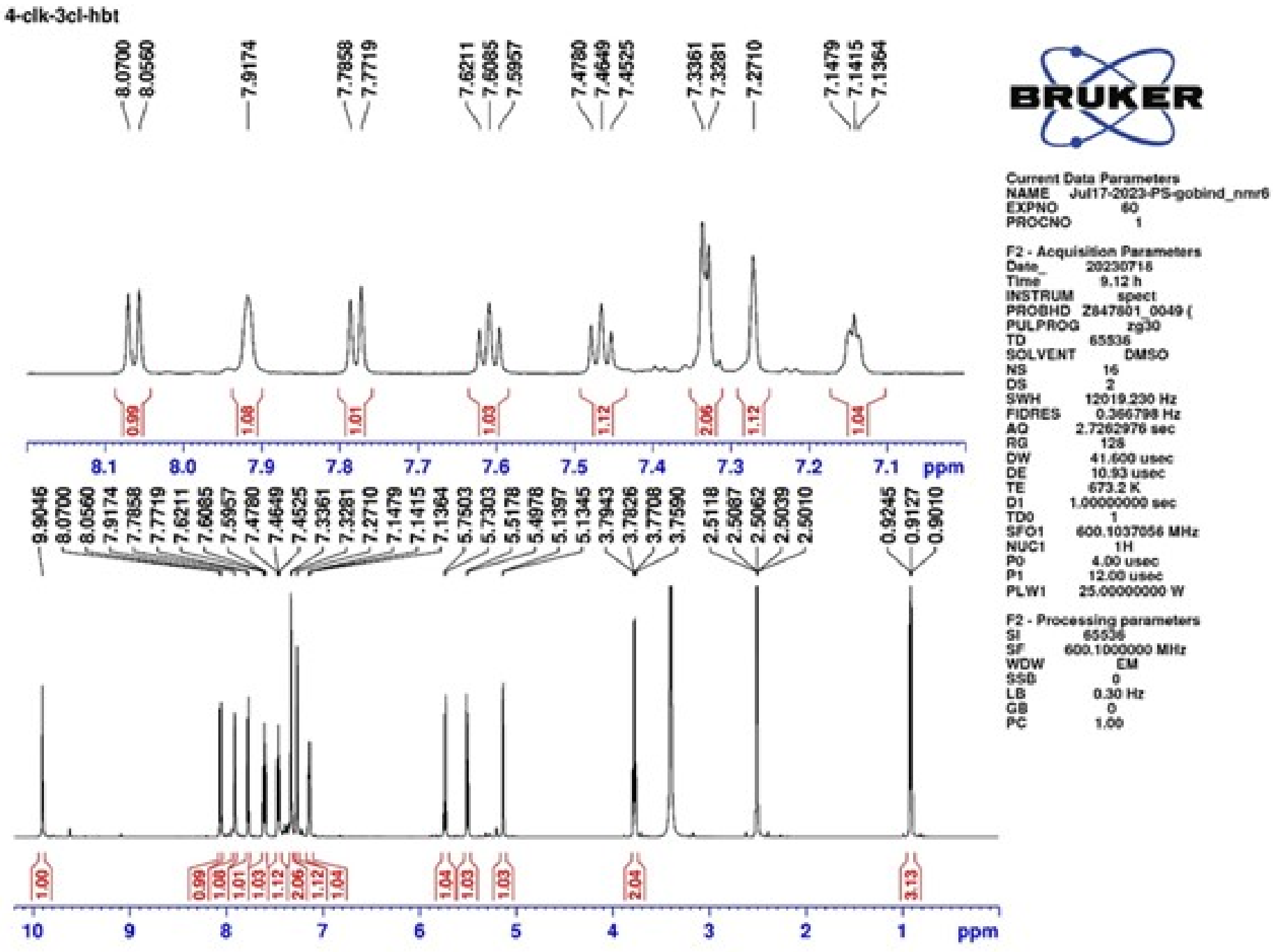

^{1}HNMR compound **11j**.

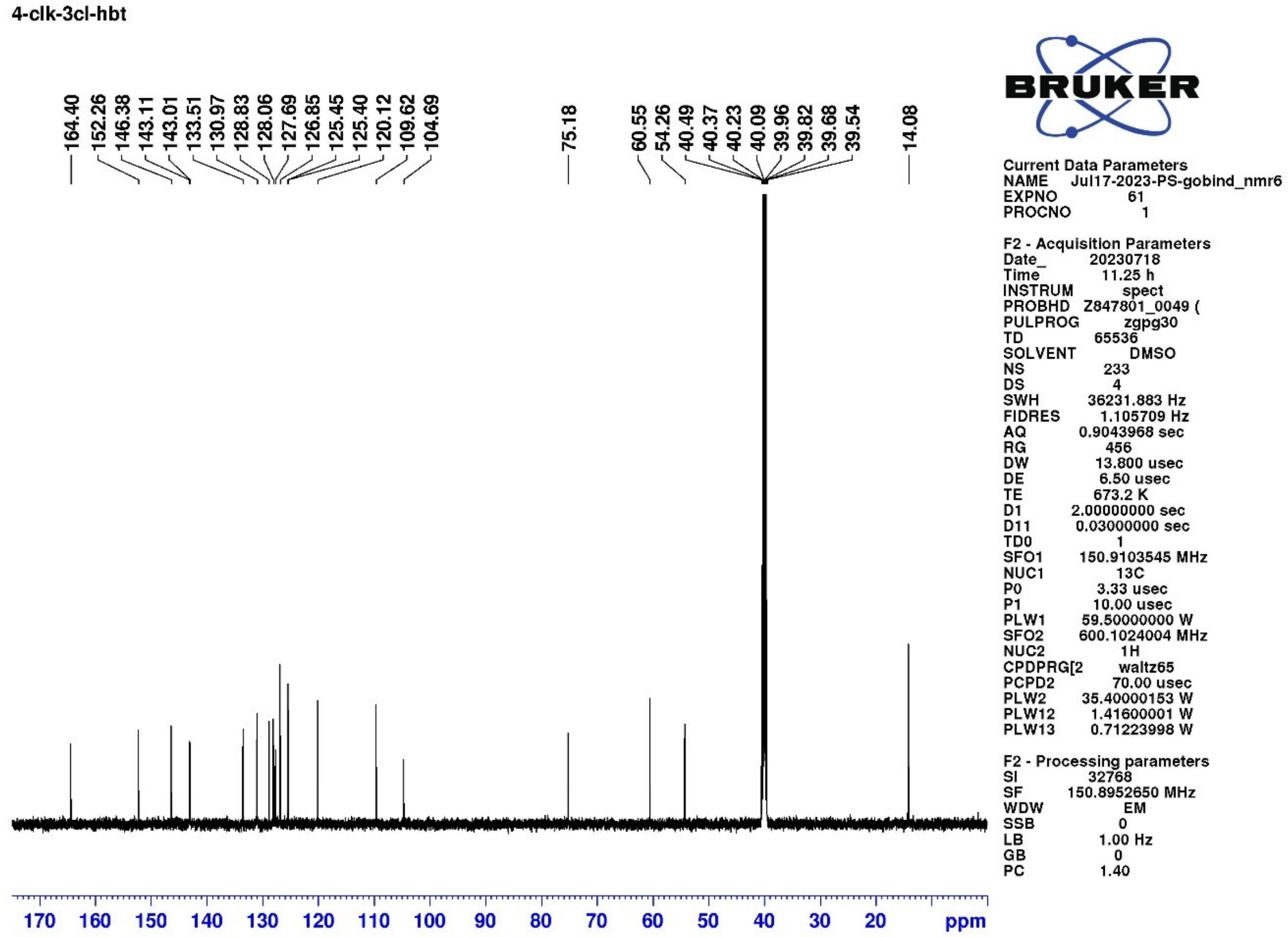

^{13}CNMR compound **11j**.

Single Mass Analysis
Tolerance = 5.0 PPM / DBE: min = -1.5, max = 50.0
Element prediction: Off
Number of isotope peaks used for i-FIT = 3

Monoisotopic Mass, Even Electron Ions
98 formula(e) evaluated with 1 results within limits (up to 50 closest results for each mass)
Elements Used:
C: 10-50 H: 10-100 N: 1-5 O: 1-5 Na: 1-1 Cl: 1-1

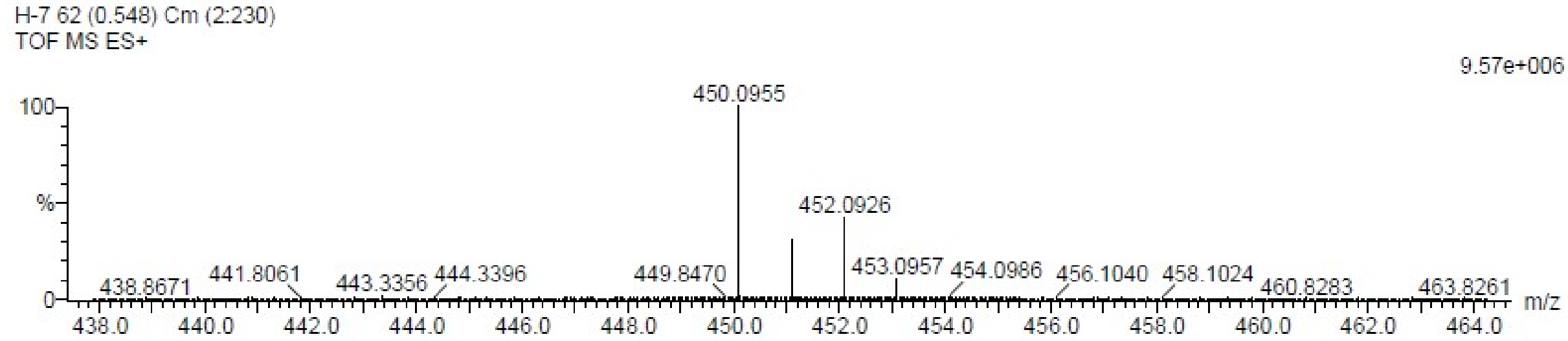

Minimum:				-1.5				
Maximum:		10.0	5.0	50.0				
Mass	Calc. Mass	mDa	PPM	DBE	i-FIT	Norm	Conf(%)	Formula
450.0955	450.0945	1.0	2.2	13.5	1336.4	n/a	n/a	C20 H18 N5 O4 Na Cl

HRMS of compound **11j**.

References

(1) Yakkala, P. A.; Panda, S. R.; Naidu, V. G.; Shafi, S.; Kamal, A. Pyridine-based 1, 2, 4-triazolo-tethered indole conjugates potentially affecting TNKS and PI3K in colorectal cancer. *ACS Medicinal Chemistry Letters* **2023**, *14* (3), 260–269.

(2) Ma, X.; Wang, J.; Zhao, Y. Synthesis of Benzotriazole by o-Nitrochlorobenzene. *FINE CHEMICALS-DALIAN-* **2006**, *23* (7), 696.

(3) Kumar, G.; Seboletswe, P.; Mishra, S.; Manhas, N.; Ghumran, S.; Kerru, N.; Roquet-Banères, F.; Foubert, M.; Kremer, L.; Bhargava, G. Isoniazid-Dihydropyrimidinone Molecular Hybrids: Design, Synthesis, Antitubercular Activity, and Cytotoxicity Investigations with Computational Validation. *ChemMedChem* **2025**, *20* (11), e202400949.
